# Genomics of Divergence along a Continuum of Parapatric Population Differentiation

**DOI:** 10.1371/journal.pgen.1004966

**Published:** 2015-02-13

**Authors:** Philine G. D. Feulner, Frédéric J. J. Chain, Mahesh Panchal, Yun Huang, Christophe Eizaguirre, Martin Kalbe, Tobias L. Lenz, Irene E. Samonte, Monika Stoll, Erich Bornberg-Bauer, Thorsten B. H. Reusch, Manfred Milinski

**Affiliations:** 1 Department of Evolutionary Ecology, Max Planck Institute for Evolutionary Biology, Plön, Germany; 2 Institute for Evolution and Biodiversity, Evolutionary Bioinformatics, Westfälische Wilhelms University, Münster, Germany; 3 School of Biological and Chemical Sciences, Queen Mary University of London, London, United Kingdom; 4 Genetic Epidemiology, Westfälische Wilhelms University, Münster, Germany; 5 Evolutionary Ecology of Marine Fishes, GEOMAR Helmholtz Centre for Ocean Research Kiel, Kiel, Germany; University of Michigan, UNITED STATES

## Abstract

The patterns of genomic divergence during ecological speciation are shaped by a combination of evolutionary forces. Processes such as genetic drift, local reduction of gene flow around genes causing reproductive isolation, hitchhiking around selected variants, variation in recombination and mutation rates are all factors that can contribute to the heterogeneity of genomic divergence. On the basis of 60 fully sequenced three-spined stickleback genomes, we explore these different mechanisms explaining the heterogeneity of genomic divergence across five parapatric lake and river population pairs varying in their degree of genetic differentiation. We find that divergent regions of the genome are mostly specific for each population pair, while their size and abundance are not correlated with the extent of genome-wide population differentiation. In each pair-wise comparison, an analysis of allele frequency spectra reveals that 25–55% of the divergent regions are consistent with a local restriction of gene flow. Another large proportion of divergent regions (38–75%) appears to be mainly shaped by hitchhiking effects around positively selected variants. We provide empirical evidence that alternative mechanisms determining the evolution of genomic patterns of divergence are not mutually exclusive, but rather act in concert to shape the genome during population differentiation, a first necessary step towards ecological speciation.

## Authors Summary

A variety of evolutionary forces influence the genomic landscape of divergence during ecological speciation. Here we characterize the evolution of genomic divergence patterns based on 60 fully sequenced three-spined stickleback genomes, contrasting lake and river populations that differ in parasite abundance. Our comparison of the size and abundance of divergent regions in the genomes across a continuum of population differentiation suggests that selection and the hitchhiking effect on neutral sites mainly contributes to the observed heterogeneous patterns of genomic divergence. Additional divergent regions of the genome can be explained by a local reduction of gene flow. Our description of genomic divergence patterns across a continuum of population differentiation combined with an analysis of molecular signatures of evolution highlights how adaptation shapes the differentiation of sticklebacks in freshwater habitats.

## Introduction

During ecological speciation, divergence along the genome has been observed to be heterogeneous in numerous taxonomic groups [e.g., [1–4]]. Typically, the average genome-wide divergence is low, interspersed with regions of exceptional differentiation. However, studies describing divergence patterns across the genome have found regions of exceptional differentiation to be either numerous and small [4] or few and large [5, 6], the latter sometimes referred to as ‘genomic islands’. A variety of explanations have been proposed for the observed heterogeneity in genomic divergence, including stochastic processes such as genetic drift, but also deterministic mechanisms such as locus-specific reduction of gene flow in the vicinity of genes causing reproductive isolation, hitchhiking around selected variants, or variation in recombination and mutation rates [7]. Generally, genetic drift, population expansion, migration, and other demographic events affect the whole genome, whereas natural selection modified by local environmental differences impact only those regions of the genome that affect the respective phenotypes and fitness.

It is not known whether or not genomic patterns such as the variation of divergence and recombination along the genome tend to follow a predictable evolutionary trajectory as populations proceed along a speciation continuum [7]. We investigated the early phase of divergence using lake-river stickleback population pairs varying in their degree of genetic differentiation. If divergence patterns are driven by locus-specific effects of gene flow and divergent selection, the extent of divergence is expected to be more localized than widespread, in line with the “island view” [6]. These regions might hold “speciation genes” maintaining reproductive isolation between species including genes underlying a fitness reduction in hybrids [8]. Furthermore, “divergence hitchhiking”, the accumulative effect of selectively advantageous loci, predicts a positive correlation between genomic divergence and island size progression [9]. An alternative explanation posits that the lack of differentiation across most of the genome is due to shared ancestral polymorphism rather than ongoing gene flow [10, 11], whereas regions of high differentiation represent regions influenced by selection at linked sites [12]. Such a hitchhiking pattern may be caused by both advantageous (positive selection) and deleterious alleles (background selection). Therefore, if adaptation alone (assuming some degree of geographic separation) shapes the genomic landscape, population genetic processes unrelated to the extent of overall genomic differentiation govern divergence patterns. Disentangling such alternative scenarios is a crucial yet challenging step in understanding the genomics of divergence, especially in parapatry where the current and historic extent of migration and gene flow contribute to the overall genomic patterns.

**Figure 1:**
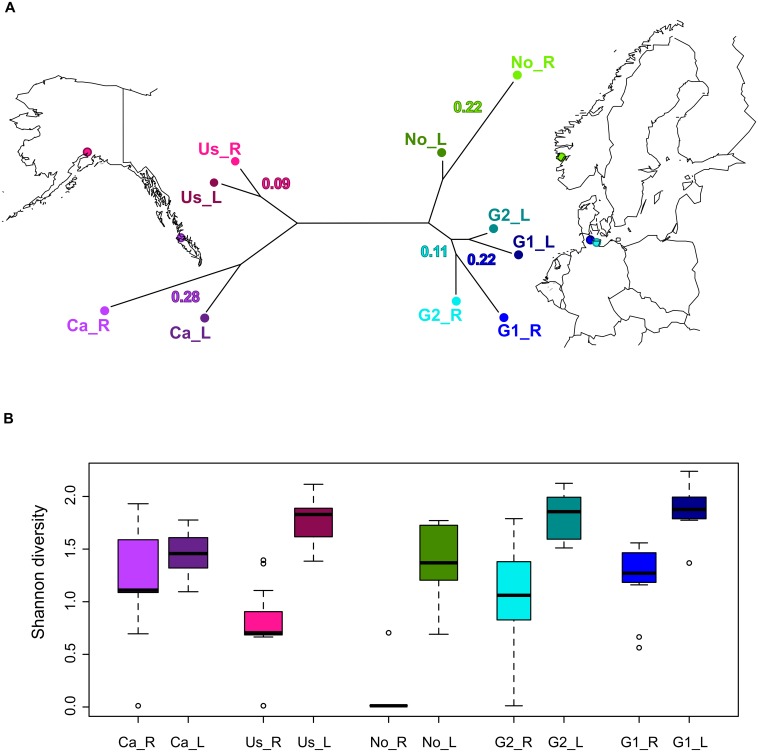
A Geographic map showing sampling locations including a neighbour-joining tree illustrating the genetic differentiation between sampled populations. Pairwise F_ST_ between parapatric lake-river populations is given on the tree. All nodes on the tree are supported by 100% bootstrap values. For sampling location labels see text and S1 Table. B Boxplot showing differences in parasite diversity across populations. Boxplots represent the distribution of Shannon diversity indices for each fish per population (n = 12–17) in which parasite counts were 4^th^ square root transformed. Except for Canada, all parapatric populations show significant differences in parasite diversity (Mann-Whitney, W1–5, P ≤ 0.001; Ca: W = 106, P = 0.292).

We tested predictions inherent to the different scenarios explaining genomic patterns of divergence using whole-genome sequencing data of replicated population pairs of three-spined sticklebacks varying in their degree of genetic differentiation. Five population pairs were sampled from connected lakes and rivers from the United States (Us), Canada (Ca), Norway (No), and from two sites in Germany (G1 and G2; Fig. 1 and S1 Table). As ice sheets covered these regions during the last glaciation, these populations represent recent colonization events (~12 000 years ago). Both lake and river populations are derived from marine ancestors that became landlocked during de-glaciation, and in which ecotype differentiation between watersheds has occurred repeatedly. Some phenotypic traits such as feeding morphology [13], brain development [7], and parasite resistance [14] seemingly differentiated in parallel with habitat (i.e. lake and river) suggestive of local adaptation. Furthermore, experimental studies have shown evidence for local adaptation to lake and river habitats mediated by parasites [15]. Hence, contrasting the differentiation between populations from distinct ecosystems permits us to study the onset of divergence, which might eventually lead to complete reproductive isolation (i.e. speciation). Here, we scan genomic divergence patterns and evaluate differences and commonalities across a wide geographic sampling of parapatric population pairs to uncover the relative importance and interaction of evolutionary factors like drift, selection, and recombination during adaptive divergence.

## Results and Discussion

### Lake and river population pairs

One consistent difference between lake and river habitats is that lake fish posses a higher parasites diversity than parapatric river fish. From previous work on three-spined sticklebacks, lakes and rivers in Northern Germany are known to harbour distinct parasite communities [14, 16]. Despite the relatively low sample size for individual locations in this study (n = 12–17), this ecological difference between lakes and rivers is here confirmed on a broader geographic scale (Fig. 1). From each of the ten sampled populations, six stickleback genomes were sequenced using a combination of paired-end and mate-pair libraries on the Illumina HiSeq platform to an average genomic coverage of 26-fold (S2 Table). Instead of sequencing many individuals with low coverage, a small number of genomes per population was chosen to be sequenced to high coverage. This approach takes advantage of the greater resolution of single nucleotide polymorphisms (SNPs) and copy number variations (CNVs; evaluated in greater detail in a companion paper [17]) plus increased genotype accuracy within each individual to decipher the divergence mechanisms acting towards an apparent repeated differentiation between lake and river fish. Besides evaluating allele frequencies, the high individual sequence coverage permits us to infer haplotypes and examine recombination patterns. After stringent quality filtering, we accessed 297,437,667 bp from the 20 autosomes (380,547,835 bp). SNP density varied from 3 to 10 SNPs per kilobase (kb) within each population (S3–S4 Tables). For each of the five parapatric comparisons, pairwise genome-wide averages of divergence (F_ST_) ranged from 0.10 to 0.28, disclosing a varying degree of differentiation in the ascending order of Us, G2, No, G1, and Ca (Table 1). The parapatric pairs emerge as repeated independent differentiation events (neighbor joining tree, Fig. 1A) except for the German populations, despite belonging to different draining systems (North Sea versus Baltic Sea). Due to low land levels and historically varying water levels, water bodies and connections across Northern Germany have most likely fluctuated over time. Thus the two lake and river population pairs in Germany (G1 and G2) might have been originally connected. Because of this, G1 and G2 share some postglacial history, common ancestral variation, and divergence while currently the two water systems are physically separated. Specifically, studies on the German system have proposed parasite communities as a promising candidate mediating divergent selection, pointing out their role in local adaptation [15, 18]. As a further global perspective of this hypothesis, we find a signal of isolation-by-adaptation (partial mantel test: r = 0.622, P = 0.0007) shown by a significant association of genome-wide F_ST_ and parasite community (jaccard distance of parasite sums across individuals, counts were 4^th^ square root transformed) while correcting for geographic distance (geodetic distance between GPS coordinates of each sampling location). As we detected isolation-by-adaptation at a spatial scale beyond which gene flow occurs, this signal might be most likely caused by a loose linkage between locally adapted loci and the genome-wide neutral regions [19]. This result suggests a role of parasites for the local adaptation of freshwater stickleback populations.

**Table 1:** Summary statistics characterizing divergent regions of exceptional differentiation across five lake-river comparisons.

**population pair**	**Us**	**G2**	**No**	**G1**	**Ca**
mean F_ST_ genome-wide *	0.09	0.11	0.22	0.22	0.28
**# divergent regions**	152	149	173	192	128
mean F_ST_*	0.48	0.46	0.59	0.59	0.73
**max size [kb]**	323	459	448	253	425
**mean size [kb]**	60 (+/- 48)	46 (+/- 50)	46 (+/- 50)	47 (+/- 37)	69 (+/- 64)
**sum length [kb]**	9164	6840	7995	8982	8790
**# genes**	510	430	391	488	479
**# background selection**	18	21	24	21	10
**# adaptation river**	20	51	69	51	23
**# adaptation lake**	43	24	35	43	20
**# reduced gene flow**	52	45	43	64	70

* SNPs were filtered for minor allele frequency > 0.25

### Repeated divergence involves distinct genomic regions

**Figure 2:**
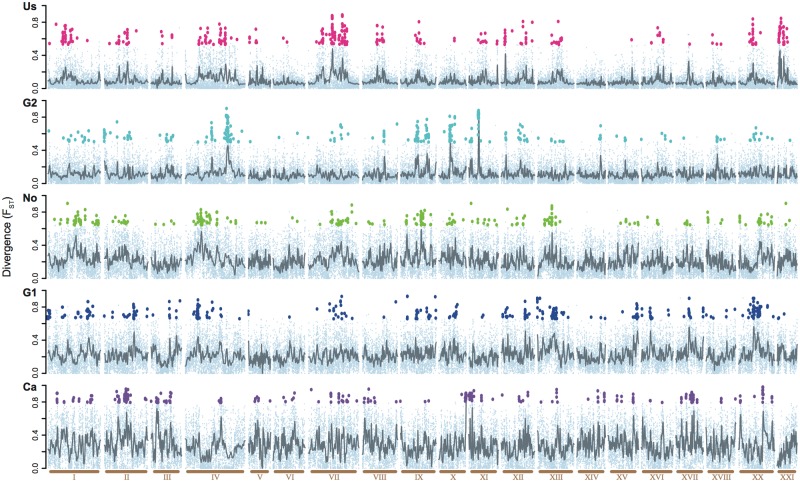
Comparison of the divergence across the genome in five parapatric lake-river pairs. Panels are ordered by increasing overall divergence (Us, G2, No, G1, Ca). Divergence (F_ST_) was averaged over 10 kb windows (small, light blue dots). Windows with exceptional differentiation (1,530 outlier windows) are highlighted with a population-specific color. Grey lines indicate smoothened averages across windows. All 20 autosomes excluding the sex chromosome (LG XIX) are shown along the x-axis in ascending order (light brown). We only find a weak correlation of F_ST_ values across the autosomes between the two geographically closest population pairs (G1 and G2, 34958 windows, Pearson correlation r = 0.0595, P << 0.001).

Spatial heterogeneity along the genome was analyzed between parapatric populations by applying a genome scan approach, which averaged genetic divergence (F_ST_) in 10 kb and 100 kb non-overlapping windows across the 20 autosomes (Fig. 2). The shape of the distribution of F_ST_ values across the genome qualitatively matches a skewed Poisson distribution, suggestive of divergence with gene flow (S1 Fig.) [9]. The pronounced right tail of the distributions aided the identification of outlier windows, which are significantly different from the genome-wide average. Outlier windows were detected for each population pair as the top 1% of the empirical distribution in addition to being significantly differentiated compared to a random permutation of markers across windows, applying a false discovery rate (FDR) of 0.01. Using the exact same approach comparing marine and freshwater populations, regions known to be under strong divergent selection such as *Eda* and *Atp1a1* were detected as outliers demonstrating the robustness and reliability of the applied methods (details see Methods). Across all five parapatric lake-river comparisons, we identified a total of 1,530 extreme 10 kb outlier windows, in which 47 are shared between at least two of the five population pairs, a proportion that is slightly more than expected by chance (10,000 permutations of random sampling gave on average 28 overlaps, one-tailed P = 0.0006), but none of the windows were shared across all five population pairs. Although we found a weak positive correlation of F_ST_ along the genome between the five lake and river ecotype pairs (Fig. 2 and S2 Fig.), there is a negative correlation of F_ST_ among the 1,530 outlier windows (Pearson correlation ranging from r = -0.2531 to -0.1064, all P<10^-4^). These results indicate that outlier windows in one population pair are often windows of low F_ST_ in the other population pairs. Hence, outlier windows are not the same across the different population pairs. Annotations for all genes overlapping common outlier windows can be found in S5 Table. None of these outlier windows overlapped with those detected in a previous lake and river comparison of different stickleback populations on the Haida Gwaii archipelago [20]. Thus outliers of exceptional differentiation appear to be locally specific for lake-river ecotypes on a wide geographic scale as well as on a narrow scale [20, 21]. This is in contrast to earlier comparisons between marine and freshwater sticklebacks where few loci are repeatedly found under divergent selection on a global scale [22, 23]. Our results are in line with the notion that the repeated differentiation between derived freshwater stickleback populations occurs as a response to different ecological pressures specific to their local environment [24]. This might reflect locally specific parasite communities, aside from the general trend of an increase in parasite diversity in lakes compared to rivers. However, genomic diversification seem to be an inevitable consequence following the dispersal across habitats, reinforcing the concept that local adaptation is a major contributor to the evolution of species.

### Patterns of divergence across a continuum of differentiation

In order to further understand processes shaping the heterogeneity of genomic divergence, we evaluated if divergence is widespread or localized along the genome. Divergence hitchhiking predicts a trend towards an increase in size of divergent regions with overall population differentiation [8, 19]. Conversely, if size was largely determined by the strength and duration of selection, the size of divergent regions would be independent of overall population differentiation. To test these predictions in our dataset, we exploited our comprehensive sequencing resolution to identify precise borders and dimensions of regions of exceptional differentiation. Amongst the 1,530 outlier windows, adjacent outlier windows were combined into 794 continuous outlier “regions” of exceptional differentiation estimated to the nearest 1 kb (S6 Table). The size of a region of exceptional differentiation was determined utilizing barrier strength (*b*, ref [25]) to contrast local divergence to the genome-wide average. We found a high degree of size heterogeneity among divergent regions within and across population pairs, with no evidence that the size of these regions increases with higher levels of genome-wide differentiation (Table 1, S3 Fig.). This also holds true when recombination rates are taken into account (see below). Therefore, the genomic pattern of divergence observed across a continuum of population differentiation suggests that selection at linked sites drives the observed pattern rather than the interplay of gene flow and divergent selection, consistent with the perspective of geographically specific local adaptation. However, additional factors such as soft sweeps resulting from adaptation based on standing genetic variation might also contribute to the observed patterns, further complicating interpretations.

**Figure 3:**
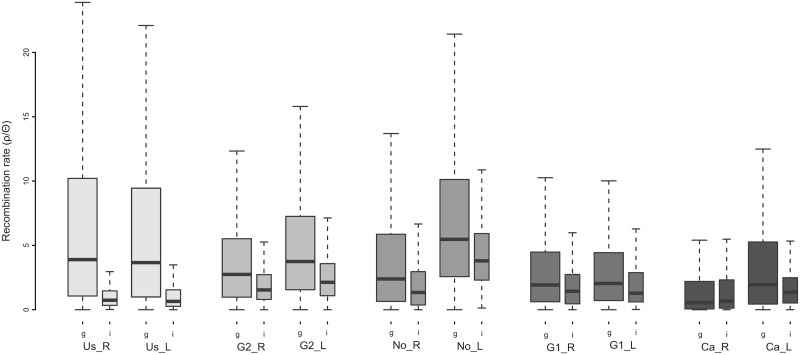
Boxplot illustrating the variation of realized population-scale recombination rates (ρ/Θ) across the genome in five lake-river population pairs. Population pairs are ordered by increasing genomic divergence (left to right), with rivers plotted first followed by lakes. Differences between genome-wide averages (first wider boxplots) and divergent regions (second narrower boxplots) are shown. In all populations except for Ca_R (Mann-Whitney, W = 2059100, P = 0.1232) and Ca_L (W = 2565656, P = 0.0078) population recombination rates are significantly reduced in divergent regions compared to the genome-wide mean (the least extreme being in G1_R, W = 3892202, P = 0.0004; Bonferroni correction for family error rate lowered significance level: alpha/m = 0.005). Notice that with increasing genomic divergence (left to right), genome-wide recombination rates decrease (Pearson r = −0.63, P = 0.05, df = 8); there is no such correlation for the average recombination rate in divergent regions (Pearson r = 0.51, P = 0.13, df = 8).

To further explore if the observed divergence patterns are indeed facilitated by selection and not induced by drift alone, we investigated fine-scale linkage patterns and their effects on genomic heterogeneity across a populations. For each population, we estimated the realized population-scaled recombination rates (ρ/Θ) along the genome. Both a local reduction of gene flow mediated by divergent selection and selection with the hitchhiking of linked neutral sites are predicted to produce a negative correlation between F_ST_ and recombination rate [12, 26], however this association would be unlikely mediated by drift alone. In addition, divergence hitchhiking predicts that over time, linkage will extend along the genome and eventually encompass large tracts of the genome [27]. In our study, realized recombination rates in regions of exceptional differentiation were often significantly reduced compared to genome-wide estimates (Fig. 3). We found that genome-wide recombination rates tended to decrease with increasing overall differentiation (Fig. 3). However, realized recombination rates in divergent regions are not significantly correlated with genome-wide differentiation, adding to the growing lack of empirical evidence for divergence hitchhiking [28]. These results suggest that either actual recombination rates coincide regions of the genome, which become divergent, or selection drives local reductions in realized recombination rates. The coalescent-based population recombination rates (4N_e_r) estimated in this study are simultaneously affected by the variation in genomic structure within and across populations, which may influence actual recombination rates, as well as by selection. Hence, selection might have locally reduced realized recombination rates in certain genomic regions or actual recombination has been reduced due to the intrinsic genomic structural variations thereby promoting genomic divergence. Previous studies evaluating large-scale map-based recombination patterns in sticklebacks have also found a correlation between recombination and divergence, suggesting that genome structure, via its influence on recombination, is important in understanding patterns of genomic differentiation [29, 30]. Here, the low correlation in divergence (F_ST_) between different population pairs (Fig. 2) suggests that local factors specific to each population pair drive genomic differentiation, and that population specific selection reduces realized recombination, particularly if genomic structure is conserved across populations. However, it is possible that genome structure is not so strongly conserved across these geographically distant pairs. Structural variations such as inversions and CNVs have been shown to be abundant within stickleback populations [31]. A companion paper [17] highlights the prevalence of CNVs among and between the populations studied here, in which CNVs tend to also be population specific. These findings indicate that genome structure might be more variable than expected, and therefore might hold potential for promoting genomic differentiation in a population specific manner. We cannot here distinguish between selection-induced influences on realized recombination rates, and actual variation in recombination rates due to differences in genome structure and resultant effects on patterns of genomic differentiation. Further understanding of genome structure’s influence on recombination rates, and its variability within and across populations, will be crucial for disentangling the combined influences of selection and recombination on patterns of genomic variation.

### Molecular signatures of selection in divergent genomic regions

**Figure 4:**
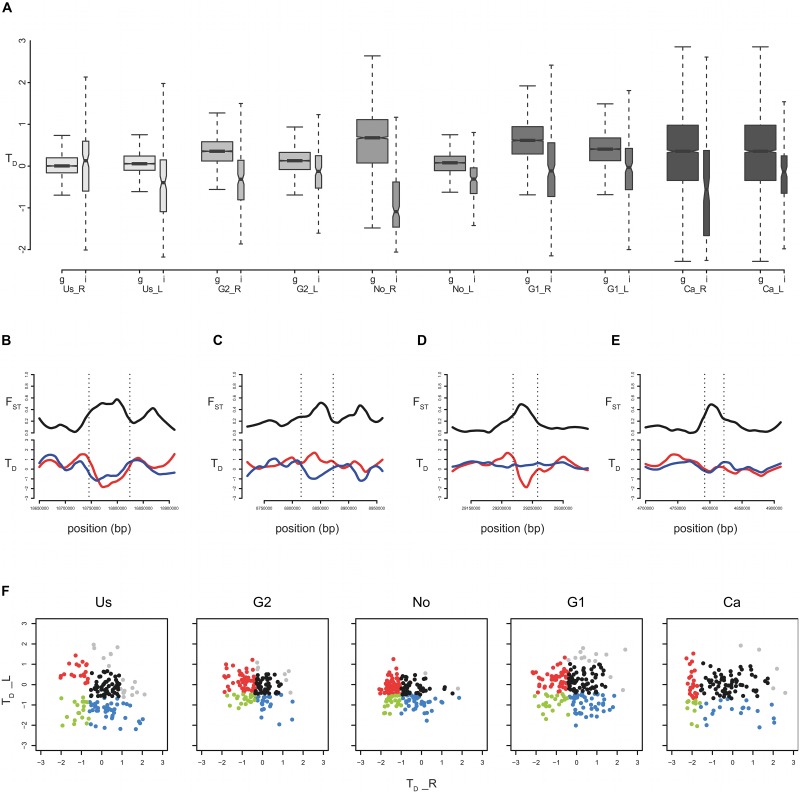
Characterizing divergent regions of exceptional differentiation by molecular signatures of evolution. **A)** Boxplot demonstrating the negative shift of Tajima’s D (T_D_) in divergent regions (right box) compared to the genome-wide means (100 kb windows; left box). Population pairs are ordered by increasing genomic divergence (left to right), with rivers plotted first followed by lakes. In all populations except for Us_R (Mann-Whitney, W = 290246, P = 0.7942) T_D_ in divergent regions is significantly different from the genome-wide mean (the least extreme being in G2_L, W = 198559, P < 10^-8^; Bonferroni correction for family error rate lowered significance level: alpha/m = 0.005). **B-E)** Representative molecular signatures of evolution used to categorize regions. Smoothed averages across 10 kb windows are given for F_ST_ (black) and T_D_ in the river population (red) and the lake population (blue). **B)** Low T_D_ values in both the lake and the river (below the 5% quantile of the genome-wide distribution) were interpreted as evidence for background selection (selection against new mutations in both populations causing a divergence signal by reducing diversity in both populations). **C)** Low T_D_ values in only the parapatric lake population was interpreted as indications for positive selection (local adaptation) as the potential cause for a local reduction of effective population size. **D)** The same as (C) except for low T_D_ values in only the parapatric river populations. **E)** Regions that fall into the middle of the T_D_ distribution (excluding the 5% most extreme values of both tails) showing neutral patterns were interpreted as being shaped by a reduction of gene flow. **F)** For each divergent region, Tajima’s D (T_D_) values are plotted for the lake (y-axis) and river (x-axis) of each population pair. Categories as given above and in Table 1 are represented by colors: red dots represent “adaptation river”, blue “adaptation lake”, green “background selection”, and black “reduced gene flow”. Please refer to Table 1 for the exact number of divergent regions per category and population pair.

Relative divergence (F_ST_) in regions with low levels of recombination might be misleadingly interpreted as conclusive evidence for a local reduction of gene flow. For this reason, measurements of absolute divergence such as D_xy_ have been suggested as a complement to more reliably identified regions of locally reduced gene flow [10, 12, 32]. However, absolute divergence measurements are unreliable statistics for nascent populations and in non-equilibrium situations during population differentiation. Hence, we aim to disentangle different mechanisms shaping regions of exceptional differentiation by assessing selective sweep signatures in one or both populations of each parapatric pair. Utilizing the base pair resolution of our whole genome sequence data, we evaluated allele frequency spectra to differentiate between molecular signatures of selection among individual regions of exceptional differentiation. In divergent regions differentiated due to a local restriction of gene flow mediated by selection, the spectrum is not expected to be affected locally and should reveal a signature of neutral evolution [12]. The opposite is true for regions resulting from selection with hitchhiking at linked sites, which causes a characteristic skew of the spectrum. An excess of rare alleles is expected in a population experiencing a selective sweep [33], or in both populations in the case of background selection [34]. Distortions in the allele frequency spectrum were calculated for each population as Tajima’s D (T_D_) across the genome in 100 kb windows and in each region of exceptional differentiation. Genome-wide averages of T_D_ varied from 0.0385 to 0.5936 suggesting predominantly neutral evolution across the genome with no indication for an excess of low frequency polymorphism in any of the populations. T_D_ values within regions of exceptional differentiation were shifted towards negative values except for the Alaskan river (Us_R, Fig. 4A). These negative shifts of T_D_ are consistent with selection as a major mechanism responsible for localized divergent regions along the genome.

In order to quantify the relative contribution of different mechanisms shaping the genomics of speciation, we partitioned individual regions of exceptional differentiation into four mutually exclusive categories with different molecular signatures of evolution based on contrasting local T_D_ values to the genome-wide average (Table 1 and Fig. 4B–F). The minority of divergent regions is consistent with background selection (12%, T_D_ reduced in both populations, Fig. 4B), whereas adaptation seems to shape most of the divergent regions (48%), consistent with the influential role of selection. Divergent regions with signals of positive selection (T_D_ reduced in one of the two populations) should harbor those genes responsible for local adaptation. Genes in divergent regions with a signature of positive selection in lakes (Fig. 4C) were overrepresented with functions involved in structural molecule activity (18 out of 260 annotated genes, P = 0.0018), while genes in divergent regions with signals of positive selection in rivers (Fig. 4D) were overrepresented with functions involved in G-protein coupled receptor activity (15 out of 105, P = 0.0038), antiporter activity (6 out of 36, P = 0.0280), and drug transmembrane transporter activity (4 out of 8, P = 0.0367), suggesting functions in environmental response. Divergent regions with neutral T_D_ patterns (T_D_ in both populations similar to genome-wide average, Fig. 4E) potentially harbor genes restricting gene flow. Despite the prominent occurrence of neutral T_D_ patterns among divergent regions (35%), we found no functional overrepresentation of genes within those regions (S6 Table). This indicates that a variety of different genes and functions might be involved in reproductive isolation, but the current state of gene annotations does not allow drawing compelling conclusions. Overall, the variety of molecular signatures of selection found in divergent regions suggests that different evolutionary processes shape regions of exceptional differentiation. We acknowledge that our approach of strictly categorizing regions based on thresholds simplifies a complex situation, in which various factors most likely interact to shape genomic divergence. However, our analysis suggests that different processes have different impacts across the genome, with selection being a probably major contributor. Therefore, the effects of a local reduction of gene flow and local adaptation are mutually compatible and probably act in concert to shape the genomic landscape of divergence between differentiating parapatric stickleback populations.

### Conclusion

We presented multiple lines of evidence for the role of adaptation shaping the genomic divergence patterns between lake-river populations of three-spined sticklebacks. Aside from adaptive processes, stochastic variation in coalescent times and variable mutation rates could further contribute to the observed heterogeneity of genomic divergence [35]. In particular, demographic history such as colonization events (population range expansions) might lead to a substantial variation in allele frequencies across the genome, possibly mimicking the patterns of adaptive hitchhiking [36]. Here, we have chosen the genome-wide average as proxy of the underlying demographic history and the effect of random drift on these populations, as detailed demographic information is scarce. Today, fish migration from the sampled rivers flowing into lake habitats is possible while migration in the opposite direction is likely constrained by physical barriers (S1 Table). However, as freshwater systems have been subject to recurrent water-level changes during de-glaciation, the spatial context at different stages of population divergence might have fluctuated over the years affecting demographic history of the populations. Due to pronounced local differences and variable genomic patterns across the sampled continuum of genetic population differentiation we conclude that the main mode of contemporary divergence between parapatric three-spined sticklebacks is associated with population-specific local adaptation. This is potentially partially mediated by differences in the parasite, as we also found a corresponding signature of isolation by adaptation. Furthermore, our fine-scale examinations of molecular evolution suggest that some heterogeneity of genomic divergence is also the result of locus-specific differences in gene flow mediated by divergent selection. Our study has taken an important step towards deciphering the underlying mechanisms responsible for the genomic patterns during speciation, one of the fundamental enigmas in evolutionary biology.

## Materials and Methods

### Sampling and data processing

Three-spined stickleback fish were caught from five pairs of lakes and rivers in North America and Northern Europe (S1 Table and Fig. 1). Between 12 and 17 fish were screened for macroparasites following established procedures [14]. Both Shannon diversity indices for each population and jaccard distance between populations were estimated on the basis of 4^th^ square root transformed parasite counts. Muscle tissue from six sampled individuals from each location was used for DNA extraction (using a Qiagen DNA Midi Kit following the manufacturer’s protocol for high molecular weight DNA) and Illumina sequencing following previous methods [31]. To capture natural variation present in the wild, we randomly picked individual fish for sequencing (albeit targeting equal sex ratio per population and similar fish sizes across populations), thus without pre-selection of any particular morphological or parasitological characteristics. For each individual, two paired-end libraries (100bp reads, average insert size of 140bp and 300bp) and a mate-pair library (50bp reads, average insert gap of 3kb) were produced, achieving an average depth of coverage of 26x (S2 Table). Data is deposited in the European Nucleotide Archive (PRJEB5198). Raw sequence data was processed and filtered following previous procedures [31] and mapped against the three-spined stickleback reference genome [22] from Ensembl version 68 [37] with BWA (Burrows-Wheeler Aligner) software [38].

Mapped reads were further filtered and processed utilizing the Picard toolkit following previous procedures [31]. SNPs and indels were called with GATKv1.6 [39, 40] using concordant SNP calls from SAMtools v0.1.18 [41] for variant recalibration. Phasing and imputation was performed with BEAGLE v3.1 [42]. VCFtools [43] was utilized for processing genotypes. Positions overlapping with ‘N’s and repeat-masked regions from the Ensembl annotations (version 68) were removed from the final genotype file. Furthermore, variants within 10bp of an indel or indicating copy number variation were also excluded. Copy number variable (CNV) regions were identified by deviations in expected read depth with the software CNVnator [44]. More details on the CNV analysis are given in a companion paper submitted by Chain *et al*. The following analyses were performed on the 20 autosomes, spanning 380,547,835 sites in the reference genome. After removing masked sites and CNV region and imputing genotypes across 60 individuals, 297,437,667 sites were reliably genotyped and used for estimating population genetics parameters.

### Validation of genotypes

We used Illumina’s Golden Gate platform for cross checking genotypes from SNP sites distributed across the genome. Each chromosome held on average 9 (range 2–21) markers and the total of 183 loci were mostly interspersed by at least 50 kb. We found a high overall concordance (98% in 12,041 comparable sites) between genotype calls from the Golden Gate assay and our sequencing pipeline.

### Population genetics

The population genetics estimators of nucleotide diversity (π and Θ) and Tajima’s D (T_D_) were calculated with VCFtools v0.1.11 [43] for each of the 10 populations (S3 Table), in addition to the relative divergence (Weir and Cockerham F_ST_) and absolute divergence (D_xy_ [45]) estimated for each of the 5 parapatric lake-river pairs (S4 Table). Numbers of polymorphic sites per population and per population pair are reported in S3–S4 Tables. To illustrate the relationship amongst all sampled populations, we utilized a set of 1,074,467 intergenic autosomal polymorphic loci to estimate pairwise divergence (Weir and Cockerham F_ST_) and built a neighbor joining tree. To gain support for the tree topology we randomly down sampled this dataset 100 times to 100,000 loci. For the genome scan, F_ST_ was calculated on the full dataset that was further filtered for minor allele frequencies below 25% across each pairwise comparison excluding uninformative polymorphism [46]. This way we evaluated the divergence between parapatric population pairs on the basis of 691,957 to 1,227,732 sites across the 20 autosomes. Population genetics estimators were averaged across the genome (20 autosomes) in non-overlapping windows to ensure statistical independence of windows. We used window sizes of 10 kb and 100 kb and confirmed that results are qualitatively the same. Diversity estimates have been corrected for the number of sites for which genotypes are available.

### Divergent regions of exceptional differentiation

Outlier windows were determined by combining an empirical approach with a permutation approach. First, windows above the top 1% of the empirical distribution were identified as putative outlier windows. Second, we applied a permutation approach in which loci across the genome were permuted 1,000,000 times and window estimates of F_ST_ were tested against permutations holding the same amount of variable sites. Putative outlier windows from this permutation approach were identified after adjusting for a FDR of 0.01. Our final set of outlier windows consisted of those windows that were significant outliers in both approaches. All statistical procedures and visualizations were implemented in R [47]. Outlier window positions were compared across the five replicated lake-river comparisons. To evaluate how many overlapping outlier windows were expected by chance, windows were permutated 10,000 times utilizing bedtools [48].

To approximate the size of regions of exceptional differentiation more in detail, adjacent outlier windows were combined to form larger contiguous divergent regions of extreme differentiation. In each resulting candidate region, the locus of maximal divergence was determined as a starting point, in which outward steps of 1 kb windows were binned to estimate barrier strength (*b*, ref [25]). Margins of divergent regions showing extreme differentiation were determined when *b* dropped below 1 (genome-wide average) in two consecutive 1 kb bins. This resulted in divergent regions of exceptional differentiation with distinct sizes estimated to the nearest 1 kb. Divergent regions with sequence coverage (sequence information accessible, see details above) spanning less than 50% of their length were excluded from subsequent analyses. Average sizes of about 50 kb are independent of the initial window size used but specific values reported here are based on the 10 kb window size approach (Table 1).

### Effect of sample size on power

We acknowledge that estimates of F_ST_ based on allele frequencies can vary depending on samples size [49]. To reduce variation of estimates between populations we kept the samples size constant at 12 alleles per populations. Additionally, our analysis did not rely on per site estimates but instead on averages of F_ST_ over larger regions (see above). We evaluated the effect of sample size on our power to describe genomic patterns, detect outlier windows, and define divergent regions in the three following ways. (i) We tested the accuracy of our F_ST_ estimates at individual loci by comparing them to estimates based on a larger sample size. The 183 loci used for validating the genotypes (see above) were also used to genotype a larger population sample (n = 26–59 per population) to validate allele frequencies and resulting F_ST_ estimates. For all population pairs, the F_ST_ estimates based on the sequencing approach with 6 individuals per population (12 alleles) had a significant positive correlation with the F_ST_ estimates from the Golden Gate assay using at least 26 individuals (Pearson correlation, r = 0.85, P< 10^-16^, df = 241, S4 Fig.). (ii) We tested the consistency of window F_ST_ estimates across the whole range of potential F_ST_ values by jack-knifing samples (S5 Fig.). On average, jack-knifed values (comparing 10 alleles per population) had 95% confidence intervals of 0.039 up to a maximum of 0.175. Windows with high F_ST_ values (>0.75) had even narrower confidence intervals (average of 0.027 and maximum of 0.088). These results support the notion that pronounced differences (“near-” and “post-fixation”) can be more reliably detected using our sample sizes than more settled differences (“pre-fixation” regime). (iii) We tested our ability to detect known candidate genes, which highly differentiate between marine and freshwater populations. For this we utilized previous sequencing data available for a marine population from Denmark [31]. Our genome scan based on F_ST_ estimates averaged across 10 kb windows reliably detected windows overlapping *ATP1a1* [50], a well known candidate gene for physiological adaptation to osmotic differences on linkage group I, in all 6 pairwise European marine-freshwater comparisons (S6 Fig.). *Eda*, the major gene (linkage group IV) underlying the reduction of lateral plate number frequently observed in freshwater populations [51], was detected in 5 out of the 6 pairwise European marine-freshwater comparisons (S7 Fig.). As expected G1_L, a lake population showing phenotypic variation at this trait did not show significant differentiation in the *Eda* region, in which two of the six sequenced individuals were fully plated and carried the same haplotype as the fully plated marine fish. This is in line with a simulation demonstrating that sampling 12 haplotypes yields between 67–95% power compared to a gold standard, while notably, sampling fewer individuals has the greatest impact in the “pre-fixation” regime (a beneficial allele is starting to rise in one population) compared to “near-fixation” and “post-fixation” regimes (a beneficial allele is nearly or completely fixed in one population) [52].

### Molecular signature of selection in divergent regions

To assess the molecular signature of selection in regions of exceptional differentiation, shifts in the allele frequency spectrum were evaluated utilizing T_D_. T_D_ in these regions was compared to the genome-wide average of each respective population. A 5% threshold was applied to classify divergent regions into four mutually exclusive categories: background selection if T_D_ dropped below the threshold in both parapatric populations, adaptation in lake or river if T_D_ dropped below the threshold only in the respective population, and reduced gene flow if T_D_ appeared neutral (not below the threshold). Comparing the utilization of population specific thresholds for each pairwise comparison with the utilization of the same overall averaged threshold for all populations resulted in minor differences in absolute numbers of regions in different categories. Furthermore, these differences did not affect qualitative changes with respect to the functional annotation of different categories, nor the proportion of different categories across the five parapatric population pairs.

### Recombination rates

Direct measures of fine-scale population recombination rates (ρ = 4N_e_r) were obtained with LDhat [53, 54] from patterns of genetic variation for each population separately. We filtered highly localized breakdowns of linkage disequilibrium (values of ρ above 100 between adjacent SNPs), as those are most likely artifacts, possibly due to local misassembly of the reference genome or clusters of erroneous SNPs [55]. Resulting recombination rate estimates were averaged over each 10 kb window and over each divergent region with exceptional differentiation, and corrected by the population specific mutation rate (Θ = 4N_e_µ) estimated as an average across all autosomes.

### Annotation

Regions overlapping with gene annotations from version 68 of Ensembl were identified using intersectBed of bedtools [48]. Annotations for shared outlier windows and divergent regions are reported in S5–S6 Tables. To determine enrichment of functional classes of genes among regions, topGO [56] was used with a universe of autosomal genes, and significance was determined at the 0.05 level using FDR adjusted p values to correct for multiple testing.

### Ethical statement

This study was performed according to the requirements of the German Protection of Animals Act (Tierschutzgesetz) and was approved by the ‘Ministry of Energy, Agriculture, the Environment and Rural Areas’ of the state of Schleswig-Holstein, Germany (reference number: V 312–72241.123–34). Wild sticklebacks were caught using minnow traps or hand nets. Before dissection, the fish were anesthetized with MS222 and sacrificed by an incision into the brain followed by immediate decapitation, and every effort was made to minimize suffering. No further animal ethics committee approval was needed. The species used in this study are not endangered or protected in any of the populations studied.

## Supporting Information

S1 Fig Frequency distribution of F_ST_ (100 kb window averages). Population pairs with a low degree of genome-wide divergence show a characteristic L-shaped distribution, which widens with increasing divergence. Locations are given above their respective figure. Loci with a minor allele frequency below 0.25 have been filtered out.
(TIFF)

S2 Fig Scatterplot comparing F_ST_ values between the two geographically close German population pairs demonstrating the low degree of correlation in divergence across the autosomes (34,958 windows, Pearson correlation r = 0.0595, P << 0.001). (TIFF)

S3 Fig Boxplot demonstrating the variation in sizes of divergent regions of exceptional differentiation within and across lake-river pairs. Genome-wide divergence between pairs increases from left to right. There is no correlation between average genome-wide divergence and average region size (Pearson r = 0.26, P = 0.67, df = 3).
(TIFF)

S4 Fig Correlation of site-specific pairwise F_ST_ estimates based on 6 individuals per population and based on more than 26 individuals of the same population. (TIFF)

S5 Fig Variation of window F_ST_ estimates evaluated by jack-knifing the sample size per population down to 5 individuals. Mean window estimates across all jack-knifed samples are plotted in increasing order (black line). Blue lines indicate the 95% confidence interval around the mean.
(TIFF)

S6 Fig Window scan (10 kb) of divergence (FST) across the region on linkage group I, where *ATP1a1* is located. Windows overlapping *ATP1a1* are highlighted in black. Note that divergence is elevated in all comparisons between a marine population from Denmark and the six European freshwater populations.
(TIFF)

S7 Fig Window scan (10 kb) of divergence (F_ST_) across the region on linkage group IV, where *Eda* is located. Windows overlapping *Eda* are highlighted in black. Note that divergence is elevated in five comparisons between a marine population from Denmark and European freshwater populations. Divergence is not increased in the comparison with G1_L, a population showing substantial variation in lateral plate number.
(TIFF)

S1 Table Summary of sample site information. (PDF)

S2 Table Summary of sequencing statistics for each individual. (PDF)

S3 Table Summary statistics for each population. (PDF)

S4 Table Summary statistics for each population pair. (PDF)

S5 Table Annotations for 47 shared outlier windows. (PDF)

S6 Table Annotations for 794 divergent regions. (PDF)
