# Correction: Genomics of Divergence along a Continuum of Parapatric Population Differentiation

**DOI:** 10.1371/journal.pgen.1005414

**Published:** 2015-07-30

**Authors:** Philine G. D. Feulner, Frédéric J. J. Chain, Mahesh Panchal, Yun Huang, Christophe Eizaguirre, Martin Kalbe, Tobias L. Lenz, Irene E. Samonte, Monika Stoll, Erich Bornberg-Bauer, Thorsten B. H. Reusch, Manfred Milinski

References 1 and 2 are incorrect. The correct references are listed here:

1. Martin SH, Dasmahapatra KK, Nadeau NJ, Salazar C, Walters JR, et al. (2013) Genome-wide evidence for speciation with gene flow in *Heliconius* butterflies. Genome Res 23: 1817–1828.
2. Ellegren H, Smeds L, Burri R, Olason PI, Backstrom N, et al. (2012) The genomic landscape of species divergence in *Ficedula* flycatchers. Nature 491: 756–760.
