# Supplementary figures and images for: Genomics of Divergence along a Continuum of Parapatric Population Differentiation
Source: PLoS Genet. 2015 Feb 13;11(2):e1004966. doi: 10.1371/journal.pgen.1004966 (PMC4334544; doi:10.1371/journal.pgen.1004966)

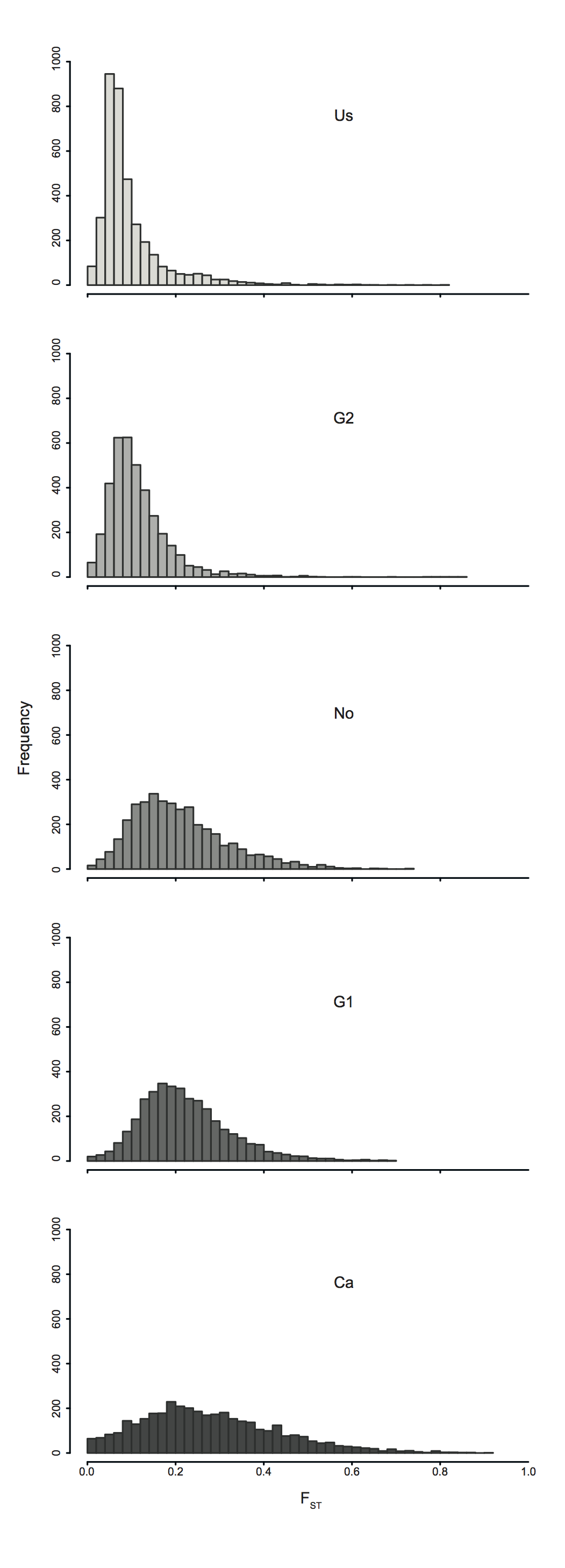

Supplement: S1 Fig — Population pairs with a low degree of genome-wide divergence show a characteristic L-shaped distribution, which widens with increasing divergence. Locations are given above their respective figure. Loci with a minor allele frequency below 0.25 have been filtered out. (TIFF) [file pgen.1004966.s001.tiff]

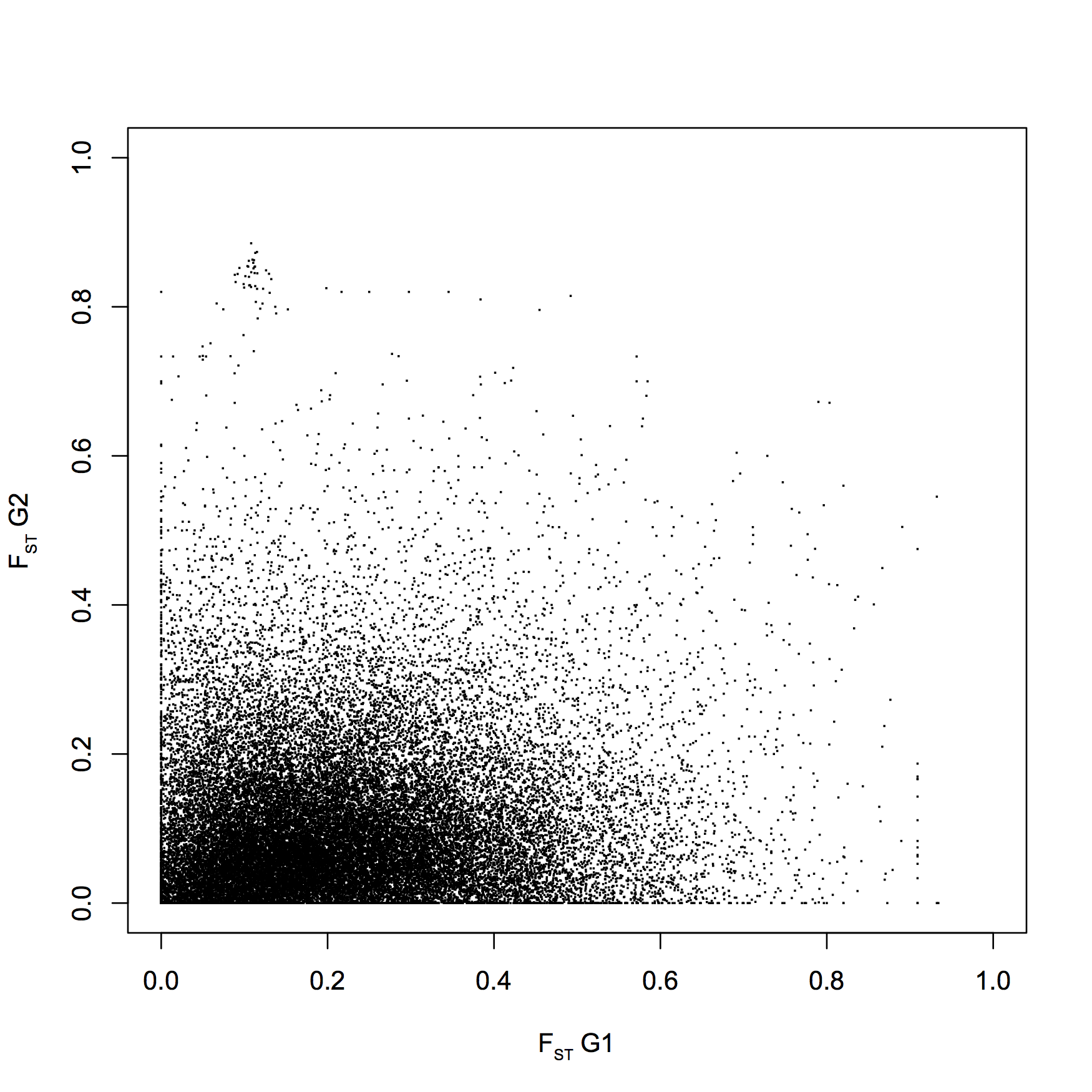

Supplement: S2 Fig — (TIFF) [file pgen.1004966.s002.tiff]

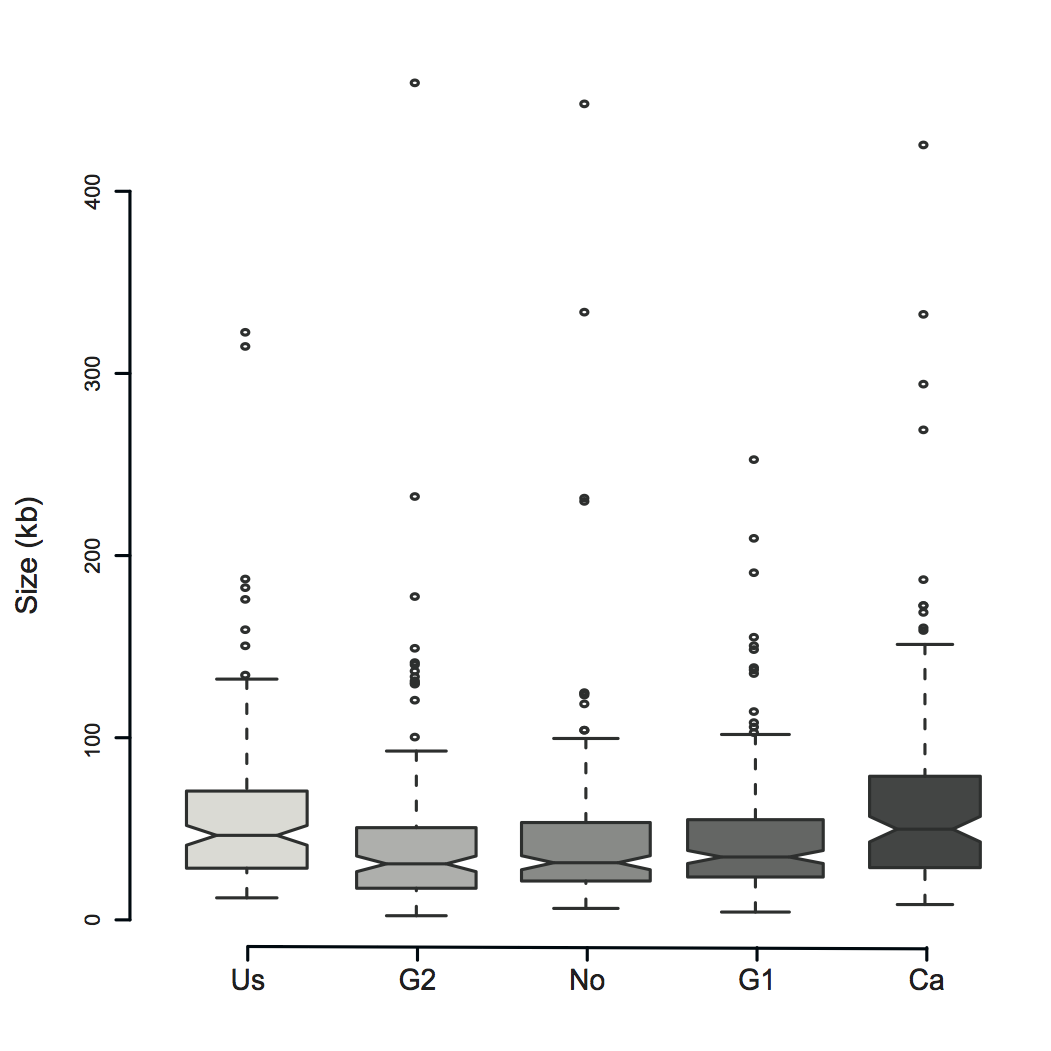

Supplement: S3 Fig — Genome-wide divergence between pairs increases from left to right. There is no correlation between average genome-wide divergence and average region size (Pearson r = 0.26, P = 0.67, df = 3). (TIFF) [file pgen.1004966.s003.tiff]

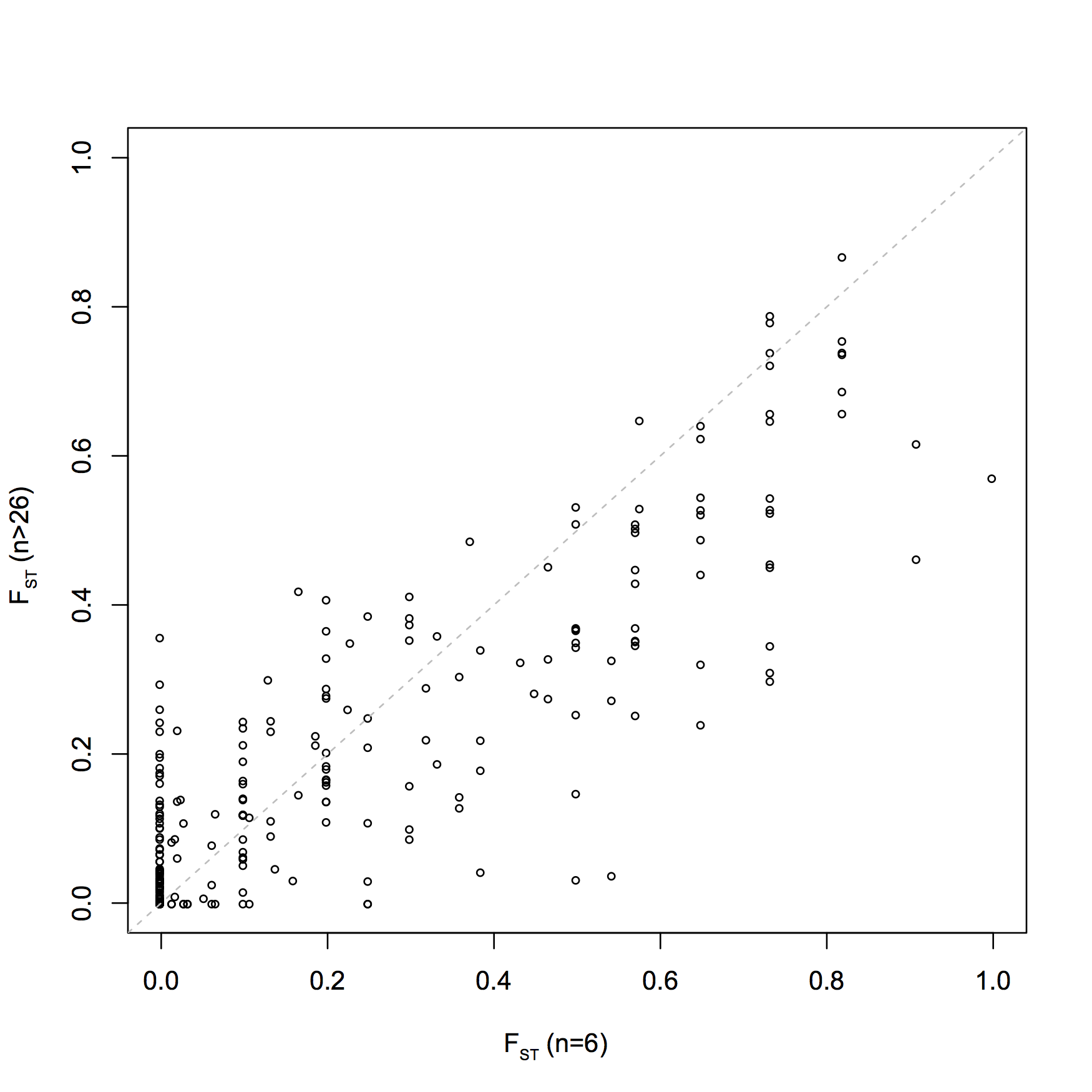

Supplement: S4 Fig — (TIFF) [file pgen.1004966.s004.tiff]

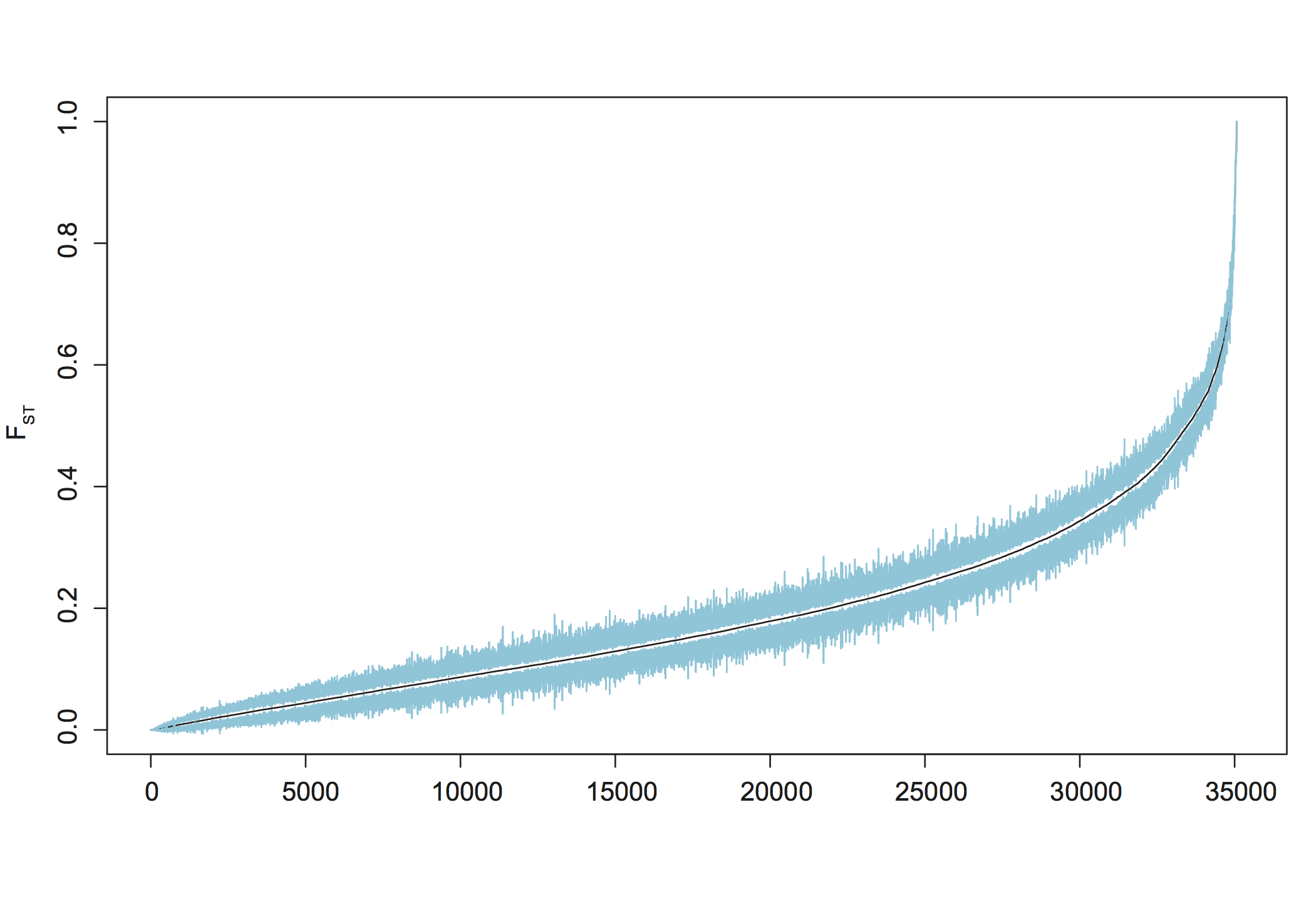

Supplement: S5 Fig — Mean window estimates across all jack-knifed samples are plotted in increasing order (black line). Blue lines indicate the 95% confidence interval around the mean. (TIFF) [file pgen.1004966.s005.tiff]

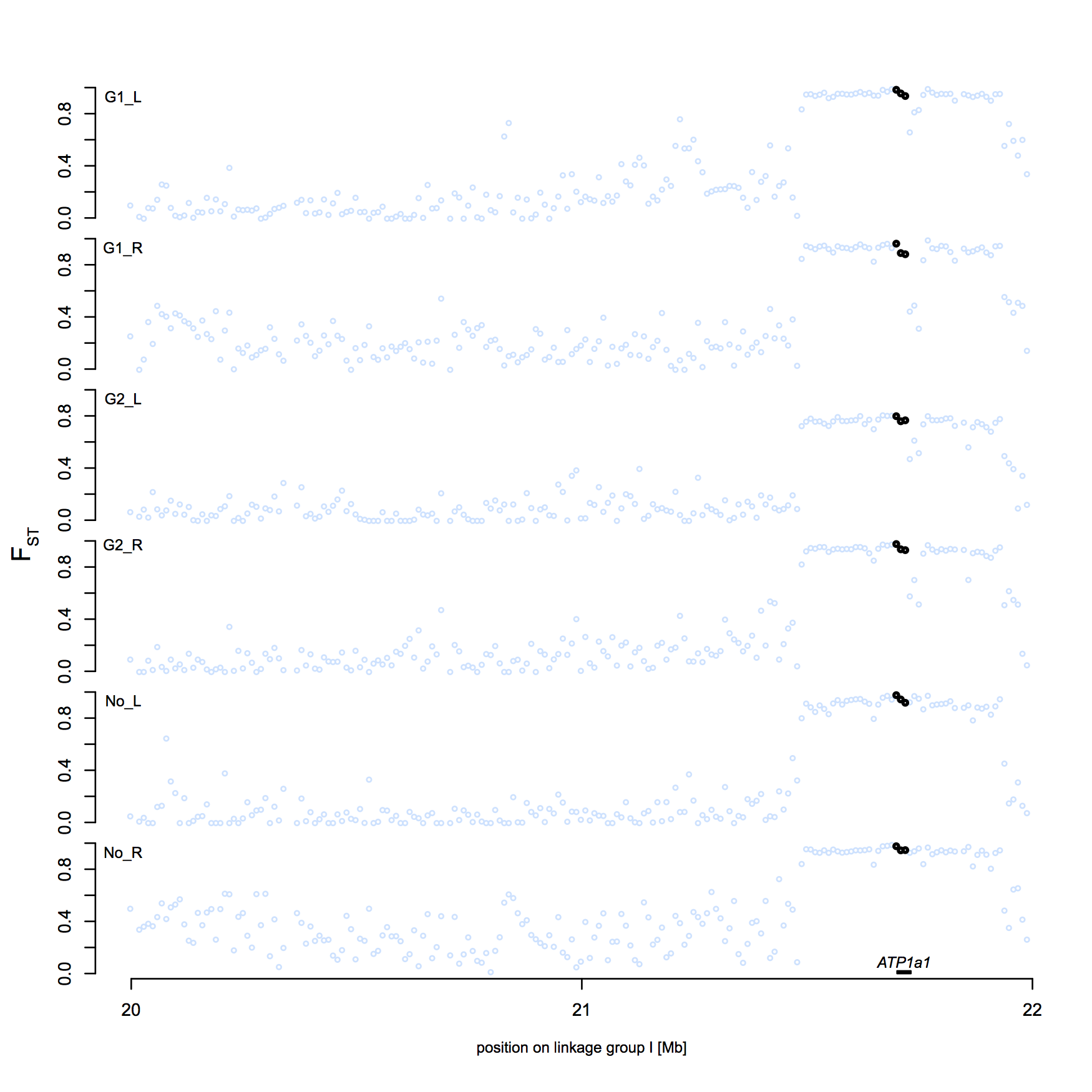

Supplement: S6 Fig — Windows overlapping ATP1a1 are highlighted in black. Note that divergence is elevated in all comparisons between a marine population from Denmark and the six European freshwater populations. (TIFF) [file pgen.1004966.s006.tiff]

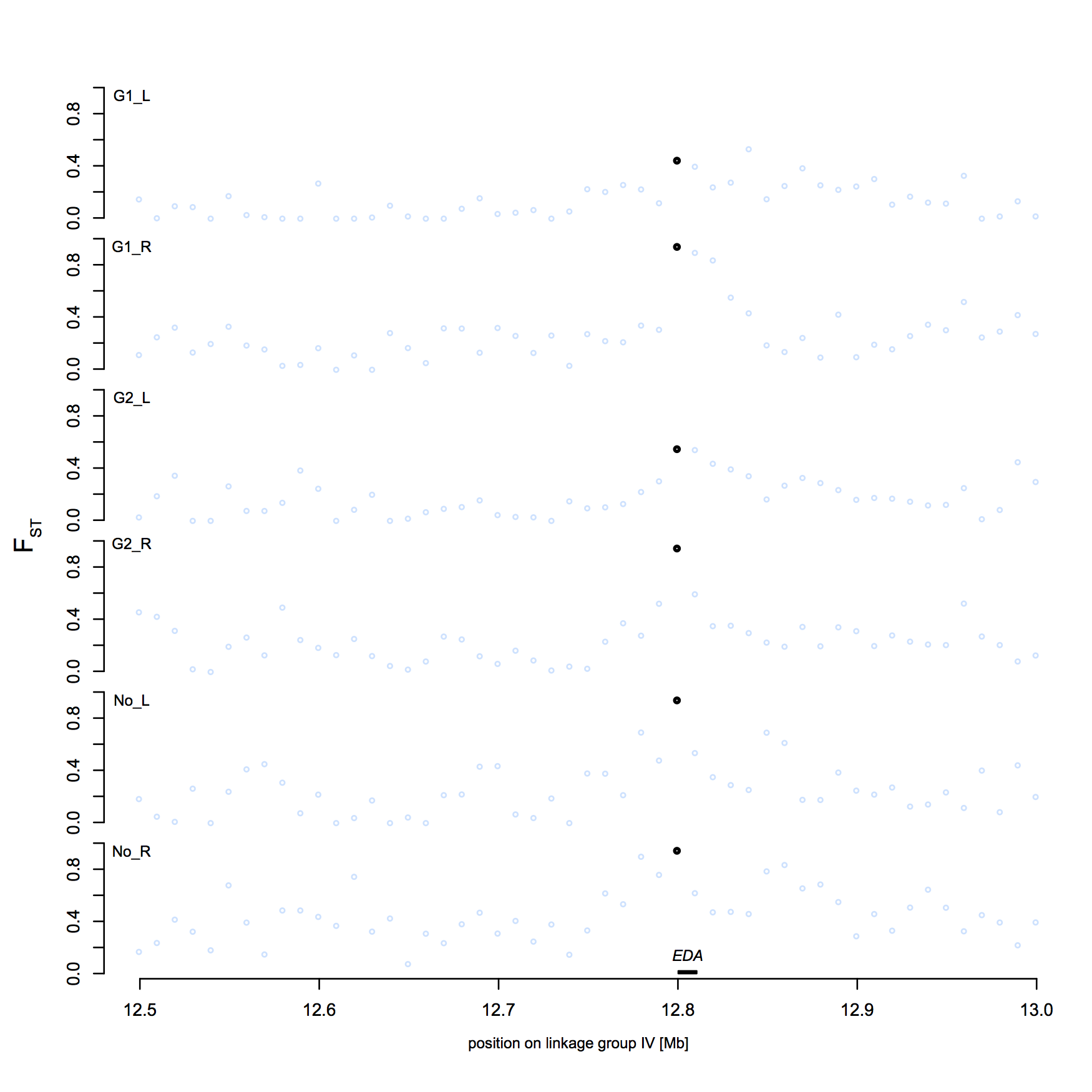

Supplement: S7 Fig — Windows overlapping Eda are highlighted in black. Note that divergence is elevated in five comparisons between a marine population from Denmark and European freshwater populations. Divergence is not increased in the comparison with G1_L, a population showing substantial variation in lateral plate number. (TIFF) [file pgen.1004966.s007.tiff]
