# Supplementary material for: Genomics of Divergence along a Continuum of Parapatric Population Differentiation
Source: PLoS Genet. 2015 Feb 13;11(2):e1004966. doi: 10.1371/journal.pgen.1004966 (PMC4334544; doi:10.1371/journal.pgen.1004966)
Supplement: S1 Table — (PDF) [file pgen.1004966.s008.pdf]

Table S1. Summary of sample site information.

Population ID	Population	Ecotype	Location	Coordinate	Width [km] Area [km^2]*	Swimming distance [km]	Air line distance [km]	Potential physical barriers for migration from lake to river
G1_R	Malenter Au	River	Schleswig-Holstein, Germany	54°12'15.08"N, 10°33'41.90"E	0.003	17.596	10.083	fish pass at a mile (~3 m height), dam at a mile
G1_L	Großer Plöner See	Lake		54° 9'21.61"N , 10°25'48.52"E	29.169			
G2_R	Eider	River	Schleswig-Holstein, Germany	54° 09' 58.07'' N, 10° 04' 31.05'' E	0.004	33.374	15.695	dam at mile (~1 m heigth)
G2_L	Westensee	Lake		54°16'39.70"N, 9°55'41.04"E	6.943			
No_R	Orraelva	River	Fusa, Norway	60° 15' 19.33"N, 5° 55' 35.68"E	0.016	1.582	1.400	waterfall of ~6 m in height
No_L	Skogseidvatnet	Lake		60° 14' 41.57"N, 5° 54' 55.39"E	5.325			
Ca_R	Misty Stream Inlet	River	Vancouver Island, Canada	50° 36' 10.40"N, 127° 15' 8.30"W	0.010	0.849	0.743	step (~30 cm in height)
Ca_L	Misty Lake	Lake		50° 36' 16.40"N, 127° 15' 42.30"W	0.358			
Us_R	Little Meadow Creek	River	Alaska, USA	61° 34' 8.76"N, 149° 45' 36.00"W	0.006	5.040	1.039	partially upstream
Us_L	Long Lake	Lake		61° 34' 33.96"N, 149° 46' 25.50"W	0.163			

* width of river at sampling site or surface area of lake
