# Supplementary material for: Genomics of Divergence along a Continuum of Parapatric Population Differentiation
Source: PLoS Genet. 2015 Feb 13;11(2):e1004966. doi: 10.1371/journal.pgen.1004966 (PMC4334544; doi:10.1371/journal.pgen.1004966)
Supplement: S2 Table — (PDF) [file pgen.1004966.s009.pdf]

Table S2. Summary of sequencing statistics for each inidvdual.

Population ID	Individual ID	Sex	Raw data (Gbp)	Final depth of coverage
G1_R	BS1	Female	16.1	25.5
	BS3	Male	20.4	31.7
	BS5	Male	18.9	26.9
	BS7	Female	22.7	33.5
	BS9	Male	16.4	25.2
	BS11	Female	15.5	22.2
G1_L	BS2	Female	27.2	34.8
	BS4	Male	27.6	39.0
	BS6	Male	24.9	29.6
	BS8	Female	16.4	25.2
	BS10	Female	21.2	31.8
	BS12	Male	15.7	22.4
G2_R	BS13	Male	19.7	24.0
	BS15	Female	16.4	21.4
	BS17	Male	24.1	30.8
	BS19	Female	19.1	21.9
	BS21	Male	14.5	21.5
	BS23	Female	17.1	28.0
G2_L	BS14	Female	13.7	16.0
	BS16	Male	15.4	24.3
	BS18	Male	17.2	22.9
	BS20	Female	17.5	17.7
	BS22	Male	13.1	19.1
	BS24	Female	16.0	23.5
No_R	BS55	Male	16.7	26.0
	BS57	Female	15.5	26.7
	BS59	Male	17.1	23.4
	BS61	Female	16.6	26.9
	BS63b	Male	19.0	33.7
	BS65	Female	13.5	21.8
No_L	BS56	Male	13.2	21.3
	BS58b	Female	16.6	28.7
	BS60	Male	13.5	15.3
	BS62	Male	16.0	29.4
	BS64b	Male	19.9	33.0
	BS66	Female	16.8	28.3
Ca_R	BS43	Male	15.7	26.1
	BS45	Female	16.4	29.1
	BS47	Male	16.0	12.7
	BS49	Female	21.3	20.6
	BS51	Male	19.5	13.7
	BS53	Female	19.1	32.9
Ca_L	BS44	Male	15.6	12.1
	BS46	Female	14.7	21.2
	BS48	Male	15.4	22.6
	BS50b	Female	19.7	29.8
	BS52b	Male	23.6	40.5
	BS54	Female	17.7	31.1
Us_R	BS31b	Female	15.5	26.7
	BS33	Male	29.0	42.2
	BS35	Female	33.1	34.8
	BS37	Male	25.4	20.7
	BS39	Female	22.6	22.9
	BS41	Male	28.6	47.9
Us_L	BS32b	Female	17.9	30.2
	BS34b	Male	13.2	23.1
	BS36b	Female	18.1	30.6
	BS38b	Male	28.1	13.5
	BS40b	Female	26.3	46.3
	BS42b	Female	24.2	37.1
