# Supplementary material for: Genomics of Divergence along a Continuum of Parapatric Population Differentiation
Source: PLoS Genet. 2015 Feb 13;11(2):e1004966. doi: 10.1371/journal.pgen.1004966 (PMC4334544; doi:10.1371/journal.pgen.1004966)
Supplement: S3 Table — (PDF) [file pgen.1004966.s010.pdf]

**Table S3.** Summary statistics for each population.

| Population ID | SNPs | π | $N_e$ | $T_D$ | Θ | ρ/Θ |
|---|---|---|---|---|---|---|
| G1_R | 1410448 | 0.0017 | 22032 | 0.5693 | 0.0013 | 4.676 |
| G1_L | 1599340 | 0.0019 | 25081 | 0.3792 | 0.0015 | 3.639 |
| G2_R | 1597865 | 0.0019 | 25157 | 0.3321 | 0.0015 | 4.685 |
| G2_L | 1871737 | 0.0021 | 29255 | 0.1324 | 0.0017 | 5.500 |
| No_R | 965465 | 0.0012 | 14991 | 0.5461 | 0.0009 | 7.233 |
| No_L | 1859884 | 0.0021 | 29023 | 0.0922 | 0.0017 | 8.162 |
| Ca_R | 1710748 | 0.0020 | 26615 | 0.2985 | 0.0016 | 9.786 |
| Ca_L | 2426796 | 0.0029 | 37607 | 0.3471 | 0.0022 | 4.987 |
| Us_R | 2979553 | 0.0033 | 45634 | 0.0428 | 0.0027 | 6.732 |
| Us_L | 2755107 | 0.0031 | 41977 | 0.0601 | 0.0025 | 6.940 |

SNPs with a minor allele frequency above 5% are given.

Nucleotide diversity is given as an average across the autosomes.

Effective population size was estimated on the basis of Watterson's theta evaluated at intergenic regions assuming a mutation rate of $1.7 \times 10^{-8}$.
