# Supplementary material for: Genomics of Divergence along a Continuum of Parapatric Population Differentiation
Source: PLoS Genet. 2015 Feb 13;11(2):e1004966. doi: 10.1371/journal.pgen.1004966 (PMC4334544; doi:10.1371/journal.pgen.1004966)
Supplement: S4 Table — (PDF) [file pgen.1004966.s011.pdf]

Table S4. Summary statistics for each population pair.

Population-pair ID	SNPs	F_{ST}	D_{xy}	$D_{f\ (autosomes)}$	$D_{f\ (divergent\ regions)}$
G1	815073	0.2229	0.0022	1838	551
G2	797275	0.1147	0.0022	256	219
No	691957	0.2173	0.0019	178	94
Ca	1201431	0.2781	0.0031	4405	2049
Us	1227732	0.0949	0.0034	480	451

SNPs with a minor allele frequency above 25% are given to exclude uniformative sites.

D_f: fixed differences
