# Supplementary material for: Genomics of Divergence along a Continuum of Parapatric Population Differentiation
Source: PLoS Genet. 2015 Feb 13;11(2):e1004966. doi: 10.1371/journal.pgen.1004966 (PMC4334544; doi:10.1371/journal.pgen.1004966)
Supplement: S5 Table — (PDF) [file pgen.1004966.s012.pdf]

Table S5. Annotations for 47 shared outlier windows.

LG	Start	End	Outlier in Us	Outlier in G2	Outlier in No	Outlier in G1	Outlier in Ca	# populations	# genes	Gene ID	Gene name	Gene function
groupI	10820000	10830000	0	0	No	G1	0	2	2	ENSGACG00000010386; ENSGACG00000010389	C3orf33; MED13 (2 of 2)	nucleic acid binding, hydrolase activity, acting on ester bonds; regulation of transcription from RNA polymerase II promoter, mediator complex
groupI	17590000	17600000	0	0	No	G1	0	2	0	-	-	-
groupI	21970000	21980000	0	G2	0	G1	0	2	1	ENSGACG00000014407	-	nucleic acid binding, ATP binding, helicase activity, ATP-dependent helicase activity
groupII	130000	140000	0	G2	0	G1	0	2	1	ENSGACG00000013938	FAM108C1	hydrolase activity
groupII	12630000	12640000	0	G2	No	0	0	2	1	ENSGACG00000015990	-	-
groupIII	10260000	10270000	0	0	0	G1	Ca	2	0	-	-	-
groupIV	7070000	7080000	0	0	No	G1	0	2	0	-	-	-
groupIV	7080000	7090000	0	0	No	G1	0	2	0	-	-	-
groupIV	7090000	7100000	0	G2	No	G1	0	3	0	-	-	-
groupIV	11580000	11590000	Us	0	No	0	0	2	0	-	-	-
groupIV	13330000	13340000	Us	0	0	G1	0	2	2	ENSGACG00000018357; ENSGACG00000018359	RMND5B; N4BP3	protein binding, protein binding, protein binding; -
groupIV	13940000	13950000	Us	0	No	0	0	2	0	-	-	-
groupIV	19510000	19520000	Us	0	0	0	Ca	2	1	ENSGACG00000018927	-	-
groupIV	19520000	19530000	Us	0	0	G1	Ca	3	0	-	-	-
groupIV	19530000	19540000	Us	0	0	0	Ca	2	0	-	-	-
groupIV	23190000	23200000	NA	G2	No	0	NA	2	1	ENSGACG00000019249	AEBP2 (2 of 2)	intracellular, zinc ion binding
groupIX	9250000	9260000	0	G2	0	G1	0	2	1	ENSGACG00000017985	CTNNA2	cell adhesion, actin cytoskeleton, structural molecule activity
groupIX	9410000	9420000	0	G2	No	0	0	2	2	ENSGACG00000017996; ENSGACG00000018001	-; -	G-protein coupled receptor signaling pathway, integral to membrane; -
groupIX	11880000	11890000	0	G2	No	0	0	2	0	-	-	-
groupIX	15080000	15090000	0	G2	0	G1	0	2	2	ENSGACG00000018992; ENSGACG00000018993	SLC25A41; -	calcium ion binding, protein binding, calcium ion binding, protein binding; signal transducer activity
groupIX	15090000	15100000	0	G2	0	G1	0	2	2	ENSGACG00000018994; ENSGACG00000018995	C19orf53; MRI1	-; cellular metabolic process, cellular biosynthetic process
groupVII	13310000	13320000	Us	G2	0	0	0	2	1	ENSGACG00000020196	WNT11	multicellular organismal development, Wnt receptor signaling pathway, calcium modulating pathway, cell migration involved in gastrulation, heart development, extracellula
groupVII	14210000	14220000	Us	0	0	G1	0	2	1	ENSGACG00000020216	CLTC	intracellular protein transport, vesicle-mediated transport, clathrin coat of trans-Golgi network vesicle, clathrin coat of coated pit, binding, structural molecule activi
groupVII	18610000	18620000	Us	G2	0	0	0	2	1	ENSGACG00000020440	RBM14 (2 of 2)	nucleic acid binding
groupVII	18620000	18630000	0	G2	0	G1	0	2	1	ENSGACG00000020441	DPP3	proteolysis, cytoplasm, dipeptidyl-peptidase activity

groupVII	19250000	19260000	Us	0	No	0	0	2	6	ENSGACG00000020493; ENSGACG00000020494; ENSGACG00000022599; ENSGACG00000022586; ENSGACG00000022642; ENSGACG00000020495	GBAS (2 of 2); CCT6A; NA; NA; NA; SUMF2	metabolic process, transferase activity, folic acid binding; protein folding, cellular protein metabolic process, ATP binding, unfolded protein binding, protein folding, cell
groupVII	21410000	21420000	Us	0	0	0	Ca	2	1	ENSGACG00000020612	-	transmembrane transport, drug transmembrane transport, membrane, drug transmembrane transporter activity, antiporter activity, transmembrane transport, drug transmembrane t
groupVII	21430000	21440000	Us	NA	0	0	Ca	2	1	ENSGACG00000020613	-	transmembrane transport, drug transmembrane transport, membrane, drug transmembrane transporter activity, antiporter activity, transmembrane transport, drug transmembrane t
groupVIII	11870000	11880000	Us	G2	0	0	0	2	0	-	-	-
groupX	6530000	6540000	0	G2	0	G1	0	2	1	ENSGACG00000004219	BCAN (2 of 2)	cell adhesion, calcium ion binding, binding, protein binding, hyaluronic acid binding
groupX	15480000	15490000	0	G2	0	0	Ca	2	1	ENSGACG00000010362	-	protein phosphorylation, ATP binding, protein kinase activity, transferase activity, transferring phosphorus-containing groups, protein serine/threonine kinase activity
groupXI	1820000	1830000	0	G2	0	NA	Ca	2	0	-	-	-
groupXI	5550000	5560000	Us	G2	0	0	0	2	0	-	-	-
groupXII	12140000	12150000	Us	0	0	0	Ca	2	1	ENSGACG00000009758	TEAD3 (2 of 2)	regulation of transcription, DNA-dependent, nucleus, sequence-specific DNA binding transcription factor activity, protein binding
groupXIII	7980000	7990000	Us	0	No	0	0	2	1	ENSGACG00000007925	VPS13A	protein localization
groupXIII	7990000	8000000	Us	0	No	0	0	2	1	ENSGACG00000007925	VPS13A	protein localization
groupXIII	9210000	9220000	Us	0	0	G1	0	2	1	ENSGACG00000009222	ACACB	metabolic process, fatty acid biosynthetic process, ATP binding, catalytic activity, ligase activity, acetyl-CoA carboxylase activity
groupXIII	10660000	10670000	0	0	0	G1	Ca	2	2	ENSGACG00000010293; ENSGACG00000010296	WDR54; NOL6	protein binding; pathogenesis, RNA binding, hydrolase activity, acting on ester bonds, ribosome binding
groupXVIII	6210000	6220000	Us	G2	0	0	0	2	1	ENSGACG00000007328	HADHB	metabolic process, catalytic activity, transferase activity, transferring acyl groups other than amino-acyl groups
groupXX	8890000	8900000	0	0	NA	G1	Ca	2	2	ENSGACG00000007563; ENSGACG00000007569	-; -	regulation of Rab GTPase activity, intracellular, Rab GTPase activator activity; -
groupXX	9120000	9130000	0	G2	0	G1	0	2	1	ENSGACG00000007711	NPHS1	skeletal muscle fiber development, kidney development, protein binding
groupXX	9690000	9700000	Us	G2	0	0	0	2	1	ENSGACG00000008221	TAX1BP1 (1 of 2)	-
groupXX	9700000	9710000	Us	G2	0	0	0	2	2	ENSGACG00000008229; ENSGACG00000008221	HIBADH (2 of 2); TAX1BP1 (1 of 2)	oxidation-reduction process, valine metabolic process, pentose-phosphate shunt, NAD binding, 3-hydroxyisobutyrate dehydrogenase activity, phosphogluconate dehydrogenase (decarboxylating) activity

groupXX	12510000	12520000	0	0	No	G1	0	2	3	ENSGACG00000011540; ENSGACG00000011594; ENSGACG00000011547	SPHK2; -; RPL18	protein kinase C-activating G-protein coupled receptor signaling pathway, diacylglycerol kinase activity; integral to membrane; translation, intracellular, ribosome, structural con
groupXX	16020000	16030000	0	G2	0	G1	0	2	1	ENSGACG00000013259	RAB11A	regulation of transcription, DNA-dependent, signal transduction, GTP catabolic process, small GTPase mediated signal transduction, intracellular protein transport, nucleocytoplasm
groupXXI	1520000	1530000	Us	0	0	G1	0	2	0	-	-	-
groupXXI	4530000	4540000	Us	0	NA	G1	0	2	1	ENSGACG00000002390	CTNND2 (2 of 2)	binding, protein binding

Windows are denoted with population ID if they are an outlier window in the respective population comparison, 0 indicates no outlier status, and NA indicates no sequence polymorphism in the respective window and population comparison.
