# Supplementary material for: Genomics of Divergence along a Continuum of Parapatric Population Differentiation
Source: PLoS Genet. 2015 Feb 13;11(2):e1004966. doi: 10.1371/journal.pgen.1004966 (PMC4334544; doi:10.1371/journal.pgen.1004966)
Supplement: S6 Table — (PDF) [file pgen.1004966.s013.pdf]

Table S6. Annotations for 794 divergent regions.

LG	Start	End	Population	Type	Gene ID	Gene name	Gene function
groupI	8419280	8457378	G2	bg	-	-	-
groupI	10970914	11009914	G2	psL	ENSGACG00000010514	-	-
groupI	10970914	11009914	G2	psL	ENSGACG00000010517	HSPA8 (2 of 2)	-
groupI	10970914	11009914	G2	psL	ENSGACG00000010543	-	membrane, integral to membrane
groupI	10970914	11009914	G2	psL	ENSGACG00000010497	SYTL2 (2 of 2)	intracellular protein transport, protein binding, Rab GTPase binding
groupI	10970914	11009914	G2	psL	ENSGACG00000010555	-	protein binding
groupI	11536487	11604487	G2	bg	ENSGACG00000010795	-	intracellular signal transduction, protein binding
groupI	11536487	11604487	G2	bg	ENSGACG00000010797	SAMD4B	binding, protein binding, binding, protein binding
groupI	11536487	11604487	G2	bg	ENSGACG00000010818	PAF1	Cdc73/Paf1 complex
groupI	12513891	12549891	G2	psL	ENSGACG00000011343	RRP8	metabolic process, methyltransferase activity
groupI	12513891	12549891	G2	psL	ENSGACG00000011369	APBB1	-
groupI	12513891	12549891	G2	psL	ENSGACG00000011342	-	-
groupI	12513891	12549891	G2	psL	ENSGACG00000011346	ILK (1 of 2)	protein phosphorylation, ATP binding, protein kinase activity, protein binding, transferase activity, transferring phosphorus-containing groups, protein serine/threonine kinase activity
groupI	14756748	14768748	G2	psR	ENSGACG00000012128	MAML2	-
groupI	16278408	16314408	G2	n	ENSGACG00000012634	GPR4	G-protein coupled receptor signaling pathway, integral to membrane
groupI	16278408	16314408	G2	n	ENSGACG00000012628	-	RNA binding
groupI	18371588	18386686	G2	psR	ENSGACG00000013496	-	oxidation-reduction process, metabolic process, oxidoreductase activity, oxidation-reduction process, metabolic process, oxidoreductase activity
groupI	18371588	18386686	G2	psR	ENSGACG00000013509	-	oxidation-reduction process, metabolic process, oxidoreductase activity
groupI	19806765	19854765	G2	psR	ENSGACG00000013701	-	-
groupI	21957552	21995029	G2	n	ENSGACG00000014396	TGDS	cellular metabolic process, biosynthetic process, steroid biosynthetic process, extracellular polysaccharide biosynthetic process, catalytic activity, coenzyme binding, 3-beta-hydroxy-delta5-steroid dehydrogenase activity, dTDP-4-dehydrorhamnose reductase activity
groupI	21957552	21995029	G2	n	ENSGACG00000014388	GPR180	mitochondrial respiratory chain, electron carrier activity, cytochrome-c oxidase activity
groupI	21957552	21995029	G2	n	ENSGACG00000014407	-	nucleic acid binding, ATP binding, helicase activity, ATP-dependent helicase activity
groupI	21957552	21995029	G2	n	ENSGACG00000014419	PTGFRN	protein binding
groupI	24819830	24846830	G2	bg	ENSGACG00000015056	IL1RAPL1 (1 of 2)	signal transduction, innate immune response, intrinsic to membrane, transmembrane signaling receptor activity, protein binding
groupII	124348	151348	G2	n	ENSGACG00000013938	FAM108C1	hydrolase activity
groupII	152317	191665	G2	bg	ENSGACG00000013947	ARNT2	regulation of transcription, DNA-dependent, signal transduction, nucleus, signal transducer activity, protein binding, regulation of transcription, DNA-dependent, signal transduction, response to hypoxia, dopaminergic neuron differentiation, diencephalon development, hypothalamus cell differentiation, cell-cell adhesion, positive regulation of transcription, DNA-dependent, nucleus, signal transducer activity, protein binding
groupII	208211	239211	G2	psL	ENSGACG00000013959	FAH	metabolic process, aromatic amino acid family metabolic process, catalytic activity, fumarylacetoacetase activity
groupII	208211	239211	G2	psL	ENSGACG00000013974	MTHFS	folic acid-containing compound biosynthetic process, ATP binding, 5-formyltetrahydrofolate cyclo-ligase activity
groupII	208211	239211	G2	psL	ENSGACG00000013982	ASB7	intracellular signal transduction, protein binding
groupII	777336	823336	G2	n	-	-	-
groupII	6901119	7035403	G2	bg	-	-	-
groupII	10838336	10855336	G2	psL	ENSGACG00000015779	ZFHX3	regulation of transcription, DNA-dependent, metal ion transport, nucleus, intracellular, sequence-specific DNA binding transcription factor activity, nucleic acid binding, zinc ion binding, metal ion binding, sequence-specific DNA binding, protein binding
groupII	12627579	12659579	G2	n	ENSGACG00000015990	-	-
groupII	12661976	12694350	G2	psR	ENSGACG00000015992	-	-
groupII	13529008	13545449	G2	psR	ENSGACG00000016129	FAM120B	DNA repair, nuclease activity
groupII	13577214	13591326	G2	psR	ENSGACG00000016137	MIS12	cell cycle, mitosis, nucleus, chromosome, centromeric region

groupII	13577214	13591326	G2	psR	ENSGACG00000016139	PPM1G	protein dephosphorylation, protein serine/threonine phosphatase complex, catalytic activity, protein serine/threonine phosphatase activity
groupII	13607939	13656324	G2	n	ENSGACG00000016144	PDCD2	cytoplasm, zinc ion binding
groupII	13607939	13656324	G2	n	ENSGACG00000016147	TBP	regulation of transcription, DNA-dependent, transcription initiation from RNA polymerase II promoter, gastrulation, mRNA catabolic process, transcription from RNA polymerase II promoter, DNA binding
groupII	13607939	13656324	G2	n	ENSGACG00000016151	PSMB1	proteolysis involved in cellular protein catabolic process, proteasome core complex, endopeptidase activity, threonine-type endopeptidase activity
groupII	13607939	13656324	G2	n	ENSGACG00000016158	PHF10	zinc ion binding, protein binding
groupII	13607939	13656324	G2	n	ENSGACG00000016166	CCSAP (1 of 2)	-
groupII	13607939	13656324	G2	n	ENSGACG00000016168	ACTC1 (3 of 3)	ATP binding
groupII	13607939	13656324	G2	n	ENSGACG00000016174	-	-
groupII	13607939	13656324	G2	n	ENSGACG00000016176	ABCB10	transport, transmembrane transport, integral to membrane, ATP binding, nucleotide binding, ATPase activity, ATPase activity, coupled to transmembrane movement of substances, nucleoside-triphosphatase activity
groupII	13607939	13656324	G2	n	ENSGACG00000016150	PSMC3	protein catabolic process, cytoplasm, ATP binding, hydrolase activity, nucleotide binding, nucleoside-triphosphatase activity, protein catabolic process, cytoplasm, ATP binding, hydrolase activity, nucleotide binding, nucleoside-triphosphatase activity
groupII	13607939	13656324	G2	n	ENSGACG00000016164	RAB4A	signal transduction, GTP catabolic process, small GTPase mediated signal transduction, intracellular protein transport, nucleocytoplasmic transport, protein transport, intracellular, GTPase activity, GTP binding
groupII	14299098	14317098	G2	n	-	-	-
groupIII	5103212	5145627	G2	psR	ENSGACG00000014579	-	G-protein coupled receptor signaling pathway, integral to membrane
groupIII	5103212	5145627	G2	psR	ENSGACG00000014581	CNT-P2-(2-of-2)	cell adhesion, calcium ion binding, protein binding
groupIII	5103212	5145627	G2	psR	ENSGACG00000014583	TMEM70	-
groupIII	8517725	8559943	G2	psR	ENSGACG00000015576	KCNN1 (1 of 2)	potassium ion transport, integral to membrane, calcium-activated potassium channel activity, calmodulin binding
groupIII	8815498	8872573	G2	psL	ENSGACG00000015666	FAM69A (2 of 2)	-
groupIII	8815498	8872573	G2	psL	ENSGACG00000015670	RPL5 (1 of 3)	translation, intracellular, ribosome, structural constituent of ribosome
groupIII	8815498	8872573	G2	psL	ENSGACG00000022640	-	-
groupIII	8815498	8872573	G2	psL	ENSGACG00000022680	-	-
groupIII	8815498	8872573	G2	psL	ENSGACG00000022637	-	-
groupIII	8815498	8872573	G2	psL	ENSGACG00000015672	-	-
groupIII	8815498	8872573	G2	psL	ENSGACG00000015674	-	intracellular, DNA binding, zinc ion binding, intracellular, DNA binding, zinc ion binding
groupIII	8815498	8872573	G2	psL	ENSGACG00000015678	RPAP2	-
groupIII	8815498	8872573	G2	psL	ENSGACG00000015634	-	actin binding
groupIII	8815498	8872573	G2	psL	ENSGACG00000015662	CCDC18	-
groupIII	8815498	8872573	G2	psL	ENSGACG00000015665	MTF2	nucleic acid binding, DNA binding, zinc ion binding, protein binding
groupIII	8922068	8940068	G2	psL	ENSGACG00000015695	LRRC8D (2 of 2)	protein binding
groupIII	8922068	8940068	G2	psL	ENSGACG00000015696	GTF2B	regulation of transcription, DNA-dependent, translational initiation, zinc ion binding, translation initiation factor activity
groupIII	8922068	8940068	G2	psL	ENSGACG00000015702	-	signal transduction, protein phosphorylation, intracellular, ATP binding, protein kinase activity, protein binding, transferase activity, transferring phosphorus-containing groups, protein serine/threonine kinase activity
groupIII	9413821	9445821	G2	bg	ENSGACG00000016113	B3GALT2 (2 of 2)	protein glycosylation, membrane, galactosyltransferase activity
groupIII	9413821	9445821	G2	bg	ENSGACG00000016114	CDC73 (1 of 2)	-
groupIII	9413821	9445821	G2	bg	ENSGACG00000016121	RGS21	signal transducer activity
groupIII	9413821	9445821	G2	bg	ENSGACG00000016122	RGS18	signal transducer activity
groupIII	9413821	9445821	G2	bg	ENSGACG00000016106	CDC73 (2 of 2)	-
groupIII	9413821	9445821	G2	bg	ENSGACG00000016116	-	signal transducer activity
groupIII	11094714	11126112	G2	n	ENSGACG00000016640	SOGA2	-
groupIII	11272658	11327703	G2	psL	ENSGACG00000016675	-	-
groupIII	11272658	11327703	G2	psL	ENSGACG00000016674	EPB41L3	cortical actin cytoskeleton organization, cytoskeleton, actin binding, structural molecule activity, cytoskeletal protein binding
groupIII	11272658	11327703	G2	psL	ENSGACG00000016677	-	protein dephosphorylation, dephosphorylation, membrane, protein tyrosine phosphatase activity, phosphatase activity, protein binding
groupIII	12058014	12070014	G2	psR	ENSGACG00000016820	-	protein dephosphorylation, dephosphorylation, protein tyrosine phosphatase activity, phosphatase activity, protein binding

groupIII	12235740	12250742	G2	psL	ENSGACG00000016890	-	intracellular, zinc ion binding
groupIII	12235740	12250742	G2	psL	ENSGACG00000016893	EPS15L1 (2 of 2)	signal transduction, intracellular, calcium ion binding, protein binding
groupIV	7072653	7108653	G2	psR	-	-	-
groupIV	11318370	11348795	G2	psR	ENSGACG00000018081	-	-
groupIV	11869871	11885871	G2	psR	ENSGACG00000018209	NR3C1 (1 of 2)	regulation of transcription, DNA-dependent, steroid hormone mediated signaling pathway, glucocorticoid receptor signaling pathway, glucocorticoid mediated signaling pathway, response to dexamethasone stimulus, response to cortisol stimulus, response to glucocorticoid stimulus, calcium ion import, embryo development, positive regulation of transcription, DNA-dependent, nucleus, cytoplasm, sequence-specific DNA binding transcription factor activity, steroid hormone receptor activity, DNA binding, zinc ion binding, steroid binding, sequence-specific DNA binding, glucocorticoid receptor activity
groupIV	14478349	14529546	G2	psL	ENSGACG00000018483	-	proteolysis, protein ubiquitination, ubiquitin ligase complex, zinc ion binding, protein binding, ATP-dependent peptidase activity, ubiquitin-protein ligase activity
groupIV	14478349	14529546	G2	psL	ENSGACG00000018485	ITK	intracellular signal transduction, protein phosphorylation, ATP binding, protein kinase activity, protein binding, transferase activity, transferring phosphorus-containing groups, protein serine/threonine kinase activity, protein tyrosine kinase activity
groupIV	14478349	14529546	G2	psL	ENSGACG00000018480	GRIA1 (2 of 2)	transport, membrane, outer membrane-bounded periplasmic space, transporter activity, ionotropic glutamate receptor activity, extracellular-glutamate-gated ion channel activity, transport, membrane, outer membrane-bounded periplasmic space, transporter activity, ionotropic glutamate receptor activity, extracellular-glutamate-gated ion channel activity
groupIV	14478349	14529546	G2	psL	ENSGACG00000018484	MED7	regulation of transcription from RNA polymerase II promoter, mediator complex
groupIV	14606821	14680160	G2	bg	-	-	-
groupIV	14776106	14835414	G2	psR	-	-	-
groupIV	16428671	16454671	G2	n	ENSGACG00000018710	GABRA4	transport, ion transport, membrane, integral to membrane, postsynaptic membrane, extracellular ligand-gated ion channel activity
groupIV	21481867	21549867	G2	n	ENSGACG00000019079	-	-
groupIV	21481867	21549867	G2	n	ENSGACG00000019082	LRMP	-
groupIV	21481867	21549867	G2	n	ENSGACG00000019084	CASC1 (1 of 2)	-
groupIV	21481867	21549867	G2	n	ENSGACG00000019086	LYRM5	-
groupIV	21481867	21549867	G2	n	ENSGACG00000019087	SLC5A8	transport, transmembrane transport, membrane, transporter activity
groupIV	21481867	21549867	G2	n	ENSGACG00000019080	-	-
groupIV	21481867	21549867	G2	n	ENSGACG00000019092	UTP20	-
groupIV	21481867	21549867	G2	n	ENSGACG00000019077	BCAT1	metabolic process, catalytic activity
groupIV	21712176	21759548	G2	n	ENSGACG00000019120	FAM19A2	-
groupIV	22473325	22603535	G2	n	ENSGACG00000019202	C1orf31	mitochondrion, cytochrome-c oxidase activity, mitochondrion, cytochrome-c oxidase activity
groupIV	22473325	22603535	G2	n	ENSGACG00000019206	-	-
groupIV	22473325	22603535	G2	n	ENSGACG00000019208	ARNTL2	regulation of transcription, DNA-dependent, signal transduction, photoperiodism, response to light stimulus, circadian rhythm, nucleus, signal transducer activity, protein binding
groupIV	22473325	22603535	G2	n	ENSGACG00000019215	SIL1	binding, binding
groupIV	22473325	22603535	G2	n	ENSGACG00000019193	PTPRZ1 (2 of 2)	protein dephosphorylation, dephosphorylation, protein tyrosine phosphatase activity, phosphatase activity, protein binding
groupIV	22473325	22603535	G2	n	ENSGACG00000019204	SLC13A4	sodium ion transport, transmembrane transport, membrane, transporter activity
groupIV	22473325	22603535	G2	n	ENSGACG00000019212	TRAPPC11	retina development in camera-type eye
groupIV	22473325	22603535	G2	n	ENSGACG00000019214	CCDC99	-
groupIV	22958919	23020919	G2	n	ENSGACG00000019233	-	positive regulation of Wnt receptor signaling pathway, positive regulation of Wnt receptor signaling pathway involved in dorsal/ventral axis specification, positive regulation of canonical Wnt receptor signaling pathway, dorsal/ventral pattern formation, protein binding
groupIV	22958919	23020919	G2	n	ENSGACG00000019236	IPO8	intracellular protein transport, binding, protein transporter activity
groupIV	22958919	23020919	G2	n	ENSGACG00000019230	-	-
groupIV	23139373	23280181	G2	-	ENSGACG00000019247	GPR22 (2 of 2)	G-protein coupled receptor signaling pathway, integral to membrane, ATP binding
groupIV	23139373	23280181	G2	-	ENSGACG00000019249	AEBP2 (2 of 2)	intracellular, zinc ion binding
groupIV	23139373	23280181	G2	-	ENSGACG00000019242	COG5	-
groupIV	23139373	23280181	G2	-	ENSGACG00000019248	NDUFA5	respiratory electron transport chain, mitochondrial inner membrane, oxidoreductase activity, acting on NADH or NADPH
groupIV	23139373	23280181	G2	-	ENSGACG00000019253	PLEKHA5 (2 of 2)	protein binding

groupIV	23292642	23429982	G2	n	ENSGACG00000019257	BRAF	signal transduction, intracellular signal transduction, protein phosphorylation, ATP binding, protein kinase activity, transferase activity, transferring phosphorus-containing groups, protein serine/threonine kinase activity, receptor signaling protein activity, signal transduction, intracellular signal transduction, protein phosphorylation, ATP binding, protein kinase activity, transferase activity, transferring phosphorus-containing groups, protein serine/threonine kinase activity, receptor signaling protein activity
groupIV	23292642	23429982	G2	n	ENSGACG00000019259	C9orf100	regulation of Rho protein signal transduction, intracellular, protein binding, Rho guanyl-nucleotide exchange factor activity
groupIV	23581475	23713478	G2	psR	ENSGACG00000019267	MGST1	-
groupIV	23581475	23713478	G2	psR	ENSGACG00000019269	DERA	deoxyribonucleotide catabolic process, cytoplasm, lyase activity, deoxyribose-phosphate aldolase activity
groupIV	23581475	23713478	G2	psR	ENSGACG00000019264	LMO3	zinc ion binding
groupIV	24970842	24986216	G2	psR	ENSGACG00000019459	NAP1L1	nucleosome assembly, nucleus
groupIV	25333833	25361833	G2	psR	ENSGACG00000019472	NAV3	protein binding
groupIV	26458518	26484028	G2	psR	ENSGACG00000019609	SLC7A11	transport, transmembrane transport, metal ion transport, membrane, metal ion binding
groupIV	29218514	29258520	G2	psR	ENSGACG00000019718	PLXNA4	signal transduction, axonal defasciculation, morphogenesis of a branching structure, axon guidance, membrane, intracellular, catalytic activity, protein binding
groupIV	31072967	31081967	G2	psR	-	-	-
groupIX	4547838	4562838	G2	n	ENSGACG00000016566	TRIP10 (2 of 2)	signal transduction, intracellular, protein binding
groupIX	4547838	4562838	G2	n	ENSGACG00000016568	SH2D3A	small GTPase mediated signal transduction, intracellular, protein binding, guanyl-nucleotide exchange factor activity
groupIX	8439431	8479686	G2	psR	ENSGACG00000017608	MOGS	oligosaccharide metabolic process, catalytic activity, mannosyl-oligosaccharide glucosidase activity
groupIX	8439431	8479686	G2	psR	ENSGACG00000017610	-	-
groupIX	8439431	8479686	G2	psR	ENSGACG00000017612	EGR4	intracellular, DNA binding, zinc ion binding
groupIX	8439431	8479686	G2	psR	ENSGACG00000017601	-	-
groupIX	8550530	8581532	G2	psR	ENSGACG00000017636	CLDND1 (1 of 2)	-
groupIX	8550530	8581532	G2	psR	ENSGACG00000017646	ACY3	metabolic process, hydrolase activity, acting on ester bonds
groupIX	8550530	8581532	G2	psR	ENSGACG00000017657	-	protein ADP-ribosylation, NAD(P)+-protein-arginine ADP-ribosyltransferase activity
groupIX	8550530	8581532	G2	psR	ENSGACG00000017658	CCDC111	-
groupIX	8550530	8581532	G2	psR	ENSGACG00000017633	GLB1	carbohydrate metabolic process, beta-galactosidase complex, hydrolase activity, hydrolyzing O-glycosyl compounds, beta-galactosidase activity
groupIX	8550530	8581532	G2	psR	ENSGACG00000017653	TACR3 (1 of 2)	G-protein coupled receptor signaling pathway, integral to membrane
groupIX	8760343	8774343	G2	n	ENSGACG00000017860	ACER2	ceramide metabolic process, integral to membrane, hydrolase activity, acting on carbon-nitrogen (but not peptide) bonds, in linear amides
groupIX	8760343	8774343	G2	n	ENSGACG00000017858	TSPAN5 (1 of 2)	mitochondrial electron transport, cytochrome c to oxygen, membrane, integral to membrane, cytochrome-c oxidase activity
groupIX	9242584	9343815	G2	bg	ENSGACG00000017985	CTNNA2	cell adhesion, actin cytoskeleton, structural molecule activity
groupIX	9508292	9539512	G2	psL	ENSGACG00000018013	PRSS12	proteolysis, membrane, catalytic activity, serine-type endopeptidase activity, scavenger receptor activity, proteolysis, membrane, catalytic activity, serine-type endopeptidase activity, scavenger receptor activity
groupIX	9508292	9539512	G2	psL	ENSGACG00000018022	NDST4	DNA recombination, double-strand break repair via homologous recombination, regulation of S phase of mitotic cell cycle, nucleus, single-stranded DNA binding, sulfotransferase activity
groupIX	9798894	9869988	G2	psL	ENSGACG00000018054	SERPING1	protein binding
groupIX	9798894	9869988	G2	psL	ENSGACG00000018056	TMEM134	-
groupIX	9798894	9869988	G2	psL	ENSGACG00000018060	AIP	protein folding, protein binding
groupIX	9798894	9869988	G2	psL	ENSGACG00000018065	CDK2AP2	-
groupIX	9798894	9869988	G2	psL	ENSGACG00000018070	ANKRD49	protein binding
groupIX	9798894	9869988	G2	psL	ENSGACG00000018071	GRIA2 (2 of 2)	transport, membrane, outer membrane-bounded periplasmic space, transporter activity, ionotropic glutamate receptor activity, extracellular-glutamate-gated ion channel activity
groupIX	9798894	9869988	G2	psL	ENSGACG00000018066	GPR83	G-protein coupled receptor signaling pathway, integral to membrane
groupIX	9872142	9945914	G2	psL	ENSGACG00000018088	-	intracellular, DNA binding, zinc ion binding
groupIX	9872142	9945914	G2	psL	ENSGACG00000018071	GRIA2 (2 of 2)	transport, membrane, outer membrane-bounded periplasmic space, transporter activity, ionotropic glutamate receptor activity, extracellular-glutamate-gated ion channel activity
groupIX	9872142	9945914	G2	psL	ENSGACG00000018090	EEF1A1	translational elongation, cytoplasm, GTPase activity, GTP binding, translation elongation factor activity
groupIX	9872142	9945914	G2	psL	ENSGACG00000018097	KCNQ5 (1 of 2)	ion transport, transmembrane transport, membrane, ion channel activity

groupIX	10015553	10081814	G2	psR	ENSGACG00000018108	-	-
groupIX	10015553	10081814	G2	psR	ENSGACG00000018110	KRT222	-
groupIX	10015553	10081814	G2	psR	ENSGACG00000018111	TNS4	protein binding
groupIX	10015553	10081814	G2	psR	ENSGACG00000018112	CCR7	G-protein coupled receptor signaling pathway, integral to membrane
groupIX	10015553	10081814	G2	psR	ENSGACG00000018104	RIMS1 (2 of 2)	transcription, DNA-dependent, intracellular protein transport, DNA binding, zinc ion binding, ATP binding, protein binding, DNA-directed RNA polymerase activity, Rab GTPase binding, protein binding, transcription, DNA-dependent, intracellular protein transport, DNA binding, zinc ion binding, protein binding, DNA-directed RNA polymerase activity, Rab GTPase binding
groupIX	10015553	10081814	G2	psR	ENSGACG00000018109	-	-
groupIX	10015553	10081814	G2	psR	ENSGACG00000018114	SMARCE1 (1 of 2)	DNA binding, protein binding
groupIX	10015553	10081814	G2	psR	ENSGACG00000018120	IGFBP4	regulation of cell growth, extracellular region, insulin-like growth factor binding
groupIX	10168106	10238394	G2	psR	ENSGACG00000018158	-	-
groupIX	10168106	10238394	G2	psR	ENSGACG00000018165	QPRT	NAD biosynthetic process, pyridine nucleotide biosynthetic process, nicotinate-nucleotide diphosphorylase (carboxylating) activity, transferase activity, transferring pentosyl groups, NAD biosynthetic process, pyridine nucleotide biosynthetic process, nicotinate-nucleotide diphosphorylase (carboxylating) activity, transferase activity, transferring pentosyl groups
groupIX	10168106	10238394	G2	psR	ENSGACG00000018168	PPP1R12C	protein binding
groupIX	10168106	10238394	G2	psR	ENSGACG00000018170	ARHGDIA (2 of 2)	cytoplasm, Rho GDP-dissociation inhibitor activity
groupIX	10168106	10238394	G2	psR	ENSGACG00000018178	ALYREF (2 of 2)	nucleic acid binding
groupIX	10168106	10238394	G2	psR	ENSGACG00000018183	ANAPC11	zinc ion binding, protein binding
groupIX	10168106	10238394	G2	psR	ENSGACG00000018184	-	protein binding
groupIX	10168106	10238394	G2	psR	ENSGACG00000018185	P4HB	cell redox homeostasis, endoplasmic reticulum, isomerase activity
groupIX	10168106	10238394	G2	psR	ENSGACG00000018160	PLA2G12B (1 of 2)	lipid catabolic process, extracellular region, calcium ion binding, phospholipase A2 activity
groupIX	10168106	10238394	G2	psR	ENSGACG00000018163	ITGAL	protein binding
groupIX	10329724	10371179	G2	n	ENSGACG00000018193	RPTOR	binding, protein binding
groupIX	11850949	11931949	G2	psR	ENSGACG00000018410	PCDH18 (1 of 2)	cell adhesion, homophilic cell adhesion, epiboly involved in gastrulation with mouth forming second, calcium-dependent cell-cell adhesion, cell migration involved in gastrulation, membrane, plasma membrane, calcium ion binding
groupIX	13305317	13323317	G2	psR	ENSGACG00000018581	SGCZ (1 of 2)	cytoskeleton organization, integral to membrane, sarcoglycan complex
groupIX	14387342	14405386	G2	psR	ENSGACG00000018766	SRL (2 of 2)	-
groupIX	14387342	14405386	G2	psR	ENSGACG00000018768	TFAP4 (2 of 2)	regulation of transcription, DNA-dependent, nucleus
groupIX	14516765	14540335	G2	-	ENSGACG00000018792	TMED1 (1 of 2)	transport, integral to membrane
groupIX	14516765	14540335	G2	-	ENSGACG00000018797	DNM2 (1 of 2)	GTPase activity, GTP binding, protein binding, IMP cyclohydrolase activity
groupIX	14560561	14611719	G2	n	ENSGACG00000018810	TECR (1 of 2)	lipid metabolic process, integral to membrane, cytoplasm, oxidoreductase activity, acting on the CH-CH group of donors
groupIX	14560561	14611719	G2	n	ENSGACG00000018831	NDUFB7	mitochondrion, NADH dehydrogenase (ubiquinone) activity, NADH dehydrogenase activity
groupIX	14560561	14611719	G2	n	ENSGACG00000018834	SAMD1 (2 of 2)	nucleus, sequence-specific DNA binding, protein binding
groupIX	14560561	14611719	G2	n	ENSGACG00000018839	ASF1B (1 of 2)	regulation of transcription, DNA-dependent, chromatin assembly or disassembly, nucleus, sequence-specific DNA binding transcription factor activity
groupIX	14560561	14611719	G2	n	ENSGACG00000018800	D-JB1-(1-of-2)	protein folding, unfolded protein binding, heat shock protein binding
groupIX	14560561	14611719	G2	n	ENSGACG00000018832	-	ATP binding, hydrolase activity, binding, nucleotide binding, ATPase activity, nucleoside-triphosphatase activity
groupIX	14560561	14611719	G2	n	ENSGACG00000018837	-	protein phosphorylation, ATP binding, protein kinase activity, transferase activity, transferring phosphorus-containing groups, protein serine/threonine kinase activity
groupIX	15060706	15238842	G2	n	ENSGACG00000018987	RFX1 (2 of 2)	regulation of transcription, DNA-dependent, nucleus, DNA binding
groupIX	15060706	15238842	G2	n	ENSGACG00000018989	DCAF15	-
groupIX	15060706	15238842	G2	n	ENSGACG00000018992	SLC25A41	calcium ion binding, protein binding, calcium ion binding, protein binding
groupIX	15060706	15238842	G2	n	ENSGACG00000018994	C19orf53	-
groupIX	15060706	15238842	G2	n	ENSGACG00000018995	MRI1	cellular metabolic process, cellular biosynthetic process
groupIX	15060706	15238842	G2	n	ENSGACG00000019002	-	-
groupIX	15060706	15238842	G2	n	ENSGACG00000019003	NANOS3	female gamete generation, oocyte growth, nucleic acid binding, zinc ion binding, RNA binding
groupIX	15060706	15238842	G2	n	ENSGACG00000019006	SLC26A11	transport, transmembrane transport, sulfate transport, membrane, integral to membrane, transporter activity, secondary active sulfate transmembrane transporter activity

groupIX	15060706	15238842	G2	n	ENSGACG00000019013	NPB (1 of 2)	-
groupIX	15060706	15238842	G2	n	ENSGACG00000019035	SIRT7	regulation of transcription, DNA-dependent, chromatin silencing, protein deacetylation, zinc ion binding, hydrolase activity, acting on carbon-nitrogen (but not peptide) bonds, in linear amides, NAD+ binding
groupIX	15060706	15238842	G2	n	ENSGACG00000019039	-	-
groupIX	15060706	15238842	G2	n	ENSGACG00000019041	MAFG	-
groupIX	15060706	15238842	G2	n	ENSGACG00000018993	-	signal transducer activity
groupIX	15060706	15238842	G2	n	ENSGACG00000018999	CC2D1A	protein binding
groupIX	15060706	15238842	G2	n	ENSGACG00000019004	-	membrane, scavenger receptor activity
groupIX	15060706	15238842	G2	n	ENSGACG00000019010	SGSH	metabolic process, catalytic activity, sulfuric ester hydrolase activity
groupIX	15060706	15238842	G2	n	ENSGACG00000019015	-	signal transduction, G-protein coupled receptor signaling pathway, signal transducer activity, guanyl nucleotide binding
groupIX	15060706	15238842	G2	n	ENSGACG00000018990	KHSRP (2 of 2)	RNA binding
groupIX	15060706	15238842	G2	n	ENSGACG00000019032	PCYT2 (1 of 2)	biosynthetic process, catalytic activity, nucleotidyltransferase activity
groupIX	16135754	16206218	G2	psR	ENSGACG00000019256	SRP68	protein binding
groupIX	16135754	16206218	G2	psR	ENSGACG00000019260	-	-
groupIX	16135754	16206218	G2	psR	ENSGACG00000019266	TEN1	-
groupIX	16135754	16206218	G2	psR	ENSGACG00000019268	ACOX1	oxidation-reduction process, metabolic process, fatty acid beta-oxidation, peroxisome, oxidoreductase activity, acting on the CH-CH group of donors, acyl-CoA oxidase activity, acyl-CoA dehydrogenase activity
groupIX	16135754	16206218	G2	psR	ENSGACG00000019276	AA-T-(1-of-3)	metabolic process, N-acetyltransferase activity
groupIX	16135754	16206218	G2	psR	ENSGACG00000019254	DDX5 (2 of 2)	nucleic acid binding, ATP binding, helicase activity, ATP-dependent helicase activity
groupIX	16135754	16206218	G2	psR	ENSGACG00000019272	RHBDF2	integral to membrane, serine-type endopeptidase activity
groupV	1224812	1243812	G2	n	ENSGACG00000002546	OAT	pyridoxal phosphate binding, transaminase activity
groupV	1224812	1243812	G2	n	ENSGACG00000002578	LHPP	metabolic process, catalytic activity
groupVI	3404108	3425287	G2	bg	ENSGACG00000003502	C10orf54	-
groupVI	3404108	3425287	G2	bg	ENSGACG00000003475	CDH23	cell adhesion, homophilic cell adhesion, detection of mechanical stimulus involved in sensory perception, inner ear receptor stereocilium organization, hair cell differentiation, cell projection organization, sensory perception of sound, equilibrioception, endocytosis, membrane, plasma membrane, cilium, calcium ion binding
groupVI	13209781	13223165	G2	psR	ENSGACG00000010558	TNFAIP3	DNA binding, zinc ion binding
groupVI	13209781	13223165	G2	psR	ENSGACG00000010566	PERP	membrane
groupVII	12440865	12449865	G2	psL	-	-	-
groupVII	13310286	13323703	G2	n	ENSGACG00000020196	WNT11	multicellular organismal development, Wnt receptor signaling pathway, calcium modulating pathway, cell migration involved in gastrulation, heart development, extracellular region
groupVII	14576890	14634661	G2	psR	ENSGACG00000020233	HSPB1	response to arsenic-containing substance, response to heat, cytoplasm, nucleus
groupVII	14576890	14634661	G2	psR	ENSGACG00000020234	YWHAG (1 of 2)	brain development, heart development
groupVII	14576890	14634661	G2	psR	ENSGACG00000020235	SRCRB4D	membrane, scavenger receptor activity
groupVII	18002763	18019506	G2	n	ENSGACG00000020402	MPDU1 (2 of 2)	-
groupVII	18002763	18019506	G2	n	ENSGACG00000020403	-	-
groupVII	18174445	18191445	G2	psR	ENSGACG00000020415	-	protein binding
groupVII	18586964	18633965	G2	psL	ENSGACG00000020442	CD248 (2 of 2)	binding
groupVII	18586964	18633965	G2	psL	ENSGACG00000020439	-	protein binding, phospholipid binding
groupVII	18586964	18633965	G2	psL	ENSGACG00000020441	DPP3	proteolysis, cytoplasm, dipeptidyl-peptidase activity
groupVII	18586964	18633965	G2	psL	ENSGACG00000020440	RBM14 (2 of 2)	nucleic acid binding
groupVII	22146415	22169415	G2	n	ENSGACG00000020667	-	transport, ion transport, membrane, integral to membrane, postsynaptic membrane, extracellular ligand-gated ion channel activity, transport, ion transport, membrane, integral to membrane, postsynaptic membrane, extracellular ligand-gated ion channel activity
groupVII	22146415	22169415	G2	n	ENSGACG00000020668	PHLDB2 (2 of 2)	protein binding
groupVIII	5868880	5882072	G2	-	ENSGACG00000005939	-	ligase activity
groupVIII	11868896	11881896	G2	psR	-	-	-

groupVIII	12045594	12136594	G2	psR	ENSGACG00000009517	ELTD1	G-protein coupled receptor signaling pathway, cell surface receptor signaling pathway, neuropeptide signaling pathway, membrane, integral to membrane, G-protein coupled receptor activity, calcium ion binding, transmembrane signaling receptor activity, protein binding
groupVIII	19195726	19223726	G2	n	ENSGACG00000014574	-	binding, protein binding
groupVIII	19195726	19223726	G2	n	ENSGACG00000014584	SELRC1	-
groupVIII	19195726	19223726	G2	n	ENSGACG00000014569	ECHDC2	metabolic process, catalytic activity
groupVIII	19195726	19223726	G2	n	ENSGACG00000014587	-	small GTPase mediated signal transduction, intracellular, protein binding, guanyl-nucleotide exchange factor activity
groupX	3305886	3317993	G2	n	ENSGACG00000002923	PRRT1	-
groupX	3305886	3317993	G2	n	ENSGACG00000002927	SKIV2L	regulation of transcription, DNA-dependent, transcription initiation, DNA-dependent, sequence-specific DNA binding transcription factor activity, nucleic acid binding, DNA binding, ATP binding, nucleotide binding, hydrolase activity, acting on acid anhydrides, in phosphorus-containing anhydrides, sigma factor activity, helicase activity, ATP-dependent helicase activity, nucleoside-triphosphatase activity
groupX	6362007	6594901	G2	psR	ENSGACG00000004109	DUS3L	oxidation-reduction process, tRNA processing, nucleic acid binding, zinc ion binding, flavin adenine dinucleotide binding, tRNA dihydrouridine synthase activity
groupX	6362007	6594901	G2	psR	ENSGACG00000004141	-	-
groupX	6362007	6594901	G2	psR	ENSGACG00000004161	CYP51A1	iron ion binding, electron carrier activity, heme binding, oxidoreductase activity, acting on paired donors, with incorporation or reduction of molecular oxygen
groupX	6362007	6594901	G2	psR	ENSGACG00000004184	-	intracellular signal transduction, lipid metabolic process, phospholipase C activity, phosphoric diester hydrolase activity
groupX	6362007	6594901	G2	psR	ENSGACG00000004192	MRPL9	-
groupX	6362007	6594901	G2	psR	ENSGACG00000004195	PRCC	-
groupX	6362007	6594901	G2	psR	ENSGACG00000004206	MRPL24	translation, intracellular, ribosome, structural constituent of ribosome
groupX	6362007	6594901	G2	psR	ENSGACG00000022181	-	-
groupX	6362007	6594901	G2	psR	ENSGACG00000004231	TMEM79 (1 of 2)	-
groupX	6362007	6594901	G2	psR	ENSGACG00000004084	CNDP2	metabolic process, hydrolase activity
groupX	6362007	6594901	G2	psR	ENSGACG00000004168	KRIT1	regulation of transcription, DNA-dependent, cytoskeleton, sequence-specific DNA binding transcription factor activity, protein binding
groupX	6362007	6594901	G2	psR	ENSGACG00000004199	HDGFL1	-
groupX	6362007	6594901	G2	psR	ENSGACG00000004219	BCAN (2 of 2)	cell adhesion, calcium ion binding, binding, protein binding, hyaluronic acid binding
groupX	6362007	6594901	G2	psR	ENSGACG00000004227	MEF2D	skeletal muscle myosin thick filament assembly, positive regulation of transcription from RNA polymerase II promoter, transcription regulatory region sequence-specific DNA binding
groupX	6362007	6594901	G2	psR	ENSGACG00000004143	PCNT	vesicle-mediated transport, fucose metabolic process, membrane, cytoplasm, nucleotide binding, L-fucose isomerase activity
groupX	6610706	6628713	G2	psR	ENSGACG00000022304	-	-
groupX	6610706	6628713	G2	psR	ENSGACG00000022220	-	-
groupX	6644058	6682058	G2	bg	ENSGACG00000004267	-	intracellular, zinc ion binding
groupX	6644058	6682058	G2	bg	ENSGACG00000004307	HCN3 (2 of 2)	-
groupX	6644058	6682058	G2	bg	ENSGACG00000004315	-	-
groupX	6644058	6682058	G2	bg	ENSGACG00000004248	-	intracellular signal transduction, nucleus, DNA binding, zinc ion binding, protein binding
groupX	6644058	6682058	G2	bg	ENSGACG00000004271	NDUFV1	oxidation-reduction process, NADH dehydrogenase (ubiquinone) activity, oxidoreductase activity, acting on NADH or NADPH, 4 iron, 4 sulfur cluster binding, NAD binding, FMN binding
groupX	6684363	6815339	G2	n	ENSGACG00000004318	-	protein binding
groupX	6684363	6815339	G2	n	ENSGACG00000004387	ZNF687 (1 of 2)	intracellular, nucleic acid binding, zinc ion binding
groupX	6684363	6815339	G2	n	ENSGACG00000004393	AQP10 (1 of 2)	transport, transmembrane transport, water transport, membrane, integral to membrane, transporter activity
groupX	6684363	6815339	G2	n	ENSGACG00000004434	HAX1	-
groupX	6684363	6815339	G2	n	ENSGACG00000004466	C1orf43 (1 of 2)	oxidoreductase activity
groupX	6684363	6815339	G2	n	ENSGACG00000004486	PYGO2	zinc ion binding, protein binding
groupX	6684363	6815339	G2	n	ENSGACG00000004501	-	calcium ion binding
groupX	6684363	6815339	G2	n	ENSGACG00000004332	PSMD4 (2 of 2)	phosphatidylinositol metabolic process, protein binding, phosphatidylinositol phosphate kinase activity
groupX	6684363	6815339	G2	n	ENSGACG00000004446	UBAP2L	protein binding
groupX	6684363	6815339	G2	n	ENSGACG00000004468	TUFT1 (1 of 2)	-

groupX	6684363	6815339	G2	n	ENSGACG00000004475	-	vesicle-mediated transport, membrane, myosin complex, motor activity
groupX	6684363	6815339	G2	n	ENSGACG00000004491	SHE (1 of 2)	protein binding
groupX	6684363	6815339	G2	n	ENSGACG00000004506	-	-
groupX	6953048	6976053	G2	n	ENSGACG00000004537	DPM3	-
groupX	6953048	6976053	G2	n	ENSGACG00000004534	EF-1-(1-of-2)	membrane
groupX	7350525	7443525	G2	n	ENSGACG00000004817	RPRD1A	-
groupX	7350525	7443525	G2	n	ENSGACG00000021205	-	-
groupX	7350525	7443525	G2	n	ENSGACG00000022312	-	-
groupX	7350525	7443525	G2	n	ENSGACG00000004879	C11orf70	-
groupX	7350525	7443525	G2	n	ENSGACG00000004837	-	intracellular, nucleic acid binding, zinc ion binding
groupX	7350525	7443525	G2	n	ENSGACG00000004846	NFYC	retina development in camera-type eye, intracellular, DNA binding, sequence-specific DNA binding
groupX	7350525	7443525	G2	n	ENSGACG00000004872	KCNQ4	ion transport, transmembrane transport, membrane, ion channel activity
groupX	7524845	7538845	G2	-	-	-	-
groupX	7728807	7870807	G2	n	ENSGACG00000005101	TRIB1	protein phosphorylation, ATP binding, protein kinase activity, transferase activity, transferring phosphorus-containing groups, protein serine/threonine kinase activity
groupX	7728807	7870807	G2	n	ENSGACG00000005113	FAM91A1	heme binding, oxygen binding
groupX	7728807	7870807	G2	n	ENSGACG00000005128	KLHL38 (1 of 2)	protein binding
groupX	7728807	7870807	G2	n	ENSGACG00000005164	ATP6V1C1 (1 of 2)	ATP hydrolysis coupled proton transport, proton-transporting V-type ATPase, V1 domain, hydrolase activity, acting on acid anhydrides, catalyzing transmembrane movement of substances
groupX	7728807	7870807	G2	n	ENSGACG00000005193	ADNP2	regulation of transcription, DNA-dependent, intracellular, sequence-specific DNA binding transcription factor activity, zinc ion binding, sequence-specific DNA binding, protein binding
groupX	7728807	7870807	G2	n	ENSGACG00000005197	GGCT	-
groupX	7728807	7870807	G2	n	ENSGACG00000005220	ANKIB1 (2 of 2)	zinc ion binding, protein binding
groupX	7728807	7870807	G2	n	ENSGACG00000005235	-	-
groupX	7728807	7870807	G2	n	ENSGACG00000005147	SPIRE1 (2 of 2)	protein binding
groupX	7728807	7870807	G2	n	ENSGACG00000005185	AZIN1 (2 of 2)	catalytic activity
groupX	7728807	7870807	G2	n	ENSGACG00000005201	SNRPD1	heart development
groupX	9077385	9098535	G2	psR	ENSGACG00000006390	BTD	nitrogen compound metabolic process, hydrolase activity, acting on carbon-nitrogen (but not peptide) bonds, hydrolase activity, acting on carbon-nitrogen (but not peptide) bonds, in linear amides
groupX	9077385	9098535	G2	psR	ENSGACG00000006364	ANKRD28 (2 of 2)	protein binding
groupX	9077385	9098535	G2	psR	ENSGACG00000006404	SNX13	cell communication, signal transducer activity, phosphatidylinositol binding, protein binding
groupX	9254974	9298014	G2	n	ENSGACG00000006571	CASD1	-
groupX	9254974	9298014	G2	n	ENSGACG00000006592	SGCE	membrane, sarcoglycan complex, calcium ion binding
groupX	9254974	9298014	G2	n	ENSGACG00000006511	COL1A2	signal transduction, collagen, extracellular matrix structural constituent, receptor binding
groupX	15469805	15504805	G2	psL	ENSGACG00000010358	-	intracellular signal transduction, cyclic nucleotide biosynthetic process, phosphorus-oxygen lyase activity
groupX	15469805	15504805	G2	psL	ENSGACG00000010362	-	protein phosphorylation, ATP binding, protein kinase activity, transferase activity, transferring phosphorus-containing groups, protein serine/threonine kinase activity
groupXI	893197	933364	G2	psR	ENSGACG00000005143	COL1A1 (2 of 2)	signal transduction, regulation of ossification, collagen, cytoplasm, extracellular matrix structural constituent, receptor binding, protein binding, peptidase inhibitor activity
groupXI	985885	1020885	G2	psR	ENSGACG00000005261	CYB561	integral to membrane
groupXI	985885	1020885	G2	psR	ENSGACG00000005267	-	-
groupXI	985885	1020885	G2	psR	ENSGACG00000005270	-	electron carrier activity, protein binding, iron-sulfur cluster binding
groupXI	1765789	1790789	G2	n	-	-	-
groupXI	1810065	1845065	G2	psR	-	-	-
groupXI	1848389	1879389	G2	n	-	-	-
groupXI	1932570	1961573	G2	-	ENSGACG00000005654	PRAF2	-
groupXI	1932570	1961573	G2	-	ENSGACG00000022243	-	-

groupXI	1932570	1961573	G2	-	ENSGACG00000005644	NFE2L1 (2 of 2)	regulation of transcription, DNA-dependent, sequence-specific DNA binding transcription factor activity, DNA binding, sequence-specific DNA binding, protein dimerization activity
groupXI	1932570	1961573	G2	-	ENSGACG00000005661	COPZ2	transport
groupXI	5429098	5888371	G2	psR	ENSGACG00000008434	FAM134C	-
groupXI	5429098	5888371	G2	psR	ENSGACG00000008462	-	GTP catabolic process, microtubule-based process, protein polymerization, protein complex, microtubule, GTPase activity, GTP binding
groupXI	5429098	5888371	G2	psR	ENSGACG00000008483	-	cell adhesion, calcium ion binding, protein binding
groupXI	5429098	5888371	G2	psR	ENSGACG00000008501	RAMP2	intracellular protein transport, regulation of G-protein coupled receptor protein signaling pathway, integral to membrane, protein transporter activity
groupXI	5429098	5888371	G2	psR	ENSGACG00000008510	-	cation transport, pore complex assembly, hemolysis in other organism involved in symbiotic interaction, pore complex, channel activity
groupXI	5429098	5888371	G2	psR	ENSGACG00000008514	-	cation transport, pore complex assembly, hemolysis in other organism involved in symbiotic interaction, pore complex, channel activity
groupXI	5429098	5888371	G2	psR	ENSGACG00000008523	FZD2	cilium assembly, heart looping, Wnt receptor signaling pathway, calcium modulating pathway, cell migration, non-canonical Wnt receptor signaling pathway involved in heart development, heart jogging, sensory epithelium regeneration, determination of left/right symmetry, convergent extension involved in gastrulation, endocrine pancreas development, plasma membrane
groupXI	5429098	5888371	G2	psR	ENSGACG00000008527	-	protein phosphorylation, ATP binding, protein kinase activity, protein binding, transferase activity, transferring phosphorus-containing groups, protein serine/threonine kinase activity
groupXI	5429098	5888371	G2	psR	ENSGACG00000008535	HSD17B1P1	oxidation-reduction process, metabolic process, estrogen biosynthetic process, androgen metabolic process, cytoplasm, oxidoreductase activity, estradiol 17-beta-dehydrogenase activity, steroid dehydrogenase activity
groupXI	5429098	5888371	G2	psR	ENSGACG00000008544	-	protein phosphorylation, ATP binding, protein kinase activity, transferase activity, transferring phosphorus-containing groups, protein serine/threonine kinase activity
groupXI	5429098	5888371	G2	psR	ENSGACG00000008605	PTRF (2 of 2)	-
groupXI	5429098	5888371	G2	psR	ENSGACG00000008607	STAT3	regulation of transcription, DNA-dependent, signal transduction, motor axon guidance, epithelial to mesenchymal transition, establishment of planar polarity, establishment of cell polarity, positive regulation of glial cell proliferation, cell migration involved in gastrulation, nucleus, sequence-specific DNA binding transcription factor activity, signal transducer activity, protein binding
groupXI	5429098	5888371	G2	psR	ENSGACG00000008634	-	regulation of transcription, DNA-dependent, signal transduction, erythrocyte development, JAK-STAT cascade, lymphocyte differentiation, positive regulation of myeloid cell differentiation, positive regulation of transcription from RNA polymerase II promoter, erythrocyte differentiation, nucleus, sequence-specific DNA binding transcription factor activity, signal transducer activity, protein binding
groupXI	5429098	5888371	G2	psR	ENSGACG00000008659	RAB5C (2 of 2)	signal transduction, GTP catabolic process, small GTPase mediated signal transduction, intracellular protein transport, nucleocytoplasmic transport, protein transport, intracellular, GTPase activity, GTP binding
groupXI	5429098	5888371	G2	psR	ENSGACG00000008679	KAT2A	regulation of transcription, DNA-dependent, metabolic process, nucleus, protein binding, N-acetyltransferase activity, histone acetyltransferase activity
groupXI	5429098	5888371	G2	psR	ENSGACG00000008740	DHX58	nucleic acid binding, DNA binding, ATP binding, hydrolase activity, nucleotide binding, helicase activity, ATP-dependent helicase activity, nucleoside-triphosphatase activity
groupXI	5429098	5888371	G2	psR	ENSGACG00000008459	-	-
groupXI	5429098	5888371	G2	psR	ENSGACG00000008473	PLEKHH3	signal transduction, cytoskeleton, protein binding
groupXI	5429098	5888371	G2	psR	ENSGACG00000008517	C1QL1 (1 of 2)	protein binding
groupXI	5429098	5888371	G2	psR	ENSGACG00000008532	-	-
groupXI	5429098	5888371	G2	psR	ENSGACG00000008553	ATP6V0A1 (2 of 2)	regulation of transcription, DNA-dependent, ATP hydrolysis coupled proton transport, intracellular, proton-transporting two-sector ATPase complex, proton-transporting domain, sequence-specific DNA binding transcription factor activity, sequence-specific DNA binding, hydrogen ion transmembrane transporter activity
groupXI	5429098	5888371	G2	psR	ENSGACG00000008641	-	signal transduction, intracellular signal transduction, lipid metabolic process, protein binding, phospholipase C activity, phosphatidylinositol phospholipase C activity, phosphoric diester hydrolase activity
groupXI	5429098	5888371	G2	psR	ENSGACG00000008648	KCNH4 (2 of 2)	regulation of transcription, DNA-dependent, signal transduction, ion transport, transmembrane transport, two-component signal transduction system (phosphorelay), membrane, signal transducer activity, protein binding, ion channel activity, two-component sensor activity
groupXI	5429098	5888371	G2	psR	ENSGACG00000008492	EZH1	G-protein coupled receptor signaling pathway, DNA repair, intracellular, heterotrimeric G-protein complex, signal transducer activity, DNA binding, zinc ion binding, binding, protein binding, endonuclease activity

groupXI	5429098	5888371	G2	psR	ENSGACG00000008519	CCDC43	-
groupXI	6903683	6933683	G2	psR	ENSGACG00000009558	ASIC2	sodium ion transport, membrane, integral to membrane, sodium channel activity, ligand-gated sodium channel activity
groupXI	11935913	11987991	G2	n	ENSGACG00000012598	LUC7L3	-
groupXI	11935913	11987991	G2	n	ENSGACG00000012606	-	-
groupXII	2644648	2664648	G2	bg	-	-	-
groupXII	2693188	2723188	G2	psL	ENSGACG00000003711	-	cation transport, cation transmembrane transporter activity
groupXII	2817568	2834568	G2	-	ENSGACG00000003721	CPNE9 (2 of 2)	protein binding
groupXII	2836125	2906899	G2	n	ENSGACG00000003739	LHFPL4 (2 of 2)	-
groupXII	2836125	2906899	G2	n	ENSGACG00000003767	PLXNB1 (2 of 2)	signal transduction, membrane, intracellular, protein binding
groupXII	2836125	2906899	G2	n	ENSGACG00000003742	-	protein phosphorylation, ATP binding, protein kinase activity, transferase activity, transferring phosphorus-containing groups, protein serine/threonine kinase activity
groupXII	9691978	9727978	G2	psR	ENSGACG00000007367	NOC2L	binding
groupXII	9691978	9727978	G2	psR	ENSGACG00000007376	SAMD11	protein binding
groupXII	10205937	10218937	G2	psR	ENSGACG00000007935	SLC16A4	regulation of transcription, DNA-dependent, transmembrane transport, integral to membrane, intracellular, sequence-specific DNA binding transcription factor activity, sequence-specific DNA binding
groupXII	10439988	10462317	G2	n	ENSGACG00000008244	C1orf106 (1 of 2)	-
groupXII	10439988	10462317	G2	n	ENSGACG00000008262	FANCE	translation, large ribosomal subunit, RNA binding, structural constituent of ribosome
groupXII	10439988	10462317	G2	n	ENSGACG00000008285	-	nucleic acid binding, zinc ion binding, protein binding
groupXII	10439988	10462317	G2	n	ENSGACG00000008288	PPARD	regulation of transcription, DNA-dependent, steroid hormone mediated signaling pathway, nucleus, sequence-specific DNA binding transcription factor activity, steroid hormone receptor activity, zinc ion binding, sequence-specific DNA binding
groupXII	10439988	10462317	G2	n	ENSGACG00000008283	-	-
groupXII	10639119	10682119	G2	psL	ENSGACG00000008474	-	ATP binding, nucleotide binding, nucleoside-triphosphatase activity
groupXII	10639119	10682119	G2	psL	ENSGACG00000008477	STAT6	regulation of transcription, DNA-dependent, signal transduction, nucleus, sequence-specific DNA binding transcription factor activity, signal transducer activity, protein binding
groupXII	10639119	10682119	G2	psL	ENSGACG00000008484	MIP (2 of 2)	transport, transmembrane transport, water transport, lens development in camera-type eye, membrane, integral to membrane, transporter activity, water transmembrane transporter activity, water channel activity
groupXII	10639119	10682119	G2	psL	ENSGACG00000008508	PTGES3 (2 of 2)	-
groupXII	10639119	10682119	G2	psL	ENSGACG00000008546	DTX3	zinc ion binding, protein binding
groupXII	10837976	10881976	G2	n	ENSGACG00000008755	WNK3	protein phosphorylation, ATP binding, protein kinase activity, transferase activity, transferring phosphorus-containing groups, protein serine/threonine kinase activity
groupXII	10837976	10881976	G2	n	ENSGACG00000008770	PHF8	embryonic viscerocranium morphogenesis, regulation of neuron apoptotic process, brain development, zinc ion binding, protein binding
groupXII	10837976	10881976	G2	n	ENSGACG00000008789	-	cellular protein modification process, intracellular, zinc ion binding, protein binding, acid-amino acid ligase activity
groupXII	11850929	11866929	G2	n	ENSGACG00000009569	-	-
groupXII	12862040	12918797	G2	n	ENSGACG00000010294	NOL4 (2 of 2)	oxidation-reduction process, oxidoreductase activity, transition metal ion binding
groupXII	14259754	14273060	G2	bg	ENSGACG00000011034	MAGI3 (1 of 2)	protein binding
groupXII	14515907	14544907	G2	psL	ENSGACG00000011124	SALL4	embryonic pectoral fin morphogenesis, positive regulation of fibroblast growth factor receptor signaling pathway, intracellular, DNA binding, zinc ion binding
groupXII	14515907	14544907	G2	psL	ENSGACG00000011089	ATP9A	metabolic process, ATP biosynthetic process, phospholipid transport, membrane, integral to membrane, magnesium ion binding, ATP binding, catalytic activity, ATPase activity, coupled to transmembrane movement of ions, phosphorylative mechanism, phospholipid-translocating ATPase activity
groupXIII	609904	633904	G2	bg	-	-	-
groupXIII	7788557	7881677	G2	psL	-	-	-
groupXIII	9866089	9885641	G2	n	ENSGACG00000010134	F2RL2 (2 of 2)	G-protein coupled receptor signaling pathway, integral to membrane
groupXIII	9866089	9885641	G2	n	ENSGACG00000010141	-	-
groupXIII	9866089	9885641	G2	n	ENSGACG00000010145	-	-
groupXIII	9866089	9885641	G2	n	ENSGACG00000010070	-	regulation of transcription, DNA-dependent, signal transduction, small GTPase mediated signal transduction, regulation of small GTPase mediated signal transduction, intracellular, myosin complex, sequence-specific DNA binding transcription factor activity, motor activity, ATP binding, sequence-specific DNA binding, protein binding, GTPase activator activity, Ras GTPase activator activity

groupXIII	9866089	9885641	G2	n	ENSGACG00000010138	F2R	G-protein coupled receptor signaling pathway, integral to membrane
groupXIII	12350008	12393671	G2	psR	ENSGACG00000011169	DBNL (1 of 2)	intracellular, actin binding, protein binding
groupXIII	12350008	12393671	G2	psR	ENSGACG00000011172	UBE2D4	acid-amino acid ligase activity
groupXIII	12350008	12393671	G2	psR	ENSGACG00000011184	TCF7L1	generation of neurons, rostrocaudal neural tube patterning, cell proliferation in dorsal spinal cord, midbrain development, brain segmentation, hindbrain development, embryonic pattern specification, positive regulation of cell proliferation, DNA binding, protein binding
groupXIII	13906591	13932592	G2	psR	ENSGACG00000012153	ASB6	intracellular signal transduction, protein binding
groupXIII	14377287	14388994	G2	psR	-	-	-
groupXIV	12178560	12206818	G2	bg	-	-	-
groupXIV	12598601	12679907	G2	psR	ENSGACG00000018194	-	-
groupXIV	12789928	12841994	G2	bg	ENSGACG00000018217	UNC13B (1 of 2)	intracellular signal transduction, protein binding
groupXV	4791811	4821811	G2	n	ENSGACG00000007642	-	binding
groupXV	4791811	4821811	G2	n	ENSGACG00000007648	TTC8	pigment granule aggregation in cell center, heart looping, melanosome transport, Kupffer's vesicle development, convergent extension, intracellular transport, neural crest cell migration, protein binding
groupXV	4791811	4821811	G2	n	ENSGACG00000007688	EML5	protein binding
groupXV	9197761	9236761	G2	psL	ENSGACG00000010507	C15orf41	-
groupXV	9245099	9271099	G2	psL	ENSGACG00000010507	C15orf41	-
groupXV	9495758	9516076	G2	n	ENSGACG00000010542	PTPN21	protein dephosphorylation, dephosphorylation, heart morphogenesis, epithelial to mesenchymal transition, somitogenesis, cytoskeleton, protein tyrosine phosphatase activity, protein tyrosine/serine/threonine phosphatase activity, phosphatase activity
groupXV	9495758	9516076	G2	n	ENSGACG00000010552	SPATA7	-
groupXV	9495758	9516076	G2	n	ENSGACG00000010557	KCNK10	-
groupXVI	263451	295458	G2	n	ENSGACG00000001462	-	growth factor activity
groupXVI	263451	295458	G2	n	ENSGACG00000001457	TMPRSS3	proteolysis, membrane, catalytic activity, protein binding, serine-type endopeptidase activity, scavenger receptor activity
groupXVI	263451	295458	G2	n	ENSGACG00000001463	CCDC93	vesicle-mediated transport, membrane, binding
groupXVI	263451	295458	G2	n	ENSGACG00000001456	PKNOX1	regulation of transcription, DNA-dependent, sequence-specific DNA binding transcription factor activity, sequence-specific DNA binding, protein binding
groupXVI	11890194	11900194	G2	psR	ENSGACG00000006226	FAM126B	-
groupXVI	13716843	13736843	G2	bg	ENSGACG00000006889	TSC22D1 (2 of 2)	regulation of transcription, DNA-dependent, sequence-specific DNA binding transcription factor activity
groupXVI	13777089	13793089	G2	bg	ENSGACG00000006935	-	oxidation-reduction process, cell surface receptor signaling pathway, metabolic process, cell migration involved in gastrulation, membrane, plasma membrane, oxidoreductase activity, transmembrane signaling receptor activity, protein binding
groupXVI	13777089	13793089	G2	bg	ENSGACG00000006937	-	G-protein coupled receptor signaling pathway, integral to membrane
groupXVI	16845085	16860085	G2	n	-	-	-
groupXVII	4401113	4427173	G2	bg	ENSGACG00000005966	CDH22	cell adhesion, homophilic cell adhesion, membrane, plasma membrane, calcium ion binding
groupXVIII	6171569	6321391	G2	psR	ENSGACG00000007466	-	-
groupXVIII	6171569	6321391	G2	psR	ENSGACG00000007478	-	-
groupXVIII	6171569	6321391	G2	psR	ENSGACG00000007328	HADHB	metabolic process, catalytic activity, transferase activity, transferring acyl groups other than amino-acyl groups
groupXVIII	6171569	6321391	G2	psR	ENSGACG00000007459	FAM59B	protein binding
groupXVIII	6171569	6321391	G2	psR	ENSGACG00000007463	SYNE2 (2 of 2)	actin binding, protein binding
groupXVIII	6171569	6321391	G2	psR	ENSGACG00000007470	-	-
groupXVIII	6171569	6321391	G2	psR	ENSGACG00000007483	-	protein binding
groupXVIII	6357926	6428130	G2	-	ENSGACG00000007522	JAG2	multicellular organismal development, cell communication, Notch signaling pathway, pancreas development, spinal cord motor neuron differentiation, ventral spinal cord interneuron differentiation, pronephros development, liver development, membrane, integral to membrane, calcium ion binding, protein binding, peptidase inhibitor activity
groupXVIII	6357926	6428130	G2	-	ENSGACG00000007543	NUDT14	metal ion binding, hydrolase activity, hydrolase activity, acting on acid anhydrides, in phosphorus-containing anhydrides
groupXVIII	6547823	6669253	G2	psR	ENSGACG00000007661	ACTC1 (2 of 3)	embryonic heart tube development, ATP binding
groupXVIII	6547823	6669253	G2	psR	ENSGACG00000007697	ARHGAP11B	signal transduction, intracellular
groupXVIII	6547823	6669253	G2	psR	ENSGACG00000007698	-	-
groupXVIII	6547823	6669253	G2	psR	ENSGACG00000007699	SCG5	neuropeptide signaling pathway, secretory granule

groupXVIII	6547823	6669253	G2	psR	ENSGACG00000007742	FNTB	catalytic activity
groupXVIII	6547823	6669253	G2	psR	ENSGACG00000007805	-	-
groupXVIII	6547823	6669253	G2	psR	ENSGACG00000007808	MAX (1 of 2)	regulation of transcription, DNA-dependent, nucleus
groupXVIII	6547823	6669253	G2	psR	ENSGACG00000007823	SLC10A1 (1 of 2)	sodium ion transport, membrane, bile acid:sodium symporter activity
groupXVIII	6547823	6669253	G2	psR	ENSGACG00000007704	FMN1 (2 of 2)	actin cytoskeleton organization, cellular component organization, actin binding
groupXVIII	6547823	6669253	G2	psR	ENSGACG00000007722	-	regulation of transcription, DNA-dependent, signal transduction, GTP catabolic process, small GTPase mediated signal transduction, intracellular protein transport, nucleocytoplasmic transport, protein transport, intracellular, ATP binding, GTPase activity, GTP binding, transcription factor binding
groupXVIII	8240442	8268442	G2	psR	ENSGACG00000008692	C6orf211	-
groupXVIII	8240442	8268442	G2	psR	ENSGACG00000008721	-	signal transduction, keratinization, membrane, cytoplasm, signal transducer activity, actin binding, protein binding
groupXVIII	8240442	8268442	G2	psR	ENSGACG00000008711	-	regulation of transcription, DNA-dependent, steroid hormone mediated signaling pathway, response to chemical stimulus, response to estrogen stimulus, response to xenobiotic stimulus, response to estradiol stimulus, posterior lateral line neuromast primordium migration, nucleus, sequence-specific DNA binding transcription factor activity, steroid hormone receptor activity, DNA binding, zinc ion binding, steroid binding, sequence-specific DNA binding
groupXVIII	10270215	10297458	G2	bg	ENSGACG00000010336	KCNH5	regulation of transcription, DNA-dependent, signal transduction, G-protein coupled receptor signaling pathway, ion transport, transmembrane transport, two-component signal transduction system (phosphorelay), membrane, integral to membrane, signal transducer activity, protein binding, ion channel activity, two-component sensor activity
groupXVIII	10270215	10297458	G2	bg	ENSGACG00000010351	SGPP1 (2 of 2)	membrane, catalytic activity
groupXVIII	10270215	10297458	G2	bg	ENSGACG00000010354	ABCD4	transport, transmembrane transport, membrane, integral to membrane, ATP binding, nucleotide binding, ATPase activity, ATPase activity, coupled to transmembrane movement of substances, nucleoside-triphosphatase activity
groupXVIII	10270215	10297458	G2	bg	ENSGACG00000010318	C14orf149	proline racemase activity
groupXX	6478533	6495533	G2	psL	ENSGACG00000006485	DNAH5	-
groupXX	6705373	6744580	G2	n	-	-	-
groupXX	8530415	8558415	G2	-	ENSGACG00000007402	-	-
groupXX	8530415	8558415	G2	-	ENSGACG00000007407	-	-
groupXX	8530415	8558415	G2	-	ENSGACG00000007410	-	-
groupXX	8530415	8558415	G2	-	ENSGACG00000007412	-	intracellular, DNA binding, zinc ion binding
groupXX	8530415	8558415	G2	-	ENSGACG00000007401	DCBLD1	-
groupXX	9128634	9131634	G2	psL	ENSGACG00000007723	-	ion transport, membrane, ion channel activity
groupXX	9666746	9717835	G2	bg	ENSGACG00000008216	JAZF1 (1 of 2)	cilium assembly, determination of left/right symmetry, intracellular, zinc ion binding
groupXX	9666746	9717835	G2	bg	ENSGACG00000008229	HIBADH (2 of 2)	oxidation-reduction process, valine metabolic process, pentose-phosphate shunt, NAD binding, 3-hydroxyisobutyrate dehydrogenase activity, phosphogluconate dehydrogenase (decarboxylating) activity
groupXX	9666746	9717835	G2	bg	ENSGACG00000008296	HOXA13 (2 of 2)	regulation of transcription, DNA-dependent, negative regulation of transcription, DNA-dependent, nucleus, sequence-specific DNA binding transcription factor activity, sequence-specific DNA binding, protein binding, nucleic acid binding transcription factor activity
groupXX	9666746	9717835	G2	bg	ENSGACG00000008297	HOXA11 (2 of 2)	regulation of transcription, DNA-dependent, nucleus, sequence-specific DNA binding transcription factor activity, sequence-specific DNA binding, protein binding
groupXX	9666746	9717835	G2	bg	ENSGACG00000008221	TAX1BP1 (1 of 2)	-
groupXX	11592217	11627217	G2	n	ENSGACG00000009917	LPCAT3	-
groupXX	11592217	11627217	G2	n	ENSGACG00000022746	-	-
groupXX	11592217	11627217	G2	n	ENSGACG00000009950	TPI1	metabolic process, triose-phosphate isomerase activity
groupXX	11592217	11627217	G2	n	ENSGACG00000010009	-	intracellular, zinc ion binding
groupXX	11592217	11627217	G2	n	ENSGACG00000009910	CLSTN3	homophilic cell adhesion, membrane, calcium ion binding
groupXX	12573128	12584378	G2	bg	ENSGACG00000011621	KIAA0907	-
groupXX	18532025	18546043	G2	psL	-	-	-
groupXXI	7821341	7876695	G2	psR	ENSGACG00000003283	FAM162B	-
groupXXI	7821341	7876695	G2	psR	ENSGACG00000003285	KPNA1	intracellular protein transport, protein import into nucleus, nucleus, cytoplasm, nuclear pore, binding, protein binding, protein transporter activity, intracellular protein transport, protein import into nucleus, nucleus, cytoplasm, nuclear pore, binding, protein binding, protein transporter activity

groupXXI	7821341	7876695	G2	psR	ENSGACG00000003337	-	-
groupXXI	7821341	7876695	G2	psR	ENSGACG00000003341	VIPR1 (1 of 2)	G-protein coupled receptor signaling pathway, cell surface receptor signaling pathway, adenylate cyclase-modulating G-protein coupled receptor signaling pathway, membrane, integral to membrane, G-protein coupled receptor activity, transmembrane signaling receptor activity
groupI	244075	255075	G1	psL	ENSGACG00000004570	ARRB1	signal transduction, embryonic hemopoiesis, lateral mesoderm development, protein binding
groupI	4163494	4189494	G1	psR	ENSGACG00000006869	TNFRSF19	binding
groupI	4163494	4189494	G1	psR	ENSGACG00000006872	-	-
groupI	4163494	4189494	G1	psR	ENSGACG00000006874	MIPEP	proteolysis, metalloendopeptidase activity
groupI	4624180	4658180	G1	-	-	-	-
groupI	4736300	4762300	G1	n	ENSGACG00000007125	PTX3	-
groupI	4736300	4762300	G1	n	ENSGACG00000007117	VEPH1	ear morphogenesis, brain development, protein binding
groupI	4773122	4844745	G1	n	ENSGACG00000007144	GOLIM4	-
groupI	4773122	4844745	G1	n	ENSGACG00000007161	SERPINI1	-
groupI	4773122	4844745	G1	n	ENSGACG00000007171	PDCD10 (2 of 2)	blood vessel development, vasculature development, embryo development, heart development
groupI	4773122	4844745	G1	n	ENSGACG00000007177	-	protein binding
groupI	4846724	4890512	G1	n	-	-	-
groupI	10466227	10538227	G1	psR	ENSGACG00000010259	P2RY1	G-protein coupled receptor signaling pathway, integral to membrane
groupI	10466227	10538227	G1	psR	ENSGACG00000010261	-	signal transduction, GTP catabolic process, small GTPase mediated signal transduction, intracellular protein transport, nucleocytoplasmic transport, protein transport, intracellular, GTPase activity, GTP binding
groupI	10466227	10538227	G1	psR	ENSGACG00000010268	ARHGEF26	-
groupI	10466227	10538227	G1	psR	ENSGACG00000010276	DHX36	-
groupI	10559223	10769223	G1	psR	ENSGACG00000010309	-	-
groupI	10559223	10769223	G1	psR	ENSGACG00000010316	-	G-protein coupled receptor signaling pathway, integral to membrane, G-protein coupled receptor activity
groupI	10559223	10769223	G1	psR	ENSGACG00000010322	-	G-protein coupled receptor signaling pathway, integral to membrane, G-protein coupled receptor activity
groupI	10559223	10769223	G1	psR	ENSGACG00000010326	-	G-protein coupled receptor signaling pathway, integral to membrane, G-protein coupled receptor activity
groupI	10559223	10769223	G1	psR	ENSGACG00000010329	-	G-protein coupled receptor signaling pathway, integral to membrane, G-protein coupled receptor activity
groupI	10559223	10769223	G1	psR	ENSGACG00000010342	-	G-protein coupled receptor signaling pathway, integral to membrane, G-protein coupled receptor activity
groupI	10559223	10769223	G1	psR	ENSGACG00000010344	-	G-protein coupled receptor signaling pathway, integral to membrane, G-protein coupled receptor activity
groupI	10559223	10769223	G1	psR	ENSGACG00000010349	-	G-protein coupled receptor signaling pathway, integral to membrane, G-protein coupled receptor activity
groupI	10559223	10769223	G1	psR	ENSGACG00000010364	-	G-protein coupled receptor signaling pathway, integral to membrane, G-protein coupled receptor activity
groupI	10559223	10769223	G1	psR	ENSGACG00000010305	MME	proteolysis, metalloendopeptidase activity, metallopeptidase activity
groupI	10559223	10769223	G1	psR	ENSGACG00000010323	-	G-protein coupled receptor signaling pathway, integral to membrane, G-protein coupled receptor activity
groupI	10559223	10769223	G1	psR	ENSGACG00000010337	-	G-protein coupled receptor signaling pathway, integral to membrane, G-protein coupled receptor activity
groupI	10559223	10769223	G1	psR	ENSGACG00000010313	-	G-protein coupled receptor signaling pathway, integral to membrane, G-protein coupled receptor activity
groupI	10817455	10838466	G1	psR	ENSGACG00000010386	C3orf33	nucleic acid binding, hydrolase activity, acting on ester bonds
groupI	10817455	10838466	G1	psR	ENSGACG00000010389	MED13 (2 of 2)	regulation of transcription from RNA polymerase II promoter, mediator complex
groupI	11597262	11647262	G1	psR	ENSGACG00000010833	TAOK1 (2 of 2)	protein phosphorylation, ATP binding, protein kinase activity, transferase activity, transferring phosphorus-containing groups, protein serine/threonine kinase activity
groupI	11597262	11647262	G1	psR	ENSGACG00000010843	TSR1	ribosome biogenesis, nucleus
groupI	11597262	11647262	G1	psR	ENSGACG00000010848	HIC1	potassium ion transport, membrane, intracellular, voltage-gated potassium channel complex, zinc ion binding, protein binding, voltage-gated potassium channel activity, DNA binding
groupI	11597262	11647262	G1	psR	ENSGACG00000010818	PAF1	Cdc73/Paf1 complex
groupI	17523164	17714357	G1	n	ENSGACG00000013412	-	transposition, DNA-mediated, DNA binding, transposase activity
groupI	18889918	18920519	G1	bg	-	-	-
groupI	21515129	21551132	G1	bg	ENSGACG00000014268	TNS1 (2 of 2)	ATP binding, protein binding
groupI	21834818	21990705	G1	psL	ENSGACG00000014349	CCDC28A	-
groupI	21834818	21990705	G1	psL	ENSGACG00000014353	ECT2L	protein binding
groupI	21834818	21990705	G1	psL	ENSGACG00000014367	-	-

groupI	21834818	21990705	G1	psL	ENSGACG00000014371	-	-
groupI	21834818	21990705	G1	psL	ENSGACG00000014373	COL8A1 (2 of 2)	protein binding
groupI	21834818	21990705	G1	psL	ENSGACG00000014376	JAGN1 (2 of 3)	-
groupI	21834818	21990705	G1	psL	ENSGACG00000014378	-	-
groupI	21834818	21990705	G1	psL	ENSGACG00000014396	TGDS	cellular metabolic process, biosynthetic process, steroid biosynthetic process, extracellular polysaccharide biosynthetic process, catalytic activity, coenzyme binding, 3-beta-hydroxy-delta5-steroid dehydrogenase activity, dTDP-4-dehydrorhamnose reductase activity
groupI	21834818	21990705	G1	psL	ENSGACG00000014388	GPR180	mitochondrial respiratory chain, electron carrier activity, cytochrome-c oxidase activity
groupI	21834818	21990705	G1	psL	ENSGACG00000014407	-	nucleic acid binding, ATP binding, helicase activity, ATP-dependent helicase activity
groupI	21834818	21990705	G1	psL	ENSGACG00000014358	-	-
groupI	22066245	22094344	G1	-	ENSGACG00000014437	SATB2 (2 of 2)	regulation of transcription, DNA-dependent, sequence-specific DNA binding transcription factor activity, DNA binding, sequence-specific DNA binding, protein binding
groupI	22066245	22094344	G1	-	ENSGACG00000014436	-	signal transduction, signal transducer activity
groupI	22733666	22761666	G1	n	ENSGACG00000014770	MAP2 (2 of 2)	negative regulation of microtubule depolymerization
groupI	22993627	23008716	G1	-	ENSGACG00000014850	-	DNA binding, protein binding
groupI	24213196	24272852	G1	psL	ENSGACG00000015027	SLITRK5 (1 of 2)	protein binding
groupI	24295693	24348146	G1	psL	-	-	-
groupII	128991	151991	G1	n	ENSGACG00000013938	FAM108C1	hydrolase activity
groupII	4467143	4482464	G1	n	-	-	-
groupII	4555642	4581642	G1	psR	ENSGACG00000014734	AP3S2	transport, intracellular protein transport, vesicle-mediated transport, membrane coat
groupII	4555642	4581642	G1	psR	ENSGACG00000014729	IGF1R (1 of 2)	protein phosphorylation, transmembrane receptor protein tyrosine kinase signaling pathway, embryonic camera-type eye development, germ cell migration, negative regulation of cysteine-type endopeptidase activity involved in apoptotic process, heart morphogenesis, spinal cord motor neuron differentiation, camera-type eye development, fin regeneration, semicircular canal morphogenesis, embryo development, heart development, insulin-like growth factor receptor signaling pathway, membrane, membrane fraction, ATP binding, protein kinase activity, protein binding, transferase activity, transferring phosphorus-containing groups, protein serine/threonine kinase activity, transmembrane receptor protein tyrosine kinase activity, protein tyrosine kinase activity, cysteine-type endopeptidase inhibitor activity involved in apoptotic process, insulin-like growth factor binding
groupII	10378581	10411993	G1	n	ENSGACG00000015714	-	signal transduction
groupII	10378581	10411993	G1	n	ENSGACG00000015715	TEAD1 (1 of 2)	regulation of transcription, DNA-dependent, nucleus, sequence-specific DNA binding transcription factor activity, protein binding, regulation of transcription, DNA-dependent, nucleus, sequence-specific DNA binding transcription factor activity, protein binding, regulation of transcription, DNA-dependent, nucleus, sequence-specific DNA binding transcription factor activity, protein binding, regulation of transcription, DNA-dependent, nucleus, sequence-specific DNA binding transcription factor activity, protein binding
groupII	10953879	10975048	G1	psL	-	-	-
groupII	12206310	12220310	G1	psL	-	-	-
groupII	12873820	12899820	G1	psR	ENSGACG00000016032	ELP4	regulation of transcription from RNA polymerase II promoter, Elongator holoenzyme complex
groupII	13605684	13659144	G1	n	ENSGACG00000016144	PDCD2	cytoplasm, zinc ion binding
groupII	13605684	13659144	G1	n	ENSGACG00000016147	TBP	regulation of transcription, DNA-dependent, transcription initiation from RNA polymerase II promoter, gastrulation, mRNA catabolic process, transcription from RNA polymerase II promoter, DNA binding
groupII	13605684	13659144	G1	n	ENSGACG00000016151	PSMB1	proteolysis involved in cellular protein catabolic process, proteasome core complex, endopeptidase activity, threonine-type endopeptidase activity
groupII	13605684	13659144	G1	n	ENSGACG00000016158	PHF10	zinc ion binding, protein binding
groupII	13605684	13659144	G1	n	ENSGACG00000016166	CCSAP (1 of 2)	-
groupII	13605684	13659144	G1	n	ENSGACG00000016168	ACTC1 (3 of 3)	ATP binding
groupII	13605684	13659144	G1	n	ENSGACG00000016174	-	-
groupII	13605684	13659144	G1	n	ENSGACG00000016176	ABCB10	transport, transmembrane transport, integral to membrane, ATP binding, nucleotide binding, ATPase activity, ATPase activity, coupled to transmembrane movement of substances, nucleoside-triphosphatase activity
groupII	13605684	13659144	G1	n	ENSGACG00000016150	PSMC3	protein catabolic process, cytoplasm, ATP binding, hydrolase activity, nucleotide binding, nucleoside-triphosphatase activity, protein catabolic process, cytoplasm, ATP binding, hydrolase activity, nucleotide binding, nucleoside-triphosphatase activity

groupII	13605684	13659144	G1	n	ENSGACG00000016179	URB2	-
groupII	13605684	13659144	G1	n	ENSGACG00000016164	RAB4A	signal transduction, GTP catabolic process, small GTPase mediated signal transduction, intracellular protein transport, nucleocytoplasmic transport, protein transport, intracellular, GTPase activity, GTP binding
groupII	15718790	15774714	G1	psR	-	-	-
groupII	15776058	15813058	G1	psR	ENSGACG00000016527	UNC13C	intracellular signal transduction, protein binding
groupII	15813680	15907422	G1	psR	ENSGACG00000016534	IGDCC4	protein binding
groupII	15813680	15907422	G1	psR	ENSGACG00000016527	UNC13C	intracellular signal transduction, protein binding
groupII	15988433	16031218	G1	n	-	-	-
groupII	16582378	16602378	G1	n	-	-	-
groupII	17020603	17038118	G1	psR	-	-	-
groupII	17504888	17535302	G1	psR	-	-	-
groupII	22858549	22873642	G1	psL	ENSGACG00000017613	PPFIA1	nucleotide binding, protein binding
groupIII	1247036	1285242	G1	psL	ENSGACG00000013375	-	regulation of transcription, DNA-dependent, intracellular signal transduction, phosphorylation, metabolic process, nucleus, DNA binding, zinc ion binding, kinase activity, protein binding, phosphotransferase activity, carboxyl group as acceptor
groupIII	10209637	10288125	G1	psR	ENSGACG00000022152	-	-
groupIII	10209637	10288125	G1	psR	ENSGACG00000022153	-	-
groupIII	10209637	10288125	G1	psR	ENSGACG00000016392	WAC (2 of 2)	protein binding
groupIII	10209637	10288125	G1	psR	ENSGACG00000016391	-	G-protein coupled receptor signaling pathway, visual perception, integral to membrane, G-protein coupled receptor signaling pathway, visual perception, integral to membrane
groupIII	10209637	10288125	G1	psR	ENSGACG00000016393	MPP7 (2 of 2)	protein binding
groupIII	10298112	10326112	G1	-	ENSGACG00000016395	RAB18	regulation of transcription, DNA-dependent, signal transduction, GTP catabolic process, small GTPase mediated signal transduction, intracellular protein transport, nucleocytoplasmic transport, protein transport, intracellular, ATP binding, GTPase activity, GTP binding, transcription factor binding
groupIII	10298112	10326112	G1	-	ENSGACG00000016401	ACBD5 (2 of 2)	fatty-acyl-CoA binding
groupIII	10298112	10326112	G1	-	ENSGACG00000016393	MPP7 (2 of 2)	protein binding
groupIII	10298112	10326112	G1	-	ENSGACG00000016397	YME1L1	proteolysis, protein catabolic process, membrane, ATP binding, metalloendopeptidase activity, nucleotide binding, nucleoside-triphosphatase activity
groupIII	10423311	10498345	G1	psR	ENSGACG00000016454	DUSP22 (1 of 2)	protein dephosphorylation, dephosphorylation, protein tyrosine/serine/threonine phosphatase activity, phosphatase activity, protein dephosphorylation, dephosphorylation, protein tyrosine/serine/threonine phosphatase activity, phosphatase activity
groupIII	10423311	10498345	G1	psR	ENSGACG00000016461	IRF4 (1 of 2)	regulation of transcription, DNA-dependent, proteolysis, sequence-specific DNA binding transcription factor activity, zinc ion binding, protein binding, metallocarboxypeptidase activity
groupIII	10423311	10498345	G1	psR	ENSGACG00000016466	-	regulation of transcription, DNA-dependent, sequence-specific DNA binding transcription factor activity, sequence-specific DNA binding
groupIII	10423311	10498345	G1	psR	ENSGACG00000016435	DIS3L2	RNA binding, ribonuclease activity
groupIII	10423311	10498345	G1	psR	ENSGACG00000016439	-	myosin complex, cytoskeleton, motor activity, ATP binding, protein binding
groupIII	10423311	10498345	G1	psR	ENSGACG00000016450	TLK1 (1 of 2)	protein phosphorylation, ATP binding, protein kinase activity, transferase activity, transferring phosphorus-containing groups, protein serine/threonine kinase activity
groupIII	13969743	14046770	G1	psL	ENSGACG00000017433	WNT3A	multicellular organismal development, Wnt receptor signaling pathway, calcium modulating pathway, mesoderm development, tail morphogenesis, convergent extension, midbrain-hindbrain boundary development, anterior/posterior pattern specification, embryonic pattern specification, notochord development, dorsal/ventral pattern formation, somitogenesis, extracellular region
groupIII	13969743	14046770	G1	psL	ENSGACG00000017437	-	small GTPase mediated signal transduction, intracellular protein transport, protein transport, intracellular, GTP binding
groupIII	13969743	14046770	G1	psL	ENSGACG00000017446	GUK1 (1 of 2)	nucleotide binding, protein binding, nucleoside-triphosphatase activity
groupIII	13969743	14046770	G1	psL	ENSGACG00000017431	WNT9A	multicellular organismal development, Wnt receptor signaling pathway, calcium modulating pathway, extracellular region
groupIII	13969743	14046770	G1	psL	ENSGACG00000017444	C1orf35	-
groupIII	14049659	14059659	G1	psL	ENSGACG00000017451	-	regulation of Rho protein signal transduction, intracellular, protein binding, Rho guanyl-nucleotide exchange factor activity
groupIII	15994292	16037292	G1	psL	ENSGACG00000017792	-	zinc ion binding, protein binding
groupIII	15994292	16037292	G1	psL	ENSGACG00000017799	METTL17	-
groupIII	15994292	16037292	G1	psL	ENSGACG00000017811	SDR39U1	cellular metabolic process, catalytic activity, coenzyme binding

groupIII	15994292	16037292	G1	psL	ENSGACG00000022748	-	-
groupIII	15994292	16037292	G1	psL	ENSGACG00000017812	-	G-protein coupled receptor signaling pathway, integral to membrane
groupIII	15994292	16037292	G1	psL	ENSGACG00000017794	PARP2	protein ADP-ribosylation, NAD+ ADP-ribosyltransferase activity
groupIV	4684075	4718076	G1	psR	ENSGACG00000016859	-	-
groupIV	4684075	4718076	G1	psR	ENSGACG00000016856	SLC25A48	-
groupIV	5049876	5067960	G1	-	ENSGACG00000016919	-	-
groupIV	5049876	5067960	G1	-	ENSGACG00000016915	CCDC88B	-
groupIV	6349406	6380507	G1	n	ENSGACG00000017312	CENPI	-
groupIV	6349406	6380507	G1	n	ENSGACG00000017316	TMEM35	-
groupIV	6349406	6380507	G1	n	ENSGACG00000017318	ARL13A	small GTPase mediated signal transduction, intracellular protein transport, intracellular, GTP binding
groupIV	6382530	6415530	G1	psR	ENSGACG00000017320	XKR3	-
groupIV	6382530	6415530	G1	psR	ENSGACG00000017326	-	nucleic acid binding
groupIV	6382530	6415530	G1	psR	ENSGACG00000017324	NOX1	oxidation-reduction process, integral to membrane, iron ion binding, electron carrier activity, oxidoreductase activity, flavin adenine dinucleotide binding
groupIV	6533013	6584013	G1	psL	-	-	-
groupIV	6790495	6926506	G1	psL	ENSGACG00000017467	PCDH19	cell adhesion, homophilic cell adhesion, neural tube formation, brain morphogenesis, membrane, plasma membrane, calcium ion binding
groupIV	7036370	7111370	G1	psL	-	-	-
groupIV	7132127	7215851	G1	psL	-	-	-
groupIV	7240201	7332718	G1	psL	-	-	-
groupIV	7965141	8026141	G1	psL	ENSGACG00000017530	BEND4	-
groupIV	7965141	8026141	G1	psL	ENSGACG00000017532	ATP8A1	ATP biosynthetic process, phospholipid transport, membrane, integral to membrane, magnesium ion binding, ATP binding, metal ion binding, nucleotide binding, ATPase activity, coupled to transmembrane movement of ions, phosphorylative mechanism, phospholipid-translocating ATPase activity, metabolic process, ATP biosynthetic process, phospholipid transport, membrane, integral to membrane, magnesium ion binding, ATP binding, metal ion binding, catalytic activity, nucleotide binding, ATPase activity, coupled to transmembrane movement of ions, phosphorylative mechanism, phospholipid-translocating ATPase activity
groupIV	11923526	11974526	G1	n	ENSGACG00000018213	-	protein binding
groupIV	11923526	11974526	G1	n	ENSGACG00000018221	FGF16	pectoral fin morphogenesis, cell proliferation, growth factor activity
groupIV	11923526	11974526	G1	n	ENSGACG00000018218	ATRX	nucleic acid binding, DNA binding, zinc ion binding, ATP binding, protein binding, helicase activity
groupIV	11923526	11974526	G1	n	ENSGACG00000018214	KIF3A	microtubule-based movement, ATP binding, microtubule motor activity
groupIV	13323370	13348370	G1	bg	ENSGACG00000018357	RMND5B	protein binding, protein binding, protein binding
groupIV	13323370	13348370	G1	bg	ENSGACG00000018359	N4BP3	-
groupIV	14849033	14898784	G1	psR	ENSGACG00000018493	ODZ2	signal transduction, integral to membrane, calcium ion binding, protein binding, 2 iron, 2 sulfur cluster binding
groupIV	15334868	15370269	G1	n	-	-	-
groupIV	19462375	19613598	G1	n	ENSGACG00000018926	LRRN3	protein binding
groupIV	19462375	19613598	G1	n	ENSGACG00000018927	-	-
groupIV	29778012	29792502	G1	psL	ENSGACG00000019748	-	neurotransmitter transport, integral to membrane, neurotransmitter:sodium symporter activity
groupIV	29778012	29792502	G1	psL	ENSGACG00000019755	PARP11	zinc ion binding, NAD+ ADP-ribosyltransferase activity, NAD+ ADP-ribosyltransferase activity
groupIX	8897892	8922393	G1	psR	ENSGACG00000017906	BDH2	metabolic process, heme metabolic process, oxidoreductase activity
groupIX	8897892	8922393	G1	psR	ENSGACG00000017930	CISD2	intracellular membrane-bounded organelle, 2 iron, 2 sulfur cluster binding
groupIX	8897892	8922393	G1	psR	ENSGACG00000017922	-	transmembrane transport, cation transport, integral to membrane, solute:hydrogen antiporter activity
groupIX	9205568	9242568	G1	bg	ENSGACG00000017992	LRRTM1	lipid catabolic process, phospholipid metabolic process, protein binding, phospholipase A2 activity, identical protein binding
groupIX	9205568	9242568	G1	bg	ENSGACG00000017985	CTNNA2	cell adhesion, actin cytoskeleton, structural molecule activity
groupIX	9244744	9275749	G1	bg	ENSGACG00000017985	CTNNA2	cell adhesion, actin cytoskeleton, structural molecule activity
groupIX	9587254	9620254	G1	psL	ENSGACG00000018024	UGT8	metabolic process, transferase activity, transferring hexosyl groups
groupIX	10156078	10176078	G1	bg	ENSGACG00000018157	-	-
groupIX	10156078	10176078	G1	bg	ENSGACG00000018158	-	-
groupIX	10156078	10176078	G1	bg	ENSGACG00000018155	SCRN2	proteolysis, dipeptidase activity

groupIX	10156078	10176078	G1	bg	ENSGACG00000018160	PLA2G12B (1 of 2)	lipid catabolic process, extracellular region, calcium ion binding, phospholipase A2 activity
groupIX	11669840	11704840	G1	psL	ENSGACG00000018409	PCDH10 (2 of 2)	cell adhesion, homophilic cell adhesion, membrane, plasma membrane, calcium ion binding
groupIX	15069624	15100057	G1	bg	ENSGACG00000018992	SLC25A41	calcium ion binding, protein binding, calcium ion binding, protein binding
groupIX	15069624	15100057	G1	bg	ENSGACG00000018994	C19orf53	-
groupIX	15069624	15100057	G1	bg	ENSGACG00000018995	MRI1	cellular metabolic process, cellular biosynthetic process
groupIX	15069624	15100057	G1	bg	ENSGACG00000018993	-	signal transducer activity
groupIX	15069624	15100057	G1	bg	ENSGACG00000018990	KHSRP (2 of 2)	RNA binding
groupIX	15978682	15990982	G1	psR	-	-	-
groupIX	16262740	16281175	G1	psR	-	-	-
groupIX	16367732	16400245	G1	bg	ENSGACG00000019296	SLC25A38 (1 of 2)	-
groupIX	16367732	16400245	G1	bg	ENSGACG00000019298	MYH9 (2 of 2)	signal transduction, intracellular, myosin complex, motor activity, ATP binding, nucleotide binding, protein binding
groupIX	17088031	17147293	G1	n	ENSGACG00000019394	HN1 (1 of 2)	-
groupIX	17088031	17147293	G1	n	ENSGACG00000019395	-	protein binding
groupIX	17088031	17147293	G1	n	ENSGACG00000019397	SEPT9 (1 of 2)	cell cycle, GTP binding, cell cycle, GTP binding
groupIX	20142746	20249479	G1	psR	ENSGACG00000020000	-	G-protein coupled receptor signaling pathway, integral to membrane
groupIX	20142746	20249479	G1	psR	ENSGACG00000020004	EIF4A3	nucleic acid binding, ATP binding, helicase activity, ATP-dependent helicase activity, nucleic acid binding, ATP binding, helicase activity, ATP-dependent helicase activity
groupIX	20142746	20249479	G1	psR	ENSGACG00000020011	-	-
groupIX	20142746	20249479	G1	psR	ENSGACG00000020013	-	-
groupIX	20142746	20249479	G1	psR	ENSGACG00000020020	RTN3 (2 of 2)	endoplasmic reticulum
groupIX	20142746	20249479	G1	psR	ENSGACG00000019998	-	protein binding
groupIX	20142746	20249479	G1	psR	ENSGACG00000020002	SRF (2 of 2)	regulation of transcription, DNA-dependent, transport, transmembrane transport, nucleus, membrane, sequence-specific DNA binding transcription factor activity, sequence-specific DNA binding, transporter activity
groupIX	20142746	20249479	G1	psR	ENSGACG00000020010	TMEM178	-
groupIX	20142746	20249479	G1	psR	ENSGACG00000020014	SOS1	small GTPase mediated signal transduction, nucleosome assembly, regulation of Rho protein signal transduction, regulation of small GTPase mediated signal transduction, nucleus, intracellular, nucleosome, DNA binding, protein binding, Rho guanyl-nucleotide exchange factor activity, guanyl-nucleotide exchange factor activity
groupIX	20142746	20249479	G1	psR	ENSGACG00000020021	SLC29A1 (2 of 2)	transport, membrane, nucleoside transmembrane transporter activity
groupV	170324	205428	G1	psL	ENSGACG00000002142	VCL (1 of 2)	oxidation-reduction process, metabolic process, cell adhesion, actin cytoskeleton, oxidoreductase activity, structural molecule activity, oxidation-reduction process, metabolic process, cell adhesion, actin cytoskeleton, oxidoreductase activity, structural molecule activity
groupV	170324	205428	G1	psL	ENSGACG00000002159	ADK (1 of 2)	phosphotransferase activity, alcohol group as acceptor
groupVI	8963799	9006874	G1	bg	ENSGACG00000007680	-	intracellular, nucleic acid binding, zinc ion binding
groupVII	4987977	5022432	G1	n	ENSGACG00000019483	-	-
groupVII	11953874	11981874	G1	bg	ENSGACG00000020129	-	protein binding, phosphotransferase activity, alcohol group as acceptor
groupVII	14113228	14222384	G1	bg	ENSGACG00000020215	RPS6KB1 (2 of 2)	protein phosphorylation, ATP binding, protein kinase activity, transferase activity, transferring phosphorus-containing groups, protein serine/threonine kinase activity
groupVII	14113228	14222384	G1	bg	ENSGACG00000021252	-	-
groupVII	14113228	14222384	G1	bg	ENSGACG00000020216	CLTC	intracellular protein transport, vesicle-mediated transport, clathrin coat of trans-Golgi network vesicle, clathrin coat of coated pit, binding, structural molecule activity
groupVII	15294113	15334264	G1	psR	ENSGACG00000020271	-	-
groupVII	15439142	15479767	G1	-	ENSGACG00000020278	FRMD8	cytoskeleton, cytoskeleton
groupVII	15439142	15479767	G1	-	ENSGACG00000020280	TM7SF2	membrane
groupVII	15439142	15479767	G1	-	ENSGACG00000020281	CNIH2	intracellular signal transduction, membrane
groupVII	15439142	15479767	G1	-	ENSGACG00000020279	C11orf2	lipid digestion, regulation of phospholipase activity, Golgi organization, Golgi vesicle transport, ADP-ribosylation factor binding
groupVII	16682312	16715040	G1	n	ENSGACG00000020313	PCDH1 (2 of 2)	cell adhesion, homophilic cell adhesion, membrane, plasma membrane, calcium ion binding
groupVII	17976959	18007943	G1	psL	ENSGACG00000020399	-	protein binding, insulin receptor binding
groupVII	17976959	18007943	G1	psL	ENSGACG00000020400	SLC25A2	-
groupVII	17976959	18007943	G1	psL	ENSGACG00000020401	-	integral to membrane, integral to membrane, integral to membrane

groupVII	17976959	18007943	G1	psL	ENSGACG00000020402	MPDU1 (2 of 2)	-
groupVII	17976959	18007943	G1	psL	ENSGACG00000020403	-	-
groupVII	18179385	18201385	G1	n	ENSGACG00000020415	-	protein binding
groupVII	18616006	18658006	G1	n	ENSGACG00000020442	CD248 (2 of 2)	binding
groupVII	18616006	18658006	G1	n	ENSGACG00000020443	RCE1 (2 of 2)	membrane
groupVII	18616006	18658006	G1	n	ENSGACG00000020441	DPP3	proteolysis, cytoplasm, dipeptidyl-peptidase activity
groupVII	18616006	18658006	G1	n	ENSGACG00000020444	EHBP1L1 (2 of 2)	protein binding,
groupVII	18698437	18721437	G1	psL	ENSGACG00000020450	-	growth factor activity
groupVII	18698437	18721437	G1	psL	ENSGACG00000020451	AFAP1L1 (2 of 2)	protein binding
groupVII	20176019	20199325	G1	n	ENSGACG00000020560	DCLK1 (1 of 2)	intracellular signal transduction, protein phosphorylation, ATP binding, protein kinase activity, transferase activity, transferring phosphorus-containing groups, protein serine/threonine kinase activity, intracellular signal transduction
groupVII	21105246	21151246	G1	n	-	-	-
groupVII	24399560	24475560	G1	n	ENSGACG00000020773	NCAM1 (1 of 2)	protein binding
groupVIII	11988622	12024622	G1	psR	ENSGACG00000009483	NEGR1	protein binding, identical protein binding
groupX	6160301	6172301	G1	n	-	-	-
groupX	6517945	6547101	G1	n	ENSGACG00000004206	MRPL24	translation, intracellular, ribosome, structural constituent of ribosome
groupX	6517945	6547101	G1	n	ENSGACG00000004219	BCAN (2 of 2)	cell adhesion, calcium ion binding, binding, protein binding, hyaluronic acid binding
groupX	8434880	8457880	G1	n	ENSGACG00000005894	-	-
groupX	8867907	8913211	G1	psR	ENSGACG00000006256	GNGT1	G-protein coupled receptor signaling pathway, heterotrimeric G-protein complex, signal transducer activity
groupX	8867907	8913211	G1	psR	ENSGACG00000022270	-	-
groupX	8867907	8913211	G1	psR	ENSGACG00000006126	-	oxidation-reduction process, metabolic process, oxidoreductase activity, acting on the CH-CH group of donors, acyl-CoA dehydrogenase activity
groupX	8867907	8913211	G1	psR	ENSGACG00000006264	TFPI2	serine-type endopeptidase inhibitor activity, serine-type endopeptidase inhibitor activity
groupX	8867907	8913211	G1	psR	ENSGACG00000006273	CALCR	G-protein coupled receptor signaling pathway, cell surface receptor signaling pathway, methylation, membrane, integral to membrane, G-protein coupled receptor activity, nucleic acid binding, transmembrane signaling receptor activity, methyltransferase activity
groupX	9542057	9560057	G1	n	ENSGACG00000006878	TRA2A	nucleic acid binding, nucleic acid binding,
groupX	9542057	9560057	G1	n	ENSGACG00000006894	IGF2BP3	nucleic acid binding, RNA binding
groupX	9719209	9768476	G1	n	ENSGACG00000007010	OXNAD1	oxidation-reduction process, oxidoreductase activity
groupX	9719209	9768476	G1	n	ENSGACG00000007024	-	-
groupX	9719209	9768476	G1	n	ENSGACG00000007031	FKBP14 (1 of 2)	protein folding, calcium ion binding
groupX	9719209	9768476	G1	n	ENSGACG00000007049	COLQ (2 of 2)	-
groupX	9719209	9768476	G1	n	ENSGACG00000007058	CYCS (2 of 2)	iron ion binding, electron carrier activity, heme binding
groupX	9719209	9768476	G1	n	ENSGACG00000022499	-	-
groupX	9719209	9768476	G1	n	ENSGACG00000007025	WIPF3	protein binding
groupX	9946542	9963803	G1	n	ENSGACG00000007155	HIBADH (1 of 2)	oxidation-reduction process, valine metabolic process, pentose-phosphate shunt, NAD binding, oxidoreductase activity, acting on the CH-OH group of donors, NAD or NADP as acceptor, 3-hydroxyisobutyrate dehydrogenase activity, cofactor binding, phosphogluconate dehydrogenase (decarboxylating) activity
groupX	10089566	10146761	G1	n	ENSGACG00000007236	TRAPPC3	-
groupX	10089566	10146761	G1	n	ENSGACG00000007231	SMAP2	regulation of ARF GTPase activity, zinc ion binding, ARF GTPase activator activity
groupX	10688016	10750697	G1	n	ENSGACG00000007359	-	signal transduction, extracellular space, receptor binding
groupXI	7704387	7722034	G1	psL	ENSGACG00000009943	-	metabolic process, oxidoreductase activity
groupXI	7704387	7722034	G1	psL	ENSGACG00000009926	NOTCH3	multicellular organismal development, cell differentiation, regulation of developmental process, Notch signaling pathway, negative regulation of neurogenesis, neural plate development, somatic stem cell maintenance, peripheral nervous system neuron axonogenesis, regulation of receptor-mediated endocytosis, rhombomere boundary formation, pronephros development, liver development, nucleus, membrane, integral to membrane, calcium ion binding, protein binding, histone-lysine N-methyltransferase activity
groupXI	8287107	8301187	G1	bg	ENSGACG00000022150	-	-

groupXI	8287107	8301187	G1	bg	ENSGACG00000010535	GCGR (1 of 2)	G-protein coupled receptor signaling pathway, cell surface receptor signaling pathway, membrane, integral to membrane, G-protein coupled receptor activity, transmembrane signaling receptor activity
groupXII	1073247	1129432	G1	psR	ENSGACG00000002965	ZFYVE20	intracellular, zinc ion binding, metal ion binding
groupXII	1073247	1129432	G1	psR	ENSGACG00000002980	TRH	hormone-mediated signaling pathway, extracellular region, neuropeptide hormone activity
groupXII	1073247	1129432	G1	psR	ENSGACG00000002982	TMCC1 (1 of 2)	-
groupXII	2565585	2609036	G1	psR	ENSGACG00000003654	C20orf151	-
groupXII	2565585	2609036	G1	psR	ENSGACG00000003657	-	-
groupXII	2565585	2609036	G1	psR	ENSGACG00000003658	HCK	protein phosphorylation, ATP binding, protein kinase activity, protein binding, transferase activity, transferring phosphorus-containing groups, protein serine/threonine kinase activity, protein tyrosine kinase activity
groupXII	2886781	2912295	G1	bg	ENSGACG00000003767	PLXNB1 (2 of 2)	signal transduction, membrane, intracellular, protein binding
groupXII	2886781	2912295	G1	bg	ENSGACG00000003742	-	protein phosphorylation, ATP binding, protein kinase activity, transferase activity, transferring phosphorus-containing groups, protein serine/threonine kinase activity
groupXII	3336732	3371908	G1	psR	-	-	-
groupXII	3576720	3613792	G1	psR	ENSGACG00000004187	-	transposition, DNA-mediated, nucleic acid binding, DNA binding, transposase activity
groupXII	3576720	3613792	G1	psR	ENSGACG00000004167	LAMB2	signal transduction, basement membrane assembly, muscle attachment, membrane, signal transducer activity, calcium ion binding, nucleotide binding, protein binding
groupXII	5077932	5096005	G1	n	ENSGACG00000004801	CHST13 (2 of 2)	mismatch repair, integral to membrane, ATP binding, mismatched DNA binding, sulfotransferase activity
groupXII	13177559	13223559	G1	psR	ENSGACG00000010477	NPBWR2	G-protein coupled receptor signaling pathway, integral to membrane
groupXII	13253104	13341703	G1	n	ENSGACG00000010484	FAM187A	-
groupXII	13253104	13341703	G1	n	ENSGACG00000010479	OPRL1	G-protein coupled receptor signaling pathway, integral to membrane, integral to cell outer membrane, neuropeptide binding
groupXII	13363694	13418887	G1	psR	ENSGACG00000010505	SOX18	DNA binding, protein binding
groupXII	13363694	13418887	G1	psR	ENSGACG00000010506	XKR7 (1 of 2)	-
groupXII	14246115	14252116	G1	n	ENSGACG00000011025	-	-
groupXII	14246115	14252116	G1	n	ENSGACG00000011031	RSBN1	-
groupXII	14508108	14523108	G1	n	ENSGACG00000011089	ATP9A	metabolic process, ATP biosynthetic process, phospholipid transport, membrane, integral to membrane, magnesium ion binding, ATP binding, catalytic activity, ATPase activity, coupled to transmembrane movement of ions, phosphorylative mechanism, phospholipid-translocating ATPase activity
groupXII	15564258	15599508	G1	-	ENSGACG00000011807	TMEM189	acid-amino acid ligase activity
groupXII	15564258	15599508	G1	-	ENSGACG00000011827	CEBPB	regulation of transcription, DNA-dependent, response to bacterium, sequence-specific DNA binding transcription factor activity, DNA binding, sequence-specific DNA binding, protein dimerization activity
groupXII	15564258	15599508	G1	-	ENSGACG00000011805	SLC52A3	-
groupXIII	0	139273	G1	psL	ENSGACG00000003228	STXBP1 (2 of 2)	vesicle-mediated transport, vesicle docking involved in exocytosis
groupXIII	0	139273	G1	psL	ENSGACG00000003297	DFNB31 (2 of 2)	protein binding
groupXIII	0	139273	G1	psL	ENSGACG00000003309	NOXA1	protein binding
groupXIII	0	139273	G1	psL	ENSGACG00000003326	-	membrane, calcium ion binding, mannosyl-oligosaccharide 1, 2-alpha-mannosidase activity
groupXIII	0	139273	G1	psL	ENSGACG00000003313	DPP7	proteolysis, serine-type peptidase activity
groupXIII	197322	267806	G1	psL	ENSGACG00000003335	-	ion transport, transmembrane transport, membrane, ion channel activity
groupXIII	197322	267806	G1	psL	ENSGACG00000003353	EHMT1 (2 of 2)	histone lysine methylation, nucleus, zinc ion binding, protein binding, histone-lysine N-methyltransferase activity
groupXIII	1458354	1552216	G1	psL	ENSGACG00000003927	DHX37	-
groupXIII	1458354	1552216	G1	psL	ENSGACG00000003986	C22orf39	-
groupXIII	1458354	1552216	G1	psL	ENSGACG00000003987	MRPL40	-
groupXIII	1458354	1552216	G1	psL	ENSGACG00000003989	ACAA2	metabolic process, catalytic activity, transferase activity, transferring acyl groups other than amino-acyl groups
groupXIII	1458354	1552216	G1	psL	ENSGACG00000004060	-	microtubule-based process, microtubule associated complex
groupXIII	1458354	1552216	G1	psL	ENSGACG00000004070	POLB	DNA repair, DNA binding, catalytic activity, DNA-directed DNA polymerase activity, nucleotidyltransferase activity
groupXIII	1458354	1552216	G1	psL	ENSGACG00000004119	VDAC3 (2 of 2)	transmembrane transport, anion transport, regulation of anion transport, mitochondrial outer membrane, voltage-gated anion channel activity
groupXIII	1458354	1552216	G1	psL	ENSGACG00000003912	SUB1	regulation of transcription, DNA-dependent, DNA binding, binding, transcription coactivator activity
groupXIII	1458354	1552216	G1	psL	ENSGACG00000003940	UFD1L	ubiquitin-dependent protein catabolic process, response to chemical stimulus

groupXIII	1458354	1552216	G1	psL	ENSGACG00000004055	RPL6	translation, embryo development, intracellular, ribosome, structural constituent of ribosome
groupXIII	1458354	1552216	G1	psL	ENSGACG00000004114	C16orf48	-
groupXIII	2960498	2986859	G1	psL	ENSGACG00000005210	IMP4	hydrolase activity
groupXIII	2960498	2986859	G1	psL	ENSGACG00000005251	TRAF2 (2 of 2)	signal transduction, regulation of apoptotic process, DNA repair, DNA binding, zinc ion binding, metal ion binding, protein binding
groupXIII	2960498	2986859	G1	psL	ENSGACG00000005204	ABCB9	-
groupXIII	2960498	2986859	G1	psL	ENSGACG00000005242	C9orf86	-
groupXIII	6936681	6966411	G1	-	-	-	-
groupXIII	7848350	7921728	G1	psR	-	-	-
groupXIII	8191581	8270033	G1	n	ENSGACG00000008122	MLXIP	regulation of transcription, DNA-dependent, nucleus
groupXIII	8191581	8270033	G1	n	ENSGACG00000008178	RSRC2	-
groupXIII	8191581	8270033	G1	n	ENSGACG00000008111	WDR66	protein binding
groupXIII	8191581	8270033	G1	n	ENSGACG00000008128	LRRC43	protein binding
groupXIII	8191581	8270033	G1	n	ENSGACG00000008142	CLIP1 (1 of 2)	regulation of transcription, DNA-dependent, sequence-specific DNA binding transcription factor activity, sequence-specific DNA binding
groupXIII	8191581	8270033	G1	n	ENSGACG00000008163	ZCCHC8	nucleic acid binding, zinc ion binding
groupXIII	8191581	8270033	G1	n	ENSGACG00000008197	KNTC1	-
groupXIII	8501371	8534372	G1	psR	ENSGACG00000008464	ANK1 (2 of 2)	protein binding
groupXIII	8689352	8727457	G1	psR	ENSGACG00000008603	EWSR1	intracellular, nucleic acid binding, zinc ion binding
groupXIII	8689352	8727457	G1	psR	ENSGACG00000008626	RHBDD3	integral to membrane, serine-type endopeptidase activity
groupXIII	8689352	8727457	G1	psR	ENSGACG00000008631	EMID1	-
groupXIII	8821619	8870619	G1	psR	ENSGACG00000008754	-	neurotransmitter transport, integral to membrane, neurotransmitter:sodium symporter activity
groupXIII	8821619	8870619	G1	psR	ENSGACG00000008766	-	translation, intracellular, ribosome, structural constituent of ribosome
groupXIII	8821619	8870619	G1	psR	ENSGACG00000008769	WSCD2	phosphorylation, metabolic process, kinase activity, phosphotransferase activity, carboxyl group as acceptor
groupXIII	8871328	8960771	G1	n	ENSGACG00000008778	CMKLR1 (1 of 2)	G-protein coupled receptor signaling pathway, integral to membrane
groupXIII	8871328	8960771	G1	n	ENSGACG00000008784	FICD	protein binding
groupXIII	8871328	8960771	G1	n	ENSGACG00000008801	ISCU (1 of 2)	iron-sulfur cluster assembly, iron ion binding, iron-sulfur cluster binding
groupXIII	8871328	8960771	G1	n	ENSGACG00000008813	-	-
groupXIII	8871328	8960771	G1	n	ENSGACG00000008814	TMEM119 (1 of 2)	-
groupXIII	8871328	8960771	G1	n	ENSGACG00000008861	DAO	oxidation-reduction process, oxidoreductase activity, D-amino-acid oxidase activity
groupXIII	8871328	8960771	G1	n	ENSGACG00000008787	SART3	RNA processing, intracellular, nucleic acid binding
groupXIII	8871328	8960771	G1	n	ENSGACG00000008856	SSH1 (1 of 2)	protein dephosphorylation, dephosphorylation, protein tyrosine/serine/threonine phosphatase activity, phosphatase activity
groupXIII	8871328	8960771	G1	n	ENSGACG00000008816	CORO1C (1 of 2)	protein binding
groupXIII	8965994	9005994	G1	psR	ENSGACG00000008917	USP30	ubiquitin-dependent protein catabolic process, ubiquitin thiolesterase activity
groupXIII	8965994	9005994	G1	psR	ENSGACG00000008931	ALKBH2	oxidoreductase activity
groupXIII	8965994	9005994	G1	psR	ENSGACG00000008939	UNG	DNA repair, base-excision repair, hydrolase activity, hydrolyzing N-glycosyl compounds, uracil DNA N-glycosylase activity
groupXIII	8965994	9005994	G1	psR	ENSGACG00000008963	PXMP2	integral to membrane
groupXIII	8965994	9005994	G1	psR	ENSGACG00000008971	ISG15	protein binding
groupXIII	8965994	9005994	G1	psR	ENSGACG00000008973	GATC	regulation of translational fidelity
groupXIII	8965994	9005994	G1	psR	ENSGACG00000008884	-	transmembrane transport, integral to membrane, transmembrane transporter activity
groupXIII	8965994	9005994	G1	psR	ENSGACG00000008977	SGSM1 (1 of 2)	regulation of Rab GTPase activity, intracellular, Rab GTPase activator activity
groupXIII	9145297	9173731	G1	psR	ENSGACG00000009149	MVK	phosphorylation, isoprenoid biosynthetic process, cytoplasm, ATP binding, kinase activity, mevalonate kinase activity
groupXIII	9145297	9173731	G1	psR	ENSGACG00000009157	-	oxidation-reduction process, metabolic process, oxidoreductase activity
groupXIII	9176940	9292143	G1	psL	ENSGACG00000009166	UBE3B	cellular protein modification process, intracellular, protein binding, acid-amino acid ligase activity
groupXIII	9176940	9292143	G1	psL	ENSGACG00000009196	KCTD10	potassium ion transport, membrane, voltage-gated potassium channel complex, protein binding, voltage-gated potassium channel activity
groupXIII	9176940	9292143	G1	psL	ENSGACG00000009294	P2RX2	ion transport, cation transport, ion transmembrane transport, membrane, receptor activity, ATP binding, ion channel activity, extracellular ATP-gated cation channel activity
groupXIII	9176940	9292143	G1	psL	ENSGACG00000009302	PES1	cell proliferation, intracellular, nucleolus,

groupXIII	9176940	9292143	G1	psL	ENSGACG00000009328	GAL3ST1 (1 of 2)	biosynthetic process, integral to membrane, Golgi apparatus, galactosylceramide sulfotransferase activity
groupXIII	9176940	9292143	G1	psL	ENSGACG00000009334	LZTR1	ATP binding, protein binding
groupXIII	9176940	9292143	G1	psL	ENSGACG00000009352	CLDN22	membrane, tight junction, structural molecule activity
groupXIII	9176940	9292143	G1	psL	ENSGACG00000021327	-	-
groupXIII	9176940	9292143	G1	psL	ENSGACG00000009207	MYO1H (2 of 2)	myosin complex, motor activity, ATP binding, protein binding
groupXIII	9176940	9292143	G1	psL	ENSGACG00000009215	FOXN4	regulation of transcription, DNA-dependent, regulation of heart contraction, positive regulation of transcription, DNA-dependent, sequence-specific DNA binding transcription factor activity, sequence-specific DNA binding
groupXIII	9176940	9292143	G1	psL	ENSGACG00000009222	ACACB	metabolic process, fatty acid biosynthetic process, ATP binding, catalytic activity, ligase activity, acetyl-CoA carboxylase activity
groupXIII	9176940	9292143	G1	psL	ENSGACG00000009265	POLE	-
groupXIII	9176940	9292143	G1	psL	ENSGACG00000009355	AIFM3	oxidation-reduction process, oxidoreductase activity, flavin adenine dinucleotide binding, 2 iron, 2 sulfur cluster binding
groupXIII	9176940	9292143	G1	psL	ENSGACG00000009399	-	-
groupXIII	9314804	9336437	G1	n	ENSGACG00000009510	-	-
groupXIII	9314804	9336437	G1	n	ENSGACG00000009529	-	oxidation-reduction process, metabolic process, oxidoreductase activity, acting on the CH-CH group of donors, acyl-CoA dehydrogenase activity
groupXIII	9314804	9336437	G1	n	ENSGACG00000009534	-	-
groupXIII	9314804	9336437	G1	n	ENSGACG00000009515	MORC2	-
groupXIII	9367924	9402924	G1	n	ENSGACG00000009556	PLEKHA2	protein binding
groupXIII	9367924	9402924	G1	n	ENSGACG00000009561	-	-
groupXIII	9367924	9402924	G1	n	ENSGACG00000009550	TBX5 (2 of 2)	regulation of transcription, DNA-dependent, nucleus, sequence-specific DNA binding transcription factor activity
groupXIII	9595080	9618080	G1	n	ENSGACG00000009839	SETBP1	DNA binding
groupXIII	9898412	9903412	G1	n	ENSGACG00000010152	SSBP2	nucleus, DNA binding
groupXIII	10323390	10382503	G1	n	ENSGACG00000010245	CCNH	regulation of cyclin-dependent protein kinase activity, holo TFIIH complex, protein kinase binding
groupXIII	10323390	10382503	G1	n	ENSGACG00000010269	-	-
groupXIII	10323390	10382503	G1	n	ENSGACG00000010257	TMEM161B	-
groupXIII	10417423	10454423	G1	psR	ENSGACG00000010270	MEF2C (1 of 2)	regulation of transcription, DNA-dependent, embryonic heart tube morphogenesis, nucleus, sequence-specific DNA binding transcription factor activity, sequence-specific DNA binding
groupXIII	10650723	10678723	G1	psL	ENSGACG00000010293	WDR54	protein binding
groupXIII	10650723	10678723	G1	psL	ENSGACG00000010296	NOL6	pathogenesis, RNA binding, hydrolase activity, acting on ester bonds, ribosome binding
groupXIII	10650723	10678723	G1	psL	ENSGACG00000010311	-	DNA binding, protein dimerization activity
groupXIII	10737507	10782507	G1	psL	-	-	-
groupXIII	13425189	13445189	G1	psR	ENSGACG00000011961	SYN1	-
groupXIII	13425189	13445189	G1	psR	ENSGACG00000011968	C9orf172 (2 of 2)	-
groupXIII	14222298	14253298	G1	psL	ENSGACG00000012305	GPR133	G-protein coupled receptor signaling pathway, cell surface receptor signaling pathway, neuropeptide signaling pathway, membrane, integral to membrane, G-protein coupled receptor activity, transmembrane signaling receptor activity
groupXIII	14222298	14253298	G1	psL	ENSGACG00000012316	ULK1 (2 of 2)	protein phosphorylation, ATP binding, protein kinase activity, transferase activity, transferring phosphorus-containing groups, protein serine/threonine kinase activity
groupXIII	14598812	14625972	G1	bg	ENSGACG00000012512	LRRTM4 (3 of 3)	protein binding, identical protein binding
groupXIII	14598812	14625972	G1	bg	ENSGACG00000012516	LRRTM4 (2 of 3)	protein binding
groupXIII	16949304	16969304	G1	psR	-	-	-
groupXIV	12877253	13015253	G1	psL	ENSGACG00000018234	-	nucleic acid binding, zinc ion binding
groupXIV	12877253	13015253	G1	psL	ENSGACG00000018236	RASSF6	signal transduction
groupXIV	12877253	13015253	G1	psL	ENSGACG00000018232	LPAR1	G-protein coupled receptor signaling pathway, lymphangiogenesis, angiogenesis, integral to membrane
groupXIV	12877253	13015253	G1	psL	ENSGACG00000018237	-	cysteine-type endopeptidase inhibitor activity
groupXIV	12877253	13015253	G1	psL	ENSGACG00000018235	MUSK	protein phosphorylation, ATP binding, protein kinase activity, protein binding, transferase activity, transferring phosphorus-containing groups, protein serine/threonine kinase activity, protein tyrosine kinase activity, protein phosphorylation, ATP binding, protein kinase activity, protein binding, transferase activity, transferring phosphorus-containing groups, protein serine/threonine kinase activity, protein tyrosine kinase activity
groupXIV	15167119	15188126	G1	n	ENSGACG00000018673	MEGF10	calcium ion binding, protein binding, calcium ion binding, protein binding

groupXV	7872184	7906502	G1	psL	ENSGACG00000009674	PTAFR	G-protein coupled receptor signaling pathway, integral to membrane
groupXV	13433359	13454359	G1	n	ENSGACG00000012839	-	protein phosphorylation, transmembrane receptor protein tyrosine kinase signaling pathway, membrane, ATP binding, protein kinase activity, transferase activity, transferring phosphorus-containing groups, protein serine/threonine kinase activity, transmembrane receptor protein tyrosine kinase activity, protein tyrosine kinase activity
groupXV	14169959	14221959	G1	psL	ENSGACG00000012929	BRE	-
groupXV	14169959	14221959	G1	psL	ENSGACG00000012914	RBKS	D-ribose metabolic process, phosphotransferase activity, alcohol group as acceptor, ribokinase activity
groupXV	14399344	14416344	G1	bg	ENSGACG00000013033	GABRR2 (2 of 2)	transport, ion transport, membrane, integral to membrane, postsynaptic membrane, extracellular ligand-gated ion channel activity
groupXV	14990463	15033463	G1	psR	-	-	-
groupXV	15038667	15073667	G1	n	-	-	-
groupXV	15255941	15359622	G1	psR	-	-	-
groupXV	15536203	15631381	G1	psL	ENSGACG00000013180	DNAL1	protein binding
groupXV	15536203	15631381	G1	psL	ENSGACG00000013199	C14orf80	-
groupXV	15536203	15631381	G1	psL	ENSGACG00000013205	PTH2	cAMP biosynthetic process, positive regulation of receptor activity, receptor agonist activity
groupXV	15536203	15631381	G1	psL	ENSGACG00000013212	RIN3	-
groupXV	15536203	15631381	G1	psL	ENSGACG00000013158	ATP10D	metabolic process, ATP biosynthetic process, cell redox homeostasis, phospholipid transport, membrane, integral to membrane, magnesium ion binding, ATP binding, catalytic activity, ATPase activity, coupled to transmembrane movement of ions, phosphorylative mechanism, phospholipid-translocating ATPase activity
groupXV	15536203	15631381	G1	psL	ENSGACG00000013166	DAPP1	protein binding
groupXV	15536203	15631381	G1	psL	ENSGACG00000013169	-	Mo-molybdopterin cofactor biosynthetic process, molybdopterin cofactor biosynthetic process
groupXV	15636748	15651748	G1	psL	-	-	-
groupXV	16086396	16128270	G1	n	ENSGACG00000022566	-	-
groupXV	16086396	16128270	G1	n	ENSGACG00000013343	KIF6	microtubule-based movement, ATP binding, nucleotide binding, nucleoside-triphosphatase activity, microtubule motor activity
groupXVI	653316	683138	G1	bg	-	-	-
groupXVI	5026718	5062718	G1	n	ENSGACG00000021409	-	-
groupXVI	5026718	5062718	G1	n	ENSGACG00000002287	OBSL1 (2 of 4)	protein binding
groupXVI	5026718	5062718	G1	n	ENSGACG00000002298	CTDSP1 (2 of 2)	phosphatase activity, protein binding
groupXVI	5267708	5325411	G1	n	-	-	-
groupXVI	5361122	5446122	G1	psR	-	-	-
groupXVI	5627841	5655125	G1	n	-	-	-
groupXVI	5738247	5792247	G1	n	ENSGACG00000002401	GULP1	oxidation-reduction process, metabolic process, oxidoreductase activity, protein binding
groupXVI	6079523	6094523	G1	n	-	-	-
groupXVI	6466859	6487882	G1	psL	ENSGACG00000002751	SLC35F5	-
groupXVI	6466859	6487882	G1	psL	ENSGACG00000002735	SYMPK	binding, protein binding
groupXVI	15734211	15751307	G1	-	ENSGACG00000007909	TMEM237 (1 of 2)	-
groupXVI	15734211	15751307	G1	-	ENSGACG00000007889	MPP4 (2 of 2)	protein binding
groupXVI	15734211	15751307	G1	-	ENSGACG00000007934	ABI2 (2 of 2)	cytoplasm, protein binding
groupXVII	4526970	4562403	G1	n	ENSGACG00000006050	TBC1D20	regulation of Rab GTPase activity, intracellular, Rab GTPase activator activity
groupXVII	4526970	4562403	G1	n	ENSGACG00000006038	ZNFX1	regulation of transcription, DNA-dependent, nucleus, intracellular, sequence-specific DNA binding transcription factor activity, zinc ion binding, electron carrier activity, oxidoreductase activity, nucleotide binding, protein binding, nucleoside-triphosphatase activity
groupXVII	4526970	4562403	G1	n	ENSGACG00000006056	RBCK1	embryonic cranial skeleton morphogenesis, intracellular, zinc ion binding, protein binding
groupXVII	4526970	4562403	G1	n	ENSGACG00000006069	NECAB3 (2 of 2)	antibiotic biosynthetic process, cytoplasm, calcium ion binding, oxidoreductase activity
groupXVII	7003850	7152959	G1	n	ENSGACG00000008085	-	-
groupXVII	7003850	7152959	G1	n	ENSGACG00000007965	HDAC7	-
groupXVII	7003850	7152959	G1	n	ENSGACG00000007975	VDR (2 of 2)	regulation of transcription, DNA-dependent, steroid hormone mediated signaling pathway, nucleus, sequence-specific DNA binding transcription factor activity, steroid hormone receptor activity, zinc ion binding, sequence-specific DNA binding
groupXVII	7003850	7152959	G1	n	ENSGACG00000007995	TENC1 (2 of 2)	intracellular signal transduction, proteolysis, zinc ion binding, protein binding, metallocarboxypeptidase activity
groupXVII	7003850	7152959	G1	n	ENSGACG00000008010	EIF4B	nucleic acid binding
groupXVII	7003850	7152959	G1	n	ENSGACG00000008033	KRT18 (2 of 2)	-

groupXVII	7193147	7267833	G1	n	ENSGACG00000008115	TGM2 (2 of 2)	peptide cross-linking, protein-glutamine gamma-glutamyltransferase activity
groupXVII	7193147	7267833	G1	n	ENSGACG00000008135	KIAA1755 (1 of 2)	regulation of Rho protein signal transduction, organ morphogenesis, mesoderm morphogenesis, intracellular, protein binding, Rho guanyl-nucleotide exchange factor activity
groupXVII	7331297	7393889	G1	psL	ENSGACG00000008148	DLGAP4 (2 of 2)	cell-cell signaling
groupXVII	7904088	7940830	G1	psR	ENSGACG00000008456	GOLGA7	-
groupXVII	7904088	7940830	G1	psR	ENSGACG00000008496	-	translational elongation, ribosome biogenesis, embryo development, intracellular, ribosome, structural constituent of ribosome
groupXVII	7904088	7940830	G1	psR	ENSGACG00000008420	LOXL2 (2 of 2)	oxidation-reduction process, membrane, copper ion binding, scavenger receptor activity, oxidoreductase activity, acting on the CH-NH2 group of donors, oxygen as acceptor
groupXVII	7904088	7940830	G1	psR	ENSGACG00000008447	R3HCC1	nucleic acid binding
groupXVII	8019358	8043359	G1	n	ENSGACG00000008650	CRYBB3 (2 of 2)	-
groupXVII	8019358	8043359	G1	n	ENSGACG00000008661	-	DNA topological change, DNA binding, ATP binding, DNA topoisomerase (ATP-hydrolyzing) activity
groupXVII	8019358	8043359	G1	n	ENSGACG00000008663	-	-
groupXVII	8019358	8043359	G1	n	ENSGACG00000008668	-	-
groupXVII	8019358	8043359	G1	n	ENSGACG00000008653	-	-
groupXVII	14489682	14501682	G1	psR	ENSGACG00000012106	MBD1	nucleus, DNA binding, zinc ion binding
groupXVII	14489682	14501682	G1	psR	ENSGACG00000012117	CXXC1 (1 of 2)	blood vessel development, DNA methylation on cytosine, hemopoiesis, DNA binding, zinc ion binding, protein binding
groupXVII	14547262	14601262	G1	n	ENSGACG00000012256	RSG1	small GTPase mediated signal transduction, protein transport, intracellular, GTP binding
groupXVII	14547262	14601262	G1	n	ENSGACG00000012265	-	-
groupXVII	14547262	14601262	G1	n	ENSGACG00000012289	EDEM1	membrane, calcium ion binding, mannosyl-oligosaccharide 1, 2-alpha-mannosidase activity
groupXVII	14547262	14601262	G1	n	ENSGACG00000012301	GNAT1	signal transduction, G-protein coupled receptor signaling pathway, small GTPase mediated signal transduction, intracellular, signal transducer activity, GTP binding, guanyl nucleotide binding
groupXVII	14547262	14601262	G1	n	ENSGACG00000012259	DDI2	protein binding
groupXVII	14547262	14601262	G1	n	ENSGACG00000012267	ARL8B (2 of 2)	small GTPase mediated signal transduction, intracellular protein transport, protein transport, intracellular, GTP binding
groupXVII	14547262	14601262	G1	n	ENSGACG00000012225	EPHA2 (2 of 2)	protein phosphorylation, membrane, ATP binding, protein kinase activity, protein binding, transferase activity, transferring phosphorus-containing groups, protein serine/threonine kinase activity, ephrin receptor activity, protein tyrosine kinase activity
groupXVIII	530149	558172	G1	-	ENSGACG00000004246	CNIH3	intracellular signal transduction, membrane
groupXVIII	4292126	4323126	G1	n	ENSGACG00000006336	-	-
groupXVIII	11291668	11306921	G1	n	ENSGACG00000011134	FAM84A	-
groupXVIII	11422986	11464495	G1	-	ENSGACG00000011162	DDX1	nucleic acid binding, ATP binding, protein binding, helicase activity, ATP-dependent helicase activity
groupXVIII	11422986	11464495	G1	-	ENSGACG00000011256	ITSN2 (2 of 2)	protein targeting, intracellular protein transport, regulation of Rho protein signal transduction, membrane, intracellular, calcium ion binding, protein binding, Rho guanyl-nucleotide exchange factor activity
groupXVIII	11422986	11464495	G1	-	ENSGACG00000011136	NBAS	nuclear-transcribed mRNA catabolic process, nonsense-mediated decay, embryo development, protein binding
groupXVIII	15953869	16029869	G1	psL	ENSGACG00000013577	-	protein phosphorylation, protein kinase activity, transferase activity, transferring phosphorus-containing groups
groupXVIII	15953869	16029869	G1	psL	ENSGACG00000013578	BUB1	protein phosphorylation, ATP binding, protein kinase activity, transferase activity, transferring phosphorus-containing groups, protein serine/threonine kinase activity
groupXVIII	15953869	16029869	G1	psL	ENSGACG00000013580	GLO1 (2 of 2)	metal ion binding, lactoylglutathione lyase activity
groupXVIII	15953869	16029869	G1	psL	ENSGACG00000013585	HEY2	regulation of transcription, DNA-dependent, blood vessel development, blood vessel morphogenesis, artery morphogenesis, dorsal aorta morphogenesis, negative regulation of transcription, DNA-dependent, heart development, Notch signaling pathway involved in arterial endothelial cell fate commitment, vasculogenesis, nucleus, DNA binding, protein binding
groupXVIII	15953869	16029869	G1	psL	ENSGACG00000013588	-	DNA binding
groupXVIII	15953869	16029869	G1	psL	ENSGACG00000013624	C14orf109	-
groupXVIII	15953869	16029869	G1	psL	ENSGACG00000013571	PEX7	protein binding
groupXVIII	15953869	16029869	G1	psL	ENSGACG00000013590	-	zinc ion binding, protein binding, ubiquitin-protein ligase activity
groupXVIII	15953869	16029869	G1	psL	ENSGACG00000013627	ASB2 (1 of 2)	intracellular signal transduction, protein binding
groupXX	5640315	5681686	G1	psR	ENSGACG00000006198	FAM84B	-
groupXX	5640315	5681686	G1	psR	ENSGACG00000006200	NSMCE2	-
groupXX	5640315	5681686	G1	psR	ENSGACG00000006206	KIAA0196	heart contraction, axonogenesis, neuron development,
groupXX	5640315	5681686	G1	psR	ENSGACG00000006234	EXT1 (2 of 2)	membrane, membrane

groupXX	6502867	6528298	G1	n	ENSGACG00000006520	ADCY2 (1 of 3)	intracellular signal transduction, cyclic nucleotide biosynthetic process, integral to membrane, adenylate cyclase activity, phosphorus-oxygen lyase activity
groupXX	6695206	6736484	G1	-	-	-	-
groupXX	7589434	7602435	G1	bg	ENSGACG00000006665	-	-
groupXX	7589434	7602435	G1	bg	ENSGACG00000006682	-	binding
groupXX	7589434	7602435	G1	bg	ENSGACG00000006650	MTSS1 (1 of 2)	signal transduction, filopodium assembly, protein binding, cytoskeletal adaptor activity, SH3 domain binding
groupXX	7589434	7602435	G1	bg	ENSGACG00000006686	COLEC10	binding
groupXX	8092576	8142761	G1	psR	ENSGACG00000007151	UBXN11	protein binding
groupXX	8092576	8142761	G1	psR	ENSGACG00000007170	WDTC1	protein binding
groupXX	8092576	8142761	G1	psR	ENSGACG00000007178	TMEM222 (2 of 2)	-
groupXX	8092576	8142761	G1	psR	ENSGACG00000007188	-	protein binding
groupXX	8092576	8142761	G1	psR	ENSGACG00000007195	-	-
groupXX	8092576	8142761	G1	psR	ENSGACG00000007198	NR0B2	regulation of transcription, DNA-dependent, steroid hormone mediated signaling pathway, nucleus, sequence-specific DNA binding transcription factor activity, steroid hormone receptor activity
groupXX	8092576	8142761	G1	psR	ENSGACG00000007199	GPATCH3	intracellular, nucleic acid binding,
groupXX	8092576	8142761	G1	psR	ENSGACG00000007141	FEZ2 (2 of 2)	-
groupXX	8092576	8142761	G1	psR	ENSGACG00000007163	CEP85	-
groupXX	8092576	8142761	G1	psR	ENSGACG00000007225	GPN2	nucleotide binding
groupXX	8092576	8142761	G1	psR	ENSGACG00000007194	C1orf172	-
groupXX	8144053	8397146	G1	n	ENSGACG00000007244	ARID1A (2 of 2)	intracellular, DNA binding, binding
groupXX	8144053	8397146	G1	n	ENSGACG00000007253	PIGV	GPI anchor biosynthetic process, integral to membrane, endoplasmic reticulum membrane, ATP binding, ATPase activity, transferase activity, transferring hexosyl groups, GPI anchor biosynthetic process, integral to membrane, endoplasmic reticulum membrane, transferase activity, transferring hexosyl groups
groupXX	8144053	8397146	G1	n	ENSGACG00000007261	ZDHHC18 (2 of 2)	regulation of transcription, DNA-dependent, nucleus, sequence-specific DNA binding transcription factor activity, zinc ion binding
groupXX	8144053	8397146	G1	n	ENSGACG00000007269	FAM46B (1 of 2)	-
groupXX	8144053	8397146	G1	n	ENSGACG00000007286	TCEA3	transcription, DNA-dependent, regulation of transcription, DNA-dependent, regulation of transcription elongation, DNA-dependent, transcription elongation, DNA-dependent, regulation of transcription from RNA polymerase II promoter, nucleus, nucleic acid binding, DNA binding, zinc ion binding, translation elongation factor activity
groupXX	8144053	8397146	G1	n	ENSGACG00000007300	OPRD1 (1 of 2)	G-protein coupled receptor signaling pathway, integral to membrane
groupXX	8144053	8397146	G1	n	ENSGACG00000007316	RCC1	-
groupXX	8144053	8397146	G1	n	ENSGACG00000007346	PHACTR4 (1 of 2)	regulation of transcription, DNA-dependent, transcription initiation, DNA-dependent, sequence-specific DNA binding transcription factor activity, DNA binding, sigma factor activity
groupXX	8144053	8397146	G1	n	ENSGACG00000007350	HIVEP3	intracellular, nucleic acid binding, zinc ion binding
groupXX	8144053	8397146	G1	n	ENSGACG00000007354	SERINC2 (2 of 2)	membrane, 2 iron, 2 sulfur cluster binding
groupXX	8144053	8397146	G1	n	ENSGACG00000007379	STMN1 (2 of 2)	intracellular signal transduction
groupXX	8144053	8397146	G1	n	ENSGACG00000007389	PAQR7	oocyte maturation, response to steroid hormone stimulus, negative regulation of adenylate cyclase activity, activation of MAPK activity, signal transduction, steroid hormone mediated signaling pathway, integral to membrane, plasma membrane, steroid binding, steroid hormone receptor activity
groupXX	8399430	8497211	G1	psR	ENSGACG00000007392	PTPRU (2 of 2)	protein dephosphorylation, dephosphorylation, membrane, protein tyrosine phosphatase activity, phosphatase activity, protein binding
groupXX	8632644	8718644	G1	-	ENSGACG00000007482	-	intracellular, zinc ion binding
groupXX	8632644	8718644	G1	-	ENSGACG00000007484	-	intracellular, zinc ion binding
groupXX	8632644	8718644	G1	-	ENSGACG00000007488	-	G-protein coupled receptor signaling pathway, integral to membrane
groupXX	8632644	8718644	G1	-	ENSGACG00000007491	-	G-protein coupled receptor signaling pathway, integral to membrane
groupXX	8632644	8718644	G1	-	ENSGACG00000007493	-	G-protein coupled receptor signaling pathway, integral to membrane
groupXX	8632644	8718644	G1	-	ENSGACG00000007496	HAS1	transferase activity, transferring hexosyl groups
groupXX	8632644	8718644	G1	-	ENSGACG00000007461	-	proteolysis, catalytic activity, serine-type endopeptidase activity
groupXX	8881054	8921054	G1	n	ENSGACG00000007563	-	regulation of Rab GTPase activity, intracellular, Rab GTPase activator activity
groupXX	8881054	8921054	G1	n	ENSGACG00000007594	CTRL	proteolysis, catalytic activity, serine-type endopeptidase activity

groupXX	8881054	8921054	G1	n	ENSGACG00000007597	-	G-protein coupled receptor signaling pathway, integral to membrane
groupXX	8881054	8921054	G1	n	ENSGACG00000007600	LIN37	-
groupXX	8881054	8921054	G1	n	ENSGACG00000007618	-	-
groupXX	8881054	8921054	G1	n	ENSGACG00000007622	-	-
groupXX	8881054	8921054	G1	n	ENSGACG00000007626	PSENEN	negative regulation of neuron apoptotic process, negative regulation of apoptotic process
groupXX	8881054	8921054	G1	n	ENSGACG00000007569	-	-
groupXX	9112424	9126606	G1	bg	ENSGACG00000007711	NPHS1	skeletal muscle fiber development, kidney development, protein binding
groupXX	10396730	10494942	G1	psL	ENSGACG00000008982	-	intracellular, zinc ion binding
groupXX	10563875	10588247	G1	psL	-	-	-
groupXX	10627010	10653600	G1	bg	ENSGACG00000009048	ERP44	cell redox homeostasis
groupXX	10627010	10653600	G1	bg	ENSGACG00000009064	TEX10	oxidation-reduction process, oxidoreductase activity, binding, protein binding
groupXX	10627010	10653600	G1	bg	ENSGACG00000009056	INVS	kidney development, cloaca development, body morphogenesis, pronephros morphogenesis, convergent extension involved in gastrulation, protein binding, phosphopantetheine binding
groupXX	10658810	10682147	G1	psL	ENSGACG00000009064	TEX10	oxidation-reduction process, oxidoreductase activity, binding, protein binding
groupXX	10658810	10682147	G1	psL	ENSGACG00000009075	-	nucleic acid binding, zinc ion binding
groupXX	10658810	10682147	G1	psL	ENSGACG00000009081	RBM12B (2 of 2)	nucleic acid binding
groupXX	10658810	10682147	G1	psL	ENSGACG00000009082	TMEM67	-
groupXX	10658810	10682147	G1	psL	ENSGACG00000009093	PDP1	protein dephosphorylation, protein serine/threonine phosphatase complex, catalytic activity, protein serine/threonine phosphatase activity
groupXX	10722399	10757399	G1	psR	ENSGACG00000009169	ESRP1	nucleic acid binding
groupXX	10722399	10757399	G1	psR	ENSGACG00000009138	KIAA1429	binding
groupXX	10722399	10757399	G1	psR	ENSGACG00000009175	EPB41L4B	oxidation-reduction process, malate metabolic process, cytoskeleton, malate dehydrogenase activity
groupXX	10851499	10884499	G1	psR	ENSGACG00000009299	ABCB4	transport, transmembrane transport, integral to membrane, ATP binding, nucleotide binding, ATPase activity, ATPase activity, coupled to transmembrane movement of substances, nucleoside-triphosphatase activity
groupXX	10851499	10884499	G1	psR	ENSGACG00000009330	RUNDC3B	-
groupXX	10851499	10884499	G1	psR	ENSGACG00000009260	CROT (2 of 2)	fatty-acyl-CoA binding
groupXX	10851499	10884499	G1	psR	ENSGACG00000009281	CROT (1 of 2)	-
groupXX	11043669	11097669	G1	psR	ENSGACG00000009447	GSK3A (2 of 2)	protein phosphorylation, heart looping, cardiac muscle cell development, dorsal/ventral pattern formation, ATP binding, protein kinase activity, transferase activity, transferring phosphorus-containing groups, protein serine/threonine kinase activity
groupXX	11043669	11097669	G1	psR	ENSGACG00000009451	-	-
groupXX	11043669	11097669	G1	psR	ENSGACG00000009439	ZNF574	definitive hemopoiesis, intracellular, DNA binding, zinc ion binding
groupXX	11043669	11097669	G1	psR	ENSGACG00000009449	ZNF526	transcription, DNA-dependent, intracellular, nucleic acid binding, zinc ion binding, metal ion binding
groupXX	11179475	11252505	G1	n	ENSGACG00000009471	POU2F2 (1 of 2)	regulation of transcription, DNA-dependent, nucleus, sequence-specific DNA binding transcription factor activity, DNA binding, sequence-specific DNA binding, protein binding
groupXX	11719578	11821763	G1	n	ENSGACG00000010169	TAPBPL	protein binding
groupXX	11719578	11821763	G1	n	ENSGACG00000010179	VAMP1	vesicle-mediated transport, integral to membrane
groupXX	11719578	11821763	G1	n	ENSGACG00000010193	MRPL51	-
groupXX	11719578	11821763	G1	n	ENSGACG00000010195	PLEKHG6	regulation of Rho protein signal transduction, intracellular, Rho guanyl-nucleotide exchange factor activity
groupXX	11719578	11821763	G1	n	ENSGACG00000010200	TNFRSF1A	signal transduction, binding, protein binding
groupXX	11719578	11821763	G1	n	ENSGACG00000010207	IFFO1 (2 of 2)	-
groupXX	11719578	11821763	G1	n	ENSGACG00000010213	-	-
groupXX	11719578	11821763	G1	n	ENSGACG00000010215	-	G-protein coupled receptor signaling pathway, integral to membrane
groupXX	11719578	11821763	G1	n	ENSGACG00000010219	-	oxidation-reduction process, glucose metabolic process, oxidoreductase activity, acting on the aldehyde or oxo group of donors, NAD or NADP as acceptor, NAD binding
groupXX	11719578	11821763	G1	n	ENSGACG00000010291	-	extracellular region, hormone activity
groupXX	11719578	11821763	G1	n	ENSGACG00000022897	-	-
groupXX	11719578	11821763	G1	n	ENSGACG00000022805	-	-
groupXX	11719578	11821763	G1	n	ENSGACG00000010330	-	translational elongation, intracellular, translation elongation factor activity

groupXX	11719578	11821763	G1	n	ENSGACG00000010317	NOP2	rRNA processing, RNA binding, methyltransferase activity, S-adenosylmethionine-dependent methyltransferase activity
groupXX	11719578	11821763	G1	n	ENSGACG00000010292	NCAPD2	mitotic sister chromatid segregation, binding, viral capsid, zinc ion binding, protein binding, structural molecule activity
groupXX	12498889	12525889	G1	bg	ENSGACG00000011540	SPHK2	protein kinase C-activating G-protein coupled receptor signaling pathway, diacylglycerol kinase activity
groupXX	12498889	12525889	G1	bg	ENSGACG00000011594	-	integral to membrane
groupXX	12498889	12525889	G1	bg	ENSGACG00000011515	CYTH2	regulation of ARF protein signal transduction, intracellular, protein binding, ARF guanyl-nucleotide exchange factor activity
groupXX	12498889	12525889	G1	bg	ENSGACG00000011547	RPL18	translation, intracellular, ribosome, structural constituent of ribosome
groupXXI	1217656	1234082	G1	bg	-	-	-
groupXXI	1511040	1532040	G1	psR	-	-	-
groupXXI	4520028	4559613	G1	n	ENSGACG00000002390	CTNND2 (2 of 2)	binding, protein binding
groupI	4487827	4543283	Us	n	ENSGACG00000007046	SLC33A1	central nervous system neuron axonogenesis, membrane
groupI	4487827	4543283	Us	n	ENSGACG00000007060	-	G-protein coupled receptor signaling pathway, integral to membrane, G-protein coupled receptor activity
groupI	4487827	4543283	Us	n	ENSGACG00000007027	RPS6KB1 (1 of 2)	protein phosphorylation, ATP binding, protein kinase activity, transferase activity, transferring phosphorus-containing groups, protein serine/threonine kinase activity
groupI	4487827	4543283	Us	n	ENSGACG00000007053	GMPS	purine nucleotide biosynthetic process, GMP biosynthetic process, ATP binding, GMP synthase (glutamine-hydrolyzing) activity
groupI	7530747	7556318	Us	-	ENSGACG00000008485	-	metabolic process, methyltransferase activity
groupI	7530747	7556318	Us	-	ENSGACG00000008504	PRPF8	nuclear mRNA splicing, via spliceosome, spliceosomal complex, protein binding
groupI	7530747	7556318	Us	-	ENSGACG00000008566	RILP	DNA binding, catalytic activity
groupI	8107799	8162799	Us	psL	ENSGACG00000009072	GRIK4	transport, membrane, outer membrane-bounded periplasmic space, transporter activity, ionotropic glutamate receptor activity, extracellular-glutamate-gated ion channel activity
groupI	8199079	8211862	Us	psR	ENSGACG00000009072	GRIK4	transport, membrane, outer membrane-bounded periplasmic space, transporter activity, ionotropic glutamate receptor activity, extracellular-glutamate-gated ion channel activity
groupI	8214565	8299728	Us	n	-	-	-
groupI	9158598	9220534	Us	-	ENSGACG00000009469	EGLN2	oxidation-reduction process, iron ion binding, oxidoreductase activity, oxidoreductase activity, acting on paired donors, with incorporation or reduction of molecular oxygen, L-ascorbic acid binding
groupI	9158598	9220534	Us	-	ENSGACG00000009491	TMEM160	-
groupI	9158598	9220534	Us	-	ENSGACG00000009494	NPAS3	regulation of transcription, DNA-dependent, signal transduction, nucleus, signal transducer activity, protein binding
groupI	9470441	9597441	Us	n	ENSGACG00000009621	-	growth, growth factor activity
groupI	9470441	9597441	Us	n	ENSGACG00000009625	-	nucleosome assembly, nucleus, intracellular, nucleosome, DNA binding, sequence-specific DNA binding
groupI	9470441	9597441	Us	n	ENSGACG00000009639	SLC30A10	transmembrane transport, cation transport, integral to membrane, ATP binding, cation transmembrane transporter activity
groupI	9470441	9597441	Us	n	ENSGACG00000022167	-	-
groupI	9470441	9597441	Us	n	ENSGACG00000009641	MRPL28	-
groupI	9470441	9597441	Us	n	ENSGACG00000009656	RELB	regulation of transcription, DNA-dependent, nucleus, sequence-specific DNA binding transcription factor activity, protein binding
groupI	9470441	9597441	Us	n	ENSGACG00000009693	TBCB	-
groupI	9470441	9597441	Us	n	ENSGACG00000009721	SIX5	regulation of transcription, DNA-dependent, nucleus, sequence-specific DNA binding transcription factor activity, sequence-specific DNA binding, protein binding
groupI	9470441	9597441	Us	n	ENSGACG00000009726	-	regulation of transcription, DNA-dependent, nucleus, sequence-specific DNA binding transcription factor activity, sequence-specific DNA binding, protein binding
groupI	9470441	9597441	Us	n	ENSGACG00000009732	QPCTL	proteolysis, peptidase activity
groupI	9470441	9597441	Us	n	ENSGACG00000009741	RRM1 (1 of 2)	oxidation-reduction process, DNA replication, ribonucleoside-diphosphate reductase complex, ATP binding, ribonucleoside-diphosphate reductase activity, thioredoxin disulfide as acceptor
groupI	9470441	9597441	Us	n	ENSGACG00000009629	PPP1R37	protein binding
groupI	9470441	9597441	Us	n	ENSGACG00000009668	CLPTM1	-
groupI	10957095	11015569	Us	-	ENSGACG00000010514	-	-
groupI	10957095	11015569	Us	-	ENSGACG00000010517	HSPA8 (2 of 2)	-
groupI	10957095	11015569	Us	-	ENSGACG00000010543	-	membrane, integral to membrane
groupI	10957095	11015569	Us	-	ENSGACG00000010488	PICALM (2 of 2)	phospholipid binding
groupI	10957095	11015569	Us	-	ENSGACG00000010497	SYTL2 (2 of 2)	intracellular protein transport, protein binding, Rab GTPase binding
groupI	10957095	11015569	Us	-	ENSGACG00000010555	-	protein binding

groupI	11277968	11320968	Us	psL -	-	-
groupI	12165614	12232642	Us	psL	ENSGACG00000011118 -	-
groupI	12165614	12232642	Us	psL	ENSGACG00000011119 TADA2A	intracellular, DNA binding, zinc ion binding, protein binding
groupI	12165614	12232642	Us	psL	ENSGACG00000011101 HNF1B	regulation of transcription, DNA-dependent, positive regulation of transcription, DNA-dependent, nucleus, sequence-specific DNA binding transcription factor activity, DNA binding, sequence-specific DNA binding, binding, protein binding
groupI	12165614	12232642	Us	psL	ENSGACG00000011112 SYNRG	protein binding
groupI	12408358	12521538	Us	psR	ENSGACG00000011316 DIABLO (2 of 2)	induction of apoptosis, activation of cysteine-type endopeptidase activity involved in apoptotic process, mitochondrion
groupI	12408358	12521538	Us	psR	ENSGACG00000011318 -	G-protein coupled receptor signaling pathway, integral to membrane
groupI	12408358	12521538	Us	psR	ENSGACG00000011320 -	G-protein coupled receptor signaling pathway, integral to membrane
groupI	12408358	12521538	Us	psR	ENSGACG00000011322 -	-
groupI	12408358	12521538	Us	psR	ENSGACG00000011325 -	G-protein coupled receptor signaling pathway, integral to membrane
groupI	12408358	12521538	Us	psR	ENSGACG00000011326 -	G-protein coupled receptor signaling pathway, integral to membrane
groupI	12408358	12521538	Us	psR	ENSGACG00000011330 DIABLO (1 of 2)	induction of apoptosis, activation of cysteine-type endopeptidase activity involved in apoptotic process, mitochondrion
groupI	12408358	12521538	Us	psR	ENSGACG00000011337 PVRL3 (1 of 2)	protein binding
groupI	12408358	12521538	Us	psR	ENSGACG00000011342 -	-
groupI	13139791	13157031	Us	psL	ENSGACG00000011405 STIM1	-
groupI	13384179	13414179	Us	n	- -	-
groupI	15770959	15799963	Us	n	ENSGACG00000012339 -	positive regulation of cysteine-type endopeptidase activity involved in apoptotic process, positive regulation of apoptotic process
groupI	15770959	15799963	Us	n	ENSGACG00000012342 SAE1	catalytic activity
groupI	15770959	15799963	Us	n	ENSGACG00000012374 ZC3H4	-
groupI	21231916	21264234	Us	n	- -	-
groupII	2903840	2937453	Us	n	- -	-
groupII	6647219	6663905	Us	bg	- -	-
groupII	7879800	8030972	Us	psL	ENSGACG00000015368 GPC5	membrane, proteinaceous extracellular matrix, protein binding, heparan sulfate proteoglycan binding
groupII	7879800	8030972	Us	psL	ENSGACG00000015374 GPC6 (1 of 2)	membrane, proteinaceous extracellular matrix, protein binding, heparan sulfate proteoglycan binding
groupII	10216999	10285601	Us	n	ENSGACG00000015701 -	RNA metabolic process, binding, protein binding
groupII	10216999	10285601	Us	n	ENSGACG00000015700 CTR9	RNA processing, pigmentation, neural crest cell development, somitogenesis, intracellular, Cdc73/Paf1 complex, nucleus, protein binding
groupII	10216999	10285601	Us	n	ENSGACG00000015704 GALNTL4 (1 of 2)	-
groupII	10216999	10285601	Us	n	ENSGACG00000015698 MRVI1	-
groupII	10991396	11067396	Us	-	ENSGACG00000015782 -	nucleic acid binding, protein binding
groupII	11994580	12025583	Us	psL	ENSGACG00000015954 DBNDD1	cytoplasm
groupII	11994580	12025583	Us	psL	ENSGACG00000015955 MTHFSD	folic acid-containing compound biosynthetic process, nucleic acid binding, ATP binding, 5-formyltetrahydrofolate cyclo-ligase activity
groupII	11994580	12025583	Us	psL	ENSGACG00000015956 FOXF1	regulation of transcription, DNA-dependent, sequence-specific DNA binding transcription factor activity, sequence-specific DNA binding
groupII	12297958	12324764	Us	psL	ENSGACG00000015978 CDYL2	metabolic process, nucleus, catalytic activity
groupII	12361189	12448667	Us	psL -	-	-
groupII	12450247	12503247	Us	psL -	-	-
groupII	13198025	13224025	Us	psL	ENSGACG00000016050 CSMD1	-
groupII	17198240	17218357	Us	bg	- -	-
groupIII	5824829	5858829	Us	n	- -	-
groupIII	6296853	6412575	Us	psL	ENSGACG00000014942 ANKRD45	protein binding
groupIII	6296853	6412575	Us	psL	ENSGACG00000014944 -	nucleic acid binding
groupIII	6296853	6412575	Us	psL	ENSGACG00000022137 -	-
groupIII	6296853	6412575	Us	psL	ENSGACG00000014947 FITM1 (2 of 2)	regulation of transcription, DNA-dependent, G-protein coupled receptor signaling pathway, nucleus, integral to membrane, sequence-specific DNA binding
groupIII	6296853	6412575	Us	psL	ENSGACG00000014948 -	vesicle-mediated transport, membrane, myosin complex, motor activity, ATP binding, nucleotide binding, protein binding

groupIII	6296853	6412575	Us	psL	ENSGACG00000014960	-	vesicle-mediated transport, embryonic heart tube development, ventricular cardiac myofibril development, membrane, myosin complex, motor activity, ATP binding, nucleotide binding, protein binding
groupIII	11614526	11702466	Us	-	ENSGACG00000016730	-	carbohydrate metabolic process, catalytic activity, cation binding
groupIII	11614526	11702466	Us	-	ENSGACG00000016740	-	carbohydrate metabolic process, catalytic activity, cation binding
groupIII	11614526	11702466	Us	-	ENSGACG00000016719	-	signal transduction, cell adhesion, collagen, extracellular matrix structural constituent, receptor binding, structural molecule activity
groupIV	7589314	7721800	Us	n	ENSGACG00000017504	ENOX2 (1 of 2)	nucleic acid binding
groupIV	8324156	8359468	Us	-	ENSGACG00000017666	SLC16A2	transmembrane transport, integral to membrane
groupIV	8324156	8359468	Us	-	ENSGACG00000017676	-	-
groupIV	8324156	8359468	Us	-	ENSGACG00000017669	-	-
groupIV	8324156	8359468	Us	-	ENSGACG00000017671	F8	oxidation-reduction process, cell adhesion, oxidoreductase activity, copper ion binding
groupIV	8497040	8559841	Us	n	ENSGACG00000017689	TSC22D3	regulation of transcription, DNA-dependent, sequence-specific DNA binding transcription factor activity
groupIV	9314165	9376941	Us	n	ENSGACG00000017830	ZDHHC5 (2 of 2)	regulation of transcription, DNA-dependent, nucleus, sequence-specific DNA binding transcription factor activity, zinc ion binding
groupIV	9314165	9376941	Us	n	ENSGACG00000017836	-	protein binding
groupIV	9314165	9376941	Us	n	ENSGACG00000017837	SLC43A3 (2 of 2)	-
groupIV	9314165	9376941	Us	n	ENSGACG00000017843	-	-
groupIV	9314165	9376941	Us	n	ENSGACG00000017844	-	intracellular, zinc ion binding
groupIV	9314165	9376941	Us	n	ENSGACG00000017832	CLP1	-
groupIV	11062430	11085457	Us	n	ENSGACG00000018040	-	G-protein coupled receptor signaling pathway, negative regulation of adenylate cyclase activity, integral to membrane
groupIV	11062430	11085457	Us	n	ENSGACG00000018041	-	-
groupIV	11212342	11243185	Us	psL	-	-	-
groupIV	11559888	11612888	Us	psL	ENSGACG00000018137	EGR1	camera-type eye development
groupIV	12379339	12459749	Us	psL	ENSGACG00000018271	-	signal transduction, GTP catabolic process, small GTPase mediated signal transduction, intracellular protein transport, nucleocytoplasmic transport, protein transport, intracellular, GTPase activity, GTP binding
groupIV	12379339	12459749	Us	psL	ENSGACG00000022698	-	-
groupIV	12379339	12459749	Us	psL	ENSGACG00000018279	ACRC	protein binding
groupIV	12379339	12459749	Us	psL	ENSGACG00000018281	CETN2	nucleic acid binding, calcium ion binding, ATP binding, ATP-dependent helicase activity
groupIV	12379339	12459749	Us	psL	ENSGACG00000018285	NSDHL	cellular metabolic process, steroid biosynthetic process, extracellular polysaccharide biosynthetic process, catalytic activity, coenzyme binding, 3-beta-hydroxy-delta5-steroid dehydrogenase activity, dTDP-4-dehydrorhamnose reductase activity
groupIV	12379339	12459749	Us	psL	ENSGACG00000018286	-	-
groupIV	12379339	12459749	Us	psL	ENSGACG00000018287	ZNF185	zinc ion binding
groupIV	12379339	12459749	Us	psL	ENSGACG00000018273	OGT	RNA processing, intracellular, protein binding
groupIV	13315254	13353873	Us	-	ENSGACG00000018357	RMND5B	protein binding, protein binding, protein binding
groupIV	13315254	13353873	Us	-	ENSGACG00000018359	N4BP3	-
groupIV	13930138	13974395	Us	-	ENSGACG00000018432	STC2	extracellular region, hormone activity
groupIV	13930138	13974395	Us	-	ENSGACG00000018433	NKX2-5	regulation of transcription, DNA-dependent, embryonic heart tube development, heart looping, heart morphogenesis, cardiac muscle cell proliferation, cardiac muscle cell differentiation, heart development, nucleus, sequence-specific DNA binding transcription factor activity, sequence-specific DNA binding, protein binding
groupIV	15566478	15592478	Us	n	ENSGACG00000018543	HDX	regulation of transcription, DNA-dependent, sequence-specific DNA binding transcription factor activity, sequence-specific DNA binding, protein binding
groupIV	15566478	15592478	Us	n	ENSGACG00000018542	-	regulation of signal transduction, membrane, cytoplasm, protein binding
groupIV	15566478	15592478	Us	n	ENSGACG00000018545	RPS6KA6	protein phosphorylation, intracellular, zinc ion binding, ATP binding, protein kinase activity, transferase activity, transferring phosphorus-containing groups, protein serine/threonine kinase activity
groupIV	18755894	18802005	Us	psL	ENSGACG00000018882	PNPLA8 (2 of 2)	metabolic process, lipid metabolic process
groupIV	18755894	18802005	Us	psL	ENSGACG00000018875	NRCAM	protein binding
groupIV	18808297	18837297	Us	n	ENSGACG00000018888	D-JB9-(2-of-2)	heat shock protein binding
groupIV	18808297	18837297	Us	n	ENSGACG00000018891	-	oxidation-reduction process, oxidoreductase activity
groupIV	18808297	18837297	Us	n	ENSGACG00000018894	-	oxidation-reduction process, oxidoreductase activity
groupIV	18808297	18837297	Us	n	ENSGACG00000018887	THAP5	nucleic acid binding

groupIV	18839597	18879597	Us	psL	ENSGACG00000018910	TFEC	regulation of transcription, DNA-dependent, nucleus
groupIV	19235599	19294646	Us	n	ENSGACG00000018919	IFRD1	binding
groupIV	19235599	19294646	Us	n	ENSGACG00000018918	TMEM168 (2 of 2)	-
groupIV	19485417	19554417	Us	n	ENSGACG00000018926	LRRN3	protein binding
groupIV	19485417	19554417	Us	n	ENSGACG00000018927	-	-
groupIV	19671285	19726492	Us	-	ENSGACG00000018935	DHX57	nucleic acid binding, zinc ion binding, ATP binding, protein binding, helicase activity, ATP-dependent helicase activity
groupIV	19671285	19726492	Us	-	ENSGACG00000018938	FAM40B	nucleobase-containing compound metabolic process, nucleic acid binding, nucleotide binding, DNA-directed DNA polymerase activity
groupIV	19671285	19726492	Us	-	ENSGACG00000018942	AHCYL2 (1 of 2)	one-carbon metabolic process, adenosylhomocysteinase activity
groupIV	20101884	20148884	Us	-	-	-	-
groupIV	21550848	21710849	Us	psR	ENSGACG00000019098	ARL1	small GTPase mediated signal transduction, intracellular protein transport, protein transport, intracellular, GTP binding
groupIV	21550848	21710849	Us	psR	ENSGACG00000019105	FGD4 (2 of 2)	regulation of Rho protein signal transduction, intracellular, metal ion binding, protein binding, Rho guanyl-nucleotide exchange factor activity
groupIV	21550848	21710849	Us	psR	ENSGACG00000019107	MAPK11	proteolysis, protein phosphorylation, zinc ion binding, ATP binding, protein kinase activity, transferase activity, transferring phosphorus-containing groups, protein serine/threonine kinase activity, metallocarboxypeptidase activity, MAP kinase activity
groupIV	21550848	21710849	Us	psR	ENSGACG00000019110	-	protein binding, protein binding, protein binding
groupIV	21550848	21710849	Us	psR	ENSGACG00000019114	XRCC6BP1	-
groupIV	21550848	21710849	Us	psR	ENSGACG00000019116	SLC16A7	transmembrane transport, organic anion transport, integral to membrane, secondary active monocarboxylate transmembrane transporter activity
groupIV	21550848	21710849	Us	psR	ENSGACG00000019092	UTP20	-
groupIV	21550848	21710849	Us	psR	ENSGACG00000019104	KIAA1147 (2 of 2)	metabolic process, transferase activity, transferring acyl groups other than amino-acyl groups
groupIV	21550848	21710849	Us	psR	ENSGACG00000019108	MAPK12 (2 of 2)	protein phosphorylation, ATP binding, protein kinase activity, transferase activity, transferring phosphorus-containing groups, protein serine/threonine kinase activity, MAP kinase activity
groupIV	21550848	21710849	Us	psR	ENSGACG00000019113	RPAP3	metabolic process, transferase activity, protein binding
groupIV	21550848	21710849	Us	psR	ENSGACG00000019115	LRIG3	-
groupIV	21925482	22113230	Us	psL	ENSGACG00000019136	PLXNC1	signal transduction, intracellular
groupIV	21925482	22113230	Us	psL	ENSGACG00000019145	HCFC2	protein binding
groupIV	21925482	22113230	Us	psL	ENSGACG00000019147	ELK3	regulation of transcription, DNA-dependent, sequence-specific DNA binding transcription factor activity, sequence-specific DNA binding
groupIV	21925482	22113230	Us	psL	ENSGACG00000019150	PHYH	-
groupIV	21925482	22113230	Us	psL	ENSGACG00000019155	MCM10	-
groupIV	21925482	22113230	Us	psL	ENSGACG00000019159	CCDC3	-
groupIV	21925482	22113230	Us	psL	ENSGACG00000019163	CDC123	-
groupIV	21925482	22113230	Us	psL	ENSGACG00000019134	RERG	small GTPase mediated signal transduction, protein transport, intracellular, GTP binding, signal transduction, GTP catabolic process, small GTPase mediated signal transduction, intracellular protein transport, nucleocytoplasmic transport, protein transport, intracellular, GTPase activity, GTP binding
groupIV	21925482	22113230	Us	psL	ENSGACG00000019139	CCDC41	-
groupIV	21925482	22113230	Us	psL	ENSGACG00000019148	CDK17 (1 of 2)	protein phosphorylation, ATP binding, protein kinase activity, transferase activity, transferring phosphorus-containing groups, protein serine/threonine kinase activity
groupIV	21925482	22113230	Us	psL	ENSGACG00000019153	UCMA (1 of 2)	-
groupIV	21925482	22113230	Us	psL	ENSGACG00000019156	OPTN	-
groupIV	21925482	22113230	Us	psL	ENSGACG00000019160	CAMK1D (2 of 2)	protein phosphorylation, ATP binding, protein kinase activity, transferase activity, transferring phosphorus-containing groups, protein serine/threonine kinase activity
groupIV	25239236	25277236	Us	psR	-	-	-
groupIV	28413742	28430742	Us	psL	ENSGACG00000019659	TMEM60 (1 of 2)	-
groupIX	8639541	8750371	Us	bg	ENSGACG00000017767	GUCY1A3	intracellular signal transduction, cyclic nucleotide biosynthetic process, cGMP biosynthetic process, phosphorus-oxygen lyase activity, guanylate cyclase activity
groupIX	8639541	8750371	Us	bg	ENSGACG00000017790	FABP2 (1 of 2)	transport, binding, transporter activity, lipid binding
groupIX	8639541	8750371	Us	bg	ENSGACG00000017796	USP53 (2 of 2)	-

groupIX	8639541	8750371	Us	bg	ENSGACG00000017804	MYOZ2 (1 of 2)	-
groupIX	8639541	8750371	Us	bg	ENSGACG00000017814	SLC22A7 (2 of 2)	transmembrane transport, integral to membrane, transmembrane transporter activity
groupIX	8639541	8750371	Us	bg	ENSGACG00000017840	SOD3	oxidation-reduction process, superoxide metabolic process, metal ion binding
groupIX	8639541	8750371	Us	bg	ENSGACG00000017841	CCDC149 (2 of 2)	-
groupIX	8639541	8750371	Us	bg	ENSGACG00000017845	LGI2 (2 of 2)	carbohydrate metabolic process, catalytic activity, protein binding, carbohydrate binding
groupIX	8639541	8750371	Us	bg	ENSGACG00000017809	PSMD5	binding
groupIX	8639541	8750371	Us	bg	ENSGACG00000017826	DHX15 (1 of 2)	nucleic acid binding, ATP binding, nucleotide binding, helicase activity, ATP-dependent helicase activity, nucleoside-triphosphatase activity
groupIX	8639541	8750371	Us	bg	ENSGACG00000017850	MTTP	lipid transport, long-chain fatty acid transport, medium-chain fatty acid transport, digestion, lipid metabolic process, lipid transporter activity
groupIX	8639541	8750371	Us	bg	ENSGACG00000017771	GUCY1B3	intracellular signal transduction, cyclic nucleotide biosynthetic process, cGMP biosynthetic process, heme binding, phosphorus-oxygen lyase activity, guanylate cyclase activity
groupIX	9507731	9564731	Us	n	ENSGACG00000018013	PRSS12	proteolysis, membrane, catalytic activity, serine-type endopeptidase activity, scavenger receptor activity, proteolysis, membrane, catalytic activity, serine-type endopeptidase activity, scavenger receptor activity
groupIX	9507731	9564731	Us	n	ENSGACG00000018022	NDST4	DNA recombination, double-strand break repair via homologous recombination, regulation of S phase of mitotic cell cycle, nucleus, single-stranded DNA binding, sulfotransferase activity
groupIX	11478480	11511943	Us	psR	ENSGACG00000018405	C4orf33	-
groupIX	11478480	11511943	Us	psR	ENSGACG00000018401	SCLT1	-
groupIX	13623617	13650617	Us	psL	ENSGACG00000018609	CLTA	intracellular protein transport, vesicle-mediated transport, clathrin coat of trans-Golgi network vesicle, clathrin coat of coated pit, structural molecule activity
groupIX	13623617	13650617	Us	psL	ENSGACG00000018604	GNE	lipopolysaccharide biosynthetic process, UDP-N-acetylglucosamine metabolic process, UDP-N-acetylglucosamine 2-epimerase activity
groupV	841568	890568	Us	psL	ENSGACG00000002397	SCD5 (1 of 2)	oxidation-reduction process, fatty acid biosynthetic process, lipid metabolic process, membrane, endoplasmic reticulum, iron ion binding, oxidoreductase activity, stearoyl-CoA 9-desaturase activity
groupV	841568	890568	Us	psL	ENSGACG00000002420	NDUFB8	-
groupV	841568	890568	Us	psL	ENSGACG00000002427	-	oxidation-reduction process, response to oxidative stress, glutathione peroxidase activity
groupV	841568	890568	Us	psL	ENSGACG00000002428	FBXL15	dorsal/ventral pattern formation, protein binding, protein binding
groupV	841568	890568	Us	psL	ENSGACG00000002414	SEC31B	protein binding
groupV	4280394	4355314	Us	n	ENSGACG00000003772	ORMDL3	negative regulation of translation, integral to membrane, rRNA N-glycosylase activity
groupV	4280394	4355314	Us	n	ENSGACG00000003779	WNT3	multicellular organismal development, Wnt receptor signaling pathway, calcium modulating pathway, extracellular region
groupV	4280394	4355314	Us	n	ENSGACG00000003786	NSF (2 of 2)	ATP binding, binding, nucleotide binding, nucleoside-triphosphatase activity
groupV	4456555	4498330	Us	-	ENSGACG00000003822	TBKBP1 (1 of 2)	-
groupV	4722030	4788030	Us	n	ENSGACG00000004095	NDEL1 (1 of 2)	-
groupV	4722030	4788030	Us	n	ENSGACG00000004117	ARMC7	binding, protein binding
groupV	4722030	4788030	Us	n	ENSGACG00000004134	CHAD	protein binding
groupV	4722030	4788030	Us	n	ENSGACG00000004081	ARHGAP44 (1 of 2)	signal transduction, intracellular, cytoplasm, protein binding
groupV	4722030	4788030	Us	n	ENSGACG00000004091	C7orf62	-
groupV	4722030	4788030	Us	n	ENSGACG00000004121	ACSF2 (1 of 2)	metabolic process, catalytic activity
groupV	4722030	4788030	Us	n	ENSGACG00000004139	-	transport, transmembrane transport, integral to membrane, ATP binding, nucleotide binding, ATPase activity, ATPase activity, coupled to transmembrane movement of substances, nucleoside-triphosphatase activity
groupVI	5707587	5735587	Us	bg	-	-	-
groupVI	7679742	7726724	Us	psL	ENSGACG00000006266	BMP5	growth, growth factor activity
groupVI	7679742	7726724	Us	psL	ENSGACG00000006193	DST	signal transduction, cell cycle arrest, membrane, cytoskeleton, signal transducer activity, calcium ion binding, actin binding, protein binding
groupVI	7679742	7726724	Us	psL	ENSGACG00000006250	-	cell adhesion, protein binding, structural molecule activity
groupVII	6251601	6281614	Us	bg	-	-	-
groupVII	7475266	7500887	Us	n	ENSGACG00000019747	SNX15	cell communication, phosphatidylinositol binding, protein binding
groupVII	7475266	7500887	Us	n	ENSGACG00000019750	-	regulation of transcription, DNA-dependent, multicellular organismal development, lymphangiogenesis, nucleus, DNA binding, protein binding

groupVII	7475266	7500887	Us	n	ENSGACG00000019751	TMEM179B	-
groupVII	7475266	7500887	Us	n	ENSGACG00000019753	BRMS1	-
groupVII	7557638	7606333	Us	bg	ENSGACG00000019763	-	-
groupVII	7557638	7606333	Us	bg	ENSGACG00000019760	PLCB3	signal transduction, intracellular signal transduction, lipid metabolic process, embryonic viscerocranium morphogenesis, cartilage morphogenesis, calcium ion binding, protein binding, phospholipase C activity, phosphatidylinositol phospholipase C activity, phosphoric diester hydrolase activity
groupVII	7557638	7606333	Us	bg	ENSGACG00000019764	-	cell communication, calcium ion transport, transmembrane transport, carbon fixation, membrane, integral to membrane, plastid, magnesium ion binding, ribulose-bisphosphate carboxylase activity, calcium:sodium antiporter activity
groupVII	7707929	7759915	Us	psR	ENSGACG00000019812	ARL2	small GTPase mediated signal transduction, intracellular protein transport, protein transport, intracellular, GTP binding
groupVII	7707929	7759915	Us	psR	ENSGACG00000022730	-	-
groupVII	7707929	7759915	Us	psR	ENSGACG00000022390	-	-
groupVII	7707929	7759915	Us	psR	ENSGACG00000022786	-	-
groupVII	7707929	7759915	Us	psR	ENSGACG00000022650	-	-
groupVII	7707929	7759915	Us	psR	ENSGACG00000022602	-	-
groupVII	7707929	7759915	Us	psR	ENSGACG00000022446	-	-
groupVII	7707929	7759915	Us	psR	ENSGACG00000022711	-	-
groupVII	7707929	7759915	Us	psR	ENSGACG00000019819	-	-
groupVII	7707929	7759915	Us	psR	ENSGACG00000022330	-	-
groupVII	7707929	7759915	Us	psR	ENSGACG00000022420	-	-
groupVII	7707929	7759915	Us	psR	ENSGACG00000022678	-	-
groupVII	7707929	7759915	Us	psR	ENSGACG00000022377	-	-
groupVII	7707929	7759915	Us	psR	ENSGACG00000022735	-	-
groupVII	7707929	7759915	Us	psR	ENSGACG00000019824	B3GAT3	membrane, galactosylgalactosylxylosylprotein 3-beta-glucuronosyltransferase activity
groupVII	7707929	7759915	Us	psR	ENSGACG00000019803	PPP2R5B	signal transduction, protein phosphatase type 2A complex, binding, protein phosphatase type 2A regulator activity
groupVII	7903927	7940621	Us	psL	ENSGACG00000019877	C2orf65	-
groupVII	8088936	8224090	Us	psL	ENSGACG00000019896	RPGRIP1	protein binding
groupVII	8088936	8224090	Us	psL	ENSGACG00000019900	PCED1A	-
groupVII	8088936	8224090	Us	psL	ENSGACG00000019913	TDRD7 (1 of 2)	nucleic acid binding
groupVII	8088936	8224090	Us	psL	ENSGACG00000019917	CABP4 (2 of 2)	calcium ion binding
groupVII	8088936	8224090	Us	psL	ENSGACG00000019919	TMEM88 (1 of 2)	-
groupVII	8088936	8224090	Us	psL	ENSGACG00000019920	KDM6B (1 of 2)	protein binding
groupVII	8088936	8224090	Us	psL	ENSGACG00000019921	-	-
groupVII	8088936	8224090	Us	psL	ENSGACG00000019895	-	protein ubiquitination, intracellular, ubiquitin ligase complex, zinc ion binding, protein binding, ubiquitin-protein ligase activity
groupVII	8088936	8224090	Us	psL	ENSGACG00000019902	-	signal transduction, neuron fate commitment, retina morphogenesis in camera-type eye, establishment of nucleus localization, apical protein localization, retina layer formation, camera-type eye photoreceptor cell differentiation, intracellular
groupVII	8274759	8315759	Us	-	-	-	-
groupVII	10103089	10126089	Us	psR	ENSGACG00000020030	NRXN2 (2 of 3)	calcium ion binding, protein binding
groupVII	11932580	11954842	Us	bg	ENSGACG00000020129	-	protein binding, phosphotransferase activity, alcohol group as acceptor
groupVII	12481549	12555549	Us	bg	ENSGACG00000020160	SKA2L	-
groupVII	12481549	12555549	Us	bg	ENSGACG00000021295	-	-
groupVII	12481549	12555549	Us	bg	ENSGACG00000021437	-	-
groupVII	12481549	12555549	Us	bg	ENSGACG00000020161	TIPRL	-
groupVII	12481549	12555549	Us	bg	ENSGACG00000020158	SIM2	regulation of transcription, DNA-dependent, signal transduction, nucleus, signal transducer activity, protein binding
groupVII	12481549	12555549	Us	bg	ENSGACG00000020159	HLCS	cellular protein modification process, catalytic activity, biotin-[acetyl-CoA-carboxylase] ligase activity, cellular protein modification process, catalytic activity, biotin-[acetyl-CoA-carboxylase] ligase activity
groupVII	12919373	13023652	Us	n	ENSGACG00000020183	JAM3 (1 of 2)	protein binding
groupVII	12919373	13023652	Us	n	ENSGACG00000020184	IGSF9B (2 of 4)	-
groupVII	12919373	13023652	Us	n	ENSGACG00000020185	IGSF9B (1 of 4)	protein binding, protein binding

groupVII	13184192	13506953	Us	n	ENSGACG00000020189	IGSF5 (1 of 2)	protein binding
groupVII	13184192	13506953	Us	n	ENSGACG00000020190	-	transport, transmembrane transport, integral to membrane, transporter activity, transport, transmembrane transport, integral to membrane, transporter activity
groupVII	13184192	13506953	Us	n	ENSGACG00000020194	PRKRIR (2 of 3)	nucleic acid binding
groupVII	13184192	13506953	Us	n	ENSGACG00000020198	DGAT2	protein binding, transferase activity, transferring acyl groups other than amino-acyl groups
groupVII	13184192	13506953	Us	n	ENSGACG00000020200	MAP6 (1 of 2)	-
groupVII	13184192	13506953	Us	n	ENSGACG00000020201	UMODL1	-
groupVII	13184192	13506953	Us	n	ENSGACG00000020202	ZNF295	intracellular, DNA binding, zinc ion binding, protein binding, intracellular, DNA binding, zinc ion binding, protein binding
groupVII	13184192	13506953	Us	n	ENSGACG00000020191	-	membrane, galactosylgalactosylxylosylprotein 3-beta-glucuronosyltransferase activity
groupVII	13184192	13506953	Us	n	ENSGACG00000020192	-	carbohydrate metabolic process, beta-galactosidase complex, hydrolase activity, hydrolyzing O-glycosyl compounds, beta-galactosidase activity
groupVII	13184192	13506953	Us	n	ENSGACG00000020193	STT3A	protein glycosylation, membrane, oligosaccharyl transferase activity, protein glycosylation, membrane, oligosaccharyl transferase activity
groupVII	13184192	13506953	Us	n	ENSGACG00000020195	PRKRIR (3 of 3)	nucleic acid binding
groupVII	13184192	13506953	Us	n	ENSGACG00000020196	WNT11	multicellular organismal development, Wnt receptor signaling pathway, calcium modulating pathway, cell migration involved in gastrulation, heart development, extracellular region
groupVII	13184192	13506953	Us	n	ENSGACG00000020197	UVRAG	protein binding
groupVII	13184192	13506953	Us	n	ENSGACG00000020199	MOGAT2	protein binding, transferase activity, transferring acyl groups other than amino-acyl groups
groupVII	13622945	13639945	Us	psL	ENSGACG00000020210	-	-
groupVII	14206861	14253284	Us	n	ENSGACG00000020217	ATP5L2	ATP synthesis coupled proton transport, mitochondrial proton-transporting ATP synthase complex, coupling factor F(o), hydrogen ion transmembrane transporter activity
groupVII	14206861	14253284	Us	n	ENSGACG00000020218	-	vesicle-mediated transport, membrane, intracellular, zinc ion binding, protein binding
groupVII	14206861	14253284	Us	n	ENSGACG00000020220	RUVBL2	ATP binding, nucleotide binding, DNA helicase activity, nucleoside-triphosphatase activity, ATP catabolic process, regulation of heart growth, negative regulation of transcription, DNA-dependent, heart development, digestive tract development, ATP binding, nucleotide binding, DNA helicase activity, nucleoside-triphosphatase activity, ATPase activity
groupVII	14206861	14253284	Us	n	ENSGACG00000020216	CLTC	intracellular protein transport, vesicle-mediated transport, clathrin coat of trans-Golgi network vesicle, clathrin coat of coated pit, binding, structural molecule activity
groupVII	14206861	14253284	Us	n	ENSGACG00000020219	-	intracellular, zinc ion binding, protein binding, intracellular, zinc ion binding, protein binding, intracellular, zinc ion binding, protein binding, intracellular, zinc ion binding, protein binding, intracellular, zinc ion binding, protein binding, protein binding
groupVII	14720025	14776561	Us	psL	-	-	-
groupVII	14849644	14907551	Us	n	ENSGACG00000020242	-	-
groupVII	14849644	14907551	Us	n	ENSGACG00000020243	-	-
groupVII	14849644	14907551	Us	n	ENSGACG00000020244	ZNHIT3	-
groupVII	14849644	14907551	Us	n	ENSGACG00000020246	PIGW	GPI anchor biosynthetic process, integral to membrane, endoplasmic reticulum membrane, transferase activity, transferring acyl groups
groupVII	14849644	14907551	Us	n	ENSGACG00000020248	DHRS11	metabolic process, oxidoreductase activity
groupVII	14849644	14907551	Us	n	ENSGACG00000020241	GUSB	carbohydrate metabolic process, hydrolase activity, hydrolyzing O-glycosyl compounds, carbohydrate metabolic process, hydrolase activity, hydrolyzing O-glycosyl compounds, carbohydrate metabolic process, hydrolase activity, hydrolyzing O-glycosyl compounds
groupVII	14849644	14907551	Us	n	ENSGACG00000020245	MYO19	myosin complex, motor activity, ATP binding, nucleotide binding, protein binding, nucleoside-triphosphatase activity
groupVII	14849644	14907551	Us	n	ENSGACG00000020247	GGNBP2	-
groupVII	17035259	17069410	Us	psR	ENSGACG00000020328	TBC1D8B	-
groupVII	17035259	17069410	Us	psR	ENSGACG00000020327	AIFM1	oxidation-reduction process, oxidoreductase activity, flavin adenine dinucleotide binding, oxidation-reduction process, oxidoreductase activity, flavin adenine dinucleotide binding, oxidation-reduction process, oxidoreductase activity, flavin adenine dinucleotide binding, oxidation-reduction process, oxidoreductase activity, flavin adenine dinucleotide binding, oxidation-reduction process, oxidoreductase activity, flavin adenine dinucleotide binding
groupVII	17035259	17069410	Us	psR	ENSGACG00000020326	GLRA4 (2 of 2)	transport, ion transport, membrane, integral to membrane, postsynaptic membrane, extracellular ligand-gated ion channel activity, transport, ion transport, membrane, integral to membrane, postsynaptic membrane, extracellular ligand-gated ion channel activity
groupVII	17183496	17221578	Us	bg	ENSGACG00000020335	FAM123B	-

groupVII	17183496	17221578	Us	bg	ENSGACG00000020336	-	protein binding
groupVII	17183496	17221578	Us	bg	ENSGACG00000020338	NPAS2	photoperiodism, response to light stimulus, circadian rhythm, double-stranded DNA binding
groupVII	17183496	17221578	Us	bg	ENSGACG00000020337	CXorf57	-
groupVII	17329821	17346821	Us	psL	ENSGACG00000020350	RTN2 (2 of 2)	endoplasmic reticulum
groupVII	17329821	17346821	Us	psL	ENSGACG00000020351	PVRL3 (2 of 2)	protein binding, protein binding
groupVII	17403453	17488609	Us	psL	ENSGACG00000020359	PAFAH1B2	lipid metabolic process, hydrolase activity, acting on ester bonds
groupVII	17403453	17488609	Us	psL	ENSGACG00000020360	MPZL2 (2 of 2)	protein binding, identical protein binding
groupVII	17403453	17488609	Us	psL	ENSGACG00000020357	-	cell communication, connexon complex
groupVII	17403453	17488609	Us	psL	ENSGACG00000020358	TAGLN (2 of 2)	cell morphogenesis, actin cytoskeleton organization, actin cytoskeleton, protein binding, protein binding
groupVII	17403453	17488609	Us	psL	ENSGACG00000020361	SCN4B (2 of 2)	protein binding
groupVII	17403453	17488609	Us	psL	ENSGACG00000020362	DSCAML1 (1 of 2)	proteolysis, homophilic cell adhesion, membrane, calcium ion binding, zinc ion binding, protein binding, metallocarboxypeptidase activity
groupVII	17667176	17698176	Us	bg	ENSGACG00000020371	PLP1 (2 of 2)	integral to membrane, integral to membrane, integral to membrane
groupVII	17667176	17698176	Us	bg	ENSGACG00000020372	-	-
groupVII	17667176	17698176	Us	bg	ENSGACG00000020373	-	nucleic acid binding, DNA binding, protein binding
groupVII	17667176	17698176	Us	bg	ENSGACG00000020374	-	nuclear mRNA splicing, via spliceosome, spliceosomal complex
groupVII	17667176	17698176	Us	bg	ENSGACG00000020370	NLGN3 (2 of 2)	-
groupVII	17667176	17698176	Us	bg	ENSGACG00000020375	MTMR2	dephosphorylation, ATP binding, protein tyrosine phosphatase activity, phosphatase activity
groupVII	18497880	18543915	Us	psL	ENSGACG00000020433	SLC9A6 (2 of 2)	sodium ion transport, transmembrane transport, cation transport, regulation of pH, integral to membrane, sodium:hydrogen antiporter activity, solute:hydrogen antiporter activity
groupVII	18497880	18543915	Us	psL	ENSGACG00000020431	GPR112 (2 of 2)	G-protein coupled receptor signaling pathway, cell surface receptor signaling pathway, neuropeptide signaling pathway, membrane, integral to membrane, G-protein coupled receptor activity, transmembrane signaling receptor activity
groupVII	18497880	18543915	Us	psL	ENSGACG00000020432	FHL1 (2 of 2)	intracellular, zinc ion binding, intracellular, zinc ion binding
groupVII	18602309	18667488	Us	n	ENSGACG00000020442	CD248 (2 of 2)	binding
groupVII	18602309	18667488	Us	n	ENSGACG00000020443	RCE1 (2 of 2)	membrane
groupVII	18602309	18667488	Us	n	ENSGACG00000020439	-	protein binding, phospholipid binding
groupVII	18602309	18667488	Us	n	ENSGACG00000020441	DPP3	proteolysis, cytoplasm, dipeptidyl-peptidase activity
groupVII	18602309	18667488	Us	n	ENSGACG00000020444	EHBP1L1 (2 of 2)	protein binding,
groupVII	18602309	18667488	Us	n	ENSGACG00000020440	RBM14 (2 of 2)	nucleic acid binding
groupVII	18745383	18824089	Us	bg	ENSGACG00000020453	ADRB2 (2 of 2)	G-protein coupled receptor signaling pathway, integral to membrane
groupVII	18745383	18824089	Us	bg	ENSGACG00000020455	IL12B (2 of 3)	-
groupVII	18745383	18824089	Us	bg	ENSGACG00000020456	UBLCP1	nucleus, phosphoprotein phosphatase activity, protein binding
groupVII	18745383	18824089	Us	bg	ENSGACG00000020457	RNF145	zinc ion binding, protein binding
groupVII	18745383	18824089	Us	bg	ENSGACG00000020454	UBTD2	protein binding
groupVII	18745383	18824089	Us	bg	ENSGACG00000020452	ABLIM3 (2 of 2)	cytoskeleton organization, zinc ion binding, actin binding
groupVII	18745383	18824089	Us	bg	ENSGACG00000020458	EBF1 (1 of 2)	regulation of transcription, DNA-dependent, protein binding
groupVII	18937368	19046723	Us	psL	ENSGACG00000020460	BOD1	-
groupVII	18937368	19046723	Us	psL	ENSGACG00000020461	BNIP1 (2 of 2)	-
groupVII	18937368	19046723	Us	psL	ENSGACG00000020462	CREBRF	-
groupVII	18937368	19046723	Us	psL	ENSGACG00000020463	PCYOX1L	oxidation-reduction process, prenylcysteine catabolic process, oxidoreductase activity, oxidoreductase activity, acting on a sulfur group of donors, oxygen as acceptor
groupVII	18937368	19046723	Us	psL	ENSGACG00000020466	-	G-protein coupled receptor signaling pathway, visual perception, integral to membrane, G-protein coupled receptor signaling pathway, visual perception, integral to membrane
groupVII	18937368	19046723	Us	psL	ENSGACG00000020467	-	metabolic process, protein-tyrosine sulfotransferase activity,
groupVII	18937368	19046723	Us	psL	ENSGACG00000020468	CRCP	cellular metabolic process, catalytic activity, nucleotide binding
groupVII	18937368	19046723	Us	psL	ENSGACG00000020469	-	catalytic activity
groupVII	18937368	19046723	Us	psL	ENSGACG00000020459	HTATSF1	nucleic acid binding
groupVII	18937368	19046723	Us	psL	ENSGACG00000020464	TCERG1	protein binding, protein binding

groupVII	18937368	19046723	Us	psL	ENSGACG00000020470	ASL	arginine biosynthetic process via ornithine, catalytic activity, argininosuccinate lyase activity, arginine biosynthetic process via ornithine, catalytic activity, argininosuccinate lyase activity, arginine biosynthetic process via ornithine, catalytic activity, argininosuccinate lyase activity
groupVII	18937368	19046723	Us	psL	ENSGACG00000020465	CLINT1 (1 of 2)	-
groupVII	19053891	19098891	Us	psR	ENSGACG00000020472	-	oxidation-reduction process, metabolic process, oxidoreductase activity, oxidation-reduction process, metabolic process, oxidoreductase activity
groupVII	19053891	19098891	Us	psR	ENSGACG00000020474	SLC13A2 (2 of 2)	sodium ion transport, transmembrane transport, membrane, transporter activity
groupVII	19053891	19098891	Us	psR	ENSGACG00000020476	-	-
groupVII	19053891	19098891	Us	psR	ENSGACG00000020473	-	immune response, scavenger receptor activity, polysaccharide binding, immune response, scavenger receptor activity, polysaccharide binding
groupVII	19053891	19098891	Us	psR	ENSGACG00000020475	UNC119 (1 of 2)	-
groupVII	19242686	19285534	Us	psL	ENSGACG00000020493	GBAS (2 of 2)	metabolic process, transferase activity, folic acid binding
groupVII	19242686	19285534	Us	psL	ENSGACG00000020494	CCT6A	protein folding, cellular protein metabolic process, ATP binding, unfolded protein binding, protein folding, cellular protein metabolic process, ATP binding, unfolded protein binding
groupVII	19242686	19285534	Us	psL	ENSGACG00000022599	-	-
groupVII	19242686	19285534	Us	psL	ENSGACG00000022586	-	-
groupVII	19242686	19285534	Us	psL	ENSGACG00000022642	-	-
groupVII	19242686	19285534	Us	psL	ENSGACG00000020495	SUMF2	binding
groupVII	19242686	19285534	Us	psL	ENSGACG00000020496	-	-
groupVII	19242686	19285534	Us	psL	ENSGACG00000020497	ABHD11	intracellular protein transport, GPI anchor metabolic process, intrinsic to endoplasmic reticulum membrane, hydrolase activity, acting on ester bonds
groupVII	19242686	19285534	Us	psL	ENSGACG00000020498	-	membrane, tight junction, structural molecule activity
groupVII	19242686	19285534	Us	psL	ENSGACG00000020499	-	membrane, tight junction, structural molecule activity
groupVII	20218240	20333240	Us	n	ENSGACG00000020565	FAM48A	-
groupVII	20218240	20333240	Us	n	ENSGACG00000020564	EXOSC8	RNA processing, RNA binding
groupVII	20218240	20333240	Us	n	ENSGACG00000020566	-	metabolic process, transferase activity, transferring hexosyl groups
groupVII	20218240	20333240	Us	n	ENSGACG00000020567	POSTN (2 of 2)	protein binding,
groupVII	20218240	20333240	Us	n	ENSGACG00000020568	TRPC4 (1 of 2)	ion transport, transmembrane transport, translation, membrane, integral to membrane, intracellular, ribosome, protein binding, structural constituent of ribosome, ion channel activity, ion transport, transmembrane transport, translation, membrane, integral to membrane, intracellular, ribosome, protein binding, structural constituent of ribosome, ion channel activity
groupVII	20218240	20333240	Us	n	ENSGACG00000020569	FREM2 (2 of 2)	cell communication, homophilic cell adhesion, membrane, integral to membrane, calcium ion binding, cell communication, homophilic cell adhesion, membrane, integral to membrane, calcium ion binding
groupVII	20409809	20461876	Us	n	ENSGACG00000020572	ARFIP2 (1 of 2)	-
groupVII	20409809	20461876	Us	n	ENSGACG00000020573	DCHS1 (2 of 2)	cell adhesion, homophilic cell adhesion, membrane, plasma membrane, calcium ion binding, cell adhesion, homophilic cell adhesion, membrane, plasma membrane, calcium ion binding
groupVII	21391936	21524060	Us	n	ENSGACG00000020612	-	transmembrane transport, drug transmembrane transport, membrane, drug transmembrane transporter activity, antiporter activity, transmembrane transport, drug transmembrane transport, membrane, drug transmembrane transporter activity, antiporter activity
groupVII	21391936	21524060	Us	n	ENSGACG00000020613	-	transmembrane transport, drug transmembrane transport, membrane, drug transmembrane transporter activity, antiporter activity, transmembrane transport, drug transmembrane transport, membrane, drug transmembrane transporter activity, antiporter activity
groupVII	21391936	21524060	Us	n	ENSGACG00000020617	CCT8	protein folding, cellular protein metabolic process, ATP binding, unfolded protein binding, protein folding, cellular protein metabolic process, ATP binding, unfolded protein binding, protein folding, cellular protein metabolic process, behavioral response to nicotine, ATP binding, unfolded protein binding
groupVII	21391936	21524060	Us	n	ENSGACG00000020619	-	glycolysis, fructose-bisphosphate aldolase activity
groupVII	21391936	21524060	Us	n	ENSGACG00000020614	-	transmembrane transport, drug transmembrane transport, membrane, drug transmembrane transporter activity, antiporter activity
groupVII	21391936	21524060	Us	n	ENSGACG00000020615	-	transmembrane transport, drug transmembrane transport, membrane, drug transmembrane transporter activity, antiporter activity
groupVII	21391936	21524060	Us	n	ENSGACG00000020618	-	transferase activity, transferring phosphorus-containing groups
groupVII	21391936	21524060	Us	n	ENSGACG00000020616	TMIGD1	protein binding

groupVII	21632504	21657126	Us	n	ENSGACG00000020635	CHEK1	protein phosphorylation, ATP binding, protein kinase activity, transferase activity, transferring phosphorus-containing groups, protein serine/threonine kinase activity
groupVII	21632504	21657126	Us	n	ENSGACG00000020637	LAYN (1 of 2)	binding
groupVII	21632504	21657126	Us	n	ENSGACG00000020636	CFB	proteolysis, complement activation, extracellular region, catalytic activity, protein binding, serine-type endopeptidase activity, proteolysis, complement activation, extracellular region, catalytic activity, protein binding, serine-type endopeptidase activity
groupVIII	7922475	7989930	Us	psL	ENSGACG00000007122	RAVER2	nucleic acid binding
groupVIII	7922475	7989930	Us	psL	ENSGACG00000007145	JAK1	protein phosphorylation, cytoskeleton, ATP binding, protein kinase activity, protein binding, transferase activity, transferring phosphorus-containing groups, protein serine/threonine kinase activity, protein tyrosine kinase activity
groupVIII	8291548	8418958	Us	n	ENSGACG00000007306	-	-
groupVIII	8291548	8418958	Us	n	ENSGACG00000007278	-	extracellular region, protein binding, peptidase inhibitor activity
groupVIII	8291548	8418958	Us	n	ENSGACG00000007288	IVNS1ABP	protein binding
groupVIII	8291548	8418958	Us	n	ENSGACG00000007270	-	-
groupVIII	8291548	8418958	Us	n	ENSGACG00000007309	-	fin development, fin morphogenesis, basement membrane organization, calcium ion binding, protein binding
groupVIII	9769411	9784476	Us	n	ENSGACG00000008635	NDUFA7	ATP synthesis coupled electron transport, mitochondrial inner membrane, NADH dehydrogenase (ubiquinone) activity
groupVIII	9769411	9784476	Us	n	ENSGACG00000008636	-	calcium ion binding, protein binding
groupVIII	9769411	9784476	Us	n	ENSGACG00000008622	MAST3 (1 of 2)	protein phosphorylation, ATP binding, protein kinase activity, protein binding, transferase activity, transferring phosphorus-containing groups, protein serine/threonine kinase activity
groupVIII	11068543	11177201	Us	n	-	-	-
groupVIII	11301931	11362144	Us	n	ENSGACG00000009078	-	transport, ion transport, membrane, integral to membrane, postsynaptic membrane, extracellular ligand-gated ion channel activity
groupVIII	11301931	11362144	Us	n	ENSGACG00000009086	KIAA0226	zinc ion binding, protein binding
groupVIII	11301931	11362144	Us	n	ENSGACG00000009134	TSC22D2 (2 of 2)	regulation of transcription, DNA-dependent, sequence-specific DNA binding transcription factor activity
groupVIII	11846963	11881697	Us	-	-	-	-
groupVIII	11883508	11928508	Us	-	ENSGACG00000009483	NEGR1	protein binding, identical protein binding
groupX	9017140	9067690	Us	-	ENSGACG00000022581	-	-
groupX	9017140	9067690	Us	-	ENSGACG00000006358	GRN	-
groupX	9017140	9067690	Us	-	ENSGACG00000006347	FAM133B	-
groupX	9017140	9067690	Us	-	ENSGACG00000006364	ANKRD28 (2 of 2)	protein binding
groupX	9017140	9067690	Us	-	ENSGACG00000006323	CDK6	protein phosphorylation, ATP binding, protein kinase activity, transferase activity, transferring phosphorus-containing groups, protein serine/threonine kinase activity
groupXI	5544301	5561301	Us	-	ENSGACG00000008517	C1QL1 (1 of 2)	protein binding
groupXI	6838319	6877319	Us	n	ENSGACG00000009540	MPP3 (1 of 2)	protein binding
groupXI	6838319	6877319	Us	n	ENSGACG00000009558	ASIC2	sodium ion transport, membrane, integral to membrane, sodium channel activity, ligand-gated sodium channel activity
groupXI	7074675	7103089	Us	psR	ENSGACG00000009598	UBTF	DNA binding, protein binding
groupXI	7074675	7103089	Us	psR	ENSGACG00000009622	SLC4A1 (2 of 2)	transport, anion transport, embryonic hemopoiesis, erythrocyte differentiation, membrane, integral to membrane, transporter activity, inorganic anion exchanger activity, anion transmembrane transporter activity, protein binding
groupXI	7074675	7103089	Us	psR	ENSGACG00000009590	-	-
groupXI	7827045	7868045	Us	bg	ENSGACG00000010121	LPAR2 (2 of 2)	G-protein coupled receptor signaling pathway, angiogenesis, integral to membrane
groupXI	7827045	7868045	Us	bg	ENSGACG00000010106	BRD4	condensed chromosome, nucleus, protein binding, histone binding
groupXI	7827045	7868045	Us	bg	ENSGACG00000010133	-	regulation of transcription, DNA-dependent, nucleus, sequence-specific DNA binding transcription factor activity, sequence-specific DNA binding, protein binding
groupXI	8365974	8386113	Us	bg	ENSGACG00000010630	EPN3 (1 of 2)	-
groupXI	8365974	8386113	Us	bg	ENSGACG00000010637	FN3K	transferase activity, transferring phosphorus-containing groups
groupXI	8365974	8386113	Us	bg	ENSGACG00000010661	ZNF750	-
groupXI	8365974	8386113	Us	bg	ENSGACG00000010647	TBCD	binding, protein binding
groupXI	9419098	9484098	Us	psL	-	-	-
groupXI	10011875	10053875	Us	psR	ENSGACG00000011350	TRAP1	response to stress, protein folding, ATP binding, unfolded protein binding
groupXI	10011875	10053875	Us	psR	ENSGACG00000011359	FAM195A (2 of 2)	-
groupXI	10011875	10053875	Us	psR	ENSGACG00000011339	ADCY9 (2 of 2)	intracellular signal transduction, cyclic nucleotide biosynthetic process, phosphorus-oxygen lyase activity

groupXI	10011875	10053875	Us	psR	ENSGACG00000011341	CREBBP (1 of 2)	regulation of transcription, DNA-dependent, nucleus, transcription cofactor activity, zinc ion binding, protein binding, histone acetyltransferase activity, transcription coactivator activity
groupXII	2156021	2285239	Us	psR	ENSGACG00000003497	-	-
groupXII	2156021	2285239	Us	psR	ENSGACG00000003505	-	protein binding
groupXII	2156021	2285239	Us	psR	ENSGACG00000003535	OVGP1 (1 of 5)	carbohydrate metabolic process, chitin catabolic process, chitin metabolic process, extracellular region, hydrolase activity, hydrolyzing O-glycosyl compounds, chitinase activity, chitin binding
groupXII	2156021	2285239	Us	psR	ENSGACG00000003565	OVGP1 (4 of 5)	carbohydrate metabolic process, chitin catabolic process, chitin metabolic process, extracellular region, hydrolase activity, hydrolyzing O-glycosyl compounds, chitinase activity, chitin binding
groupXII	2156021	2285239	Us	psR	ENSGACG00000003501	-	metabolic process, lipid metabolic process
groupXII	2156021	2285239	Us	psR	ENSGACG00000003580	DENND2D	-
groupXII	2156021	2285239	Us	psR	ENSGACG00000003596	STK38	protein phosphorylation, ATP binding, protein kinase activity, transferase activity, transferring phosphorus-containing groups, protein serine/threonine kinase activity
groupXII	2156021	2285239	Us	psR	ENSGACG00000003602	MTCH1	-
groupXII	2285374	2600392	Us	psL	ENSGACG00000003612	-	-
groupXII	2285374	2600392	Us	psL	ENSGACG00000003613	STK35 (2 of 2)	protein phosphorylation, ATP binding, protein kinase activity, transferase activity, transferring phosphorus-containing groups, protein serine/threonine kinase activity
groupXII	2285374	2600392	Us	psL	ENSGACG00000003616	PDYN	-
groupXII	2285374	2600392	Us	psL	ENSGACG00000003620	GMEB2	nucleus, DNA binding, binding
groupXII	2285374	2600392	Us	psL	ENSGACG00000003625	-	hydrolase activity
groupXII	2285374	2600392	Us	psL	ENSGACG00000003630	-	signal transduction, GTP catabolic process, small GTPase mediated signal transduction, intracellular protein transport, nucleocytoplasmic transport, protein transport, intracellular, GTPase activity, GTP binding
groupXII	2285374	2600392	Us	psL	ENSGACG00000021304	-	-
groupXII	2285374	2600392	Us	psL	ENSGACG00000022313	-	-
groupXII	2285374	2600392	Us	psL	ENSGACG00000003654	C20orf151	-
groupXII	2285374	2600392	Us	psL	ENSGACG00000003657	-	-
groupXII	2285374	2600392	Us	psL	ENSGACG00000003649	GATA5	regulation of transcription, DNA-dependent, positive regulation of transcription, DNA-dependent, endoderm formation, pancreas development, myeloid cell differentiation, heart morphogenesis, Kupffer's vesicle development, cardioblast migration to the midline involved in heart field formation, left/right pattern formation, thyroid gland development, thymus development, post-embryonic foregut morphogenesis, positive regulation of transcription from RNA polymerase II promoter, heart development, endoderm development, liver development, nucleus, sequence-specific DNA binding transcription factor activity, DNA binding, zinc ion binding, sequence-specific DNA binding, protein binding
groupXII	10925827	10944827	Us	psL	ENSGACG00000008823	-	protein binding
groupXII	11497511	11518051	Us	psR	ENSGACG00000009372	RARG (1 of 2)	regulation of transcription, DNA-dependent, steroid hormone mediated signaling pathway, pharyngeal system development, nucleus, sequence-specific DNA binding transcription factor activity, steroid hormone receptor activity, zinc ion binding, sequence-specific DNA binding
groupXII	12140576	12160162	Us	bg	ENSGACG00000009758	TEAD3 (2 of 2)	regulation of transcription, DNA-dependent, nucleus, sequence-specific DNA binding transcription factor activity, protein binding
groupXII	13737599	13834163	Us	n	ENSGACG00000010842	ANKRD52 (2 of 2)	protein binding
groupXII	13737599	13834163	Us	n	ENSGACG00000010849	COQ10A	-
groupXII	13737599	13834163	Us	n	ENSGACG00000010851	CS	cellular carbohydrate metabolic process, tricarboxylic acid cycle, transferase activity, transferring acyl groups, acyl groups converted into alkyl on transfer, citrate (Si)-synthase activity
groupXII	13737599	13834163	Us	n	ENSGACG00000010885	-	-
groupXII	13737599	13834163	Us	n	ENSGACG00000010890	-	cytoplasm, protein binding
groupXII	13737599	13834163	Us	n	ENSGACG00000010906	DAZAP2	-
groupXII	13737599	13834163	Us	n	ENSGACG00000010925	POU6F1	-
groupXII	13737599	13834163	Us	n	ENSGACG00000010929	TFCP2	protein binding
groupXII	13737599	13834163	Us	n	ENSGACG00000010877	-	proteolysis, membrane, serine-type peptidase activity
groupXII	13737599	13834163	Us	n	ENSGACG00000010902	-	-
groupXIII	7972287	8022580	Us	n	ENSGACG00000007973	FOXB2	-

groupXIII	7972287	8022580	Us	n	ENSGACG00000007980	-	-
groupXIII	7972287	8022580	Us	n	ENSGACG00000007925	VPS13A	protein localization
groupXIII	9202236	9231236	Us	psR	ENSGACG00000009215	FOXN4	regulation of transcription, DNA-dependent, regulation of heart contraction, positive regulation of transcription, DNA-dependent, sequence-specific DNA binding transcription factor activity, sequence-specific DNA binding
groupXIII	9202236	9231236	Us	psR	ENSGACG00000009222	ACACB	metabolic process, fatty acid biosynthetic process, ATP binding, catalytic activity, ligase activity, acetyl-CoA carboxylase activity
groupXIII	9202236	9231236	Us	psR	ENSGACG00000009265	POLE	-
groupXIII	10308749	10336228	Us	n	ENSGACG00000010245	CCNH	regulation of cyclin-dependent protein kinase activity, holo TFIIH complex, protein kinase binding
groupXIII	10611632	10629632	Us	n	ENSGACG00000010281	-	small GTPase mediated signal transduction, protein transport, intracellular, zinc ion binding, GTP binding, protein binding
groupXIII	11509924	11555132	Us	psR	ENSGACG00000010571	MBLAC2	hydrolase activity
groupXIII	11509924	11555132	Us	psR	ENSGACG00000010576	-	signal transduction, intracellular, protein binding
groupXIII	11837144	11920602	Us	n	ENSGACG00000010750	-	-
groupXIII	11837144	11920602	Us	n	ENSGACG00000010753	-	-
groupXIII	11837144	11920602	Us	n	ENSGACG00000010756	ENTPD4	hydrolase activity
groupXIII	11837144	11920602	Us	n	ENSGACG00000010776	NKX3-1	regulation of transcription, DNA-dependent, nucleus, sequence-specific DNA binding transcription factor activity, sequence-specific DNA binding, protein binding
groupXIII	11837144	11920602	Us	n	ENSGACG00000010780	NKX2-6	regulation of transcription, DNA-dependent, embryonic heart tube development, heart looping, heart morphogenesis, cardiac muscle cell proliferation, cardiac muscle cell differentiation, nucleus, sequence-specific DNA binding transcription factor activity, sequence-specific DNA binding, protein binding
groupXIII	11837144	11920602	Us	n	ENSGACG00000010716	GDA	hydrolase activity
groupXIII	11837144	11920602	Us	n	ENSGACG00000010765	SLC25A37	-
groupXIII	11837144	11920602	Us	n	ENSGACG00000010784	STC1 (1 of 2)	extracellular region, ATP binding, hormone activity
groupXIII	12771187	12838733	Us	psR	ENSGACG00000011263	SDS	metabolic process, cellular amino acid metabolic process, catalytic activity, pyridoxal phosphate binding
groupXIII	12771187	12838733	Us	psR	ENSGACG00000011293	C12orf51 (2 of 2)	-
groupXIII	12771187	12838733	Us	psR	ENSGACG00000011278	C12orf51 (1 of 2)	cellular protein modification process, intracellular, acid-amino acid ligase activity
groupXIII	12983355	13029382	Us	psL	ENSGACG00000011510	TMC2 (2 of 2)	integral to membrane
groupXIII	12983355	13029382	Us	psL	ENSGACG00000011514	-	iron ion binding, electron carrier activity, heme binding, oxidoreductase activity, acting on paired donors, with incorporation or reduction of molecular oxygen
groupXIII	12983355	13029382	Us	psL	ENSGACG00000011501	TMC1	integral to membrane
groupXIII	12983355	13029382	Us	psL	ENSGACG00000011546	ANXA1 (1 of 2)	calcium ion binding, calcium-dependent phospholipid binding
groupXIII	13355139	13427412	Us	psL	ENSGACG00000011927	DGCR6L	-
groupXIII	13355139	13427412	Us	psL	ENSGACG00000011948	SLC20A1 (2 of 2)	phosphate ion transport, membrane, binding, inorganic phosphate transmembrane transporter activity
groupXIII	13355139	13427412	Us	psL	ENSGACG00000011961	SYN1	-
groupXIII	13355139	13427412	Us	psL	ENSGACG00000011938	DTX1	zinc ion binding, protein binding
groupXIII	13355139	13427412	Us	psL	ENSGACG00000011954	-	-
groupXIII	13355139	13427412	Us	psL	ENSGACG00000011932	RASAL1	signal transduction, intracellular signal transduction, regulation of small GTPase mediated signal transduction, intracellular, protein binding, GTPase activator activity
groupXV	12655665	12693614	Us	psL	-	-	-
groupXVI	5959507	6055021	Us	psL	-	-	-
groupXVI	9490632	9558899	Us	n	ENSGACG00000004299	STK24 (1 of 2)	protein phosphorylation, ATP binding, protein kinase activity, transferase activity, transferring phosphorus-containing groups, protein serine/threonine kinase activity
groupXVI	9490632	9558899	Us	n	ENSGACG00000004314	INO80D	-
groupXVI	9490632	9558899	Us	n	ENSGACG00000004237	DOCK9 (1 of 2)	GTP binding, protein binding, guanyl-nucleotide exchange factor activity, GTPase binding
groupXVI	9490632	9558899	Us	n	ENSGACG00000004265	SLC15A1 (2 of 2)	oligopeptide transport, membrane, transporter activity
groupXVI	10646828	10709828	Us	n	ENSGACG00000005385	GALNT3	-
groupXVI	10646828	10709828	Us	n	ENSGACG00000005428	TTC21B	protein binding, identical protein binding
groupXVI	10646828	10709828	Us	n	ENSGACG00000005450	-	ion transport, sodium ion transport, transmembrane transport, membrane, voltage-gated sodium channel complex, ion channel activity, voltage-gated sodium channel activity
groupXVI	10646828	10709828	Us	n	ENSGACG00000005466	SLC38A11	-

groupXVI	10646828	10709828	Us	n	ENSGACG00000005477	COBLL1	-
groupXVI	10646828	10709828	Us	n	ENSGACG00000005482	-	-
groupXVI	12597737	12610737	Us	n	ENSGACG00000006497	ERBB4 (1 of 2)	protein phosphorylation, transmembrane receptor protein tyrosine kinase signaling pathway, membrane, ATP binding, protein kinase activity, transferase activity, transferring phosphorus-containing groups, protein serine/threonine kinase activity, transmembrane receptor protein tyrosine kinase activity, protein tyrosine kinase activity
groupXVI	12635047	12651047	Us	bg	ENSGACG00000006497	ERBB4 (1 of 2)	protein phosphorylation, transmembrane receptor protein tyrosine kinase signaling pathway, membrane, ATP binding, protein kinase activity, transferase activity, transferring phosphorus-containing groups, protein serine/threonine kinase activity, transmembrane receptor protein tyrosine kinase activity, protein tyrosine kinase activity
groupXVI	12704546	12733874	Us	bg	ENSGACG00000006497	ERBB4 (1 of 2)	protein phosphorylation, transmembrane receptor protein tyrosine kinase signaling pathway, membrane, ATP binding, protein kinase activity, transferase activity, transferring phosphorus-containing groups, protein serine/threonine kinase activity, transmembrane receptor protein tyrosine kinase activity, protein tyrosine kinase activity
groupXVIII	3818080	3896352	Us	n	ENSGACG00000006261	-	-
groupXVIII	3818080	3896352	Us	n	ENSGACG00000006268	EPHA7	protein phosphorylation, transmembrane receptor protein tyrosine kinase signaling pathway, membrane, integral to membrane, ATP binding, protein kinase activity, protein binding, transferase activity, transferring phosphorus-containing groups, protein serine/threonine kinase activity, ephrin receptor activity, protein tyrosine kinase activity
groupXVIII	6203256	6272378	Us	psR	ENSGACG00000007328	HADHB	metabolic process, catalytic activity, transferase activity, transferring acyl groups other than amino-acyl groups
groupXVIII	6203256	6272378	Us	psR	ENSGACG00000007459	FAM59B	protein binding
groupXVIII	8454028	8471217	Us	psR	ENSGACG00000008882	CEP57L1	-
groupXVIII	8454028	8471217	Us	psR	ENSGACG00000008895	SESN1	cell cycle arrest, determination of left/right symmetry, nucleus
groupXX	6203653	6226724	Us	bg	ENSGACG00000006438	GRB10 (1 of 2)	signal transduction, protein binding
groupXX	6232759	6350493	Us	n	ENSGACG00000006455	-	G-protein coupled receptor signaling pathway, integral to membrane
groupXX	6232759	6350493	Us	n	ENSGACG00000006459	-	G-protein coupled receptor signaling pathway, integral to membrane, G-protein coupled receptor signaling pathway, integral to membrane
groupXX	6232759	6350493	Us	n	ENSGACG00000006438	GRB10 (1 of 2)	signal transduction, protein binding
groupXX	6232759	6350493	Us	n	ENSGACG00000006446	COBL	protein binding
groupXX	6232759	6350493	Us	n	ENSGACG00000006457	-	G-protein coupled receptor signaling pathway, integral to membrane
groupXX	7076535	7141289	Us	psL	-	-	-
groupXX	7360920	7380920	Us	n	ENSGACG00000006622	CDKAL1	RNA modification, macromolecule modification, cellular component, transferase activity, catalytic activity, 4 iron, 4 sulfur cluster binding, iron-sulfur cluster binding
groupXX	7784420	7808660	Us	-	ENSGACG00000006955	ZFAT	DNA repair, intracellular, DNA binding, zinc ion binding, protein binding
groupXX	7784420	7808660	Us	-	ENSGACG00000006970	PTP4A3	protein dephosphorylation, dephosphorylation, protein tyrosine phosphatase activity, protein tyrosine/serine/threonine phosphatase activity, phosphatase activity
groupXX	7784420	7808660	Us	-	ENSGACG00000006944	MTBP	-
groupXX	7879192	7898192	Us	bg	ENSGACG00000007045	-	membrane, cytoskeletal protein binding
groupXX	7879192	7898192	Us	bg	ENSGACG00000007035	MRS2	transmembrane transport, metal ion transport, membrane, metal ion transmembrane transporter activity
groupXX	7879192	7898192	Us	bg	ENSGACG00000007040	-	cell adhesion, cell-matrix adhesion, integrin-mediated signaling pathway, integrin complex, receptor activity, binding
groupXX	7879192	7898192	Us	bg	ENSGACG00000007051	CPQ	proteolysis, metabolic process, peptidase activity, hydrolase activity
groupXX	7974519	8100519	Us	psL	ENSGACG00000007079	-	-
groupXX	7974519	8100519	Us	psL	ENSGACG00000007130	CDCP1 (3 of 3)	-
groupXX	7974519	8100519	Us	psL	ENSGACG00000007136	CLEC3B (1 of 2)	binding
groupXX	7974519	8100519	Us	psL	ENSGACG00000007151	UBXN11	protein binding
groupXX	7974519	8100519	Us	psL	ENSGACG00000007065	-	cell adhesion, protein binding, structural molecule activity
groupXX	7974519	8100519	Us	psL	ENSGACG00000007081	DEPTOR	intracellular signal transduction, protein binding
groupXX	7974519	8100519	Us	psL	ENSGACG00000007087	LIMD1	intracellular signal transduction, zinc ion binding
groupXX	7974519	8100519	Us	psL	ENSGACG00000007099	LARS2	translation, tRNA aminoacylation for protein translation, leucyl-tRNA aminoacylation, cytoplasm, ATP binding, nucleotide binding, aminoacyl-tRNA ligase activity, leucine-tRNA ligase activity
groupXX	7974519	8100519	Us	psL	ENSGACG00000007141	FEZ2 (2 of 2)	-
groupXX	8182914	8236005	Us	n	ENSGACG00000007269	FAM46B (1 of 2)	-

groupXX	8182914	8236005	Us	n	ENSGACG00000007286	TCEA3	transcription, DNA-dependent, regulation of transcription, DNA-dependent, regulation of transcription elongation, DNA-dependent, transcription elongation, DNA-dependent, regulation of transcription from RNA polymerase II promoter, nucleus, nucleic acid binding, DNA binding, zinc ion binding, translation elongation factor activity
groupXX	8182914	8236005	Us	n	ENSGACG00000007300	OPRD1 (1 of 2)	G-protein coupled receptor signaling pathway, integral to membrane
groupXX	8182914	8236005	Us	n	ENSGACG00000007316	RCC1	-
groupXX	8182914	8236005	Us	n	ENSGACG00000007346	PHACTR4 (1 of 2)	regulation of transcription, DNA-dependent, transcription initiation, DNA-dependent, sequence-specific DNA binding transcription factor activity, DNA binding, sigma factor activity
groupXX	9654453	9719667	Us	-	ENSGACG00000008216	JAZF1 (1 of 2)	cilium assembly, determination of left/right symmetry, intracellular, zinc ion binding
groupXX	9654453	9719667	Us	-	ENSGACG00000008229	HIBADH (2 of 2)	oxidation-reduction process, valine metabolic process, pentose-phosphate shunt, NAD binding, 3-hydroxyisobutyrate dehydrogenase activity, phosphogluconate dehydrogenase (decarboxylating) activity
groupXX	9654453	9719667	Us	-	ENSGACG00000008296	HOXA13 (2 of 2)	regulation of transcription, DNA-dependent, negative regulation of transcription, DNA-dependent, nucleus, sequence-specific DNA binding transcription factor activity, sequence-specific DNA binding, protein binding, nucleic acid binding transcription factor activity
groupXX	9654453	9719667	Us	-	ENSGACG00000008297	HOXA11 (2 of 2)	regulation of transcription, DNA-dependent, nucleus, sequence-specific DNA binding transcription factor activity, sequence-specific DNA binding, protein binding
groupXX	9654453	9719667	Us	-	ENSGACG00000008212	CREB5	regulation of transcription, DNA-dependent, intracellular, sequence-specific DNA binding transcription factor activity, DNA binding, zinc ion binding, sequence-specific DNA binding, protein dimerization activity
groupXX	9654453	9719667	Us	-	ENSGACG00000008221	TAX1BP1 (1 of 2)	-
groupXX	12153275	12175313	Us	psL	ENSGACG00000010639	CACNG8 (2 of 2)	membrane
groupXX	12153275	12175313	Us	psL	ENSGACG00000010641	CACNG7 (2 of 2)	membrane
groupXXI	1429423	1539933	Us	n	-	-	-
groupXXI	1624705	1807705	Us	n	ENSGACG00000022561	-	-
groupXXI	1624705	1807705	Us	n	ENSGACG00000001786	-	glycolysis, cytoplasm, 6-phosphofructokinase complex, 6-phosphofructokinase activity
groupXXI	1956117	2132709	Us	n	ENSGACG00000001871	SNX16	cell communication, phosphatidylinositol binding, protein binding, cell communication, phosphatidylinositol binding, protein binding
groupXXI	1956117	2132709	Us	n	ENSGACG00000001881	SERPINB2 (1 of 2)	-
groupXXI	1956117	2132709	Us	n	ENSGACG00000001884	SERPINB2 (2 of 2)	-
groupXXI	1956117	2132709	Us	n	ENSGACG00000001917	PIGM	GPI anchor biosynthetic process, integral to membrane, endoplasmic reticulum membrane, transferase activity, transferring hexosyl groups
groupXXI	1956117	2132709	Us	n	ENSGACG00000001930	ANKS6	protein binding
groupXXI	1956117	2132709	Us	n	ENSGACG00000001940	RPS20	translation, intracellular, ribosome, small ribosomal subunit, RNA binding, structural constituent of ribosome, translation, intracellular, ribosome, small ribosomal subunit, RNA binding, structural constituent of ribosome
groupXXI	1956117	2132709	Us	n	ENSGACG00000022779	-	-
groupXXI	1956117	2132709	Us	n	ENSGACG00000001956	-	-
groupXXI	1956117	2132709	Us	n	ENSGACG00000001958	CCDC90A	-
groupXXI	1956117	2132709	Us	n	ENSGACG00000001961	DPH2	peptidyl-diphthamide biosynthetic process from peptidyl-histidine, cytoplasm
groupXXI	1956117	2132709	Us	n	ENSGACG00000001969	FAM59A	protein binding
groupXXI	1956117	2132709	Us	n	ENSGACG00000001925	-	regulation of transcription, DNA-dependent, signal transduction, response to chemical stimulus, regulation of transcription from RNA polymerase II promoter, nucleus, signal transducer activity
groupXXI	1956117	2132709	Us	n	ENSGACG00000001949	-	microtubule-based movement, ATP binding, microtubule motor activity
groupXXI	1956117	2132709	Us	n	ENSGACG00000001966	NCF2	protein binding
groupXXI	1956117	2132709	Us	n	ENSGACG00000001974	PRKDC	double-strand break repair via nonhomologous end joining, nucleus, DNA binding, ATP binding, binding, protein binding, transferase activity, transferring phosphorus-containing groups, phosphotransferase activity, alcohol group as acceptor, DNA-dependent protein kinase activity,
groupXXI	2724804	2807804	Us	psL	ENSGACG00000002116	NSMAF	protein binding
groupXXI	2724804	2807804	Us	psL	ENSGACG00000002128	CNGB3	ion transport, transmembrane transport, membrane, ion channel activity
groupXXI	4046432	4096479	Us	n	-	-	-
groupXXI	4171481	4221729	Us	-	-	-	-
groupXXI	4335682	4357200	Us	psR	ENSGACG00000002369	TMEM56	integral to membrane, integral to membrane

groupXXI	4335682	4357200	Us	psR	ENSGACG00000002374	BCMO1 (1 of 2)	eye photoreceptor cell differentiation, hindbrain development, oxidation-reduction process, beta-carotene 15, 15'-monooxygenase activity
groupXXI	4335682	4357200	Us	psR	ENSGACG00000002373	-	-
groupXXI	4519973	4555973	Us	psR	ENSGACG00000002390	CTNND2 (2 of 2)	binding, protein binding
groupXXI	6063085	6133269	Us	n	ENSGACG00000002670	SEMA5A	proteolysis, axon extension, membrane, catalytic activity, protein binding, cysteine-type endopeptidase activity
groupXXI	6485712	6521712	Us	psL	ENSGACG00000002723	PI15	extracellular region
groupXXI	6485712	6521712	Us	psL	ENSGACG00000002714	CRISPLD1 (1 of 2)	extracellular region
groupI	4960585	5104133	Ca	psR	ENSGACG00000007181	SI	carbohydrate metabolic process, methylation, nucleic acid binding, hydrolase activity, hydrolyzing O-glycosyl compounds, methyltransferase activity
groupI	5170476	5204551	Ca	psR	ENSGACG00000007230	-	-
groupI	8787521	8839086	Ca	n	ENSGACG00000009256	SHOX2	regulation of transcription, DNA-dependent, heart development, nucleus, sequence-specific DNA binding transcription factor activity, sequence-specific DNA binding, protein binding
groupI	8787521	8839086	Ca	n	ENSGACG00000009247	RSRC1	-
groupI	13577265	13647013	Ca	-	ENSGACG00000011579	DCLK1 (2 of 2)	intracellular signal transduction, protein phosphorylation, ATP binding, protein kinase activity, transferase activity, transferring phosphorus-containing groups, protein serine/threonine kinase activity
groupI	13577265	13647013	Ca	-	ENSGACG00000011555	NBEA (1 of 2)	protein binding, transferase activity, transferring phosphorus-containing groups
groupI	13577265	13647013	Ca	-	ENSGACG00000011596	SPG20 (1 of 2)	oxidation-reduction process, metabolic process, oxidoreductase activity
groupI	13929521	13988926	Ca	psL	ENSGACG00000011744	-	membrane, tight junction, structural molecule activity
groupI	13929521	13988926	Ca	psL	ENSGACG00000011753	-	membrane, tight junction, structural molecule activity
groupI	13929521	13988926	Ca	psL	ENSGACG00000011755	GRIK1 (1 of 2)	membrane, ionotropic glutamate receptor activity, extracellular-glutamate-gated ion channel activity
groupI	13929521	13988926	Ca	psL	ENSGACG00000011798	BACH1	regulation of transcription, DNA-dependent, sequence-specific DNA binding transcription factor activity, DNA binding, sequence-specific DNA binding, protein binding, protein dimerization activity
groupI	13929521	13988926	Ca	psL	ENSGACG00000011804	USP16	ubiquitin-dependent protein catabolic process, zinc ion binding, ubiquitin thiolesterase activity
groupI	13929521	13988926	Ca	psL	ENSGACG00000011812	RWDD2B	protein binding
groupI	13929521	13988926	Ca	psL	ENSGACG00000011816	CADM2 (1 of 2)	protein binding
groupI	16775583	16826316	Ca	psR	ENSGACG00000013006	NAPA	intracellular protein transport
groupI	16775583	16826316	Ca	psR	ENSGACG00000013015	GLTSCR1	-
groupI	16775583	16826316	Ca	psR	ENSGACG00000013042	NUP88	-
groupI	16775583	16826316	Ca	psR	ENSGACG00000013044	PSMD8	-
groupI	16775583	16826316	Ca	psR	ENSGACG00000013023	EHD2 (1 of 2)	calcium ion binding, GTPase activity, GTP binding, protein binding
groupI	16775583	16826316	Ca	psR	ENSGACG00000013083	SPRED3	-
groupI	16775583	16826316	Ca	psR	ENSGACG00000012993	BCKDHA	metabolic process, oxidoreductase activity, acting on the aldehyde or oxo group of donors, disulfide as acceptor
groupI	16833535	16861190	Ca	psR	ENSGACG00000013085	DHX34	nuclear-transcribed mRNA catabolic process, nonsense-mediated decay, embryo development, nucleic acid binding, ATP binding, electron carrier activity, metal ion binding, nucleotide binding, helicase activity, nucleoside-triphosphatase activity
groupI	16833535	16861190	Ca	psR	ENSGACG00000013091	ST14 (1 of 3)	proteolysis, epidermis development, catalytic activity, protein binding, serine-type endopeptidase activity
groupI	16833535	16861190	Ca	psR	ENSGACG00000013115	APLP2	integral to membrane, binding, serine-type endopeptidase inhibitor activity,
groupI	21236349	21288020	Ca	n	ENSGACG00000014230	-	-
groupI	22937800	22953890	Ca	n	ENSGACG00000014843	ARHGEF7 (2 of 2)	regulation of Rho protein signal transduction, blood vessel development, epiboly involved in gastrulation with mouth forming second, protein localization to adherens junction, intracellular, protein binding, Rho guanyl-nucleotide exchange factor activity
groupI	22956267	22966438	Ca	n	-	-	-
groupI	23001251	23034127	Ca	psR	ENSGACG00000014851	ATP11A (1 of 2)	ATP biosynthetic process, phospholipid transport, membrane, integral to membrane, magnesium ion binding, ATP binding, ATPase activity, coupled to transmembrane movement of ions, phosphorylative mechanism, phospholipid-translocating ATPase activity
groupI	23305198	23335911	Ca	psL	ENSGACG00000014926	ZRANB3	intracellular, nucleic acid binding, DNA binding, zinc ion binding, ATP binding, endonuclease activity, helicase activity
groupI	23722574	23783973	Ca	psL	ENSGACG00000015007	NDFIP2	-
groupII	5842830	5903218	Ca	psR	ENSGACG00000015040	-	-
groupII	5842830	5903218	Ca	psR	ENSGACG00000022753	-	-
groupII	5842830	5903218	Ca	psR	ENSGACG00000022812	-	-
groupII	5842830	5903218	Ca	psR	ENSGACG00000015047	WHAMM	-

groupII	5842830	5903218	Ca	psR	ENSGACG00000015051	HOMER2	protein binding
groupII	5842830	5903218	Ca	psR	ENSGACG00000015037	-	regulation of transcription, DNA-dependent, signal transduction, two-component signal transduction system (phosphorelay), signal transducer activity, catalytic activity, two-component response regulator activity, 3', 5'-cyclic-nucleotide phosphodiesterase activity, phosphoric diester hydrolase activity
groupII	5842830	5903218	Ca	psR	ENSGACG00000015044	FSD2	protein binding
groupII	6738132	6768135	Ca	psR	-	-	-
groupII	7093099	7200100	Ca	n	ENSGACG00000015262	PCDH20	cell adhesion, homophilic cell adhesion, membrane, plasma membrane, calcium ion binding
groupII	7093099	7200100	Ca	n	ENSGACG00000022515	-	-
groupII	7093099	7200100	Ca	n	ENSGACG00000015264	TDRD3	protein binding, mRNA processing, nucleus, cytoplasm, nucleic acid binding, RNA binding, protein binding, phosphotransferase activity, alcohol group as acceptor
groupII	7093099	7200100	Ca	n	ENSGACG00000015270	DIAPH3	actin cytoskeleton organization, cellular component organization, binding, actin binding, Rho GTPase binding
groupII	7635067	7711452	Ca	n	ENSGACG00000015283	BLZF1	-
groupII	7635067	7711452	Ca	n	ENSGACG00000015292	ERCC5	DNA repair, DNA binding, catalytic activity, nuclease activity, hydrolase activity, acting on ester bonds
groupII	7635067	7711452	Ca	n	ENSGACG00000015294	-	-
groupII	7635067	7711452	Ca	n	ENSGACG00000015297	METTL21C (2 of 2)	-
groupII	7635067	7711452	Ca	n	ENSGACG00000015298	-	-
groupII	7635067	7711452	Ca	n	ENSGACG00000015305	PROS1	extracellular region, calcium ion binding, protein binding
groupII	7635067	7711452	Ca	n	ENSGACG00000015281	RG9MTD1	-
groupII	7635067	7711452	Ca	n	ENSGACG00000015300	TPP2	proteolysis, serine-type endopeptidase activity
groupII	7635067	7711452	Ca	n	ENSGACG00000015302	RASA3	signal transduction, intracellular signal transduction, regulation of small GTPase mediated signal transduction, intracellular, protein binding, GTPase activator activity
groupII	8123901	8197571	Ca	n	ENSGACG00000015376	KCTD12	potassium ion transport, membrane, voltage-gated potassium channel complex, protein binding, voltage-gated potassium channel activity
groupII	8123901	8197571	Ca	n	ENSGACG00000015378	SLC5A7 (2 of 2)	transport, transmembrane transport, membrane, transporter activity
groupII	8123901	8197571	Ca	n	ENSGACG00000015382	ARL6	small GTPase mediated signal transduction, intracellular protein transport, protein transport, cilium assembly, melanosome transport, Kupffer's vesicle development, determination of left/right symmetry, pectoral fin development, intracellular, GTP binding
groupII	8123901	8197571	Ca	n	ENSGACG00000015384	EPHA6 (2 of 2)	protein phosphorylation, transmembrane receptor protein tyrosine kinase signaling pathway, membrane, integral to membrane, ATP binding, protein kinase activity, protein binding, transferase activity, transferring phosphorus-containing groups, protein serine/threonine kinase activity, ephrin receptor activity, protein tyrosine kinase activity
groupII	10753951	10810150	Ca	-	ENSGACG00000015779	ZFHX3	regulation of transcription, DNA-dependent, metal ion transport, nucleus, intracellular, sequence-specific DNA binding transcription factor activity, nucleic acid binding, zinc ion binding, metal ion binding, sequence-specific DNA binding, protein binding
groupII	10895790	10971790	Ca	psL	-	-	-
groupII	11659826	11819378	Ca	n	ENSGACG00000015876	-	-
groupII	11659826	11819378	Ca	n	ENSGACG00000015877	GIN1	-
groupII	11659826	11819378	Ca	n	ENSGACG00000015878	GFOD2	oxidoreductase activity
groupII	11659826	11819378	Ca	n	ENSGACG00000015879	THAP11	oxidation-reduction process, nucleic acid binding, oxidoreductase activity, flavin adenine dinucleotide binding
groupII	11659826	11819378	Ca	n	ENSGACG00000015880	SLC38A8 (2 of 2)	-
groupII	11659826	11819378	Ca	n	ENSGACG00000015882	FTSJD1	methylation, nucleic acid binding, methyltransferase activity, methylation, nucleic acid binding, methyltransferase activity
groupII	11659826	11819378	Ca	n	ENSGACG00000015885	GOT2	biosynthetic process, catalytic activity, pyridoxal phosphate binding, transferase activity, transferring nitrogenous groups, biosynthetic process, catalytic activity, pyridoxal phosphate binding, transferase activity, transferring nitrogenous groups, biosynthetic process, catalytic activity, pyridoxal phosphate binding, transferase activity, transferring nitrogenous groups
groupII	11659826	11819378	Ca	n	ENSGACG00000015895	SLC38A7	-
groupII	11659826	11819378	Ca	n	ENSGACG00000015897	-	proteolysis, serine-type carboxypeptidase activity
groupII	11659826	11819378	Ca	n	ENSGACG00000015908	-	-
groupII	11659826	11819378	Ca	n	ENSGACG00000015883	CALB2 (2 of 2)	calcium ion binding
groupII	11659826	11819378	Ca	n	ENSGACG00000015906	GAS8 (1 of 3)	-
groupII	11659826	11819378	Ca	n	ENSGACG00000015909	STRC	-
groupII	11659826	11819378	Ca	n	ENSGACG00000015911	FURIN (1 of 2)	proteolysis, cartilage development, cartilage morphogenesis, fin morphogenesis, serine-type endopeptidase activity

groupII	11827734	11877735	Ca	n	ENSGACG00000022262	-	-
groupII	11827734	11877735	Ca	n	ENSGACG00000015919	FES	protein phosphorylation, ATP binding, protein kinase activity, protein binding, transferase activity, transferring phosphorus-containing groups, fatty-acyl-CoA binding, protein serine/threonine kinase activity, protein tyrosine kinase activity
groupII	11827734	11877735	Ca	n	ENSGACG00000015923	RHCG (2 of 2)	-
groupII	11827734	11877735	Ca	n	ENSGACG00000015929	KIF7	microtubule-based movement, tubulin complex assembly, negative regulation of smoothened signaling pathway, determination of left/right symmetry, microtubule, Hedgehog signaling complex, ATP binding, unfolded protein binding, microtubule motor activity, protein binding
groupII	12060739	12118432	Ca	n	ENSGACG00000015958	IRF8	regulation of transcription, DNA-dependent, sequence-specific DNA binding transcription factor activity, protein binding
groupII	12060739	12118432	Ca	n	ENSGACG00000015963	COX4I1	response to hypoxia, cytochrome-c oxidase activity
groupII	12060739	12118432	Ca	n	ENSGACG00000015965	EMC8	-
groupII	12060739	12118432	Ca	n	ENSGACG00000015967	GINS2	-
groupII	12060739	12118432	Ca	n	ENSGACG00000015961	-	protein dephosphorylation, dephosphorylation, protein tyrosine phosphatase activity, protein tyrosine/serine/threonine phosphatase activity, phosphatase activity
groupII	12708573	12785554	Ca	n	ENSGACG00000016001	DEPDC7 (1 of 2)	intracellular signal transduction, phosphopantetheine binding
groupII	12708573	12785554	Ca	n	ENSGACG00000016002	QSER1	-
groupII	12708573	12785554	Ca	n	ENSGACG00000015995	HIPK3 (1 of 2)	protein phosphorylation, ATP binding, protein kinase activity, transferase activity, transferring phosphorus-containing groups, protein serine/threonine kinase activity
groupII	12708573	12785554	Ca	n	ENSGACG00000015997	CSTF3	RNA processing, mRNA processing, nucleus, intracellular
groupII	13981683	14010379	Ca	psR	ENSGACG00000016243	-	-
groupII	13981683	14010379	Ca	psR	ENSGACG00000016246	-	-
groupII	13981683	14010379	Ca	psR	ENSGACG00000016245	AHCTF1	nuclear pore complex assembly, digestive tract development
groupII	13981683	14010379	Ca	psR	ENSGACG00000016247	-	microtubule-based movement, ATP binding, protein binding, microtubule motor activity
groupII	14160454	14193070	Ca	n	ENSGACG00000016262	USH2A	cell adhesion, eye photoreceptor cell development, protein binding, structural molecule activity
groupII	22412750	22442750	Ca	n	ENSGACG00000017513	ZDHHC1	-
groupII	22412750	22442750	Ca	n	ENSGACG00000017514	HSD11B2	metabolic process, oxidoreductase activity, metabolic process, oxidoreductase activity
groupIII	239710	321207	Ca	n	ENSGACG00000012674	OXSR1 (1 of 2)	protein phosphorylation, ATP binding, protein kinase activity, transferase activity, transferring phosphorus-containing groups, protein serine/threonine kinase activity
groupIII	239710	321207	Ca	n	ENSGACG00000021273	-	-
groupIII	239710	321207	Ca	n	ENSGACG00000012795	HHATL (1 of 2)	-
groupIII	239710	321207	Ca	n	ENSGACG00000012831	KBTBD5 (2 of 2)	DNA repair, protein binding, methylated-DNA-[protein]-cysteine S-methyltransferase activity
groupIII	239710	321207	Ca	n	ENSGACG00000012842	CASP8	proteolysis, regulation of apoptotic process, apoptotic process, protein binding, cysteine-type endopeptidase activity, cysteine-type peptidase activity
groupIII	239710	321207	Ca	n	ENSGACG00000012847	C2orf62	signal transduction, cAMP-dependent protein kinase regulator activity
groupIII	239710	321207	Ca	n	ENSGACG00000012849	-	proteolysis, regulation of apoptotic process, apoptotic process, intracellular, protein binding, cysteine-type endopeptidase activity, cysteine-type peptidase activity
groupIII	239710	321207	Ca	n	ENSGACG00000012859	-	protein binding
groupIII	239710	321207	Ca	n	ENSGACG00000012863	CXorf38	-
groupIII	239710	321207	Ca	n	ENSGACG00000012708	PLCD1 (2 of 2)	signal transduction, intracellular signal transduction, lipid metabolic process, calcium ion binding, protein binding, phospholipase C activity, phosphatidylinositol phospholipase C activity, phosphoric diester hydrolase activity
groupIII	239710	321207	Ca	n	ENSGACG00000012836	VIPR1 (2 of 2)	G-protein coupled receptor signaling pathway, cell surface receptor signaling pathway, membrane, integral to membrane, G-protein coupled receptor activity, transmembrane signaling receptor activity
groupIII	239710	321207	Ca	n	ENSGACG00000012774	CTDSPL (2 of 2)	phosphatase activity, protein binding
groupIII	3044389	3469677	Ca	n	ENSGACG00000014270	NOP9	RNA binding, binding
groupIII	3044389	3469677	Ca	n	ENSGACG00000014287	-	transposition, DNA-mediated, DNA binding, transposase activity
groupIII	3044389	3469677	Ca	n	ENSGACG00000014245	CDH24	cell adhesion, homophilic cell adhesion, membrane, plasma membrane, calcium ion binding
groupIII	3044389	3469677	Ca	n	ENSGACG00000014253	LRRC16B (2 of 2)	protein binding
groupIII	3044389	3469677	Ca	n	ENSGACG00000014262	-	-

groupIII	3044389	3469677	Ca	n	ENSGACG00000014265	REM2 (2 of 2)	small GTPase mediated signal transduction, protein transport, dopaminergic neuron differentiation, intracellular, nucleotide binding, GTP binding, nucleoside-triphosphatase activity
groupIII	3044389	3469677	Ca	n	ENSGACG00000014290	FAM5B	-
groupIII	3509912	3547026	Ca	n	-	-	-
groupIII	3726483	3850207	Ca	n	ENSGACG00000014317	PAPPA2	cell differentiation, membrane
groupIII	3726483	3850207	Ca	n	ENSGACG00000014303	ASTN1	protein binding
groupIII	3901803	4171166	Ca	psL	ENSGACG00000014339	TOE1	regulation of transcription, DNA-dependent, nucleus, sequence-specific DNA binding transcription factor activity, nucleic acid binding, zinc ion binding
groupIII	3901803	4171166	Ca	psL	ENSGACG00000014352	-	integral to membrane
groupIII	3901803	4171166	Ca	psL	ENSGACG00000014354	FABP6	binding, bile acid binding
groupIII	3901803	4171166	Ca	psL	ENSGACG00000014377	UROD	porphyrin-containing compound biosynthetic process, porphyrin-containing compound metabolic process, uroporphyrinogen decarboxylase activity
groupIII	3901803	4171166	Ca	psL	ENSGACG00000014381	LYN	protein phosphorylation, ATP binding, protein kinase activity, protein binding, transferase activity, transferring phosphorus-containing groups, protein serine/threonine kinase activity, protein tyrosine kinase activity
groupIII	3901803	4171166	Ca	psL	ENSGACG00000014398	-	-
groupIII	3901803	4171166	Ca	psL	ENSGACG00000014328	XRN1	intracellular, nucleic acid binding, exonuclease activity
groupIII	3901803	4171166	Ca	psL	ENSGACG00000014348	-	selenium binding
groupIII	3901803	4171166	Ca	psL	ENSGACG00000014366	ZSWIM5	zinc ion binding
groupIII	3901803	4171166	Ca	psL	ENSGACG00000014401	CTNND2 (1 of 2)	binding, protein binding
groupIII	5561006	5633137	Ca	psL	ENSGACG00000014732	PENK	-
groupIII	5561006	5633137	Ca	psL	ENSGACG00000014733	SDR16C5	metabolic process, oxidoreductase activity
groupIII	5561006	5633137	Ca	psL	ENSGACG00000014747	SLC44A5 (2 of 2)	-
groupIII	7439985	7465237	Ca	n	ENSGACG00000015299	MEX3D	zinc ion binding, RNA binding, protein binding
groupIII	7439985	7465237	Ca	n	ENSGACG00000015282	TCF3 (2 of 2)	regulation of transcription, DNA-dependent, striated muscle cell development, protein binding
groupIII	7439985	7465237	Ca	n	ENSGACG00000015296	-	-
groupIII	8549327	8624152	Ca	psL	ENSGACG00000015582	-	-
groupIII	8549327	8624152	Ca	psL	ENSGACG00000015588	-	-
groupIII	8549327	8624152	Ca	psL	ENSGACG00000015590	ANO8 (2 of 2)	-
groupIII	8549327	8624152	Ca	psL	ENSGACG00000015576	KCNN1 (1 of 2)	potassium ion transport, integral to membrane, calcium-activated potassium channel activity, calmodulin binding
groupIII	8549327	8624152	Ca	psL	ENSGACG00000015580	-	metal ion binding
groupIII	8549327	8624152	Ca	psL	ENSGACG00000015583	NR2F6 (1 of 2)	regulation of transcription, DNA-dependent, steroid hormone mediated signaling pathway, nucleus, sequence-specific DNA binding transcription factor activity, steroid hormone receptor activity, zinc ion binding, sequence-specific DNA binding
groupIII	8549327	8624152	Ca	psL	ENSGACG00000015594	DDA1 (1 of 2)	-
groupIII	10256794	10270838	Ca	psL	-	-	-
groupIII	10600649	10625505	Ca	n	ENSGACG00000016469	MYLK4 (2 of 2)	protein phosphorylation, ATP binding, protein kinase activity, transferase activity, transferring phosphorus-containing groups, protein serine/threonine kinase activity
groupIII	10626712	10750873	Ca	n	ENSGACG00000016471	HECTD3	cell adhesion, cellular protein modification process, intracellular, acid-amino acid ligase activity
groupIII	10626712	10750873	Ca	n	ENSGACG00000016489	RPS8	translation, intracellular, ribosome, structural constituent of ribosome
groupIII	10626712	10750873	Ca	n	ENSGACG00000022724	-	-
groupIII	10626712	10750873	Ca	n	ENSGACG00000022386	-	-
groupIII	10626712	10750873	Ca	n	ENSGACG00000022870	-	-
groupIII	10626712	10750873	Ca	n	ENSGACG00000022882	-	-
groupIII	10626712	10750873	Ca	n	ENSGACG00000022325	-	-
groupIII	10626712	10750873	Ca	n	ENSGACG00000022380	-	-
groupIII	10626712	10750873	Ca	n	ENSGACG00000022438	-	-
groupIII	10626712	10750873	Ca	n	ENSGACG00000016497	ACADM	oxidation-reduction process, metabolic process, oxidoreductase activity, acting on the CH-CH group of donors, acyl-CoA dehydrogenase activity
groupIII	10626712	10750873	Ca	n	ENSGACG00000016522	RABGGTB	catalytic activity

groupIII	10626712	10750873	Ca	n	ENSGACG00000016529	ST6GALNAC3	protein glycosylation, integral to Golgi membrane, sialyltransferase activity
groupIII	10626712	10750873	Ca	n	ENSGACG00000016532	ST6GAL-C5-(1-of-2)	protein glycosylation, integral to Golgi membrane, sialyltransferase activity
groupIII	10805588	10830135	Ca	n	ENSGACG00000016538	AK5	signal transduction, nucleobase-containing compound metabolic process, ATP binding, nucleotide binding, nucleobase-containing compound kinase activity, nucleoside-triphosphatase activity, cAMP-dependent protein kinase regulator activity, nucleobase-containing compound metabolic process, ATP binding, nucleobase-containing compound kinase activity
groupIII	10849623	10934254	Ca	n	ENSGACG00000016560	FAM73A	-
groupIII	10849623	10934254	Ca	n	ENSGACG00000022857	-	-
groupIII	10849623	10934254	Ca	n	ENSGACG00000016567	SLC25A24 (3 of 3)	calcium ion binding
groupIII	10849623	10934254	Ca	n	ENSGACG00000016570	DNAJB4	protein folding, unfolded protein binding, heat shock protein binding
groupIII	10849623	10934254	Ca	n	ENSGACG00000016582	TXNDC12	cell redox homeostasis
groupIII	10849623	10934254	Ca	n	ENSGACG00000016586	BTF3L4 (2 of 2)	-
groupIII	10849623	10934254	Ca	n	ENSGACG00000016551	USP33	ubiquitin-dependent protein catabolic process, zinc ion binding, ubiquitin thiolesterase activity
groupIII	10849623	10934254	Ca	n	ENSGACG00000016576	GIPC2	protein binding
groupIII	10849623	10934254	Ca	n	ENSGACG00000016595	ZFYVE9 (1 of 3)	methylation, nucleic acid binding, metal ion binding, methyltransferase activity
groupIII	10849623	10934254	Ca	n	ENSGACG00000016563	FUBP1	-
groupIII	11244935	11263935	Ca	psR	ENSGACG00000016672	-	-
groupIV	18541913	18598794	Ca	psR	ENSGACG00000018851	-	integral to membrane
groupIV	18541913	18598794	Ca	psR	ENSGACG00000018853	PUS7L	pseudouridine synthesis, RNA modification, RNA binding, pseudouridine synthase activity
groupIV	18541913	18598794	Ca	psR	ENSGACG00000018852	-	integral to membrane
groupIV	19106685	19147353	Ca	psR	-	-	-
groupIV	19368508	19464145	Ca	bg	ENSGACG00000018923	DOCK4	ATP binding, GTP binding, protein binding, guanyl-nucleotide exchange factor activity, GTPase binding
groupIV	19464803	19638023	Ca	n	ENSGACG00000018926	LRRN3	protein binding
groupIV	19464803	19638023	Ca	n	ENSGACG00000018927	-	-
groupIV	19464803	19638023	Ca	n	ENSGACG00000018929	CALD1 (1 of 2)	-
groupIV	19639233	19770357	Ca	n	ENSGACG00000018945	TMEM209	-
groupIV	19639233	19770357	Ca	n	ENSGACG00000018951	-	cell cycle arrest, nucleus, cyclin-dependent protein kinase inhibitor activity
groupIV	19639233	19770357	Ca	n	ENSGACG00000018953	SLC37A3	transmembrane transport, integral to membrane
groupIV	19639233	19770357	Ca	n	ENSGACG00000018929	CALD1 (1 of 2)	-
groupIV	19639233	19770357	Ca	n	ENSGACG00000018935	DHX57	nucleic acid binding, zinc ion binding, ATP binding, protein binding, helicase activity, ATP-dependent helicase activity
groupIV	19639233	19770357	Ca	n	ENSGACG00000018938	FAM40B	nucleobase-containing compound metabolic process, nucleic acid binding, nucleotide binding, DNA-directed DNA polymerase activity
groupIV	19639233	19770357	Ca	n	ENSGACG00000018942	AHCYL2 (1 of 2)	one-carbon metabolic process, adenosylhomocysteinase activity
groupIV	19639233	19770357	Ca	n	ENSGACG00000018950	PRDM4	-
groupIV	19639233	19770357	Ca	n	ENSGACG00000018930	DNM1L (1 of 2)	GTPase activity, GTP binding
groupIX	89398	103516	Ca	n	-	-	-
groupIX	16179366	16188484	Ca	n	ENSGACG00000019268	ACOX1	oxidation-reduction process, metabolic process, fatty acid beta-oxidation, peroxisome, oxidoreductase activity, acting on the CH-CH group of donors, acyl-CoA oxidase activity, acyl-CoA dehydrogenase activity
groupV	3705816	3719313	Ca	psR	ENSGACG00000003504	C10orf2	nucleotide binding, nucleoside-triphosphatase activity
groupV	3971488	4042295	Ca	psR	ENSGACG00000003707	TMEM106A	-
groupV	3971488	4042295	Ca	psR	ENSGACG00000003710	-	small GTPase mediated signal transduction, intracellular protein transport, protein transport, intracellular, GTP binding
groupV	3971488	4042295	Ca	psR	ENSGACG00000003702	NBR1 (2 of 2)	intracellular, zinc ion binding, protein binding
groupV	3971488	4042295	Ca	psR	ENSGACG00000003719	SOST	extracellular space
groupV	4946095	4988621	Ca	n	ENSGACG00000004243	MAPT (1 of 2)	negative regulation of microtubule depolymerization
groupV	4946095	4988621	Ca	n	ENSGACG00000004258	KANSL1	phosphoenolpyruvate-dependent sugar phosphotransferase system, sugar:hydrogen symporter activity
groupV	5681034	5709891	Ca	n	ENSGACG00000004597	-	-
groupV	5681034	5709891	Ca	n	ENSGACG00000004617	PDAP1	-
groupV	5681034	5709891	Ca	n	ENSGACG00000004626	CBY1	-
groupV	5681034	5709891	Ca	n	ENSGACG00000004637	PLBD1 (2 of 2)	-

groupV	5681034	5709891	Ca	n	ENSGACG00000004643	KCTD17	potassium ion transport, membrane, voltage-gated potassium channel complex, protein binding, voltage-gated potassium channel activity
groupV	5681034	5709891	Ca	n	ENSGACG00000004601	SH3BP1	signal transduction, intracellular, cytoplasm, protein binding
groupV	5791653	5814653	Ca	psR	ENSGACG00000004836	EP300 (2 of 2)	regulation of transcription, DNA-dependent, nucleus, transcription cofactor activity, zinc ion binding, protein binding, histone acetyltransferase activity, transcription coactivator activity
groupV	12059810	12077810	Ca	psL	ENSGACG00000010113	-	regulation of transcription, DNA-dependent, transcription initiation from RNA polymerase II promoter, DNA binding
groupV	12059810	12077810	Ca	psL	ENSGACG00000010117	-	-
groupV	12059810	12077810	Ca	psL	ENSGACG00000010120	ANKFN1	protein binding
groupVI	5852309	5884101	Ca	psR	ENSGACG00000004737	RRBP1	protein transport, integral to endoplasmic reticulum membrane
groupVI	5852309	5884101	Ca	psR	ENSGACG00000004750	-	intracellular, actin binding
groupVI	5852309	5884101	Ca	psR	ENSGACG00000004767	CAPN2 (2 of 3)	proteolysis, intracellular, calcium ion binding, cysteine-type endopeptidase activity, calcium-dependent cysteine-type endopeptidase activity
groupVI	6869880	6914880	Ca	n	ENSGACG00000005596	ERLEC1	-
groupVI	6869880	6914880	Ca	n	ENSGACG00000005682	GPR75	-
groupVI	6869880	6914880	Ca	n	ENSGACG00000005686	PSME4 (1 of 2)	binding, protein binding
groupVI	6869880	6914880	Ca	n	ENSGACG00000005759	WDR27	protein binding
groupVI	11126533	11168385	Ca	psL	ENSGACG00000009390	SORCS3 (2 of 2)	integral to membrane
groupVI	11126533	11168385	Ca	psL	ENSGACG00000009419	SORCS1 (2 of 2)	integral to membrane
groupVI	11656170	11717339	Ca	n	ENSGACG00000009677	METTL10	metabolic process, tRNA modification, methyltransferase activity, tRNA (guanine-N7-)-methyltransferase activity
groupVI	11656170	11717339	Ca	n	ENSGACG00000009665	FAM175B	-
groupVI	11656170	11717339	Ca	n	ENSGACG00000009695	FAM53B (1 of 2)	-
groupVI	11656170	11717339	Ca	n	ENSGACG00000009658	ZRANB1 (2 of 2)	intracellular, zinc ion binding
groupVII	1804939	1834167	Ca	psL	ENSGACG00000018948	-	binding, binding, binding
groupVII	1804939	1834167	Ca	psL	ENSGACG00000018949	-	transport, transmembrane transport, membrane
groupVII	1804939	1834167	Ca	psL	ENSGACG00000018954	-	G-protein coupled receptor signaling pathway, integral to membrane
groupVII	1804939	1834167	Ca	psL	ENSGACG00000018947	-	-
groupVII	1804939	1834167	Ca	psL	ENSGACG00000018955	-	transport, sodium ion transport, transmembrane transport, chloride transport, membrane, integral to membrane, cation:chloride symporter activity
groupVII	5838359	5931359	Ca	n	ENSGACG00000019528	PTPRD (2 of 2)	protein dephosphorylation, dephosphorylation, protein tyrosine phosphatase activity, phosphatase activity, protein binding, protein dephosphorylation, dephosphorylation, protein tyrosine phosphatase activity, phosphatase activity, protein binding
groupVII	12605026	12752524	Ca	psL	ENSGACG00000020164	IRF2BP1	-
groupVII	12605026	12752524	Ca	psL	ENSGACG00000020165	HNRNPL (2 of 2)	nucleic acid binding
groupVII	12605026	12752524	Ca	psL	ENSGACG00000020166	-	cell communication, calcium ion transport, transmembrane transport, membrane, integral to membrane, calcium:sodium antiporter activity, cell communication, calcium ion transport, transmembrane transport, membrane, integral to membrane, calcium:sodium antiporter activity
groupVII	12605026	12752524	Ca	psL	ENSGACG00000020168	CKM (1 of 2)	kinase activity, transferase activity, transferring phosphorus-containing groups
groupVII	12605026	12752524	Ca	psL	ENSGACG00000020171	-	regulation of phosphorylation, cytoplasm
groupVII	12605026	12752524	Ca	psL	ENSGACG00000020174	-	calcium ion binding
groupVII	12605026	12752524	Ca	psL	ENSGACG00000020167	NCCRP1	protein catabolic process
groupVII	12605026	12752524	Ca	psL	ENSGACG00000020169	MARK4 (2 of 2)	protein phosphorylation, ATP binding, protein kinase activity, protein binding, transferase activity, transferring phosphorus-containing groups, protein serine/threonine kinase activity
groupVII	12605026	12752524	Ca	psL	ENSGACG00000020172	-	multicellular organismal development, cell communication, Notch signaling pathway, membrane, integral to membrane, calcium ion binding, protein binding
groupVII	12605026	12752524	Ca	psL	ENSGACG00000020173	-IF1-(2-of-3)	protein binding
groupVII	12605026	12752524	Ca	psL	ENSGACG00000020170	-	-
groupVII	16988582	17047582	Ca	bg	ENSGACG00000020323	-	nucleic acid binding, metal ion binding, hydrolase activity
groupVII	16988582	17047582	Ca	bg	ENSGACG00000020324	-	-
groupVII	16988582	17047582	Ca	bg	ENSGACG00000020325	KDM6B (2 of 2)	fin regeneration, protein binding

groupVII	16988582	17047582	Ca	bg	ENSGACG00000020322	NLGN2 (2 of 2)	-
groupVII	16988582	17047582	Ca	bg	ENSGACG00000020327	AIFM1	oxidation-reduction process, oxidoreductase activity, flavin adenine dinucleotide binding, oxidation-reduction process, oxidoreductase activity, flavin adenine dinucleotide binding, oxidation-reduction process, oxidoreductase activity, flavin adenine dinucleotide binding, oxidation-reduction process, oxidoreductase activity, flavin adenine dinucleotide binding, oxidation-reduction process, oxidoreductase activity, flavin adenine dinucleotide binding
groupVII	16988582	17047582	Ca	bg	ENSGACG00000020326	GLRA4 (2 of 2)	transport, ion transport, membrane, integral to membrane, postsynaptic membrane, extracellular ligand-gated ion channel activity, transport, ion transport, membrane, integral to membrane, postsynaptic membrane, extracellular ligand-gated ion channel activity
groupVII	18658618	18672817	Ca	-	ENSGACG00000020445	SSSCA1	-
groupVII	18658618	18672817	Ca	-	ENSGACG00000020446	ARR3 (2 of 2)	signal transduction, visual perception, signal transduction
groupVII	18658618	18672817	Ca	-	ENSGACG00000020444	EHBP1L1 (2 of 2)	protein binding,
groupVII	19290469	19381959	Ca	psL	ENSGACG00000020500	-	membrane
groupVII	19290469	19381959	Ca	psL	ENSGACG00000020501	-	membrane, tight junction, structural molecule activity, membrane, tight junction, structural molecule activity
groupVII	19290469	19381959	Ca	psL	ENSGACG00000020502	-	membrane, tight junction, structural molecule activity
groupVII	19290469	19381959	Ca	psL	ENSGACG00000020503	-	membrane, integral to membrane, tight junction, structural molecule activity
groupVII	19290469	19381959	Ca	psL	ENSGACG00000020504	-	membrane, tight junction, structural molecule activity
groupVII	19290469	19381959	Ca	psL	ENSGACG00000020506	-	membrane
groupVII	19290469	19381959	Ca	psL	ENSGACG00000020507	-	cytoplasm, cytoskeleton, extrinsic to membrane, cytoskeletal protein binding
groupVII	19290469	19381959	Ca	psL	ENSGACG00000020508	WBSCR27 (1 of 2)	metabolic process, methyltransferase activity
groupVII	19290469	19381959	Ca	psL	ENSGACG00000020509	-	protein binding
groupVII	19290469	19381959	Ca	psL	ENSGACG00000020512	SDF2	membrane, membrane
groupVII	19290469	19381959	Ca	psL	ENSGACG00000020505	-	membrane, integral to membrane, tight junction, structural molecule activity
groupVII	19290469	19381959	Ca	psL	ENSGACG00000020510	-	protein binding
groupVII	19290469	19381959	Ca	psL	ENSGACG00000020511	-	calcium ion binding
groupVII	19290469	19381959	Ca	psL	ENSGACG00000020513	SUPT6H	embryonic heart tube development, otic vesicle development, heart development, somitogenesis, nucleobase-containing compound metabolic process, RNA binding, protein binding, hydrolase activity, acting on ester bonds
groupVII	21390879	21499482	Ca	psR	ENSGACG00000020612	-	transmembrane transport, drug transmembrane transport, membrane, drug transmembrane transporter activity, antiporter activity, transmembrane transport, drug transmembrane transport, membrane, drug transmembrane transporter activity, antiporter activity
groupVII	21390879	21499482	Ca	psR	ENSGACG00000020613	-	transmembrane transport, drug transmembrane transport, membrane, drug transmembrane transporter activity, antiporter activity, transmembrane transport, drug transmembrane transport, membrane, drug transmembrane transporter activity, antiporter activity
groupVII	21390879	21499482	Ca	psR	ENSGACG00000020614	-	transmembrane transport, drug transmembrane transport, membrane, drug transmembrane transporter activity, antiporter activity
groupVII	21390879	21499482	Ca	psR	ENSGACG00000020615	-	transmembrane transport, drug transmembrane transport, membrane, drug transmembrane transporter activity, antiporter activity
groupVII	21390879	21499482	Ca	psR	ENSGACG00000020616	TMIGD1	protein binding
groupVII	21925471	21967682	Ca	n	ENSGACG00000020655	PURA	double-stranded DNA binding
groupVII	21925471	21967682	Ca	n	ENSGACG00000020656	SH2D4A (2 of 2)	-
groupVII	21925471	21967682	Ca	n	ENSGACG00000020654	NRG2 (1 of 2)	embryo development, receptor binding, protein binding
groupVII	22530582	22575899	Ca	n	ENSGACG00000020696	KC-B3-(1-of-2)	potassium ion transport, cytoplasm, voltage-gated potassium channel activity, potassium ion transport, cytoplasm, voltage-gated potassium channel activity
groupVII	22530582	22575899	Ca	n	ENSGACG00000020698	CHRDL2	dorsal/ventral pattern formation, protein binding, peptidase inhibitor activity, protein binding, peptidase inhibitor activity
groupVII	22530582	22575899	Ca	n	ENSGACG00000020697	NEU3	-
groupVII	26935378	26964299	Ca	n	ENSGACG00000020855	RABEP1	vesicle-mediated transport, cilium assembly, membrane, growth factor activity, GTPase activator activity, growth factor activity, GTPase activator activity
groupVII	26935378	26964299	Ca	n	ENSGACG00000020856	VPS37D	-
groupVIII	1170952	1204952	Ca	n	ENSGACG00000003176	-	protein binding
groupVIII	3398247	3435996	Ca	-	ENSGACG00000004896	TBC1D7	regulation of Rab GTPase activity, intracellular, Rab GTPase activator activity
groupVIII	3398247	3435996	Ca	-	ENSGACG00000004923	DSP (2 of 2)	cytoskeleton, nucleotide binding
groupVIII	3398247	3435996	Ca	-	ENSGACG00000004884	PHACTR1 (2 of 2)	regulation of transcription, DNA-dependent, transcription initiation, DNA-dependent, sequence-specific DNA binding transcription factor activity, DNA binding, sigma factor activity

groupVIII	3711279	3744471	Ca	n	ENSGACG00000005020	GMDS	cellular metabolic process, GDP-mannose metabolic process, retina layer formation, dorsal motor nucleus of vagus nerve development, vagus nerve development, Notch signaling pathway, intracellular, oxidoreductase activity, catalytic activity, coenzyme binding, GDP-mannose 4, 6-dehydratase activity, fucosyltransferase activity
groupVIII	6746676	6792643	Ca	psL	ENSGACG00000006461	STXBP3	vesicle-mediated transport, vesicle docking involved in exocytosis
groupVIII	6746676	6792643	Ca	psL	ENSGACG00000006499	-SP-(2-of-2)	protein binding
groupVIII	6746676	6792643	Ca	psL	ENSGACG00000006451	FNDC7 (1 of 3)	protein binding
groupVIII	6746676	6792643	Ca	psL	ENSGACG00000006490	ANKMY1	zinc ion binding, protein binding
groupVIII	6746676	6792643	Ca	psL	ENSGACG00000006515	-	membrane, proteinaceous extracellular matrix, heparan sulfate proteoglycan binding
groupVIII	7018909	7121002	Ca	bg	ENSGACG00000006617	C1orf52	-
groupVIII	7018909	7121002	Ca	bg	ENSGACG00000006695	ZNHIT6	-
groupVIII	7018909	7121002	Ca	bg	ENSGACG00000006637	COL24A1	cell adhesion, collagen, extracellular matrix structural constituent, structural molecule activity
groupVIII	18766800	18790513	Ca	n	ENSGACG00000014307	EXOC1	-
groupVIII	18766800	18790513	Ca	n	ENSGACG00000014325	CEP135	-
groupX	15139527	15189527	Ca	bg	ENSGACG00000009869	-	-
groupX	15139527	15189527	Ca	bg	ENSGACG00000009862	GRIK5 (1 of 2)	membrane, ionotropic glutamate receptor activity, extracellular-glutamate-gated ion channel activity
groupX	15363050	15657440	Ca	bg	ENSGACG00000010048	-	-
groupX	15363050	15657440	Ca	bg	ENSGACG00000010061	-	-
groupX	15363050	15657440	Ca	bg	ENSGACG00000010063	JTB	integral to membrane, integral to membrane
groupX	15363050	15657440	Ca	bg	ENSGACG00000010080	CCT3	protein folding, cellular protein metabolic process, embryonic eye morphogenesis, negative regulation of retinal cell programmed cell death, ATP binding, unfolded protein binding
groupX	15363050	15657440	Ca	bg	ENSGACG00000010328	-	DNA binding, protein binding
groupX	15363050	15657440	Ca	bg	ENSGACG00000010333	-	-
groupX	15363050	15657440	Ca	bg	ENSGACG00000010334	-	-
groupX	15363050	15657440	Ca	bg	ENSGACG00000010341	INTS3	-
groupX	15363050	15657440	Ca	bg	ENSGACG00000010358	-	intracellular signal transduction, cyclic nucleotide biosynthetic process, phosphorus-oxygen lyase activity
groupX	15363050	15657440	Ca	bg	ENSGACG00000010381	RTL1	-
groupX	15363050	15657440	Ca	bg	ENSGACG00000010409	LRP12	protein binding
groupX	15363050	15657440	Ca	bg	ENSGACG00000010420	BOP1	rRNA processing, nucleus, protein binding
groupX	15363050	15657440	Ca	bg	ENSGACG00000010448	-	-
groupX	15363050	15657440	Ca	bg	ENSGACG00000010456	-	-
groupX	15363050	15657440	Ca	bg	ENSGACG00000010045	-	-
groupX	15363050	15657440	Ca	bg	ENSGACG00000010051	-	intracellular, nucleic acid binding, DNA binding, zinc ion binding
groupX	15363050	15657440	Ca	bg	ENSGACG00000010069	C1orf85	-
groupX	15363050	15657440	Ca	bg	ENSGACG00000010314	-	cell redox homeostasis, endoplasmic reticulum, isomerase activity
groupX	15363050	15657440	Ca	bg	ENSGACG00000010362	-	protein phosphorylation, ATP binding, protein kinase activity, transferase activity, transferring phosphorus-containing groups, protein serine/threonine kinase activity
groupX	15363050	15657440	Ca	bg	ENSGACG00000010376	-	protein binding
groupX	15363050	15657440	Ca	bg	ENSGACG00000010384	-	DNA repair, intracellular, DNA binding, zinc ion binding
groupX	15363050	15657440	Ca	bg	ENSGACG00000010388	DNAI2	protein binding, protein binding
groupX	15363050	15657440	Ca	bg	ENSGACG00000010415	WDYHV1	-
groupX	15363050	15657440	Ca	bg	ENSGACG00000010434	HSF1	regulation of transcription, DNA-dependent, nucleus, sequence-specific DNA binding transcription factor activity, DNA binding, sequence-specific DNA binding
groupXI	0	26231	Ca	psL	ENSGACG00000004178	ALDOA (1 of 2)	glycolysis, fructose-bisphosphate aldolase activity
groupXI	0	26231	Ca	psL	ENSGACG00000004234	GPS1	protein binding
groupXI	682193	749234	Ca	bg	ENSGACG00000005008	-	protein binding
groupXI	682193	749234	Ca	bg	ENSGACG00000005084	PSMD11 (1 of 2)	protein binding
groupXI	682193	749234	Ca	bg	ENSGACG00000005117	DLX3 (2 of 2)	regulation of transcription, DNA-dependent, nucleus, sequence-specific DNA binding transcription factor activity, sequence-specific DNA binding, protein binding

groupXI	682193	749234	Ca	bg	ENSGACG00000005125	-	-
groupXI	682193	749234	Ca	bg	ENSGACG00000005060	RPAIN	-
groupXI	682193	749234	Ca	bg	ENSGACG00000005120	DLX4 (2 of 2)	regulation of transcription, DNA-dependent, nucleus, sequence-specific DNA binding transcription factor activity, sequence-specific DNA binding, protein binding
groupXI	750788	911811	Ca	bg	ENSGACG00000005126	-GS-(2-of-2)	cellular amino acid biosynthetic process
groupXI	750788	911811	Ca	bg	ENSGACG00000005130	-GS-(1-of-2)	arginine biosynthetic process
groupXI	750788	911811	Ca	bg	ENSGACG00000005131	B4GALNT2	-
groupXI	750788	911811	Ca	bg	ENSGACG00000005138	-	-
groupXI	750788	911811	Ca	bg	ENSGACG00000005139	-	intracellular, DNA binding, zinc ion binding, binding
groupXI	750788	911811	Ca	bg	ENSGACG00000005143	COL1A1 (2 of 2)	signal transduction, regulation of ossification, collagen, cytoplasm, extracellular matrix structural constituent, receptor binding, protein binding, peptidase inhibitor activity
groupXI	1809317	1845492	Ca	n	-	-	-
groupXI	1876491	2024038	Ca	n	ENSGACG00000005654	PRAF2	-
groupXI	1876491	2024038	Ca	n	ENSGACG00000022243	-	-
groupXI	1876491	2024038	Ca	n	ENSGACG00000005722	PDK2 (1 of 2)	signal transduction, peptidyl-histidine phosphorylation, ATP binding, two-component sensor activity, protein histidine kinase activity
groupXI	1876491	2024038	Ca	n	ENSGACG00000005637	CBX1 (2 of 2)	-
groupXI	1876491	2024038	Ca	n	ENSGACG00000005644	NFE2L1 (2 of 2)	regulation of transcription, DNA-dependent, sequence-specific DNA binding transcription factor activity, DNA binding, sequence-specific DNA binding, protein dimerization activity
groupXI	1876491	2024038	Ca	n	ENSGACG00000005661	COPZ2	transport
groupXI	1876491	2024038	Ca	n	ENSGACG00000005720	PRR15L (2 of 2)	-
groupXI	2024649	2171900	Ca	n	ENSGACG00000005722	PDK2 (1 of 2)	signal transduction, peptidyl-histidine phosphorylation, ATP binding, two-component sensor activity, protein histidine kinase activity
groupXI	2024649	2171900	Ca	n	ENSGACG00000005761	-	proteasome activator complex
groupXI	2024649	2171900	Ca	n	ENSGACG00000005755	KCNC3 (2 of 2)	potassium ion transport, ion transport, transmembrane transport, membrane, voltage-gated potassium channel complex, protein binding, ion channel activity, voltage-gated potassium channel activity
groupXI	3068370	3116429	Ca	psL	ENSGACG00000006540	THRA (2 of 2)	regulation of transcription, DNA-dependent, steroid hormone mediated signaling pathway, nucleus, sequence-specific DNA binding transcription factor activity, steroid hormone receptor activity, zinc ion binding, sequence-specific DNA binding
groupXI	7313397	7391397	Ca	n	ENSGACG00000009661	NDUFA4 (1 of 2)	-
groupXI	7313397	7391397	Ca	n	ENSGACG00000009684	SLC35B1	transmembrane transport
groupXI	7313397	7391397	Ca	n	ENSGACG00000009718	FAM117A (1 of 2)	-
groupXI	7313397	7391397	Ca	n	ENSGACG00000009738	RNF113B	nucleic acid binding, zinc ion binding, protein binding
groupXI	7313397	7391397	Ca	n	ENSGACG00000009767	STRADA	protein phosphorylation, ATP binding, protein kinase activity, transferase activity, transferring phosphorus-containing groups, protein serine/threonine kinase activity
groupXI	7313397	7391397	Ca	n	ENSGACG00000009722	RUNDC3A (2 of 2)	-
groupXI	7313397	7391397	Ca	n	ENSGACG00000009736	-	binding
groupXI	7313397	7391397	Ca	n	ENSGACG00000009782	DDX42	nucleic acid binding, ATP binding, helicase activity, ATP-dependent helicase activity
groupXI	13652918	13675918	Ca	n	ENSGACG00000013889	-	iron ion binding, heme binding
groupXI	13652918	13675918	Ca	n	ENSGACG00000013891	-	iron ion binding, heme binding
groupXI	13652918	13675918	Ca	n	ENSGACG00000013895	-	iron ion binding, heme binding
groupXI	13652918	13675918	Ca	n	ENSGACG00000013902	-	iron ion binding, heme binding
groupXI	13652918	13675918	Ca	n	ENSGACG00000013918	-	iron ion binding, heme binding
groupXI	13652918	13675918	Ca	n	ENSGACG00000013930	NPRL3	-
groupXI	13652918	13675918	Ca	n	ENSGACG00000013945	MPG	base-excision repair, DNA binding, catalytic activity, alkylbase DNA N-glycosylase activity
groupXII	815557	832573	Ca	n	-	-	-
groupXII	9989286	10046399	Ca	n	ENSGACG00000007655	-	protein binding
groupXII	9989286	10046399	Ca	n	ENSGACG00000007674	-	protein binding
groupXII	9989286	10046399	Ca	n	ENSGACG00000007705	GNAT3	signal transduction, G-protein coupled receptor signaling pathway, detection of light stimulus involved in visual perception, signal transducer activity, guanyl nucleotide binding
groupXII	9989286	10046399	Ca	n	ENSGACG00000007643	EPS8L3 (1 of 2)	protein binding

groupXII	9989286	10046399	Ca	n	ENSGACG00000007691	AMPD2 (1 of 2)	purine ribonucleoside monophosphate biosynthetic process, deaminase activity, AMP deaminase activity
groupXII	9989286	10046399	Ca	n	ENSGACG00000007741	-	signal transduction, G-protein coupled receptor signaling pathway, small GTPase mediated signal transduction, intracellular, signal transducer activity, GTP binding, guanyl nucleotide binding
groupXII	10115006	10166512	Ca	n	ENSGACG00000007903	-	-
groupXII	10115006	10166512	Ca	n	ENSGACG00000007907	KCNC4	potassium ion transport, ion transport, transmembrane transport, membrane, voltage-gated potassium channel complex, protein binding, ion channel activity, voltage-gated potassium channel activity
groupXII	10115006	10166512	Ca	n	ENSGACG00000007894	AHCYL1 (1 of 2)	one-carbon metabolic process, adenosylhomocysteinase activity
groupXII	10115006	10166512	Ca	n	ENSGACG00000007913	SLC6A17 (2 of 2)	neurotransmitter transport, integral to membrane, neurotransmitter:sodium symporter activity
groupXII	11072495	11110495	Ca	n	ENSGACG00000008964	ZNF385A (1 of 2)	intracellular, nucleic acid binding, zinc ion binding
groupXII	11072495	11110495	Ca	n	ENSGACG00000008969	-	-
groupXII	12132540	12197373	Ca	n	ENSGACG00000009752	PKP1 (1 of 2)	binding, protein binding
groupXII	12132540	12197373	Ca	n	ENSGACG00000009758	TEAD3 (2 of 2)	regulation of transcription, DNA-dependent, nucleus, sequence-specific DNA binding transcription factor activity, protein binding
groupXII	12132540	12197373	Ca	n	ENSGACG00000022180	-	-
groupXII	12132540	12197373	Ca	n	ENSGACG00000022196	-	-
groupXII	12132540	12197373	Ca	n	ENSGACG00000009805	-	regulation of transcription, DNA-dependent, sequence-specific DNA binding transcription factor activity, sequence-specific DNA binding
groupXII	12132540	12197373	Ca	n	ENSGACG00000009806	LGR6	G-protein coupled receptor signaling pathway, integral to membrane, protein binding
groupXII	14768943	14848436	Ca	n	ENSGACG00000011155	TNFRSF11A	external side of plasma membrane, binding, CD154 receptor binding
groupXII	14768943	14848436	Ca	n	ENSGACG00000011160	-	regulation of transcription, DNA-dependent, embryonic retina morphogenesis in camera-type eye, hindbrain morphogenesis, retina layer formation, hindbrain development, neuron differentiation, nucleus, sequence-specific DNA binding transcription factor activity, DNA binding, sequence-specific DNA binding, protein binding
groupXII	14768943	14848436	Ca	n	ENSGACG00000011163	-	protein binding
groupXII	14768943	14848436	Ca	n	ENSGACG00000011168	PLEKHG5	regulation of Rho protein signal transduction, intracellular, protein binding, Rho guanyl-nucleotide exchange factor activity
groupXIII	2114566	2169566	Ca	n	ENSGACG00000004641	LIPG	protein binding
groupXIII	2114566	2169566	Ca	n	ENSGACG00000004685	SLC20A2	phosphate ion transport, membrane, inorganic phosphate transmembrane transporter activity
groupXIII	2114566	2169566	Ca	n	ENSGACG00000004678	KCMF1 (2 of 2)	intracellular, zinc ion binding
groupXIII	8277165	8296165	Ca	n	ENSGACG00000008235	-	-
groupXIII	8277165	8296165	Ca	n	ENSGACG00000008222	HIP1R	actin binding, phospholipid binding
groupXIII	10629917	10684029	Ca	psL	ENSGACG00000010293	WDR54	protein binding
groupXIII	10629917	10684029	Ca	psL	ENSGACG00000010296	NOL6	pathogenesis, RNA binding, hydrolase activity, acting on ester bonds, ribosome binding
groupXIII	10629917	10684029	Ca	psL	ENSGACG00000010311	-	DNA binding, protein dimerization activity
groupXIII	11040110	11131282	Ca	n	ENSGACG00000010397	FAM172A	protein folding, endoplasmic reticulum, calcium ion binding, unfolded protein binding
groupXIV	6240210	6284277	Ca	n	ENSGACG00000016869	BMPR1B (2 of 2)	protein phosphorylation, membrane, ATP binding, protein kinase activity, transferase activity, transferring phosphorus-containing groups, transmembrane receptor protein serine/threonine kinase activity, transforming growth factor beta-activated receptor activity, protein serine/threonine kinase activity
groupXIV	6240210	6284277	Ca	n	ENSGACG00000016879	PDLIM5 (1 of 2)	zinc ion binding, protein binding
groupXIV	11324616	11342791	Ca	bg	ENSGACG00000018121	GRIN1	transport, membrane, outer membrane-bounded periplasmic space, transporter activity, ionotropic glutamate receptor activity, extracellular-glutamate-gated ion channel activity, transport, membrane, outer membrane-bounded periplasmic space, transporter activity, ionotropic glutamate receptor activity, extracellular-glutamate-gated ion channel activity
groupXIV	11412157	11469157	Ca	n	ENSGACG00000018142	GTF3C4	-
groupXIV	14722480	14891949	Ca	n	ENSGACG00000018624	SLC38A9	-
groupXIV	14722480	14891949	Ca	n	ENSGACG00000018575	HMGCS1	metabolic process, isoprenoid biosynthetic process, catalytic activity, hydroxymethylglutaryl-CoA synthase activity
groupXIV	14722480	14891949	Ca	n	ENSGACG00000018584	TLN1	cell adhesion, cytoskeleton, actin cytoskeleton, actin binding, structural molecule activity
groupXIV	14722480	14891949	Ca	n	ENSGACG00000018619	IL6ST	protein binding
groupXIV	14722480	14891949	Ca	n	ENSGACG00000018632	NRG1	embryo development, receptor binding, protein binding
groupXIV	14722480	14891949	Ca	n	ENSGACG00000018599	TPM2	signal transduction, intracellular
groupXV	4900646	4979783	Ca	n	ENSGACG00000007827	NGB	response to hypoxia, iron ion binding, heme binding
groupXV	4900646	4979783	Ca	n	ENSGACG00000007850	ZDHHC22	regulation of transcription, DNA-dependent, nucleus, sequence-specific DNA binding transcription factor activity, zinc ion binding

groupXV	4900646	4979783	Ca	n	ENSGACG00000007852	PGF	membrane, growth factor activity
groupXV	4900646	4979783	Ca	n	ENSGACG00000007781	COQ6	oxidation-reduction process, ubiquinone biosynthetic process, monooxygenase activity, flavin adenine dinucleotide binding, oxidoreductase activity, acting on paired donors, with incorporation or reduction of molecular oxygen, NADH or NADPH as one donor, and incorporation of one atom of oxygen
groupXV	4900646	4979783	Ca	n	ENSGACG00000007840	TMEM63C	membrane
groupXV	4900646	4979783	Ca	n	ENSGACG00000007855	SLC35F4	membrane, binding
groupXV	4982178	5046634	Ca	n	ENSGACG00000007869	AP5M1	intracellular protein transport, vesicle-mediated transport, clathrin adaptor complex, protein binding
groupXV	4982178	5046634	Ca	n	ENSGACG00000007896	EXOC5	pronephros development, determination of left/right symmetry, exocytosis, vesicle docking, cytoplasm
groupXV	4982178	5046634	Ca	n	ENSGACG00000007855	SLC35F4	membrane, binding
groupXV	7356346	7404588	Ca	psR	ENSGACG00000009488	NIPAL3	-
groupXV	7356346	7404588	Ca	psR	ENSGACG00000009482	RCAN3	calcium-mediated signaling
groupXV	7356346	7404588	Ca	psR	ENSGACG00000009501	GRHL3	-
groupXV	13423851	13611254	Ca	psL	ENSGACG00000012839	-	protein phosphorylation, transmembrane receptor protein tyrosine kinase signaling pathway, membrane, ATP binding, protein kinase activity, transferase activity, transferring phosphorus-containing groups, protein serine/threonine kinase activity, transmembrane receptor protein tyrosine kinase activity, protein tyrosine kinase activity
groupXVI	5326551	5394349	Ca	psL	-	-	-
groupXVI	12042939	12117939	Ca	bg	ENSGACG00000006314	-	-
groupXVI	12042939	12117939	Ca	bg	ENSGACG00000006317	MXRA5 (2 of 2)	protein binding
groupXVI	12042939	12117939	Ca	bg	ENSGACG00000006325	ARSE	metabolic process, catalytic activity, sulfuric ester hydrolase activity
groupXVII	3084684	3257913	Ca	n	ENSGACG00000005003	IGFN1 (3 of 5)	protein binding
groupXVII	3084684	3257913	Ca	n	ENSGACG00000005005	IGFN1 (5 of 5)	protein binding
groupXVII	3084684	3257913	Ca	n	ENSGACG00000005015	SYT2 (1 of 2)	protein binding
groupXVII	3084684	3257913	Ca	n	ENSGACG00000005023	-	carbohydrate metabolic process, protein binding, phosphotransferase activity, alcohol group as acceptor
groupXVII	3084684	3257913	Ca	n	ENSGACG00000004954	GRM4 (2 of 2)	regulation of transcription, DNA-dependent, G-protein coupled receptor signaling pathway, integral to membrane, intracellular, sequence-specific DNA binding transcription factor activity, G-protein coupled receptor activity, sequence-specific DNA binding
groupXVII	3084684	3257913	Ca	n	ENSGACG00000004985	IGFN1 (4 of 5)	extracellular region, hormone activity, protein binding
groupXVII	3084684	3257913	Ca	n	ENSGACG00000004994	IGFN1 (2 of 5)	carbohydrate metabolic process, protein binding, phosphotransferase activity, alcohol group as acceptor
groupXVII	5569784	5608831	Ca	n	ENSGACG00000006741	C12orf10	-
groupXVII	5569784	5608831	Ca	n	ENSGACG00000006817	-	protein binding
groupXVII	5569784	5608831	Ca	n	ENSGACG00000006819	COL2A1 (1 of 2)	signal transduction, collagen, extracellular matrix structural constituent, receptor binding, protein binding, peptidase inhibitor activity
groupXVII	5706588	5748654	Ca	psL	ENSGACG00000006994	SHISA5	-
groupXVII	5706588	5748654	Ca	psL	ENSGACG00000006983	ITGA7	-
groupXVII	7810659	7838659	Ca	n	-	-	-
groupXVII	7998539	8027681	Ca	n	ENSGACG00000008650	CRYBB3 (2 of 2)	-
groupXVII	7998539	8027681	Ca	n	ENSGACG00000008653	-	-
groupXVII	7998539	8027681	Ca	n	ENSGACG00000008628	CIT	intracellular signal transduction, protein phosphorylation, metabolic process, ATP binding, protein kinase activity, catalytic activity, protein binding, transferase activity, transferring phosphorus-containing groups, protein serine/threonine kinase activity, small GTPase regulator activity
groupXVII	8493517	8644989	Ca	bg	ENSGACG00000008905	GLRX	cell redox homeostasis, electron carrier activity, protein disulfide oxidoreductase activity
groupXVII	8493517	8644989	Ca	bg	ENSGACG00000008945	-	protein binding
groupXVII	8493517	8644989	Ca	bg	ENSGACG00000008960	-	-
groupXVII	8493517	8644989	Ca	bg	ENSGACG00000009018	-	intracellular, nucleic acid binding, zinc ion binding
groupXVII	8493517	8644989	Ca	bg	ENSGACG00000009021	-	multicellular organismal development, camera-type eye development, brain development, extracellular region, hormone activity
groupXVII	8493517	8644989	Ca	bg	ENSGACG00000008899	RHOBTB3	protein binding
groupXVII	8493517	8644989	Ca	bg	ENSGACG00000008926	PCSK1	proteolysis, serine-type endopeptidase activity
groupXVII	8493517	8644989	Ca	bg	ENSGACG00000008930	CAST	-
groupXVII	8493517	8644989	Ca	bg	ENSGACG00000008967	DNAH8	proteolysis, microtubule-based movement, dynein complex, ATP binding, nucleotide binding, ATPase activity, cysteine-type endopeptidase activity, nucleoside-triphosphatase activity, microtubule motor activity

groupXVII	8493517	8644989	Ca	bg	ENSGACG00000008906	ELL2	nucleotide binding
groupXVII	8493517	8644989	Ca	bg	ENSGACG00000009034	PTPRA	protein dephosphorylation, dephosphorylation, regulation of protein kinase activity, convergent extension involved in gastrulation, retina development in camera-type eye, protein tyrosine phosphatase activity, phosphatase activity
groupXVII	8649227	8676321	Ca	psR	ENSGACG00000009049	IDH3B	oxidation-reduction process, tricarboxylic acid cycle, magnesium ion binding, NAD binding, oxidoreductase activity, acting on the CH-OH group of donors, NAD or NADP as acceptor, isocitrate dehydrogenase (NAD+) activity
groupXVII	8649227	8676321	Ca	psR	ENSGACG00000009111	WDR1	protein binding
groupXVII	8649227	8676321	Ca	psR	ENSGACG00000009129	SLC2A9	transport, transmembrane transport, membrane, integral to membrane, transporter activity, substrate-specific transmembrane transporter activity, transmembrane transporter activity, transport, transmembrane transport, membrane, integral to membrane, transporter activity, transmembrane transporter activity
groupXVII	8649227	8676321	Ca	psR	ENSGACG00000009034	PTPRA	protein dephosphorylation, dephosphorylation, regulation of protein kinase activity, convergent extension involved in gastrulation, retina development in camera-type eye, protein tyrosine phosphatase activity, phosphatase activity
groupXVII	8684309	8701309	Ca	psR	ENSGACG00000009129	SLC2A9	transport, transmembrane transport, membrane, integral to membrane, transporter activity, substrate-specific transmembrane transporter activity, transmembrane transporter activity, transport, transmembrane transport, membrane, integral to membrane, transporter activity, transmembrane transporter activity
groupXVII	9097537	9111537	Ca	n	ENSGACG00000009341	MAGI1 (1 of 2)	protein binding
groupXVII	9377111	9709696	Ca	psR	ENSGACG00000009434	TMF1	-
groupXVII	9377111	9709696	Ca	psR	ENSGACG00000009496	FGD5 (2 of 2)	regulation of Rho protein signal transduction, intracellular, metal ion binding, binding, protein binding, Rho guanyl-nucleotide exchange factor activity
groupXVII	9377111	9709696	Ca	psR	ENSGACG00000009440	UBA3 (1 of 2)	cellular protein modification process, ATP binding, catalytic activity, small protein activating enzyme activity
groupXVII	9377111	9709696	Ca	psR	ENSGACG00000009541	SFMBT1	regulation of transcription, DNA-dependent, nucleus, sequence-specific DNA binding, protein binding
groupXVII	9377111	9709696	Ca	psR	ENSGACG00000009400	EOGT	transferase activity, transferring glycosyl groups
groupXVII	9377111	9709696	Ca	psR	ENSGACG00000009511	PRKCD (2 of 2)	intracellular signal transduction, protein phosphorylation, ATP binding, protein kinase activity, protein binding, transferase activity, transferring phosphorus-containing groups, protein serine/threonine kinase activity
groupXVII	9377111	9709696	Ca	psR	ENSGACG00000009393	-	-
groupXVII	11389652	11476067	Ca	n	ENSGACG00000010974	-	-
groupXVII	11389652	11476067	Ca	n	ENSGACG00000010977	NUCKS1	-
groupXVII	11389652	11476067	Ca	n	ENSGACG00000010962	MFSD4 (1 of 2)	-
groupXVII	11389652	11476067	Ca	n	ENSGACG00000010970	ELK4	regulation of transcription, DNA-dependent, sequence-specific DNA binding transcription factor activity, sequence-specific DNA binding
groupXVII	11389652	11476067	Ca	n	ENSGACG00000010953	CDK18	-
groupXVIII	8455554	8484969	Ca	-	ENSGACG00000008882	CEP57L1	-
groupXVIII	8455554	8484969	Ca	-	ENSGACG00000008895	SESN1	cell cycle arrest, determination of left/right symmetry, nucleus
groupXVIII	8579077	8659466	Ca	n	ENSGACG00000008921	SNX3	cell communication, phosphatidylinositol binding, protein binding
groupXVIII	8579077	8659466	Ca	n	ENSGACG00000008950	OSTM1	-
groupXVIII	8579077	8659466	Ca	n	ENSGACG00000008916	LACE1 (2 of 2)	ATP binding, nucleotide binding, nucleoside-triphosphatase activity
groupXVIII	8579077	8659466	Ca	n	ENSGACG00000008934	NR2E1	regulation of transcription, DNA-dependent, steroid hormone mediated signaling pathway, nucleus, sequence-specific DNA binding transcription factor activity, steroid hormone receptor activity, zinc ion binding, sequence-specific DNA binding
groupXVIII	8579077	8659466	Ca	n	ENSGACG00000008956	OTOF (1 of 2)	integral to membrane, protein binding
groupXVIII	16233395	16266397	Ca	n	ENSGACG00000013807	CCR6 (1 of 2)	G-protein coupled receptor signaling pathway, integral to membrane
groupXVIII	16233395	16266397	Ca	n	ENSGACG00000013799	KIF26A (2 of 2)	microtubule-based movement, ATP binding, microtubule motor activity
groupXVIII	16233395	16266397	Ca	n	ENSGACG00000013808	FGFR1OP	protein binding
groupXX	1130999	1238921	Ca	n	ENSGACG00000003528	VPS13B (1 of 3)	protein binding
groupXX	1245211	1269211	Ca	psR	-	-	-
groupXX	1381478	1419534	Ca	n	ENSGACG00000003671	NCALD	calcium ion binding
groupXX	8888372	8911980	Ca	n	ENSGACG00000007563	-	regulation of Rab GTPase activity, intracellular, Rab GTPase activator activity
groupXX	8888372	8911980	Ca	n	ENSGACG00000007594	CTRL	proteolysis, catalytic activity, serine-type endopeptidase activity
groupXX	8888372	8911980	Ca	n	ENSGACG00000007597	-	G-protein coupled receptor signaling pathway, integral to membrane
groupXX	8888372	8911980	Ca	n	ENSGACG00000007600	LIN37	-

groupXX	8888372	8911980	Ca	n	ENSGACG00000007569	-	-
groupXX	13280249	13430768	Ca	psR	ENSGACG00000012131	TUFT1 (2 of 2)	-
groupXX	13280249	13430768	Ca	psR	ENSGACG00000012154	AQP10 (2 of 2)	transport, transmembrane transport, water transport, arsenite transport, glycerol transport, urea transmembrane transport, ion transmembrane transport, membrane, integral to membrane, transporter activity, water transmembrane transporter activity, glycerol transmembrane transporter activity, arsenite transmembrane transporter activity, urea transmembrane transporter activity
groupXX	13280249	13430768	Ca	psR	ENSGACG00000012156	ATP8B2	ATP biosynthetic process, phospholipid transport, membrane, integral to membrane, magnesium ion binding, ATP binding, ATPase activity, coupled to transmembrane movement of ions, phosphorylative mechanism, phospholipid-translocating ATPase activity
groupXX	13280249	13430768	Ca	psR	ENSGACG00000012171	-	-
groupXX	13280249	13430768	Ca	psR	ENSGACG00000012175	ZNF687 (2 of 2)	intracellular, zinc ion binding
groupXX	13280249	13430768	Ca	psR	ENSGACG00000012231	ECM1	extracellular region, extracellular space
groupXX	13280249	13430768	Ca	psR	ENSGACG00000012239	RORC	-
groupXX	13280249	13430768	Ca	psR	ENSGACG00000012243	THEM4	-
groupXX	13280249	13430768	Ca	psR	ENSGACG00000012247	-	-
groupXX	13280249	13430768	Ca	psR	ENSGACG00000012251	-	-
groupXX	13280249	13430768	Ca	psR	ENSGACG00000022251	-	-
groupXX	13280249	13430768	Ca	psR	ENSGACG00000012254	NBN	oxidation-reduction process, intracellular, oxidoreductase activity, protein binding
groupXX	13280249	13430768	Ca	psR	ENSGACG00000012271	OSGIN2	-
groupXX	13280249	13430768	Ca	psR	ENSGACG00000012277	GTPBP10	intracellular, GTP binding
groupXX	13280249	13430768	Ca	psR	ENSGACG00000012147	C1orf43 (2 of 2)	-
groupXX	13280249	13430768	Ca	psR	ENSGACG00000012182	PSMD4 (1 of 2)	phosphatidylinositol metabolic process, protein binding, phosphatidylinositol phosphate kinase activity
groupXX	13280249	13430768	Ca	psR	ENSGACG00000012292	-	proteolysis, zinc ion binding, metalloendopeptidase activity, protein binding
groupXX	13737344	13775527	Ca	psR	ENSGACG00000012464	-	-
groupXX	13737344	13775527	Ca	psR	ENSGACG00000012467	IGLON5	protein binding
groupXX	18375162	18395709	Ca	n	ENSGACG00000014132	GRIK2 (1 of 2)	membrane, ionotropic glutamate receptor activity, extracellular-glutamate-gated ion channel activity
groupXX	18396301	18427488	Ca	n	ENSGACG00000014132	GRIK2 (1 of 2)	membrane, ionotropic glutamate receptor activity, extracellular-glutamate-gated ion channel activity
groupXX	18431079	18498760	Ca	n	ENSGACG00000014132	GRIK2 (1 of 2)	membrane, ionotropic glutamate receptor activity, extracellular-glutamate-gated ion channel activity
groupI	3640578	3668766	No	psL	-	-	-
groupI	6669196	6684212	No	psR	ENSGACG00000008129	GRIA4 (2 of 2)	transport, membrane, outer membrane-bounded periplasmic space, transporter activity, ionotropic glutamate receptor activity, extracellular-glutamate-gated ion channel activity
groupI	7066648	7073648	No	n	ENSGACG00000008344	-	nucleic acid binding, zinc ion binding
groupI	10551080	10634322	No	n	ENSGACG00000010309	-	-
groupI	10551080	10634322	No	n	ENSGACG00000010316	-	G-protein coupled receptor signaling pathway, integral to membrane, G-protein coupled receptor activity
groupI	10551080	10634322	No	n	ENSGACG00000010322	-	G-protein coupled receptor signaling pathway, integral to membrane, G-protein coupled receptor activity
groupI	10551080	10634322	No	n	ENSGACG00000010302	GPR149	G-protein coupled receptor signaling pathway, integral to membrane
groupI	10551080	10634322	No	n	ENSGACG00000010305	MME	proteolysis, metalloendopeptidase activity, metallopeptidase activity
groupI	10551080	10634322	No	n	ENSGACG00000010323	-	G-protein coupled receptor signaling pathway, integral to membrane, G-protein coupled receptor activity
groupI	10551080	10634322	No	n	ENSGACG00000010313	-	G-protein coupled receptor signaling pathway, integral to membrane, G-protein coupled receptor activity
groupI	10635564	10686564	No	psR	ENSGACG00000010326	-	G-protein coupled receptor signaling pathway, integral to membrane, G-protein coupled receptor activity
groupI	10635564	10686564	No	psR	ENSGACG00000010329	-	G-protein coupled receptor signaling pathway, integral to membrane, G-protein coupled receptor activity
groupI	10635564	10686564	No	psR	ENSGACG00000010323	-	G-protein coupled receptor signaling pathway, integral to membrane, G-protein coupled receptor activity
groupI	10635564	10686564	No	psR	ENSGACG00000010337	-	G-protein coupled receptor signaling pathway, integral to membrane, G-protein coupled receptor activity
groupI	10810499	10833499	No	psL	ENSGACG00000010386	C3orf33	nucleic acid binding, hydrolase activity, acting on ester bonds
groupI	10810499	10833499	No	psL	ENSGACG00000010389	MED13 (2 of 2)	regulation of transcription from RNA polymerase II promoter, mediator complex
groupI	14084293	14127684	No	psR	ENSGACG00000011821	GBE1	carbohydrate metabolic process, catalytic activity, hydrolase activity, hydrolyzing O-glycosyl compounds, cation binding
groupI	14460461	14503461	No	n	ENSGACG00000011982	GUCA1C (2 of 2)	calcium ion binding
groupI	14460461	14503461	No	n	ENSGACG00000012003	THRSP	-
groupI	14460461	14503461	No	n	ENSGACG00000012006	RAB30	signal transduction, GTP catabolic process, small GTPase mediated signal transduction, intracellular protein transport, nucleocytoplasmic transport, protein transport, intracellular, GTPase activity, GTP binding

groupI	14460461	14503461	No	n	ENSGACG00000012026	PRCP	proteolysis, serine-type peptidase activity
groupI	14460461	14503461	No	n	ENSGACG00000012067	FAM181B	-
groupI	14867532	14986925	No	psR	ENSGACG00000012165	ARHGAP42 (1 of 2)	signal transduction, intracellular, protein binding
groupI	14867532	14986925	No	psR	ENSGACG00000012176	CNTN5	protein binding
groupI	14992970	15025971	No	bg	ENSGACG00000012227	FBXO40 (1 of 3)	zinc ion binding, protein binding
groupI	14992970	15025971	No	bg	ENSGACG00000012229	TTC36	protein binding
groupI	15040178	15140178	No	psR	ENSGACG00000012236	PVRL1 (1 of 2)	protein binding
groupI	15141068	15188452	No	psR	ENSGACG00000012245	-	-
groupI	15141068	15188452	No	psR	ENSGACG00000012236	PVRL1 (1 of 2)	protein binding
groupI	16149136	16166665	No	psR	-	-	-
groupI	16208580	16247580	No	psR	ENSGACG00000012627	-	-
groupI	16208580	16247580	No	psR	ENSGACG00000012628	-	RNA binding
groupI	16773172	16844435	No	n	ENSGACG00000013006	NAPA	intracellular protein transport
groupI	16773172	16844435	No	n	ENSGACG00000013015	GLTSCR1	-
groupI	16773172	16844435	No	n	ENSGACG00000013042	NUP88	-
groupI	16773172	16844435	No	n	ENSGACG00000013044	PSMD8	-
groupI	16773172	16844435	No	n	ENSGACG00000013085	DHX34	nuclear-transcribed mRNA catabolic process, nonsense-mediated decay, embryo development, nucleic acid binding, ATP binding, electron carrier activity, metal ion binding, nucleotide binding, helicase activity, nucleoside-triphosphatase activity
groupI	16773172	16844435	No	n	ENSGACG00000013023	EHD2 (1 of 2)	calcium ion binding, GTPase activity, GTP binding, protein binding
groupI	16773172	16844435	No	n	ENSGACG00000013083	SPRED3	-
groupI	16773172	16844435	No	n	ENSGACG00000013091	ST14 (1 of 3)	proteolysis, epidermis development, catalytic activity, protein binding, serine-type endopeptidase activity
groupI	16773172	16844435	No	n	ENSGACG00000012993	BCKDHA	metabolic process, oxidoreductase activity, acting on the aldehyde or oxo group of donors, disulfide as acceptor
groupI	17422835	17443056	No	psR	ENSGACG00000013367	INTS4	binding, protein binding
groupI	17422835	17443056	No	psR	ENSGACG00000013383	KCTD14	potassium ion transport, membrane, voltage-gated potassium channel complex, protein binding, voltage-gated potassium channel activity
groupI	17538140	17770140	No	n	ENSGACG00000013412	-	transposition, DNA-mediated, DNA binding, transposase activity
groupI	20065969	20119969	No	bg	ENSGACG00000013815	FAM117B	-
groupI	20065969	20119969	No	bg	ENSGACG00000013817	BMPR2 (1 of 2)	protein phosphorylation, membrane, ATP binding, protein kinase activity, transferase activity, transferring phosphorus-containing groups, transmembrane receptor protein serine/threonine kinase activity, transforming growth factor beta-activated receptor activity, protein serine/threonine kinase activity
groupI	20065969	20119969	No	bg	ENSGACG00000013821	DOCK9 (2 of 2)	GTP binding, protein binding, guanyl-nucleotide exchange factor activity, GTPase binding
groupI	24086139	24129140	No	n	-	-	-
groupI	26157890	26282104	No	psR	ENSGACG00000015207	NLGN4Y	-
groupI	27084804	27134192	No	psL	ENSGACG00000015339	DGKZ (2 of 2)	intracellular signal transduction, protein kinase C-activating G-protein coupled receptor signaling pathway, catalytic activity, protein binding, flavin adenine dinucleotide binding, diacylglycerol kinase activity
groupI	27084804	27134192	No	psL	ENSGACG00000015348	MDK	growth factor activity
groupI	27084804	27134192	No	psL	ENSGACG00000015349	CHRM4	G-protein coupled receptor signaling pathway, integral to membrane
groupI	27239156	27275751	No	n	ENSGACG00000015358	-	-
groupI	27239156	27275751	No	n	ENSGACG00000015361	-	G-protein coupled receptor signaling pathway, integral to membrane
groupII	4718324	4744324	No	psR	-	-	-
groupII	6023477	6086478	No	n	ENSGACG00000015100	ACE2	proteolysis, membrane, metallopeptidase activity, peptidyl-dipeptidase activity
groupII	6023477	6086478	No	n	ENSGACG00000015112	-	extracellular space
groupII	6023477	6086478	No	n	ENSGACG00000015118	PIR	-
groupII	6023477	6086478	No	n	ENSGACG00000015113	-	-
groupII	6023477	6086478	No	n	ENSGACG00000015098	NHS (2 of 2)	-
groupII	7849386	7923689	No	psR	ENSGACG00000015368	GPC5	membrane, proteinaceous extracellular matrix, protein binding, heparan sulfate proteoglycan binding
groupII	9299140	9314140	No	n	-	-	-
groupII	10573694	10618736	No	psR	ENSGACG00000015752	RPL13	translation, regulation of cell cycle, intracellular, ribosome, structural constituent of ribosome

groupII	10573694	10618736	No	psR	ENSGACG00000015754	CPNE7	protein binding, protein binding
groupII	10625190	10639190	No	bg	ENSGACG00000015773	-	G-protein coupled receptor signaling pathway, integral to membrane
groupII	10625190	10639190	No	bg	ENSGACG00000015774	CYB5B	heme binding, heme binding
groupII	10625190	10639190	No	bg	ENSGACG00000015776	DHX38	-
groupII	10709045	10807045	No	bg	ENSGACG00000015779	ZFHX3	regulation of transcription, DNA-dependent, metal ion transport, nucleus, intracellular, sequence-specific DNA binding transcription factor activity, nucleic acid binding, zinc ion binding, metal ion binding, sequence-specific DNA binding, protein binding
groupII	11181167	11200167	No	bg	ENSGACG00000015799	MTMR10	dephosphorylation, phosphatase activity
groupII	11181167	11200167	No	bg	ENSGACG00000015798	ZNF821	intracellular, DNA binding, zinc ion binding
groupII	11359686	11383686	No	psR	ENSGACG00000015829	GCOM1	-
groupII	11634168	11663168	No	n	ENSGACG00000015872	TRADD	signal transduction, positive regulation of I-kappaB kinase/NF-kappaB cascade, induction of apoptosis, cytoplasm, signal transducer activity, protein binding, signal transduction, positive regulation of I-kappaB kinase/NF-kappaB cascade, induction of apoptosis, positive regulation of apoptotic process, cytoplasm, signal transducer activity, protein binding
groupII	11634168	11663168	No	n	ENSGACG00000015873	G-O1-(2-of-2)	signal transduction, G-protein coupled receptor signaling pathway, small GTPase mediated signal transduction, intracellular, signal transducer activity, GTP binding, guanyl nucleotide binding
groupII	11634168	11663168	No	n	ENSGACG00000015876	-	-
groupII	11634168	11663168	No	n	ENSGACG00000015877	GIN1	-
groupII	11634168	11663168	No	n	ENSGACG00000015875	COG4	-
groupII	12186277	12212277	No	psL	-	-	-
groupII	12618205	12654205	No	psL	ENSGACG00000015990	-	-
groupIII	2643074	2666074	No	n	-	-	-
groupIII	7153675	7171062	No	psR	ENSGACG00000022187	-	-
groupIII	11312635	11328635	No	psL	ENSGACG00000016677	-	protein dephosphorylation, dephosphorylation, membrane, protein tyrosine phosphatase activity, phosphatase activity, protein binding
groupIII	12706686	12732686	No	n	ENSGACG00000017050	-	binding
groupIII	12706686	12732686	No	n	ENSGACG00000017052	-	protein binding
groupIII	12706686	12732686	No	n	ENSGACG00000017045	-	binding
groupIV	4779983	4817541	No	bg	-	-	-
groupIV	5928008	5941366	No	n	ENSGACG00000017204	FGF13	growth factor activity
groupIV	6766536	6832536	No	psR	ENSGACG00000017466	TNMD	-
groupIV	7048628	7124333	No	psL	-	-	-
groupIV	7919856	7938856	No	bg	ENSGACG00000017524	SFXN5 (2 of 2)	transmembrane transport, cation transport, membrane, cation transmembrane transporter activity
groupIV	7919856	7938856	No	bg	ENSGACG00000017521	SMYD5	protein binding
groupIV	8515346	8529697	No	psR	ENSGACG00000017689	TSC22D3	regulation of transcription, DNA-dependent, sequence-specific DNA binding transcription factor activity
groupIV	8559406	9007176	No	psR	ENSGACG00000017692	PCDH11Y	cell adhesion, homophilic cell adhesion, membrane, plasma membrane, calcium ion binding
groupIV	8559406	9007176	No	psR	ENSGACG00000017694	KLHL4	protein binding
groupIV	9078127	9110127	No	bg	ENSGACG00000017717	-	transport, ion transport, membrane, integral to membrane, postsynaptic membrane, extracellular ligand-gated ion channel activity
groupIV	9078127	9110127	No	bg	ENSGACG00000017720	CHRNB3 (1 of 2)	transport, ion transport, membrane, integral to membrane, postsynaptic membrane, extracellular ligand-gated ion channel activity
groupIV	9326969	9401969	No	psR	ENSGACG00000017836	-	protein binding
groupIV	9326969	9401969	No	psR	ENSGACG00000017837	SLC43A3 (2 of 2)	-
groupIV	9326969	9401969	No	psR	ENSGACG00000017843	-	-
groupIV	9326969	9401969	No	psR	ENSGACG00000017844	-	intracellular, zinc ion binding
groupIV	9326969	9401969	No	psR	ENSGACG00000017848	-	-
groupIV	9326969	9401969	No	psR	ENSGACG00000017846	CTNND1 (2 of 2)	binding, protein binding
groupIV	10319718	10351718	No	bg	ENSGACG00000017928	ETF1	-
groupIV	11551794	11606794	No	n	-	-	-

groupIV	11883990	11906990	No	bg	ENSGACG00000018209	NR3C1 (1 of 2)	regulation of transcription, DNA-dependent, steroid hormone mediated signaling pathway, glucocorticoid receptor signaling pathway, glucocorticoid mediated signaling pathway, response to dexamethasone stimulus, response to cortisol stimulus, response to glucocorticoid stimulus, calcium ion import, embryo development, positive regulation of transcription, DNA-dependent, nucleus, cytoplasm, sequence-specific DNA binding transcription factor activity, steroid hormone receptor activity, DNA binding, zinc ion binding, steroid binding, sequence-specific DNA binding, glucocorticoid receptor activity
groupIV	11883990	11906990	No	bg	ENSGACG00000018211	KCTD16 (2 of 2)	potassium ion transport, membrane, voltage-gated potassium channel complex, protein binding, voltage-gated potassium channel activity
groupIV	11951841	12001583	No	bg	ENSGACG00000018221	FGF16	pectoral fin morphogenesis, cell proliferation, growth factor activity
groupIV	11951841	12001583	No	bg	ENSGACG00000018218	ATRX	nucleic acid binding, DNA binding, zinc ion binding, ATP binding, protein binding, helicase activity
groupIV	12036368	12058368	No	bg	-	-	-
groupIV	13316184	13329223	No	psL	-	-	-
groupIV	13502481	13573820	No	psR	ENSGACG00000018406	LRRTM2	protein binding
groupIV	13502481	13573820	No	psR	ENSGACG00000018391	CTNNA1	cell adhesion, actin cytoskeleton, structural molecule activity
groupIV	13807552	13874552	No	psR	ENSGACG00000018407	SNCB	dopaminergic neuron differentiation, larval locomotory behavior
groupIV	13807552	13874552	No	psR	ENSGACG00000018408	CDHR2	cell adhesion, homophilic cell adhesion, membrane, plasma membrane, calcium ion binding
groupIV	13807552	13874552	No	psR	ENSGACG00000018413	RNF44	zinc ion binding, protein binding
groupIV	13807552	13874552	No	psR	ENSGACG00000018417	FAF2	-
groupIV	13936707	13961395	No	n	-	-	-
groupIV	17316716	17408198	No	psL	-	-	-
groupIV	17509167	17614167	No	psR	-	-	-
groupIV	23176499	23230500	No	psL	ENSGACG00000019249	AEBP2 (2 of 2)	intracellular, zinc ion binding
groupIV	23176499	23230500	No	psL	ENSGACG00000019242	COG5	-
groupIV	23176499	23230500	No	psL	ENSGACG00000019248	NDUFA5	respiratory electron transport chain, mitochondrial inner membrane, oxidoreductase activity, acting on NADH or NADPH
groupIV	23176499	23230500	No	psL	ENSGACG00000019253	PLEKHA5 (2 of 2)	protein binding
groupIV	25014649	25038649	No	n	-	-	-
groupIV	27689358	27743358	No	psR	-	-	-
groupIV	27764840	27803117	No	n	ENSGACG00000019633	MYOT	protein binding
groupIV	27764840	27803117	No	n	ENSGACG00000019640	-	protein phosphorylation, ATP binding, protein kinase activity, transferase activity, transferring phosphorus-containing groups, protein serine/threonine kinase activity, small GTPase regulator activity
groupIX	3275713	3309713	No	psR	ENSGACG00000016386	VEGFC	sprouting angiogenesis, lymphangiogenesis, angiogenesis, vascular endothelial growth factor receptor signaling pathway, endoderm development, membrane, growth factor activity, vascular endothelial growth factor receptor binding
groupIX	3513100	3527109	No	bg	-	-	-
groupIX	3529157	3584157	No	bg	-	-	-
groupIX	3816789	3831789	No	n	ENSGACG00000016390	ODZ3 (1 of 2)	signal transduction, integral to membrane, calcium ion binding, catalytic activity, protein binding
groupIX	8103529	8125354	No	bg	ENSGACG00000017501	AIMP1 (1 of 2)	tRNA binding
groupIX	8103529	8125354	No	bg	ENSGACG00000017502	TBCK	protein phosphorylation, regulation of Rab GTPase activity, intracellular, ATP binding, protein kinase activity, transferase activity, transferring phosphorus-containing groups, Rab GTPase activator activity, protein serine/threonine kinase activity
groupIX	8988452	9028452	No	psL	-	-	-
groupIX	9846445	9876486	No	psL	ENSGACG00000018071	GRIA2 (2 of 2)	transport, membrane, outer membrane-bounded periplasmic space, transporter activity, ionotropic glutamate receptor activity, extracellular-glutamate-gated ion channel activity
groupIX	11604130	11937922	No	psL	ENSGACG00000018410	PCDH18 (1 of 2)	cell adhesion, homophilic cell adhesion, epiboly involved in gastrulation with mouth forming second, calcium-dependent cell-cell adhesion, cell migration involved in gastrulation, membrane, plasma membrane, calcium ion binding
groupIX	11604130	11937922	No	psL	ENSGACG00000018409	PCDH10 (2 of 2)	cell adhesion, homophilic cell adhesion, membrane, plasma membrane, calcium ion binding
groupIX	12040164	12118165	No	psL	-	-	-
groupIX	12198878	12253446	No	psL	ENSGACG00000018448	-	-
groupIX	12198878	12253446	No	psL	ENSGACG00000018451	UNC93B1	-
groupIX	12198878	12253446	No	psL	ENSGACG00000018452	TIMM10	protein targeting to mitochondrion, protein import into mitochondrial inner membrane, mitochondrial intermembrane space protein transporter complex

groupIX	12198878	12253446	No	psL	ENSGACG00000018434	-	protein binding
groupIX	12198878	12253446	No	psL	ENSGACG00000018436	-	-
groupIX	12281856	12312856	No	psL	ENSGACG00000018459	SLC43A3 (1 of 2)	G-protein coupled receptor signaling pathway, transmembrane transport, integral to membrane
groupIX	12281856	12312856	No	psL	ENSGACG00000018463	-	protein dimerization activity
groupIX	12281856	12312856	No	psL	ENSGACG00000018465	CCRN4L (2 of 2)	-
groupIX	12281856	12312856	No	psL	ENSGACG00000018469	ELF2 (1 of 2)	regulation of transcription, DNA-dependent, sequence-specific DNA binding transcription factor activity, sequence-specific DNA binding
groupIX	12953788	13006788	No	bg	ENSGACG00000018535	PSMD12	protein binding
groupIX	12953788	13006788	No	bg	ENSGACG00000018530	-	nucleic acid binding, zinc ion binding
groupIX	13878722	13889722	No	psL	-	-	-
groupIX	13908576	13937576	No	psR	ENSGACG00000018691	PDCD4	-
groupIX	13908576	13937576	No	psR	ENSGACG00000018706	RBM20 (2 of 2)	-
groupIX	13908576	13937576	No	psR	ENSGACG00000018688	SHOC2	protein binding
groupIX	17192703	17229094	No	psR	-	-	-
groupIX	17347136	17385168	No	n	ENSGACG00000019412	ADAP2 (2 of 2)	regulation of ARF GTPase activity, zinc ion binding, protein binding, ARF GTPase activator activity
groupIX	17347136	17385168	No	n	ENSGACG00000019419	TEFM	-
groupIX	17347136	17385168	No	n	ENSGACG00000019420	-	-
groupIX	17347136	17385168	No	n	ENSGACG00000019410	RHOT1 (2 of 2)	small GTPase mediated signal transduction, protein transport, intracellular, calcium ion binding, GTP binding
groupV	4797325	4810325	No	n	ENSGACG00000004164	KCTD2	potassium ion transport, membrane, voltage-gated potassium channel complex, protein binding, voltage-gated potassium channel activity
groupV	4797325	4810325	No	n	ENSGACG00000004150	SLC16A5 (2 of 2)	transmembrane transport, integral to membrane
groupV	7309790	7346790	No	psL	-	-	-
groupV	9469391	9492778	No	psR	ENSGACG00000007239	PAQR4 (2 of 2)	integral to membrane
groupV	9469391	9492778	No	psR	ENSGACG00000007245	PELO	-
groupV	9469391	9492778	No	psR	ENSGACG00000007364	-	hydrolase activity
groupV	9469391	9492778	No	psR	ENSGACG00000007397	ATP2A1 (1 of 2)	calcium ion transport, metabolic process, ATP biosynthetic process, cation transport, membrane, integral to membrane, ATP binding, metal ion binding, catalytic activity, nucleotide binding, ATPase activity, coupled to transmembrane movement of ions, phosphorylative mechanism, calcium-transporting ATPase activity
groupVI	10549302	10562302	No	psL	ENSGACG00000009043	PCSK2	proteolysis, serine-type endopeptidase activity
groupVI	10549302	10562302	No	psL	ENSGACG00000009101	BFSP1	-
groupVI	13120817	13130817	No	bg	-	-	-
groupVII	6600254	6621254	No	psR	ENSGACG00000019575	LGI2 (1 of 2)	carbohydrate metabolic process, catalytic activity, protein binding, carbohydrate binding
groupVII	6600254	6621254	No	psR	ENSGACG00000019587	SEPSECS	transferase activity
groupVII	12535703	12560228	No	psL	ENSGACG00000020160	SKA2L	-
groupVII	12535703	12560228	No	psL	ENSGACG00000021295	-	-
groupVII	12535703	12560228	No	psL	ENSGACG00000021437	-	-
groupVII	12535703	12560228	No	psL	ENSGACG00000020161	TIPRL	-
groupVII	13006322	13023322	No	n	ENSGACG00000020185	IGSF9B (1 of 4)	protein binding, protein binding
groupVII	13188990	13212990	No	psL	ENSGACG00000020189	IGSF5 (1 of 2)	protein binding
groupVII	13269871	13280871	No	psL	ENSGACG00000020192	-	carbohydrate metabolic process, beta-galactosidase complex, hydrolase activity, hydrolyzing O-glycosyl compounds, beta-galactosidase activity
groupVII	13269871	13280871	No	psL	ENSGACG00000020193	STT3A	protein glycosylation, membrane, oligosaccharyl transferase activity, protein glycosylation, membrane, oligosaccharyl transferase activity
groupVII	15782798	15839798	No	psR	ENSGACG00000020293	GABRQ	transport, ion transport, membrane, integral to membrane, postsynaptic membrane, extracellular ligand-gated ion channel activity
groupVII	15782798	15839798	No	psR	ENSGACG00000020294	VSIG1	protein binding
groupVII	15782798	15839798	No	psR	ENSGACG00000020296	MPP1	protein binding
groupVII	15782798	15839798	No	psR	ENSGACG00000020297	VAMP2	vesicle-mediated transport, integral to membrane, vesicle-mediated transport, integral to membrane, cleavage furrow, vesicle, plasma membrane

groupVII	15782798	15839798	No	psR	ENSGACG00000020295	TMLHE	oxidation-reduction process, carnitine biosynthetic process, iron ion binding, oxidoreductase activity, L-ascorbic acid binding, trimethyllysine dioxygenase activity
groupVII	16470769	16529479	No	n	ENSGACG00000020311	-	protein binding, protein binding
groupVII	16470769	16529479	No	n	ENSGACG00000020312	ROBO2 (2 of 2)	protein binding, protein binding
groupVII	16575725	16593763	No	-	-	-	-
groupVII	16647076	16701076	No	psL	-	-	-
groupVII	16888482	16932482	No	bg	ENSGACG00000020319	NPM1	nucleic acid binding, hemopoiesis, nucleolus, nucleic acid binding
groupVII	16888482	16932482	No	bg	ENSGACG00000020320	CLDN7 (1 of 2)	membrane, tight junction, structural molecule activity
groupVII	16888482	16932482	No	bg	ENSGACG00000020318	KCNIP1 (2 of 2)	calcium ion binding
groupVII	16888482	16932482	No	bg	ENSGACG00000020321	FGF11 (2 of 2)	growth factor activity
groupVII	16939303	16950303	No	psR	-	-	-
groupVII	17868332	17907332	No	psR	ENSGACG00000020391	GLTPD2 (1 of 2)	-
groupVII	17868332	17907332	No	psR	ENSGACG00000020390	ATP1B2 (1 of 2)	potassium ion transport, sodium ion transport, ATP biosynthetic process, membrane, sodium:potassium-exchanging ATPase activity, potassium ion transport, sodium ion transport, ATP biosynthetic process, inner ear morphogenesis, otolith development, startle response, posterior lateral line neuromast hair cell differentiation, heart development, membrane, sodium:potassium-exchanging ATPase activity
groupVII	17868332	17907332	No	psR	ENSGACG00000020392	CHRNE	transport, ion transport, membrane, integral to membrane, postsynaptic membrane, extracellular ligand-gated ion channel activity, transport, ion transport, membrane, integral to membrane, postsynaptic membrane, extracellular ligand-gated ion channel activity
groupVII	17868332	17907332	No	psR	ENSGACG00000020393	PLD2	cell communication, metabolic process, phosphatidylinositol binding, catalytic activity, protein binding
groupVII	18008902	18113902	No	psR	ENSGACG00000020403	-	-
groupVII	18008902	18113902	No	psR	ENSGACG00000020405	DPF2 (1 of 2)	intracellular, zinc ion binding, protein binding
groupVII	18008902	18113902	No	psR	ENSGACG00000020407	SIPA1	regulation of small GTPase mediated signal transduction, intracellular, protein binding, GTPase activator activity
groupVII	18008902	18113902	No	psR	ENSGACG00000020404	SPTBN2	actin binding, protein binding
groupVII	18008902	18113902	No	psR	ENSGACG00000020406	-	transmembrane transport, integral to membrane
groupVII	18128628	18159628	No	psR	ENSGACG00000020410	-	cobalamin transport, cobalamin binding
groupVII	18128628	18159628	No	psR	ENSGACG00000020411	HINFP	transcription, DNA-dependent, intracellular, nucleic acid binding, zinc ion binding, transcription, DNA-dependent, intracellular, nucleic acid binding, zinc ion binding
groupVII	18128628	18159628	No	psR	ENSGACG00000020409	ABCG4 (2 of 2)	membrane, ATP binding, nucleotide binding, ATPase activity, nucleoside-triphosphatase activity
groupVII	18128628	18159628	No	psR	ENSGACG00000020412	DPAGT1	integral to membrane, phospho-N-acetylmuramoyl-pentapeptide-transferase activity
groupVII	18128628	18159628	No	psR	ENSGACG00000020413	C2CD2L	-
groupVII	18186355	18222355	No	n	ENSGACG00000020416	ATF5	regulation of transcription, DNA-dependent, sequence-specific DNA binding transcription factor activity, DNA binding, sequence-specific DNA binding, protein dimerization activity
groupVII	18186355	18222355	No	n	ENSGACG00000020417	STK36	protein phosphorylation, cilium axoneme assembly, determination of left/right symmetry, dorsal/ventral pattern formation, smoothened signaling pathway, ATP binding, protein kinase activity, transferase activity, transferring phosphorus-containing groups, protein serine/threonine kinase activity
groupVII	18186355	18222355	No	n	ENSGACG00000020418	ADAMTS15 (2 of 2)	proteolysis, extracellular matrix, zinc ion binding, metalloendopeptidase activity
groupVII	18186355	18222355	No	n	ENSGACG00000020415	-	protein binding
groupVII	18302840	18345846	No	psR	ENSGACG00000022227	-	-
groupVII	18302840	18345846	No	psR	ENSGACG00000021426	-	-
groupVII	18302840	18345846	No	psR	ENSGACG00000022268	-	-
groupVII	18450084	18473084	No	psL	ENSGACG00000020430	CPEB4 (1 of 2)	nucleic acid binding, nucleic acid binding
groupVII	19232382	19266382	No	-	ENSGACG00000020492	MRPS17	translation, intracellular, ribosome, structural constituent of ribosome
groupVII	19232382	19266382	No	-	ENSGACG00000020493	GBAS (2 of 2)	metabolic process, transferase activity, folic acid binding
groupVII	19232382	19266382	No	-	ENSGACG00000020494	CCT6A	protein folding, cellular protein metabolic process, ATP binding, unfolded protein binding, protein folding, cellular protein metabolic process, ATP binding, unfolded protein binding
groupVII	19232382	19266382	No	-	ENSGACG00000022599	-	-
groupVII	19232382	19266382	No	-	ENSGACG00000022586	-	-
groupVII	19232382	19266382	No	-	ENSGACG00000022642	-	-

groupVII	19232382	19266382	No	-	ENSGACG00000020495	SUMF2	binding
groupVII	19232382	19266382	No	-	ENSGACG00000020496	-	-
groupVII	20904868	20939868	No	psR	ENSGACG00000020596	PROSER1 (2 of 2)	-
groupVII	20904868	20939868	No	psR	ENSGACG00000020597	PROSER1 (1 of 2)	-
groupVII	20904868	20939868	No	psR	ENSGACG00000020598	STOML3 (1 of 2)	-
groupVII	20904868	20939868	No	psR	ENSGACG00000020595	VPS36	-
groupVII	20904868	20939868	No	psR	ENSGACG00000020599	MTUS2 (2 of 2)	-
groupVII	21849090	21881090	No	psR	ENSGACG00000020652	-	carbohydrate metabolic process, pyridoxal phosphate binding, phosphorylase activity
groupVII	21849090	21881090	No	psR	ENSGACG00000020653	-	transmembrane transport, integral to membrane, transmembrane transporter activity, transmembrane transport, integral to membrane, transmembrane transporter activity
groupVII	22034155	22051155	No	bg	ENSGACG00000020659	DNAJC18	methylation, nucleic acid binding, methyltransferase activity, heat shock protein binding
groupVII	22034155	22051155	No	bg	ENSGACG00000020658	-	hydrolase activity, hydrolase activity
groupVII	22057521	22108982	No	psR	ENSGACG00000020661	TMEM45B	-
groupVII	22057521	22108982	No	psR	ENSGACG00000020660	BRD8 (1 of 2)	protein binding
groupVII	22057521	22108982	No	psR	ENSGACG00000020662	NFRKB	-
groupVII	22057521	22108982	No	psR	ENSGACG00000020663	RYR1 (2 of 2)	oxidation-reduction process, ion transport, transmembrane transport, calcium ion transmembrane transport, cellular calcium ion homeostasis, membrane, integral to membrane, protein binding, ion channel activity, calcium channel activity, ryanodine-sensitive calcium-release channel activity, oxidoreductase activity, acting on paired donors, with incorporation or reduction of molecular oxygen, reduced pteridine as one donor, and incorporation of one atom of oxygen
groupVII	24162977	24288546	No	psR	ENSGACG00000020764	ALKBH4	-
groupVII	24162977	24288546	No	psR	ENSGACG00000020765	LRWD1	protein binding
groupVII	24162977	24288546	No	psR	ENSGACG00000020766	-	signal transduction, peptidyl-histidine phosphorylation, ATP binding, two-component sensor activity, protein histidine kinase activity, signal transduction, peptidyl-histidine phosphorylation, ATP binding, two-component sensor activity, protein histidine kinase activity
groupVII	24162977	24288546	No	psR	ENSGACG00000020767	ORAI2	-
groupVII	24162977	24288546	No	psR	ENSGACG00000020770	HTR3A	transport, ion transport, membrane, integral to membrane, postsynaptic membrane, extracellular ligand-gated ion channel activity, transport, ion transport, membrane, integral to membrane, postsynaptic membrane, extracellular ligand-gated ion channel activity, transport, ion transport, membrane, integral to membrane, postsynaptic membrane, extracellular ligand-gated ion channel activity
groupVII	24162977	24288546	No	psR	ENSGACG00000020762	MCAM (1 of 2)	protein binding
groupVII	24162977	24288546	No	psR	ENSGACG00000020763	CBL (2 of 2)	cell surface receptor signaling pathway, nucleus, signal transducer activity, zinc ion binding, protein binding
groupVII	24162977	24288546	No	psR	ENSGACG00000020768	USP28	ubiquitin-dependent protein catabolic process, ubiquitin thiolesterase activity
groupVII	24162977	24288546	No	psR	ENSGACG00000020769	HTR3B	transport, ion transport, membrane, integral to membrane, postsynaptic membrane, extracellular ligand-gated ion channel activity
groupVII	24162977	24288546	No	psR	ENSGACG00000020771	ZBTB16 (1 of 2)	intracellular, zinc ion binding, protein binding
groupVIII	3592495	3634762	No	psR	ENSGACG00000005009	FOXQ1	regulation of transcription, DNA-dependent, sequence-specific DNA binding transcription factor activity, sequence-specific DNA binding
groupVIII	3592495	3634762	No	psR	ENSGACG00000005011	-	regulation of transcription, DNA-dependent, sequence-specific DNA binding transcription factor activity, sequence-specific DNA binding
groupVIII	3592495	3634762	No	psR	ENSGACG00000005016	-	regulation of transcription, DNA-dependent, sequence-specific DNA binding transcription factor activity, sequence-specific DNA binding
groupVIII	3592495	3634762	No	psR	ENSGACG00000005020	GMDS	cellular metabolic process, GDP-mannose metabolic process, retina layer formation, dorsal motor nucleus of vagus nerve development, vagus nerve development, Notch signaling pathway, intracellular, oxidoreductase activity, catalytic activity, coenzyme binding, GDP-mannose 4, 6-dehydratase activity, fucosyltransferase activity
groupVIII	5728988	5755416	No	psR	-	-	-
groupVIII	12106496	12176892	No	bg	-	-	-
groupVIII	12334932	12351145	No	n	-	-	-
groupX	4988182	5006182	No	psR	-	-	-
groupX	6212410	6240410	No	psR	ENSGACG00000004037	GPR20 (2 of 2)	G-protein coupled receptor signaling pathway, integral to membrane
groupX	6212410	6240410	No	psR	ENSGACG00000004039	C16orf80 (1 of 3)	-
groupX	6212410	6240410	No	psR	ENSGACG00000004032	SLC45A4 (1 of 2)	transmembrane transport, integral to membrane

groupX	6278406	6351671	No	psL	ENSGACG00000004072	-	intracellular, zinc ion binding
groupX	6278406	6351671	No	psL	ENSGACG00000004076	SLC39A4	transmembrane transport, metal ion transport, membrane, metal ion transmembrane transporter activity
groupX	7547359	7575359	No	n	ENSGACG00000005059	RUNX1T1	regulation of transcription, DNA-dependent, sequence-specific DNA binding transcription factor activity, zinc ion binding
groupX	7593285	7655931	No	psR	-	-	-
groupX	7799350	7817350	No	psR	ENSGACG00000005164	ATP6V1C1 (1 of 2)	ATP hydrolysis coupled proton transport, proton-transporting V-type ATPase, V1 domain, hydrolase activity, acting on acid anhydrides, catalyzing transmembrane movement of substances
groupX	7799350	7817350	No	psR	ENSGACG00000005147	SPIRE1 (2 of 2)	protein binding
groupX	7838462	7864487	No	psL	ENSGACG00000005193	ADNP2	regulation of transcription, DNA-dependent, intracellular, sequence-specific DNA binding transcription factor activity, zinc ion binding, sequence-specific DNA binding, protein binding
groupX	7838462	7864487	No	psL	ENSGACG00000005197	GGCT	-
groupX	7838462	7864487	No	psL	ENSGACG00000005220	ANKIB1 (2 of 2)	zinc ion binding, protein binding
groupX	7838462	7864487	No	psL	ENSGACG00000005201	SNRPD1	heart development
groupX	8479060	8493060	No	psR	ENSGACG00000005895	GABBR2	G-protein coupled receptor signaling pathway, integral to membrane, G-protein coupled receptor activity
groupX	8809293	8851859	No	psR	ENSGACG00000006123	-	binding
groupX	8809293	8851859	No	psR	ENSGACG00000006108	RAPGEF5 (2 of 2)	intracellular signal transduction, small GTPase mediated signal transduction, regulation of small GTPase mediated signal transduction, intracellular, guanyl-nucleotide exchange factor activity
groupX	9306768	9337768	No	psR	ENSGACG00000006652	-	protein binding
groupXI	2178216	2205216	No	psL	ENSGACG00000005784	AOC2	oxidation-reduction process, amine metabolic process, copper ion binding, primary amine oxidase activity, quinone binding
groupXI	2178216	2205216	No	psL	ENSGACG00000005803	-	-
groupXI	2178216	2205216	No	psL	ENSGACG00000005761	-	proteasome activator complex
groupXI	5301185	5324185	No	bg	-	-	-
groupXI	8598827	8620930	No	n	-	-	-
groupXI	9111146	9134146	No	psR	ENSGACG00000011028	USP36	-
groupXI	9111146	9134146	No	psR	ENSGACG00000011059	LGALS3BP	membrane, protein binding, scavenger receptor activity
groupXI	9111146	9134146	No	psR	ENSGACG00000011036	TIMP2 (1 of 2)	metalloendopeptidase inhibitor activity
groupXI	10850527	10864527	No	psL	ENSGACG00000011808	E4F1	intracellular, nucleic acid binding, DNA binding, zinc ion binding
groupXI	10850527	10864527	No	psL	ENSGACG00000011814	NARFL	-
groupXI	10850527	10864527	No	psL	ENSGACG00000011858	HAGH	hydrolase activity
groupXI	15769060	15788060	No	n	ENSGACG00000014700	STX1B	intracellular protein transport, vesicle-mediated transport, membrane, protein binding, SNAP receptor activity
groupXII	3669944	3691945	No	psR	ENSGACG00000004196	TNNC2 (2 of 2)	calcium ion binding
groupXII	3669944	3691945	No	psR	ENSGACG00000004193	-	phosphatidylinositol phosphorylation, phosphatidylinositol-mediated signaling, phosphatidylinositol 3-kinase complex, binding, protein binding, transferase activity, transferring phosphorus-containing groups, 1-phosphatidylinositol-3-kinase activity, phosphotransferase activity, alcohol group as acceptor
groupXII	6190053	6232053	No	psR	-	-	-
groupXII	8633693	8651913	No	psR	ENSGACG00000006798	ZNF362 (1 of 2)	intracellular, zinc ion binding
groupXII	8742616	8778792	No	n	-	-	-
groupXII	9151507	9172507	No	psL	ENSGACG00000007105	PEX14	membrane, peroxisome,
groupXII	12193678	12233238	No	n	ENSGACG00000009813	UBE2T	acid-amino acid ligase activity
groupXII	12193678	12233238	No	n	ENSGACG00000009831	ETV7	regulation of transcription, DNA-dependent, nucleus, sequence-specific DNA binding transcription factor activity, sequence-specific DNA binding, protein binding
groupXII	12193678	12233238	No	n	ENSGACG00000009806	LGR6	G-protein coupled receptor signaling pathway, integral to membrane, protein binding
groupXII	12382635	12406635	No	psR	ENSGACG00000022656	-	-
groupXII	12382635	12406635	No	psR	ENSGACG00000022657	-	-
groupXII	12382635	12406635	No	psR	ENSGACG00000009922	USP21	ubiquitin-dependent protein catabolic process, ubiquitin thiolesterase activity
groupXII	12382635	12406635	No	psR	ENSGACG00000009903	UBA1	cellular protein modification process, ATP binding, catalytic activity, small protein activating enzyme activity
groupXII	12382635	12406635	No	psR	ENSGACG00000009911	-	regulation of Rho protein signal transduction, intracellular, protein binding, Rho guanyl-nucleotide exchange factor activity

groupXII	13083520	13091525	No	psL	ENSGACG00000010405	LAMA5	signal transduction, cell adhesion, regulation of cell migration, regulation of cell adhesion, regulation of embryonic development, pectoral fin morphogenesis, fin development, fin morphogenesis, medial fin morphogenesis, protein localization, cell-cell adhesion, membrane, extracellular matrix, laminin-1 complex, basement membrane, signal transducer activity, calcium ion binding, binding, receptor binding, protein binding
groupXIII	4069584	4168514	No	psR	ENSGACG00000005772	-	proteolysis, intracellular, cysteine-type endopeptidase activity, aminopeptidase activity
groupXIII	4069584	4168514	No	psR	ENSGACG00000005837	FAHD2B	metabolic process, catalytic activity
groupXIII	4069584	4168514	No	psR	ENSGACG00000005726	PCNA	regulation of DNA replication, response to bacterium, response to stress, PCNA complex, nucleus, DNA binding, DNA polymerase processivity factor activity
groupXIII	4069584	4168514	No	psR	ENSGACG00000005887	GPAT2	metabolic process
groupXIII	5230249	5258542	No	n	-	-	-
groupXIII	5806669	5827681	No	n	-	-	-
groupXIII	6599200	6662200	No	psR	ENSGACG00000007345	GRID2	transport, membrane, outer membrane-bounded periplasmic space, transporter activity, ionotropic glutamate receptor activity, extracellular-glutamate-gated ion channel activity
groupXIII	7937987	7952987	No	psR	ENSGACG00000007806	WDR31	protein binding
groupXIII	7937987	7952987	No	psR	ENSGACG00000007821	G-Q-(1-of-3)	signal transduction, G-protein coupled receptor signaling pathway, signal transducer activity, guanyl nucleotide binding
groupXIII	7953754	8183975	No	psR	ENSGACG00000007973	FOXB2	-
groupXIII	7953754	8183975	No	psR	ENSGACG00000007980	-	-
groupXIII	7953754	8183975	No	psR	ENSGACG00000007984	PRUNE2 (2 of 2)	-
groupXIII	7953754	8183975	No	psR	ENSGACG00000007993	GCNT1	membrane, acetylglucosaminyltransferase activity
groupXIII	7953754	8183975	No	psR	ENSGACG00000008000	RFK	riboflavin biosynthetic process, riboflavin kinase activity
groupXIII	7953754	8183975	No	psR	ENSGACG00000008074	KIF24	microtubule-based movement, ATP binding, protein binding, microtubule motor activity
groupXIII	7953754	8183975	No	psR	ENSGACG00000008080	RFESD (1 of 2)	oxidation-reduction process, oxidoreductase activity, 2 iron, 2 sulfur cluster binding
groupXIII	7953754	8183975	No	psR	ENSGACG00000008097	PSMD9	protein binding
groupXIII	7953754	8183975	No	psR	ENSGACG00000008103	-	DNA binding, zinc ion binding
groupXIII	7953754	8183975	No	psR	ENSGACG00000007821	G-Q-(1-of-3)	signal transduction, G-protein coupled receptor signaling pathway, signal transducer activity, guanyl nucleotide binding
groupXIII	7953754	8183975	No	psR	ENSGACG00000007900	-	signal transduction, G-protein coupled receptor signaling pathway, signal transducer activity, guanyl nucleotide binding
groupXIII	7953754	8183975	No	psR	ENSGACG00000008043	UBAP2 (2 of 2)	protein binding
groupXIII	7953754	8183975	No	psR	ENSGACG00000008083	GTF2H3	regulation of transcription, DNA-dependent, DNA repair, core TFIIH complex
groupXIII	7953754	8183975	No	psR	ENSGACG00000007925	VPS13A	protein localization
groupXIII	7953754	8183975	No	psR	ENSGACG00000008029	PCSK5	proteolysis, regulation of cell growth, extracellular region, protein binding, serine-type endopeptidase activity, insulin-like growth factor binding
groupXIII	10120445	10196445	No	psR	ENSGACG00000010214	EDIL3	cell adhesion, calcium ion binding, protein binding
groupXIII	12480257	12544360	No	psR	-	-	-
groupXV	9672357	9797450	No	psR	ENSGACG00000010625	WDR20 (2 of 2)	protein binding
groupXV	9672357	9797450	No	psR	ENSGACG00000010657	ELAVL1 (1 of 2)	nucleic acid binding, RNA binding
groupXV	9672357	9797450	No	psR	ENSGACG00000010673	-	dicarboxylic acid transport, membrane, sodium:dicarboxylate symporter activity
groupXV	9672357	9797450	No	psR	ENSGACG00000010701	DIRAS1 (1 of 2)	signal transduction, GTP catabolic process, small GTPase mediated signal transduction, intracellular protein transport, nucleocytoplasmic transport, protein transport, intracellular, GTPase activity, GTP binding
groupXV	9672357	9797450	No	psR	ENSGACG00000010717	-	protein binding
groupXV	9672357	9797450	No	psR	ENSGACG00000010718	-	-
groupXV	9672357	9797450	No	psR	ENSGACG00000010724	-	defense response, DNA binding, protein binding
groupXV	9672357	9797450	No	psR	ENSGACG00000010727	-	-
groupXV	9672357	9797450	No	psR	ENSGACG00000010634	-	-
groupXV	9672357	9797450	No	psR	ENSGACG00000010688	REEP6	-
groupXV	9672357	9797450	No	psR	ENSGACG00000010706	GADD45B (2 of 2)	somitogenesis
groupXV	9672357	9797450	No	psR	ENSGACG00000010720	IGKC (24 of 24)	protein binding
groupXV	9672357	9797450	No	psR	ENSGACG00000010728	-	protein binding
groupXV	11640529	11669804	No	psR	ENSGACG00000011810	L2HGDH	oxidoreductase activity

groupXV	11640529	11669804	No	psR	ENSGACG00000011842	CDKL1	protein phosphorylation, ATP binding, protein kinase activity, transferase activity, transferring phosphorus-containing groups, protein serine/threonine kinase activity
groupXV	11640529	11669804	No	psR	ENSGACG00000011836	ATP5S	-
groupXV	11640529	11669804	No	psR	ENSGACG00000011850	MAP4K5	protein phosphorylation, ATP binding, protein kinase activity, transferase activity, transferring phosphorus-containing groups, protein serine/threonine kinase activity, small GTPase regulator activity
groupXV	14264687	14324687	No	psR	ENSGACG00000012967	MATN3 (2 of 2)	calcium ion binding, protein binding
groupXV	14264687	14324687	No	psR	ENSGACG00000012974	ODC1	catalytic activity
groupXV	14264687	14324687	No	psR	ENSGACG00000013000	NOL10	-
groupXV	14264687	14324687	No	psR	ENSGACG00000013004	ATP6V1C2	ATP hydrolysis coupled proton transport, proton-transporting V-type ATPase, V1 domain, hydrolase activity, acting on acid anhydrides, catalyzing transmembrane movement of substances
groupXV	14264687	14324687	No	psR	ENSGACG00000012959	WDR35	protein binding
groupXV	16008051	16059082	No	bg	ENSGACG00000013340	KCNK16	-
groupXV	16008051	16059082	No	bg	ENSGACG00000013319	RAB3GAP2	translation, tRNA aminoacylation for protein translation, cytoplasm, ATP binding, nucleotide binding, aminoacyl-tRNA ligase activity
groupXV	16008051	16059082	No	bg	ENSGACG00000013332	KCNK5 (1 of 2)	-
groupXV	16118354	16187408	No	psR	ENSGACG00000013366	DAAM2	vesicle-mediated transport, actin cytoskeleton organization, cellular component organization, membrane, binding, actin binding, Rho GTPase binding
groupXVI	3823972	3847046	No	psL	-	-	-
groupXVI	9255836	9318530	No	psR	ENSGACG00000004159	-	membrane, tight junction, structural molecule activity
groupXVI	9255836	9318530	No	psR	ENSGACG00000004162	-	membrane, tight junction, structural molecule activity
groupXVI	9255836	9318530	No	psR	ENSGACG00000004166	DNAJC3	translation, intracellular, ribosome, protein binding, structural constituent of ribosome, heat shock protein binding
groupXVI	9255836	9318530	No	psR	ENSGACG00000004126	ABCC4 (1 of 4)	transport, transmembrane transport, integral to membrane, ATP binding, nucleotide binding, ATPase activity, ATPase activity, coupled to transmembrane movement of substances, nucleoside-triphosphatase activity
groupXVI	9255836	9318530	No	psR	ENSGACG00000004153	CLDN10 (2 of 2)	membrane, tight junction, structural molecule activity, membrane, tight junction, structural molecule activity
groupXVI	9848880	9876880	No	n	ENSGACG00000004584	KIAA1715 (1 of 2)	-
groupXVI	9848880	9876880	No	n	ENSGACG00000004579	-	-
groupXVII	4954228	4982586	No	psR	ENSGACG00000006306	TUBB1	GTP catabolic process, microtubule-based process, protein polymerization, protein complex, microtubule, GTPase activity, GTP binding
groupXVII	4954228	4982586	No	psR	ENSGACG00000022571	-	-
groupXVII	4954228	4982586	No	psR	ENSGACG00000006335	SLMO2 (1 of 2)	-
groupXVII	4954228	4982586	No	psR	ENSGACG00000006354	AURKA	protein phosphorylation, ATP binding, protein kinase activity, transferase activity, transferring phosphorus-containing groups, protein serine/threonine kinase activity
groupXVII	4954228	4982586	No	psR	ENSGACG00000006379	SLC32A1 (1 of 2)	-
groupXVII	4954228	4982586	No	psR	ENSGACG00000006249	-	G-protein coupled receptor signaling pathway, signal transducer activity, guanyl nucleotide binding
groupXVII	6004396	6085437	No	n	ENSGACG00000007297	-	-
groupXVII	6004396	6085437	No	n	ENSGACG00000007304	-	-
groupXVII	6004396	6085437	No	n	ENSGACG00000007307	-	-
groupXVII	6004396	6085437	No	n	ENSGACG00000007311	-	-
groupXVII	6004396	6085437	No	n	ENSGACG00000007333	HRH1	G-protein coupled receptor signaling pathway, integral to membrane
groupXVII	6004396	6085437	No	n	ENSGACG00000007265	SLC6A1 (1 of 2)	neurotransmitter transport, integral to membrane, neurotransmitter:sodium symporter activity
groupXVII	6004396	6085437	No	n	ENSGACG00000007339	ATG7	autophagy, cytoplasm, catalytic activity
groupXVII	6113126	6133126	No	n	ENSGACG00000007347	VGLL4	-
groupXVII	6198066	6225066	No	n	ENSGACG00000007391	-	protein phosphorylation, ATP binding, protein kinase activity, transferase activity, transferring phosphorus-containing groups, protein serine/threonine kinase activity
groupXVII	6198066	6225066	No	n	ENSGACG00000007398	FOXJ3 (1 of 2)	regulation of transcription, DNA-dependent, sequence-specific DNA binding transcription factor activity, sequence-specific DNA binding
groupXVIII	1218091	1247566	No	psL	ENSGACG00000004653	-	-
groupXVIII	1218091	1247566	No	psL	ENSGACG00000004657	CNST (1 of 3)	-
groupXVIII	1218091	1247566	No	psL	ENSGACG00000004546	S-P25-(1-of-2)	vesicle-mediated transport, membrane, protein binding

groupXVIII	1218091	1247566	No	psL	ENSGACG00000004618	-	oxidation-reduction process, metal ion binding, protein binding, oxidoreductase activity, acting on single donors with incorporation of molecular oxygen, incorporation of two atoms of oxygen
groupXVIII	1218091	1247566	No	psL	ENSGACG00000004658	CNST (3 of 3)	-
groupXVIII	1445479	1482479	No	psR	ENSGACG00000004803	-	nucleic acid binding
groupXVIII	1445479	1482479	No	psR	ENSGACG00000004820	TDRD6	nucleic acid binding
groupXVIII	1445479	1482479	No	psR	ENSGACG00000004893	INTS9	-
groupXVIII	1445479	1482479	No	psR	ENSGACG00000004825	PLA2G7	-
groupXVIII	1445479	1482479	No	psR	ENSGACG00000004784	VSNL1 (1 of 2)	calcium ion binding
groupXVIII	1531272	1539933	No	bg	-	-	-
groupXVIII	8300041	8329296	No	psL	ENSGACG00000008721	-	signal transduction, keratinization, membrane, cytoplasm, signal transducer activity, actin binding, protein binding
groupXVIII	9029949	9044303	No	n	ENSGACG00000009366	PREP	proteolysis, serine-type peptidase activity, serine-type endopeptidase activity, , proteolysis, serine-type peptidase activity, serine-type endopeptidase activity
groupXX	949459	1007926	No	n	ENSGACG00000003524	OSR2	kidney development, negative regulation of transcription from RNA polymerase II promoter, pronephros development, intracellular, zinc ion binding
groupXX	949459	1007926	No	n	ENSGACG00000003526	VPS13B (3 of 3)	-
groupXX	4143873	4157873	No	psR	ENSGACG00000005481	NFATC1 (2 of 2)	regulation of transcription, DNA-dependent, nucleus, sequence-specific DNA binding transcription factor activity, protein binding
groupXX	10858034	10878034	No	n	ENSGACG00000009299	ABCB4	transport, transmembrane transport, integral to membrane, ATP binding, nucleotide binding, ATPase activity, ATPase activity, coupled to transmembrane movement of substances, nucleoside-triphosphatase activity
groupXX	10858034	10878034	No	n	ENSGACG00000009281	CROT (1 of 2)	-
groupXX	11339915	11367371	No	n	-	-	-
groupXX	11513416	11558416	No	psR	ENSGACG00000009790	-	-
groupXX	11513416	11558416	No	psR	ENSGACG00000009825	-	binding
groupXX	11513416	11558416	No	psR	ENSGACG00000009840	USP5	ubiquitin-dependent protein catabolic process, zinc ion binding, protein binding, ubiquitin thiolesterase activity
groupXX	11513416	11558416	No	psR	ENSGACG00000009793	COPS7A	protein binding
groupXX	11513416	11558416	No	psR	ENSGACG00000009832	-	protein binding
groupXX	11696205	11759226	No	n	ENSGACG00000010128	-	binding
groupXX	11696205	11759226	No	n	ENSGACG00000010169	TAPBPL	protein binding
groupXX	11696205	11759226	No	n	ENSGACG00000010179	VAMP1	vesicle-mediated transport, integral to membrane
groupXX	11696205	11759226	No	n	ENSGACG00000010193	MRPL51	-
groupXX	11696205	11759226	No	n	ENSGACG00000010195	PLEKHG6	regulation of Rho protein signal transduction, intracellular, Rho guanyl-nucleotide exchange factor activity
groupXX	11696205	11759226	No	n	ENSGACG00000010200	TNFRSF1A	signal transduction, binding, protein binding
groupXX	11696205	11759226	No	n	ENSGACG00000010125	RBP5	binding
groupXX	11696205	11759226	No	n	ENSGACG00000010132	C1R	proteolysis, calcium ion binding, catalytic activity, protein binding, serine-type endopeptidase activity
groupXX	12093612	12156612	No	psR	ENSGACG00000010597	ATG12	autophagic vacuole assembly, cytoplasm
groupXX	12093612	12156612	No	psR	ENSGACG00000010601	-	calcium ion binding
groupXX	12093612	12156612	No	psR	ENSGACG00000010614	-	protein binding
groupXX	12093612	12156612	No	psR	ENSGACG00000010622	CCDC106 (1 of 2)	-
groupXX	12093612	12156612	No	psR	ENSGACG00000010632	U2AF2	mRNA processing, nucleus, nucleic acid binding, RNA binding
groupXX	12093612	12156612	No	psR	ENSGACG00000010639	CACNG8 (2 of 2)	membrane
groupXX	12438628	12484628	No	psL	ENSGACG00000011464	PRPF31	nuclear mRNA splicing, via spliceosome, retina development in camera-type eye
groupXX	12438628	12484628	No	psL	ENSGACG00000011504	-	-
groupXX	12438628	12484628	No	psL	ENSGACG00000011489	CNOT3 (2 of 2)	regulation of transcription, DNA-dependent, nucleus, nucleotide binding
groupXX	12438628	12484628	No	psL	ENSGACG00000011507	-	signal transduction, filopodium assembly, protein binding, cytoskeletal adaptor activity, SH3 domain binding
groupXX	12506449	12533449	No	psL	ENSGACG00000011540	SPHK2	protein kinase C-activating G-protein coupled receptor signaling pathway, diacylglycerol kinase activity
groupXX	12506449	12533449	No	psL	ENSGACG00000011594	-	integral to membrane
groupXX	12506449	12533449	No	psL	ENSGACG00000011547	RPL18	translation, intracellular, ribosome, structural constituent of ribosome
groupXX	12506449	12533449	No	psL	ENSGACG00000011600	MYH14	regulation of transcription, DNA-dependent, myosin complex, sequence-specific DNA binding transcription factor activity, motor activity, ATP binding, sequence-specific DNA binding, nucleotide binding, protein binding

groupXX	14247752	14283752	No	n	ENSGACG00000012632	C3orf75	-
groupXX	14247752	14283752	No	n	ENSGACG00000012618	SCAP	integral to membrane, protein binding
groupXX	14247752	14283752	No	n	ENSGACG00000012641	-	-
groupXX	16432445	16480445	No	psR	-	-	-
groupXX	17944465	18042465	No	n	ENSGACG00000014023	POPDC3	membrane
groupXX	17944465	18042465	No	n	ENSGACG00000014027	CNIH4	intracellular signal transduction, membrane
groupXX	17944465	18042465	No	n	ENSGACG00000014036	WDR26 (2 of 2)	protein binding
groupXX	17944465	18042465	No	n	ENSGACG00000014009	-	protein binding
groupXX	17944465	18042465	No	n	ENSGACG00000014015	BVES	membrane
groupXX	17944465	18042465	No	n	ENSGACG00000014012	VEGFA (1 of 2)	blood vessel development, regulation of heart contraction, motor axon guidance, sprouting angiogenesis, vasculature development, artery morphogenesis, blood vessel endothelial cell proliferation involved in sprouting angiogenesis, regulation of angioblast cell migration involved in selective angioblast sprouting, angioblast cell migration from lateral mesoderm to midline, thyroid gland development, activation of phospholipase C activity, protein kinase B signaling cascade, angiogenesis, hemopoiesis, phosphorylation, vascular endothelial growth factor receptor signaling pathway, pronephros development, patterning of blood vessels, vasculogenesis, membrane, growth factor activity, protein binding
groupXXI	5490371	5539913	No	n	ENSGACG00000002473	-	-
groupXXI	5490371	5539913	No	n	ENSGACG00000002461	CNT-P2-(1-of-2)	cell adhesion
groupXXI	7544330	7566465	No	bg	ENSGACG00000003081	PAN3	protein phosphorylation, ATP binding, transferase activity, transferring phosphorus-containing groups, protein serine/threonine kinase activity
groupXXI	7544330	7566465	No	bg	ENSGACG00000003090	-	protein phosphorylation, transmembrane receptor protein tyrosine kinase signaling pathway, membrane, ATP binding, protein kinase activity, transferase activity, transferring phosphorus-containing groups, transmembrane receptor protein tyrosine kinase activity
groupXXI	7544330	7566465	No	bg	ENSGACG00000003094	-	protein phosphorylation, protein kinase activity, transferase activity, transferring phosphorus-containing groups
groupXXI	7544330	7566465	No	bg	ENSGACG00000003095	PRHOXNB	-
groupXXI	7544330	7566465	No	bg	ENSGACG00000003099	PDX1	regulation of transcription, DNA-dependent, pancreas development, determination of pancreatic left/right asymmetry, determination of liver left/right asymmetry, determination of digestive tract left/right asymmetry, endocrine pancreas development, nucleus, sequence-specific DNA binding transcription factor activity, sequence-specific DNA binding, protein binding

Type refers to categories inferred by signatures of selection: n reduced gene flow, bg background selection, psL adaptation to lake, psR adaptation to river.
